# Strategic timing for the decarbonization contributions by China’s photovoltaic manufacturers in global energy transition

**DOI:** 10.1038/s41467-026-76424-4

**Published:** 2026-08-06

**Authors:** Qingyang Lin, Chenglin Li, Kai Wang, Kai Fang, Meixuan Xin, Siqi Wang, Anqi Xu, Zhenci Xu, Yue Lu, Xiang Gao

**Affiliations:** 1 State Key Laboratory of Clean Energy Utilization, Zhejiang University, Hangzhou, China; 2 School of Public Affairs, Zhejiang University, Hangzhou, China; 3 Institute for Public Policy, Zhejiang University, Hangzhou, China; 4 Lab of Inclusive & Smart Governance for Urban-Rural Integration, Zhejiang University, Hangzhou, China; 5 School of Environment and Safety Engineering, Qingdao University of Science and Technology, Qingdao, China; 6 School of Economics and Management, Zhejiang Sci-Tech University, Hangzhou, China; 7 Department of Geography, The University of Hong Kong, Hong Kong, China; 8 School of International Trade and Economics, University of International Business and Economics, Beijing, China; 9 Dalian University of Technology, Dalian, China

**Keywords:** Carbon and energy, Climate-change mitigation

## Abstract

Photovoltaic (PV) technology is central to clean electricity and the Sustainable Development Goals. However, China’s PV manufacturing industry, which accounts for nearly two-thirds of global capacity, is often criticized for its high carbon emissions. Existing studies typically assign emission responsibility to manufacturers while attributing decarbonization benefits to power generation, overlooking upstream producers’ contributions. Here we propose a Producer Decarbonization Contribution framework to quantify these long-overlooked contributions at national and global scales. We show that China’s PV manufacturers contribute 31.4%-73.8% of total PV decarbonization in China and 27.9%-45.4% globally. We further identify a strategic timing, defined as the point at which the PV’s decarbonization contributions reach their maximum, occurring around 2035 in China under the Announced Pledges Scenario and 2030 globally under the Net Zero Emissions Scenario proposed by the International Energy Agency. These findings suggest maintaining supportive policies for China before 2035, while adopting more stringent measures globally after 2030 to avoid overproduction. The proposed framework that enables adaptive industrial policy design can be extended to other renewable energy technologies.

## Abstract (web summary)

This study quantifies the overlooked decarbonization contributions of photovoltaic manufacturers and identifies strategic timing around 2035 for China and 2030 globally, informing a shift from supportive to restrictive policies.

## Introduction

Renewable energy plays a critical role in achieving the Sustainable Development Goals (SDGs) concerning clean and affordable energy, as well as climate action^1–4^. Photovoltaics (PV) stand out as a promising clean energy source that supports both the development of renewable energy industries^5,6^ and net-zero goals^7–10^. The lifecycle of PV systems encompasses various stages, including manufacturing, transportation, construction, operation, and decommissioning^10,11^. The main emission reduction benefit of PV systems comes at the operation stage, as they generate green electricity in comparison with electricity mainly generated by fossil fuels. Most carbon emissions of PV systems come at the manufacturing phase^12–14^. Nevertheless, these carbon emissions are a necessary precursor to the decarbonization during operation stage, making the PV manufacturers part of the decarbonization contribution body^15–17^.

China has contributed 35.0% of the cumulative PV capacity and 44% of the newly installed PV capacity globally in 2022^18^. Furthermore, China – the world’s largest PV producer – holds a dominant share of global mass production for PV cells and modules at 84% in 2021, but its domestic demand for PV products only accounted for 36.4% of the world’s total^19,20^. The surplus has been exported to regions like Europe and North America^21^. Meanwhile, China has achieved domestic self-sufficiency (> 98%) for critical PV materials since 2020, including steel, aluminum, and PV glass^22^. These emphasize China’s influential role in guiding a net-zero transition through the PV manufacturing industry^23,24^.

China accounts for about one-third of global anthropogenic carbon emissions^6^. In response to climate change, China aims to peak its emissions before 2030 and achieve net-zero emissions before 2060^25^. While this strategy is crucial for addressing long-term environmental goals by prompting technological transitions, it, however, may pose substantial short-term challenges to various industries, including the PV manufacturing industry, as they normally produce most carbon emissions^26–28^. In practice, this involves setting carbon emissions or energy use ‘budgets’ at the regional, sectoral and corporate levels^29^. The PV industry, in particular, has a long production chain involving numerous upstream manufacturers labeled as high emitters^14,30,31^. In some cases, key upstream suppliers within the PV value chain (e.g., PV glass manufacturers) are not explicitly recognized as part of the PV industry and may therefore be subject to regulatory treatment that does not fully reflect their role in enabling downstream decarbonization. Stringent short-term policies may disrupt upstream manufacturers by constraining capacity expansion and increasing production costs^20,32–34^, thereby weakening incentives for innovation^35,36^, and ultimately hindering the development of the PV industry and even the energy transition^37^. These even undermine progress towards SDGs, including affordable and clean energy (SDG 7), responsible consumption and production (SDG 12), and climate action (SDG 13).

Existing studies on PV systems tend to assign emission responsibilities across the value chain, often attributing a substantial share to PV manufacturers while overlooking their role in enabling downstream decarbonization^17,38^. For example, prevailing accounting frameworks such as the Greenhouse Gas (GHG) Protocol treat emission reductions from products separately from the Scope 1, 2, and 3 inventories^39^, and do not explicitly incorporate them into core accounting boundaries^40^. If emission reductions are taken into account, producers may be associated with low or even zero emissions^41,42^. However, this perspective reflects merely a reduction in responsibility rather than an explicit recognition of contribution. This gap raises a fundamental question: how large are the decarbonization contributions of PV manufacturers? Furthermore, even if the decarbonization contributions of PV manufacturers have been recognized, they should not be assumed to increase indefinitely. This is because the avoided emissions enabled by PV depend on the carbon intensity of the electricity that PV replaces. As power systems become increasingly decarbonized, the marginal contribution of upcoming PV deployment may narrow. This raises a further question: how will the decarbonization contributions of PV manufacturers vary over time?

To close these gaps, this study makes an effort to show the overlooked contributions of the PV manufacturing industry to both China’s and global decarbonization progress. We propose a Producer Decarbonization Contribution (PDC) framework by quantifying the contributions of emission reduction that producers make over the lifecycle of the PV system by allocating a reasonable share of these contributions to the manufacturing stage using three alternative criteria: energy consumption, economic costs, and carbon emissions, respectively. We then introduce a concept of strategic timing, defined as the predicted time point when the decarbonization contributions from PV manufacturers reach their maximum. Our analysis takes into account future electricity production structure and deployment of PV under three scenarios released by the International Energy Agency (IEA)^6^: the Stated Policies Scenario (STEPS), the Announced Pledges Scenario (APS), and the Net Zero Emissions by 2050 Scenario (NZE). This is followed by a discussion of policy implications for supporting the PV manufacturing industry within China and globally. Given China’s important role in global PV manufacturing, findings derived from the China-specific context would have global implications for energy transition and can be extended to other renewables.

## Results

### Strategic timing for photovoltaic manufacturers’ decarbonization contributions in China

**Fig. 1:**
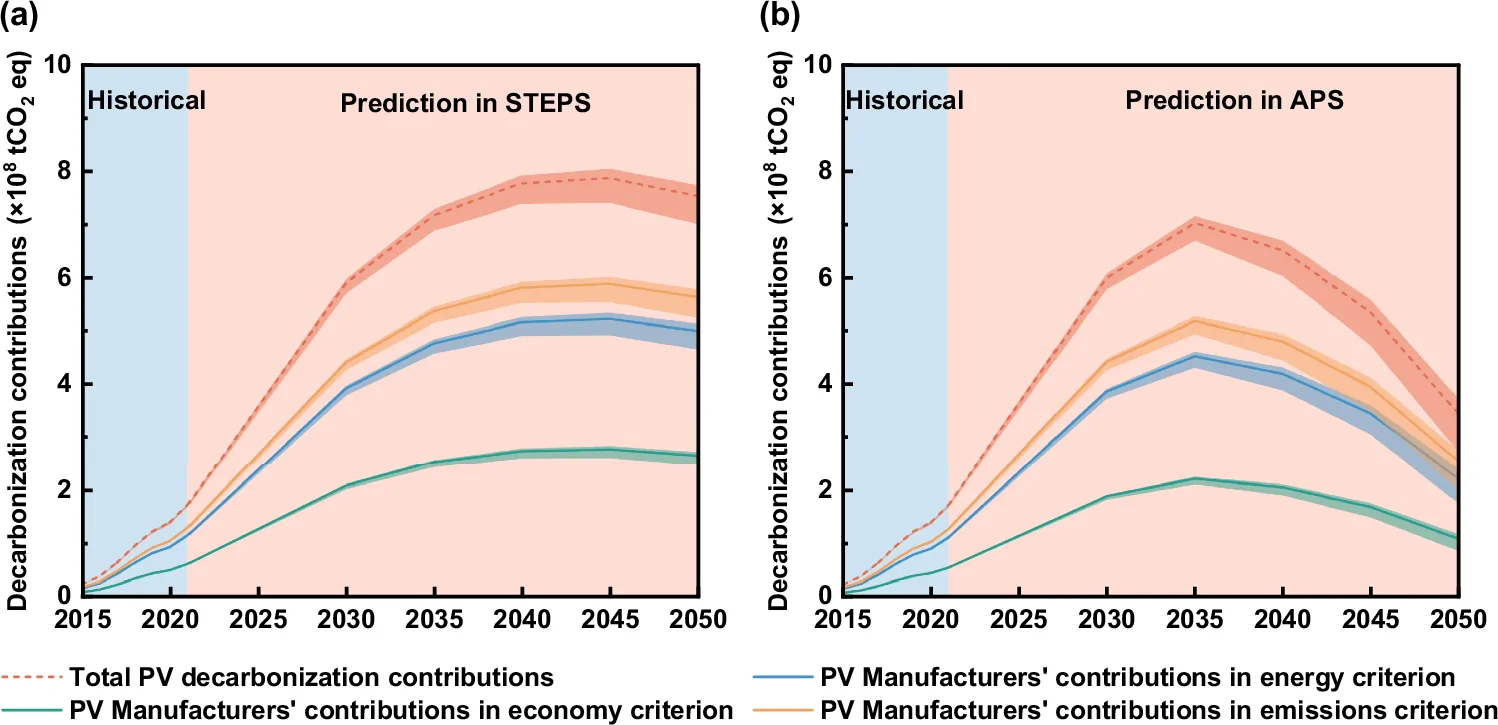
The decarbonization contributions and associated strategic timing of the photovoltaic (PV) manufacturers at China’s national level. **a** The decarbonization contributions under the Stated Policies Scenario (STEPS). **b** The decarbonization contributions under the Announced Pledges Scenario (APS).

PV-generated electricity has a carbon intensity of 16.94 gCO_2_ eq kWh^−1^ (grams of carbon dioxide equivalent per kilowatt-hour), much lower than the intensity of coal-fired electricity ( ~ 820 gCO_2_ eq kWh^−1^)^43^, providing a low-emission energy source within China’s energy structure (see “Methods” for detailed methodology). Payback time serves as a key parameter for assessing PV’s decarbonization contributions. The shorter the payback time is, the greater the assigned decarbonization contributions that PV produces. By evaluating the annual electricity output, revenue generation, and decarbonization contributions enabled by PV systems, we calculate the payback time required to offset lifecycle energy consumption, economic costs, and carbon emissions^44^ for a typical 1 MWp PV system operating for 30 years in China as an example. Specifically, the payback time for energy consumption (${T}_{{en}}$) is 2.7 years, for economic costs (${T}_{{eco}}$) is 8.2 years, and for carbon emissions (${T}_{{em}}$) is 1.3 years. The decarbonization contributions attributed to PV manufacturers, expressed as percentages, vary across different energy transition scenarios and different criteria. Our projections of the decarbonization contributions by China’s PV manufacturers from 2015 to 2050 are shown in Fig. 1, and proportions of total PV’s decarbonization contributions at four stages of the lifecycle under different assessment criteria and scenarios are summarized in Table 1. Under the STEPS scenario, manufacturers are estimated to contribute 66.5% to PV’s decarbonization for the energy consumption criterion, 35.4% for the economic costs criterion, and 74.9% for the carbon emissions criterion. Under the energy consumption and carbon emissions criteria, the PV manufacturing stage makes the most substantial contributions to decarbonization, indicating that manufacturers play a critical role in achieving China’s carbon reduction goals. However, under an economic costs criterion, the assigned decarbonization contributions decrease to 60.2% under the STEPS scenario and to 53.5% under APS scenario due to the considerably longer payback time for costs (${T}_{{eco}}$), bringing the shares of manufacturers in PV’s decarbonization contributions down to about one-third (35.4% for STEPS and 31.4% for APS, in Table 1), though manufacturers remain the most significant stage compared to others (Table 1). These results underscore the essential role of PV manufacturing in advancing China’s energy transition, particularly under the energy consumption and carbon emissions criteria. As a result, there is a need for supportive policies to reinforce PV manufacturers as a way of China’s low-carbon transition. Supporting data are available in Supplementary Notes 1–3.

**Table 1:** Proportions of total photovoltaic (PV)’s decarbonization contributions at four stages of the lifecycle under different assessment criteria and scenarios

China-STEPS							
Criteria	Manufacturing	Transportation and construction	Operation	Decommissioning	Unassigned	Sum	China’s PV manufacturers*
Energy	66.5%	9.9%	0.1%	8.9%	14.6%	100%	66.5%
Economy	35.4%	11.3%	7.2%	6.4%	39.8%	100%	35.4%
Emissions	74.9%	9.3%	0.1%	8.5%	7.2%	100%	74.9%

China-APS							
Criteria	Manufacturing	Transportation and construction	Operation	Decommissioning	Unassigned	Sum	China’s PV manufacturers*
Energy	64.4%	9.6%	0.1%	8.7%	17.2%	100%	64.4%
Economy	31.4%	10.0%	6.4%	5.7%	46.5%	100%	31.4%
Emissions	73.8%	9.2%	0.1%	8.4%	8.5%	100%	73.8%

Global-STEPS							
Criteria	Manufacturing	Transportation and construction	Operation	Decommissioning	Unassigned	Sum	China’s PV manufacturers*
Energy	65.6%	10.9%	0.1%	8.8%	14.6%	100%	45.4%
Economy	51.1%	16.3%	7.1%	6.4%	19.2%	100%	35.3%
Emissions	72.5%	10.3%	0.1%	8.3%	8.8%	100%	50.2%

Global-APS							
Criteria	Manufacturing	Transportation and construction	Operation	Decommissioning	Unassigned	Sum	China’s PV manufacturers*
Energy	62.7%	10.4%	0.1%	8.4%	18.4%	100%	43.4%
Economy	48.0%	15.3%	6.7%	6.0%	24.0%	100%	33.2%
Emissions	70.6%	10.0%	0.1%	8.0%	11.2%	100%	48.9%

Global-NZE							
Criteria	Manufacturing	Transportation and construction	Operation	Decommissioning	Unassigned	Sum	China’s PV manufacturers*
Energy	55.3%	9.2%	0.1%	7.4%	27.9%	100%	38.3%
Economy	40.4%	12.9%	5.6%	5.1%	36.1%	100%	27.9%
Emissions	65.6%	9.3%	0.1%	7.5%	17.5%	100%	45.4%

Global-Storage-NZE							
Criteria	Manufacturing	Transportation and construction	Operation	Decommissioning	Storage and Unassigned	Sum	China’s PV manufacturers*
Energy	53.4%	8.3%	0.1%	6.6%	10.3% + 21.2%	100%	37.0%
Economy	27.6%	8.8%	5.6%	5.1%	10.5% + 42.3%	100%	19.1%
Emissions	53.1%	8.1%	0.1%	6.5%	3.8% + 28.4%	100%	36.8%

*We multiply the predicted China’s PV production share by the share of the manufacturing stage. In China, the share value is set to be 1; at the global level, the share value is set to be 69.2% based on the prediction^51^. Results under three future electricity scenarios: the Stated Policies Scenario (STEPS), the Announced Pledges Scenario (APS), and the Net Zero Emissions by 2050 Scenario (NZE).

The predicted decarbonization contributions from PV manufacturers experience a rapid increase since 2015 and gradually reach a maximum under all three criteria (Fig. 1). This is because, in reality, the carbon intensity for grid electricity is dynamically changing. As the carbon intensity gap between PV and grid electricity narrows, the manufacturers’ decarbonization contributions would first increase, followed by a decline. Under the STEPS scenario, the strategic timing is predicted to occur around 2045 across all three criteria. When it comes to the APS scenario, the strategic timing is anticipated to occur around 2035, ten years earlier than the former. Despite variations in the absolute decarbonization contributions across the criteria, the associated strategic timing is consistent, providing a clear time window for implementing supportive policies to address potential challenges facing the PV manufacturers, such as trade barriers, difficulties in capacity expansion, and increased rare-earth elements costs^20,32,45–47^. All these policy measures would allow for maximizing PV’s decarbonization contributions in keeping with national decarbonization objectives. Once this strategic timing has passed and the decarbonization contributions of PV manufacturers begin to decline, more stringent regulations and policies would be required to prevent potential issues such as overproduction and resource waste within the PV industry^48,49^. Notably, the strategic timing in the APS scenario (2035) occurs five years later than China’s national target of peaking carbon emissions by 2030^25^. Since it is likely that more strict decarbonization policies would be applied to manufacturers after 2030 to ensure the net-zero goal in China before 2060, an additional five-year policy support for PV manufacturers may be essential to ensure the decarbonization contributions of PV manufacturers in achieving China’s ambitious long-term energy transition goals^50^.

### Strategic timing for photovoltaic manufacturers’ decarbonization contributions globally

**Fig. 2:**
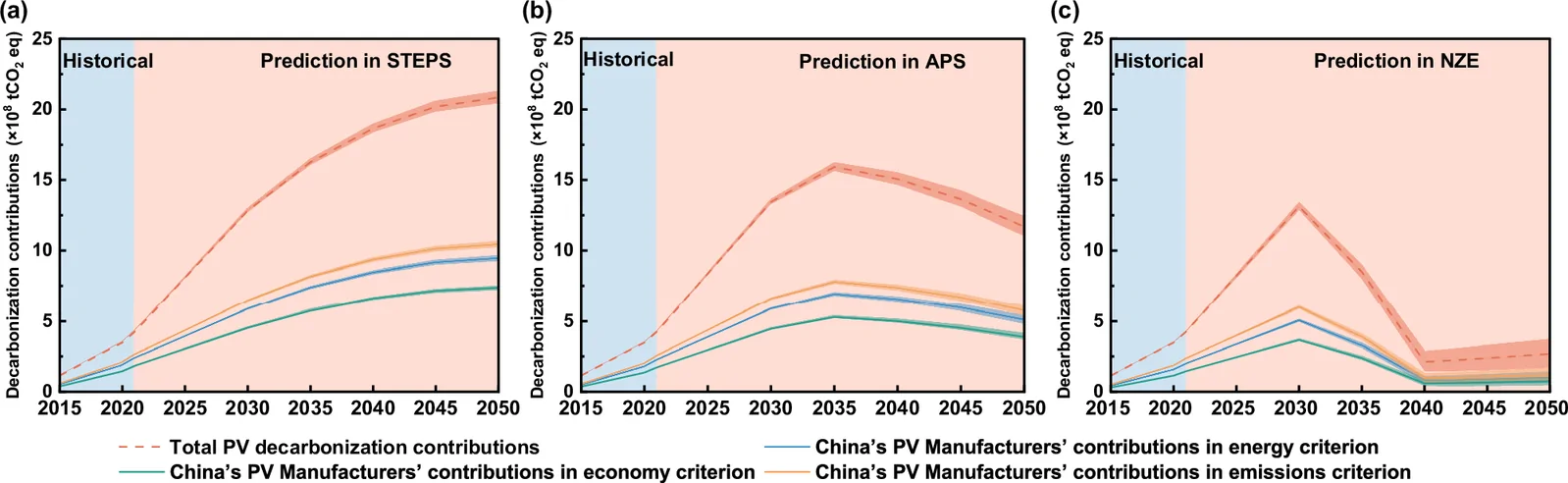
The decarbonization contributions and associated strategic timing of China’s photovoltaic (PV) manufacturers at the global level. **a** The decarbonization contributions under the Stated Policies Scenario (STEPS). **b** The decarbonization contributions under the Announced Pledges Scenario (APS). **c** The decarbonization contributions under the Net Zero Emissions by 2050 Scenario (NZE).

China’s PV manufacturing industry plays a crucial role in global decarbonization. Currently, it accounts for 84% of global mass PV production, with its share projected to decrease slightly to 69.2% by 2027^19,51^. To evaluate the potential global decarbonization contributions of China’s PV manufacturers, we compare three scenarios: STEPS, APS, and NZE, as illustrated in Fig. 2. At the global level, the energy consumption payback time (${T}_{{en}}$ = 2.6 years) and the carbon emissions payback time (${T}_{{em}}$ = 1.6 years) remain similar to that of China at the national level (${T}_{{en}}$ = 2.7 years, ${T}_{{em}}$ = 1.3 years). However, the economic costs payback time at the global level (${T}_{{eco}}=\,3.5{{{\rm{years}}}}$) is significantly shorter than that on a national scale in China (${T}_{{eco}}=\,8.2{{{\rm{years}}}}$). This is primarily due to the lower average electricity price as compared to the global average, see Supplementary Note 4 for the detailed setup for the electricity price. Consequently, the revenue generated by PV-produced electricity is much lower in China, making it much longer to offset the lifecycle economic costs for PV systems. This results in larger assigned decarbonization contributions from the economic costs criterion at the global level compared to the national level in China, as shown in Table 1. Assuming that China’s share of global PV production remains at 69.2% from 2027 onward, the decarbonization contributions of China’s PV manufacturing within the PV industry at the global level vary across the three criteria—energy consumption, economic costs, and carbon emissions—depending on the scenario: 45.4%, 35.3% and 50.2% under STEPS; 43.4%, 33.2% and 48.9% under APS; and 38.3%, 27.9% and 45.4% under NZE. Further details, including sensitivity analyses on electricity prices and discount rates, can be found in Supplementary Note 4.

At the global level, the choice of decarbonization pathways would result in different levels of PV manufacturers’ decarbonization contributions and time windows to implement policies. Under the STEPS scenario, China’s PV manufacturers show a consistent increase in decarbonization contributions across all three criteria from 2015 to 2050, without a strategic timing observed. In the APS scenario, the strategic timing is identified as 2035 at the global level, aligning with the predicted strategic timing at the national level in support of China’s PV manufacturers. In contrast, the strategic timing under the NZE scenario, which represents a more ambitious decarbonization goal, is projected to be 2030. This coincides with China’s national goal of peaking carbon emissions by 2030. Nevertheless, the anticipated decline in global decarbonization contributions from China’s PV manufacturers after 2030 suggests a need to shift policy focus towards China’s domestic market beyond that time point.

### Strategic timing when considering industrial symbiosis’ decarbonization contributions

**Fig. 3:**
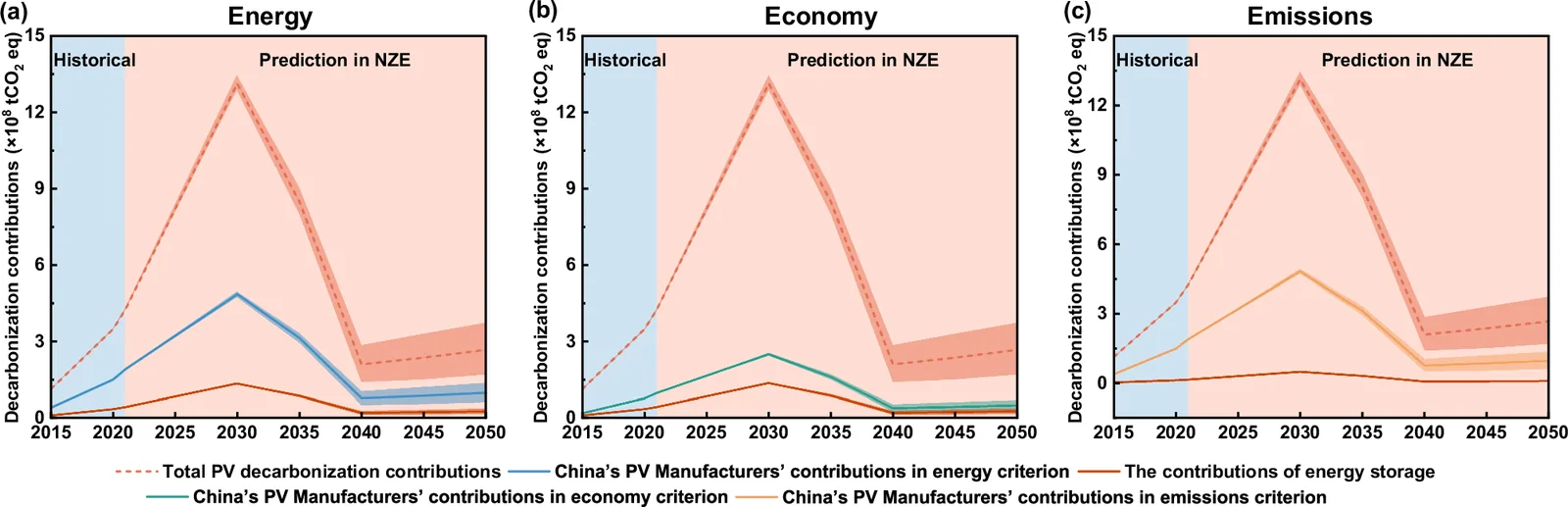
The decarbonization contributions and associated strategic timing of China’s photovoltaic (PV) manufacturing and battery energy storage system (BESS) at the global level under the Net Zero Emissions by 2050 Scenario (NZE). **a** The decarbonization contributions under the energy consumption criterion. **b** The decarbonization contributions under the economic costs criterion. **c** The decarbonization contributions under the carbon emissions criterion.

Large-scale energy storage is essential for ensuring a stable and sustainable power supply, thereby improving the overall efficiency and resilience of PV systems^52^. In this study, a lithium-ion battery energy storage system (BESS) with a storage capacity of up to 20% of the installed PV capacity is considered^53,54^, with additional details available in Supplementary Note 5. By this setup, BESS serves as producers with contributions to decarbonization, with their decarbonization efforts assessed alongside PV systems. The combination of PV and BESS, particularly through coordinated industrial processes, leads to enhanced decarbonization contributions for the entire manufacturing industry. However, with the inclusion of BESS, the decarbonization contributions attributed to PV manufacturers are reduced. Figure 3 shows decarbonization contributions and strategic timing of China’s PV manufacturing and BESS under the NZE scenario. The proportions of assigned decarbonization contributions for PV and BESS within the energy storage integrated PV industry under the three criteria range from 57.7–78.8% (Table 1). This allocation shifts the proportions of the PV manufacturing industry in total PV’s decarbonization contributions from a range of 40.4–65.6% to 27.6–53.4%, though PV manufacturing remains the primary contributor.

The strategic timing for China’s PV manufacturing decarbonization contributions, even with the inclusion of BESS, remains consistent at 2030 across all three criteria under the NZE scenario. For this reason, in the contemporary energy supply landscape, manufacturers involved in renewable energy industries—including BESS—should all be recognized as integral contributors to the decarbonization efforts with supportive policies implemented prior to the strategic timing^50^. The inclusion of BESS does not undermine the primary role of PV manufacturers, and both PV and BESS manufacturers share the same strategic timing.

## Discussion

Our research demonstrates the substantial decarbonization contributions of the PV manufacturing industry in China. In that sense, carbon emissions from the PV manufacturing stage are integral to the overall decarbonization efforts, as they enable the subsequent climate benefits at the PV operation stage. By fairly allocating PV’s decarbonization contributions to the manufacturing stage based on energy consumption, economic costs, and carbon emissions, we provide a more integrated view of the lifecycle contributions of PV systems; that is, China’s PV manufacturers could contribute 31.4–73.8% of total PV decarbonization towards achieving China’s decarbonization goals in energy transition under the APS scenario at the strategic timing of 2035. At the global level, under the NZE scenario to achieve net-zero emissions for the power sector by 2050, China’s PV manufacturers are anticipated to contribute 27.9–45.4%, and this figure slightly decreases to 19.1–37.0% with consideration of energy storage solutions at the strategic timing of 2030. Table 1 compares the proportions of total PV’s decarbonization contributions at four stages of the lifecycle under different assessment criteria and scenarios.

Chinese policymakers are contemplating the imposition of more stringent regulations for those manufacturers that generate enormous carbon emissions. This plausible regulatory tightening could pose obstacles to the PV capacity growth, thus undermining the low-carbon transition of grid systems^55^. By recognizing and determining the decarbonization contributions by the PV manufacturers in China, we show that the strategic timing is expected to take place by 2030 at the global level under the NZE scenario.

Prior to this timing, we anticipate a rising trajectory in the decarbonization contributions of the PV manufacturing industry, primarily driven by the expanding deployment of PV systems, as the carbon intensity of PV-generated electricity is substantially lower than that of the grid electricity at present. During this period, supportive regulations and policies are essential to ensure the rapid development of the PV manufacturing industry for a successful energy transition. In allocating carbon quotas for the emissions trading scheme, for instance, not only emissions but also decarbonization contributions should be considered simultaneously^56^, thus avoiding the risk of quota restrictions on PV manufacturers^57^. Nevertheless, it is necessary to move away from one-size-fits-all decarbonization strategies by distinguishing renewable industries based on their decarbonization contributions, and prioritizing support for those manufacturers that are key to decarbonization. Note that policy support for PV manufacturing should not be simply understood as the removal of constraints on upstream emissions to increase production. Rather, it should be more detailed and targeted by going beyond output expansion merely to include support for innovation in PV module manufacturing technologies^35,58,59^, the reshaping of the spatial configuration^60^, and the removal of bottlenecks in upstream material supply^19^. Such measures would ensure that policy support for PV manufacturing is aligned with reducing the risks of lock-in emissions from incumbent high-carbon production and avoiding innovation crowding-out by refocusing incentives towards low-carbon technologies^61–63^. More importantly, technological innovation facilitates shortening the payback time by reducing energy consumption, economic costs, and carbon emissions at the production stage, thereby increasing the magnitude and duration of the net decarbonization contributions in manufacturing. In this sense, support for PV manufacturing is not aimed at sustaining high-emission production, but at enabling earlier and greater net decarbonization contributions through accelerated technological improvement.

The strategic timing under the NZE scenario is projected to occur in 2030 at the global level, five years earlier than 2035—the strategic timing under the APS scenario for China. Given the leading role in the world’s PV production, China’s potential overproduction is of particular concern^48,49,64^. While our analysis demonstrates the substantial decarbonization contributions by China’s PV manufacturers at both national and global levels, we emphasize that when the world has reached its strategic timing, while China has not yet, a shift in policy focus from the global market to China’s domestic market is needed to avoid potential PV overproduction at the global level^65^. This strategy agrees quite well with China’s latest nationally determined contributions to expand installed capacity of solar and wind to over six times the 2020 level by 2035^66^.

After the strategic timing, we observe a decline in these contributions as the carbon intensity of grid electricity reaches a significantly low level that is very close to the PV-generated electricity. This suggests that the mitigation advantage of further PV deployment diminishes once the system reaches a low-carbon electricity mix. Accordingly, more stringent regulations and policies can be applied to the PV manufacturing industry after the strategic timing to prevent potential overproduction and resource waste^48,49^, such as phasing out inefficient capacity and enforcing stricter standards on carbon emissions^67,68^. Such measures could help reduce the lifecycle carbon intensity of PV-generated electricity, thereby maintaining or even expanding the mitigation advantage over grid electricity. By doing so, the PV manufacturing can continue to support energy transition, not merely through capacity expansion, but through low-carbon production. After 2035—the strategic timing in China under APS, the decarbonization contributions from China’s PV manufacturers would begin to decline; therefore, more stringent regulations should be implemented for China’s PV manufacturers to avoid potential issues such as excess production capacity.

Our scenario analysis indicates that the more rigorous the decarbonization goal is, the shorter the strategic timing to take action on developing the PV industry. This is particularly true given the fundamental role of the installed capacity supplied by PV manufacturers towards net-zero emissions^23^. Previous government subsidies, such as the feed-in tariff policy, have been primarily intended for PV power generation enterprises^69^, leaving out the decarbonization contributions of the PV manufacturers. Therefore, our study in this paper calls for more reasonable subsidy policies spanning the lifecycle of PV and other renewable energy industries, with an emphasis on enhanced incentives and investments at the manufacturing stage. This could benefit the supply chain that spans numerous manufacturing industries, from mineral extraction and processing, mid-product processing, auxiliary equipment manufacturing, to system integration manufacturers. Furthermore, our PDC framework can be readily extended to evaluate the decarbonization contributions of industrial symbiosis beyond PV by recognizing their roles as producers^70^. This includes various energy supply and storage technologies in building a flexible, efficient and safe energy system. It provides a reasonable assessment of the shared decarbonization contributions within the renewable energy system, supporting the sustainable development of renewable energy. Nevertheless, precautionary policies would be required to minimize potential negative outcomes of renewable energy deployment, such as increased land appropriation, biodiversity loss, and mining of rare earth metals^71,72^.

In addition to the substantial decarbonization contributions throughout the world in the past and the future, PV manufacturing aligns with the endorsement of the SDGs. About 85% of the SDG targets call for actions within the energy system, including PV in pursuit of SDG 7^73^. SDG 12 emphasizes the reduction of resource waste and environmental impacts by PV manufacturers in support of responsible consumption and production. SDG 13 calls for the integration of climate action, such as the replacement of fossil fuels with renewables, into national policies, strategies and planning^74^. Unfortunately, the contributions of manufacturers, particularly those supporting energy transition, often go unnoticed within the SDGs. This requires more attention to renewable energy producers as a basis for low-carbon transitions in the whole supply chain. The recognition of decarbonization contributions from producers is therefore meant to complement the 2030 Agenda for Sustainable Development. Given that 2030 marks a milestone for both the Paris Agreement and the SDGs, the PDC framework can further serve as a valuable tool to assist policymakers in effectively monitoring global progress in energy transition and creating policy synergies.

## Methods

### Lifecycle carbon intensity per unit electricity production for a photovoltaic system

When calculating ${{CI}}_{{PV}}$, PV system is divided into four stages in this study, including the manufacturing stage, the transportation and construction stage, the operation stage, and the decommissioning stage. The use of carbon equivalents aims to standardize the emissions of main greenhouse gases, including CO_2_, CH_4_, N_2_O, and SF_6_^43^. The total carbon emissions of PV systems are calculated as the sum of four stages, as shown in Eqs. (1, 2).

1 $${E}_{{total}}={E}_{{manu}}+{E}_{{trans}}+{E}_{{op}}+{E}_{{de}}$$

2 $${E}_{i}={\sum }_{i=1}^{j}({{CI}}_{i}{M}_{i})+{{CI}}_{{grid}}{Q}_{e}$$

where ${E}_{{total}}$ represents the total carbon emissions from the PV system (tCO_2_ eq); ${E}_{{manu}}$, ${E}_{{trans}}$, ${E}_{{op}}$ and ${E}_{{de}}$ represent the carbon emissions of the manufacturing stage, the transportation and construction stage, the operation stage and the decommissioning stage, respectively (tCO_2_ eq); ${E}_{i}$ represents the carbon emissions for individual stage (tCO_2_ eq); ${{CI}}_{i}$ represents carbon emission coefficient of the *i*^th^ materials (tCO_2_ eq t^−1^) (see Supplementary Note 2 for the coefficient of each material); ${M}_{i}$ represents the consumption of the *i*^th^ materials (t); j represents the number of types of consumption materials or energy; ${{CI}}_{{grid}}$ represents the carbon intensity of per unit electricity from grid in China using manufacturing data in 2022 (tCO_2_ eq MWh^−1^); ${Q}_{e}$ represents the electricity consumption during the stage over the lifetime of the system (MWh), and this value accounts for 0.1% of the total carbon emissions.

The carbon intensity of per unit electricity production from PV is calculated by dividing total electric generation over 30 years, as shown in Eqs. (3, 4).

3 $${q}_{t}=\left\{\begin{array}{c}{WH}{\eta }_{{PR}}{\eta }_{{tran}}({1-\varepsilon }_{0}),t=1 \hfill \\ {WH}{\eta }_{{PR}}{\eta }_{{tran}}({1-\varepsilon }_{0}){(1-{\varepsilon }_{y})}^{t},2\le t\le 30\end{array}\right.$$

4 $${{CI}}_{{PV}}=\frac{{E}_{{total}}}{{\sum }_{t=1}^{30}{q}_{t}}$$

where ${q}_{t}$ represents the *t*^th^ year generating electricity by PV system (MWh); W represents the installed capacity of PV system (MWp); H represents the average peak sunshine hours (h); ${\eta }_{{PR}}$ represents the performance ratio of the PV power plant, which is assumed to be 79%^13,75^; ${\eta }_{{tran}}$ represents the average loss coefficients of electricity transmission in China, and those of 2018–2022 are shown in Supplementary Note 2; ${\varepsilon }_{0}$ represents the attenuation coefficient of PV system at the first year, which is assumed to be 2%; ${\varepsilon }_{y}$ represents the annual attenuation coefficient of PV system at every year after the first year, which is assumed to be 0.6%; ${{CI}}_{{PV}}$ represents the carbon intensity of electricity generation by PV (tCO_2_ eq MWh^−1^) considering both emissions and recycling.

As an example, we measure the impact of a 1 MWp PV system on carbon emissions and decarbonization contributions over a 30-year period using China’s manufacturing performance data in 2022 (see Supplementary Note 2 for more details). We have three layers in our framework (see Supplementary Fig. 2). The first, referred to as Layer I, examines the detailed production steps for PV panels. Layer II assessment covers various stages, including manufacturing (like PV panels and inverters), transportation, construction, operation, and decommissioning processes. The third, known as Layer III, further incorporates decarbonization contributions. Specifically, the Layer II encompasses Scopes 1, 2, and 3 of the GHG Protocol. Additional information about manufacturing data and carbon footprint in 2018–2022 is available in Supplementary Note 2. Of all the stages, the manufacturing stage that amounts to 1013 tCO_2_ eq is responsible for 80.7% of the total carbon emissions. The primary contributors to these carbon emissions are electricity and metal usage, making up 37.8% and 37.0%, respectively. Most of these carbon emissions come from the manufacturing stage. The remaining carbon emissions are relatively small in comparison. They account for 10.0%, 0.1%, and 9.2% at the transportation and construction, operation, and decommissioning stages, respectively. The decarbonization contributions occur primarily during the operation and decommissioning stages. During decommissioning, in addition to PV modules, a significant amount of metal resources, especially from components including aluminum frames, inverters, PV holders, and cables, can be recycled^76^. The associated carbon emissions due to non-recyclable materials, such as concrete, are considered as well. The carbon intensity of electricity generated from PV panels in China, considering both carbon emissions and resource recovery, is 16.94 gCO_2_ eq kWh^−1^ in 2022. At the global level, the carbon intensity of electricity generated from PV is calculated to be 16.87 gCO_2_ eq kWh^−1^ in 2022, slightly lower than the value in China, which is the average intensity values of 18 countries (including top ten countries in terms of newly installed PV capacity in 2021) weighted by their administrative areas, see Supplementary Note 4. For detailed findings and sensitivity analyses, please see Supplementary Notes 2-4.

### Total decarbonization contributions of photovoltaic systems

The carbon emissions associated with PV manufacturing are determined by the electricity intensity of the region where production occurs, whereas the decarbonization contributions of PV systems are determined by the gap between the carbon intensity of PV-generated electricity and the average grid intensity at the deployment location. The regional grid intensity is calculated based on the mix of different electricity generation technologies, including coal, natural gas, wind, solar PV, oil, fossil fuels with CCUS, and others^43^. Rather than comparing PV with coal-fired power plants, we define the decarbonization contributions of PV systems as the sum of reduced carbon emissions by PV-generated electricity replacing grid electricity^77^. To determine the annual decarbonization contributions, we utilize a dynamically changing average carbon intensity for the grid, as projected by the IEA under different scenarios, including STEPS, APS and NZE. This dynamic approach accounts for predicted electricity structure from various technologies and the total amount of generated electricity until 2050. The annual decarbonization contributions of the PV industry are derived from the electricity generation by PV (${Q}_{t}$), the carbon intensity of the grid at each year (${{CI}}_{{grid},t}$) and the carbon intensity of electricity from PV (${{CI}}_{{PV}}$), as shown in Eq. (5). Note that the scope of this study does not include the end-use applications of PV-generated electricity (e.g., power-to-X). While such uses may have a limited impact on the total decarbonization contributions of PV, they do not affect the proportional contributions from PV manufacturers or the predicted strategic timing, and therefore would not compromise the policy recommendations of our study.

5 $${D}_{t}={Q}_{t}({{CI}}_{{grid},t}-{{CI}}_{{PV}}){\gamma }_{t}$$

where ${D}_{t}$ represents the decarbonization contributions of the PV industry at the t^th^ year (tCO_2_ eq); ${Q}_{t}$ represents the electricity generated by PV at the t^th^ year (MWh); ${{CI}}_{{grid},t}$ represents the carbon intensity of grid at the t^th^ year (tCO_2_ eq MWh^−1^); ${{CI}}_{{PV}}$ represents the carbon intensity of electricity generation by PV (tCO_2_ eq MWh^−1^) considering both carbon emissions and recycling; ${\gamma }_{t}$ represents the China production proportion coefficient at the t^th^ year, indicating the percentage of PV modules produced in China compared to the global PV module production. The China production proportion coefficient (${\gamma }_{t}$), representing share of China’s PV production at the global level, was set to be 1 when assessing PV manufacturers’ decarbonization contributions in China. When evaluating at the global level, ${\gamma }_{t}$ undergoes a linear transformation from 84% in 2021^19^ to 69.2% in 2027^51^, afterwards it is assumed to remain constant.

The annual carbon intensity of the grid (${{CI}}_{{grid},t}$) is calculated based on the energy supply structure and carbon intensities for different means using Eq. (6). In this study, we consider ten different electricity generation methods, including coal, natural gas, wind, solar PV, modern bioenergy, hydro, nuclear, oil, fossil fuels with CCUS, and others (see Supplementary Notes 3-4 for more details).

6 $${{CI}}_{{grid},t}=\sum ({{CI}}_{z}{{\rm P}}_{z,t})$$

where ${{\rm P}}_{z,t}$ represents the proportion of electricity generation at the ${t}^{{th}}$ year by the ${z}^{{th}}$ method; ${{CI}}_{z}$ represents the carbon intensity of electricity from the ${z}^{{th}}$ method (tCO_2_ eq MWh^−1^).

Note that in the energy transition context, the increase in the proportion of electricity generated from the PV system, as well as from other renewables will speed up decarbonization. However, the decarbonization contributions of PV will decrease as the average carbon intensity becomes lower when the energy is dominated by renewables. This suggests a strategic timing for the largest decarbonization contributions by PV manufacturers from different criteria.

### Payback times under the three criteria

We introduce three criteria: energy consumption, economic costs, and carbon emissions (see Supplementary Note 1 for more details). We employ the energy consumption, the economic costs and the carbon emissions as the proxy for each criterion, respectively. The decarbonization contributions can be categorized into two components, unassigned and assigned contributions. The former category spans from the initiation of PV system operation until the payback time, while the latter spans from the payback time to the decommissioning of the PV system. Under the three criteria, we calculate the cumulative values of energy consumption (${{EN}}_{c}$), economic costs (${{ECO}}_{c}$) and carbon emissions (${{EM}}_{c}$) throughout the lifetime of 30 years and determine the associated payback time (${T}_{{en}}$, ${T}_{{eco}}$ and ${T}_{{em}}$) when the cumulative values reach zero, as shown in Eqs. (7–9).

7 $${{EN}}_{c}={{EN}}_{{initial}}-{\sum }_{i=1}^{t}{Q}_{i}$$

where ${{EN}}_{c}$ represents the cumulative energy consumption (kWh); ${{EN}}_{{initial}}$ represents the initial amount of energy consumption at the manufacturing stage, the transportation and construction stage, and the operation stage (kWh); ${Q}_{i}$ represents the i year generating electricity by PV system (kWh); t represents the t^th^ year.

8 $${ECO}={{ECO}}_{{initial}}-{\sum }_{i=1}^{t}\frac{{{Q}_{i}{p}_{{grid}}-{ECO}}_{{op}}}{{\left(1+d\right)}^{i}}$$

where ${{ECO}}_{c}$ represents the cumulative economic revenue (CNY); ${{ECO}}_{{initial}}$ represents the initial amount of economic costs at the manufacturing stage, the transportation and construction stage, and the operation stage (CNY); ${p}_{{grid}}$ represents the price of electricity from PV connected to the grid (CNY kWh^−1^); ${{ECO}}_{{op}}$ represents the economic costs of annual operation (CNY year^−1^); d represents the discount rate.

9 $${EM}={{EM}}_{{initial}}-{\sum }_{i=1}^{t}({Q}_{i}{{CI}}_{{grid},t})$$

where ${{EM}}_{c}$ represents the cumulative carbon emissions (tCO_2_ eq); ${{EM}}_{{initial}}$ represents the initial amount of carbon emissions at the manufacturing stage, the transportation and construction stage, and the operation stage (tCO_2_ eq); ${{CI}}_{{grid}}$ represents carbon intensity of per unit electricity from grid in China (tCO_2_ eq MWh^−1^), ${Q}_{i}{{CI}}_{{grid},t}$ represents the avoided carbon emissions using grid electricity.

The assigned decarbonization contributions of the PV industry under each criterion, ${D}_{t,{assigned}}$, can be calculated by adding up the decarbonization contributions between each payback time and the end of the lifecycle, as shown in Eq. (10).

10 $${D}_{t,{assigned}}={\sum }_{t={T}_{{en}},{T}_{{eco}},\,{T}_{{em}}}^{t=30}{D}_{t}$$

where ${D}_{t}$ is the annual decarbonization contributions of the PV industry, shown in Eq. (5).

### Decarbonization contributions for manufacturers within the photovoltaic industry

**Fig. 4:**
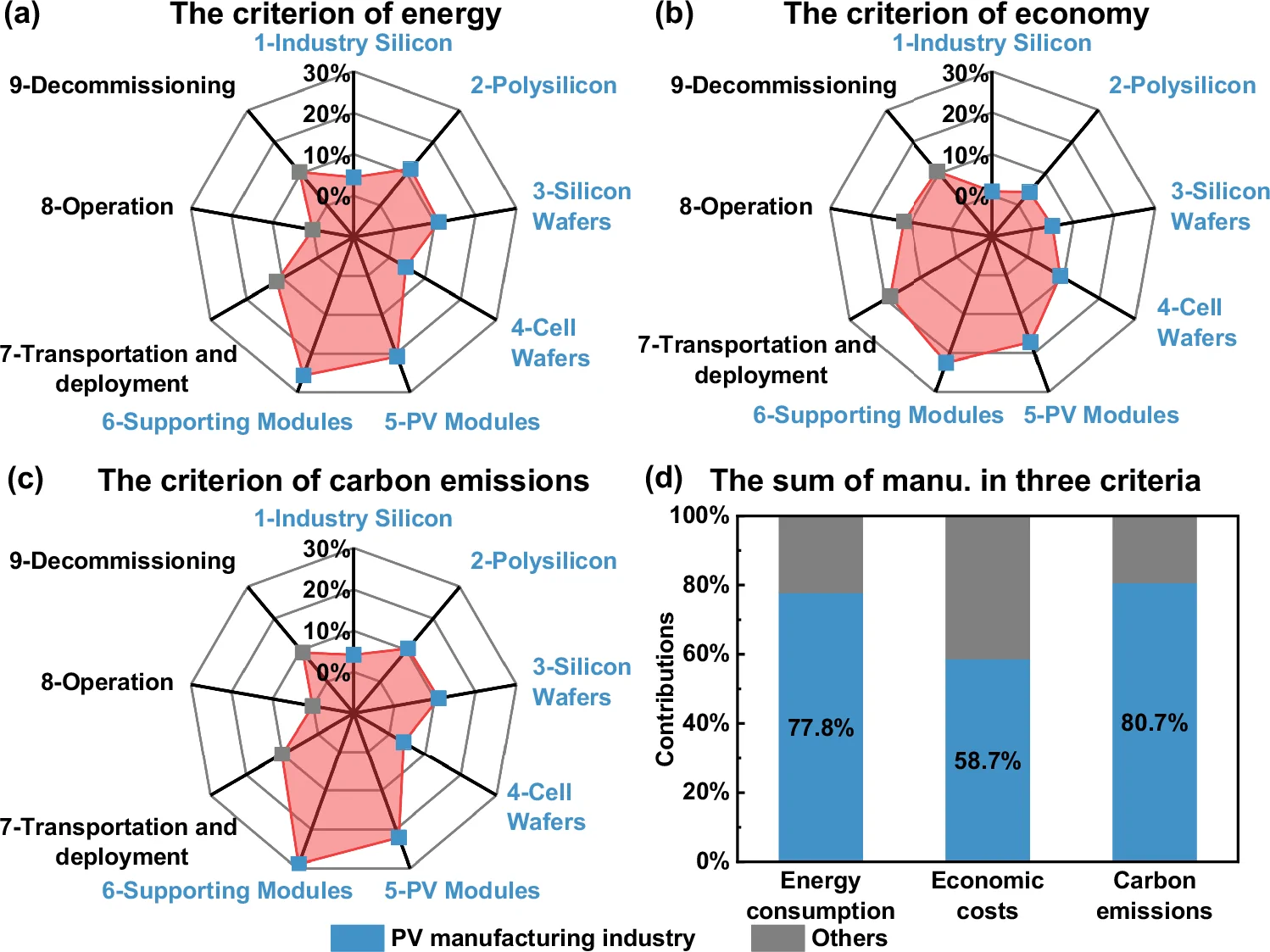
The cost segregation from the three criteria for a photovoltaic (PV) system. **a**–**c** The relative contributions of the nine life-cycle processes of the PV system allocated under energy consumption, economic costs, and carbon emissions criteria, respectively. **d** The cumulative contributions of the PV manufacturing industry (processes 1–6 in blue).

The assigned decarbonization contributions of the PV industry under each criterion are distributed into four stages, namely manufacturing, transportation and construction, operation, and decommissioning. These four stages involve nine processes: 1 - industrial silicon, 2 - solar-grade polysilicon, 3 - silicon wafers, 4 - cells, 5 - PV modules, 6 - supporting modules, 7 - transportation and construction, 8 - operation, and 9 - decommissioning. The numbering of these stages corresponds to that shown in Fig. 4. Notably, the first six stages are subject to PV manufacturing. We assess the impact on the decarbonization of these six PV manufacturing stages across the three criteria. We distribute the assigned decarbonization among the nine processes once the associated payback time is met. Additional details and the sources for the data can be found in Supplementary Notes 1-2.

The assigned coefficient of decarbonization contributions for manufacturers under each criterion is shown in Eqs. (11, 12).

11 $${\omega }_{x}=\frac{{\delta }_{x}}{{\sum }_{x=1}^{9}{\delta }_{x}}$$

12 $${\omega }_{x,{manu}}={\sum }_{x=1}^{6}{\omega }_{x}$$

where ${\omega }_{x}$ represents the assigned coefficient of decarbonization contributions of the ${x}^{{th}}$ unit process; ${\omega }_{x,{manu}}$ represents the assigned coefficient of decarbonization contributions for manufacturers; ${\delta }_{x}$ represents the representative index of the ${x}^{{th}}$ unit process, determined by energy consumption, economic costs and carbon emissions following the criteria of energy, economy and carbon emission, respectively.

The decarbonization contributions for manufacturing stage in *t*^th^ year involving process 1-6 under each criterion can be calculated as shown in Eq. (13).

13 $${D}_{t,{manu}}={\sum }_{x=1}^{6}{D}_{t,{assigned}}{\omega }_{x}$$

where ${D}_{t,{manu}}$ represents the assigned decarbonization for manufacturers involving the ${1-6}^{{th}}$ process (tCO_2_ eq). Figure 3 indicates that, using the relevant data in 2022, the PV manufacturing stage accounts for 77.8% of energy consumption, 58.7% of economic costs, and 80.7% of carbon emissions.

### Scenarios for the electricity structure in China and at the global level

**Fig. 5:**
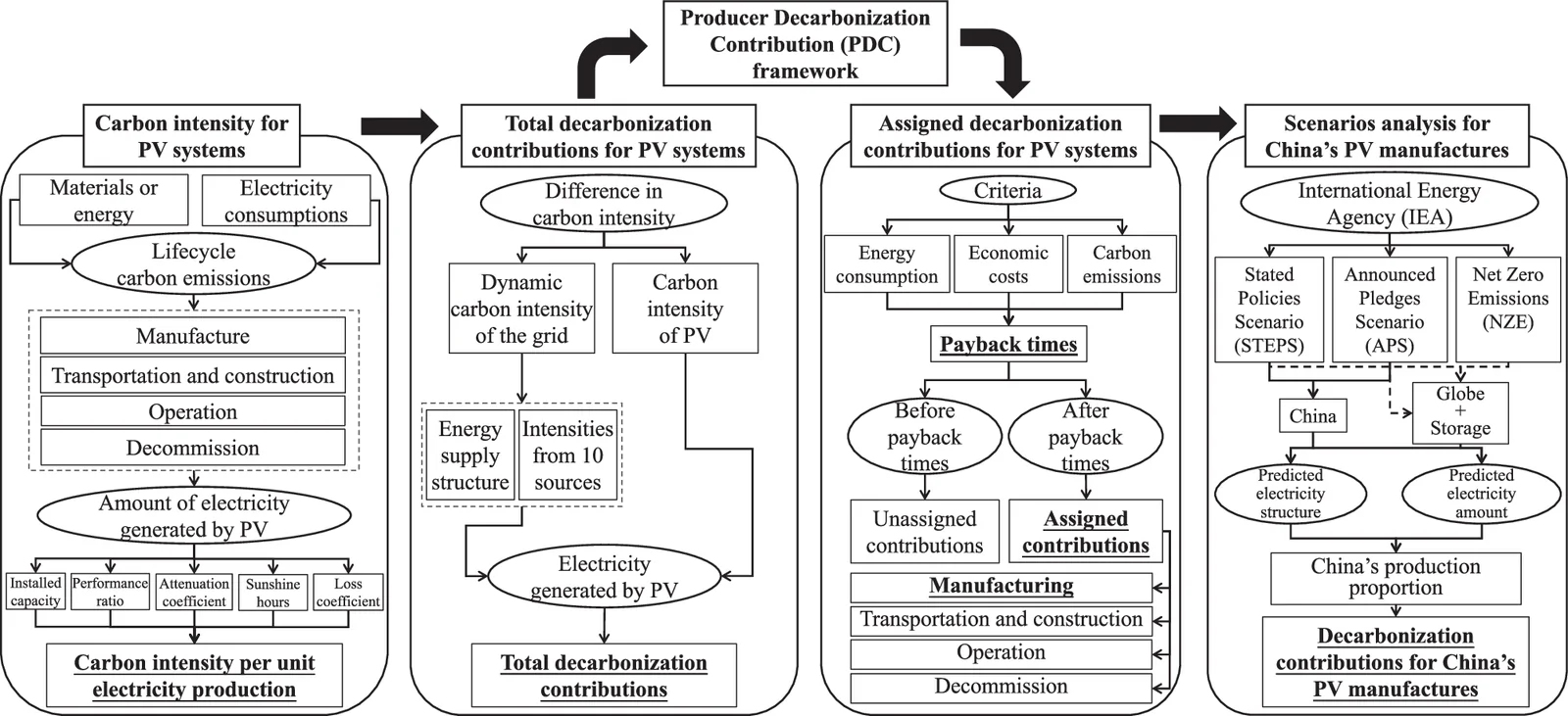
The PDC framework determining the decarbonization contributions for China’s photovoltaic (PV) manufacturers under different scenarios and criteria. The framework consists of four steps, including the calculation of carbon intensity for PV systems, quantification of total decarbonization contributions for PV systems, allocation of assigned decarbonization contributions for PV manufacturers, and scenario analysis to evaluate the contribution of PV manufacturers in China to global decarbonization. First, the carbon intensity for PV systems is determined by both lifecycle carbon emissions and electricity generation over the lifetime of PV systems. We consider emissions and recovery benefits from manufacturing, transportation, construction, operation, and decommissioning stages. The electricity generation of PV systems is estimated by key parameters: installed capacity, performance ratio, attenuation coefficient, sunshine hours, and transmission loss coefficient. Second, the total decarbonization contributions for PV systems are quantified by integrating PV electricity generation, the carbon intensity of PV electricity, and dynamic grid carbon intensity under different energy transition scenarios. The grid carbon intensity is calculated based on the projected energy supply structure and the corresponding carbon intensities of different electricity generation technologies. Third, the assigned decarbonization contributions for PV systems are allocated according to the Producer Decarbonization Contribution (PDC) framework using three criteria, including energy consumption, economic costs, and carbon emissions. The corresponding payback times are determined under each criterion and then used as thresholds for dividing the total decarbonization contributions into unassigned and assigned contributions. After the payback period, the assigned decarbonization contributions are further distributed among different lifecycle stages and processes, with the manufacturing-related processes representing the decarbonization contributions for PV manufacturers. Finally, scenario analysis of the decarbonization contributions for China’s PV manufacturers is conducted by combining the allocated contributions with projected electricity structures and PV deployment under different scenarios, including the Stated Policies Scenario (STEPS), Announced Pledges Scenario (APS), and Net Zero Emissions by 2050 Scenario (NZE). We perform the analysis at both national and global levels.

We use the predicted electricity structure and amount from IEA World Energy Outlook 2022 as inputs to account for the decarbonization contributions in China and at the global level under the Stated Policies Scenario (STEPS) and Announced Pledges Scenario (APS) to accommodate net-zero pledges. We further explore the IEA’s Net Zero Emissions by 2050 Scenario (NZE) that allows the global power sector to achieve net-zero emissions by 2050. Figure 5 visualizes the PDC framework for measuring the decarbonization contributions by China’s PV manufacturers under different scenarios and criteria.

### Data sources

In this study, we collect information on the performance of PV modules, the electricity used during production, and the materials used in the manufacturing process. We obtain the data from various sources, including published literature, industrial reports, government census data, and established industrial standards. To calculate the carbon emissions produced by the materials used in each step of the process, we get access to the GaBi databases (GaBi: https://gabi.sphera.com). These databases provide sufficient information on the resources and energy consumed at each stage of production, such as electricity, aluminum, and steel. We prioritize data from China, but for materials not covered in the database, we refer to the literature. We also replace some unavailable data, such as the carbon emission coefficients of EVA (Ethylene-vinyl acetate copolymer), HNO_3_ (Nitric acid), and TPT (Tedlar-PET-Tedlar) for China, with those that are available for other countries from the GaBi databases or the literature. In addition, some data related to manufacturing and recycling processes might be outdated, and these details are documented and discussed in Supplementary Notes 1, 2. More details about the sources of our data can be found in Supplementary Notes 1, 2. The electricity structure data for the three scenarios (STEPS, APS, and NZE) are obtained from the World Energy Outlook 2022. While differences across the data sources and publication years may affect the absolute values of PV-generated electricity, our analysis focuses on the relative share of decarbonization contributions from PV manufacturers and the associated strategic timing, both of which remain robust across datasets. For estimating the absolute decarbonization contributions, however, the most up-to-date data are preferred.

### Limitations

As an exploration of the decarbonization contributions by China’s PV in global energy transition, our study remains some limitations that should be addressed in future work. For simplicity, we assume that all electricity generated by PV systems can be entirely integrated into the national grid. Moreover, our study focuses exclusively on PV and BESS, although it can be easily extended to other renewables, such as wind power, with minor changes to the current PDC framework. In that sense, decarbonization contributions should consider not only PV manufacturers but also all other manufacturers across the entire power supply chain, with contributions assigned accordingly. This broader perspective is nevertheless beyond the scope of this study and warrants a follow-up analysis using the PDC framework developed in this work.

### Reporting summary

Further information on research design is available in the Nature Portfolio Reporting Summary linked to this article.

## Supplementary information

Supplementary Information

Reporting Summary

Transparent Peer Review file

## Source data

Source Data

## Data Availability

The authors declare that the data supporting the findings of this study are available within the paper and the Supplementary Information files. Additional questions about the data supporting the findings of this study can be directed to the corresponding authors. Source data are provided with this paper.

## References

[1] Goldthau, A. & Tagliapietra, S. Energy crisis: five questions that must be answered in 2023. *Nature* **612**, 627–630 (2022). 36526908 10.1038/d41586-022-04467-w

[2] Buonocore, J. J. et al. Health and climate benefits of different energy-efficiency and renewable energy choices. *Nat. Clim. Change* **6**, 100–105 (2016).

[3] Kammen, D. M. & Sunter, D. A. City-integrated renewable energy for urban sustainability. *Science* **352**, 922–928 (2016). 27199413 10.1126/science.aad9302

[4] Peng, K. et al. The global power sector’s low-carbon transition may enhance sustainable development goal achievement. *Nat. Commun.* **14**, 3144 (2023). 37253805 10.1038/s41467-023-38987-4 PMC10229651

[5] Renewable capacity statistics 2023. *International Renewable Energy Agency*, (Abu Dhabi, 2023).

[6] IEA. World Energy Outlook 2022. *International Energy Agency (IEA)*. (Paris, 2022).

[7] Creutzig, F. et al. The underestimated potential of solar energy to mitigate climate change. *Nat. Energy* **2**, 17140 (2017).

[8] Gulagi, A. et al. The role of renewables for the rapid transitioning of the power sector across states in India. *Nat. Commun.* **13**, 5499 (2022). 36130937 10.1038/s41467-022-33048-8 PMC9492668

[9] Cherp, A., Vinichenko, V., Tosun, J., Gordon, J. A. & Jewell, J. National growth dynamics of wind and solar power compared to the growth required for global climate targets. *Nat. Energy* **6**, 742–754 (2021).

[10] Liu, W. et al. Flexible solar cells based on foldable silicon wafers with blunted edges. *Nature* **617**, 717–723 (2023). 37225883 10.1038/s41586-023-05921-z PMC10208971

[11] Müller, A. et al. A comparative life cycle assessment of silicon PV modules: Impact of module design, manufacturing location and inventory. *Sol. Energy Mater. Sol. Cells* **230**, 111277 (2021).

[12] Hou, G. et al. Life cycle assessment of grid-connected photovoltaic power generation from crystalline silicon solar modules in China. *Appl. Energy* **164**, 882–890 (2016).

[13] Yu, Z. et al. Life cycle assessment of grid-connected power generation from a metallurgical route multi-crystalline silicon photovoltaic system in China. *Appl. Energy* **185**, 68–81 (2017).

[14] Mehedi, T. H., Gemechu, E. & Kumar, A. Life cycle greenhouse gas emissions and energy footprints of utility-scale solar energy systems. *Appl. Energy* **314**, 118918 (2022).

[15] Liu, D., Liu, J., Wang, S., Xu, M. & Akbar, S. J. Contribution of international photovoltaic trade to global greenhouse gas emission reduction: the example of China. *Resour., Conserv. Recy.* **143**, 114–118 (2019).

[16] Jiang, M., Gao, X., Guan, Q., Hao, X. & An, F. The structural roles of sectors and their contributions to global carbon emissions: A complex network perspective. *J. Cleaner Prod.* **208**, 426–435 (2019).

[17] Banerjee, S., Aamir Khan, M. & Iftikhar ul Husnain, M. Searching appropriate system boundary for accounting India’s emission inventory for the responsibility to reduce carbon emissions. *J. Environ. Manage.* **295**, 112907 (2021). 34157542 10.1016/j.jenvman.2021.112907

[18] IEA. Snapshot of Global PV Markets 2023. *International Energy Agency (IEA)* (Paris, 2023).

[19] IEA. Solar PV Global Supply Chains Analysis. *International Energy Agency (IEA)* (Paris, 2022).

[20] Helveston, J. P., He, G. & Davidson, M. R. Quantifying the cost savings of global solar photovoltaic supply chains. *Nature* **612**, 83–87 (2022). 36289345 10.1038/s41586-022-05316-6

[21] CPIA. China’s Photovoltaic Industry Development Roadmap (2018–2022). *China Photovoltaic Industry Association (CPIA**)* (2023).

[22] Ministry of Industry and Information Technology of the People’s Republic of China, Ministry of Science and Technology of the People’s Republic of China & Ministry of Natural Resources of the People’s Republic of China. *14th Five-Year Plan for the Development of the Raw Materials Industry (in Chinese**)*, https://www.gov.cn/zhengce/zhengceku/2021-12/29/content_5665166.htm(2021).

[23] Wang, Y. et al. Accelerating the energy transition towards photovoltaic and wind in China. *Nature* **619**, 761–767 (2023). 37495878 10.1038/s41586-023-06180-8 PMC10371865

[24] Cui, C., Lonergan, K. E. & Sansavini, G. Policy-driven transformation of global solar PV supply chains and resulting impacts. *Nat. Commun.* **16**, 6742 (2025). 40695823 10.1038/s41467-025-61979-5 PMC12284120

[25] Working Guidance for Carbon *Dioxide Peaking and Carbon Neutrality in Full and Faithful Implementation of the New Development Philosophy*, https://en.ndrc.gov.cn/policies/202110/t20211024_1300725.html (2021).

[26] Khawaja, M. K., Ghaith, M. & Alkhalidi, A. Public-private partnership versus extended producer responsibility for end-of-life of photovoltaic modules management policy. *Sol. Energy* **222**, 193–201 (2021).

[27] Jenkins, S., Kuijper, M., Helferty, H., Girardin, C. & Allen, M. Extended producer responsibility for fossil fuels*. *Environ. Res. Lett.* **18**, 011005 (2023).

[28] Heath, G. A. et al. Research and development priorities for silicon photovoltaic module recycling to support a circular economy. *Nat. Energy* **5**, 502–510 (2020).

[29] Wei, Y., Zhao, T., Wang, J., Zhang, X. & Li, Z. Two-level allocation and its future change of CO_2_ reduction responsibility in China’s power sector. *Environ. Impact Assess. Rev.* **99**, 107031 (2023).

[30] Gan, Y. et al. Greenhouse gas emissions embodied in the U.S. solar photovoltaic supply chain. *Environ. Res. Lett.* **18**, 104012 (2023).

[31] Tawalbeh, M. et al. Environmental impacts of solar photovoltaic systems: A critical review of recent progress and future outlook. *Sci. Total Environ.* **759**, 143528 (2021). 33234276 10.1016/j.scitotenv.2020.143528

[32] Kozul-Wright, R. China’s policy strategies for green low-carbon development: Perspective from South-South cooperation. *United Nations Conference on Trade and Development (UNCTAD)* (2023).

[33] Liang, H. & You, F. Reshoring silicon photovoltaics manufacturing contributes to decarbonization and climate change mitigation. *Nat. Commun.* **14**, 1274 (2023). 36890141 10.1038/s41467-023-36827-z PMC9995667

[34] Nijsse, F. et al. The momentum of the solar energy transition. *Nat. Commun.* **14**, 6542 (2023). 37848437 10.1038/s41467-023-41971-7 PMC10582067

[35] Quagraine, K. A., Lynas, M. & Ellis, E. C. As we breach 1.5 degrees C, we must replace temperature limits with clean-energy targets. *Nature* **649**, 1103–1106 (2026). 41588227 10.1038/d41586-026-00246-z

[36] Zhang, X., Zhu, Q., Li, X. & Pan, Y. The impact of government subsidy on photovoltaic enterprises' independent innovation based on the evolutionary game theory. *Energy* **285**, 129385 (2023).

[37] Ou, Y., Hsiao, C. Y.-L. & Chui, C. M. How does the supply chain market respond to policy shocks? Evidence from solar photovoltaic sectors in China. *Renew. Energ.* **232**, 121129 (2024).

[38] Ye, Q. et al. Allocating capital-associated CO_2_ emissions along the full lifespan of capital investments helps diffuse emission responsibility. *Nat. Commun.* **14**, 2727 (2023). 37169782 10.1038/s41467-023-38358-z PMC10173932

[39] Greenhouse Gas Protocol. Scope 3 Frequently Asked Questions. *World Resources Institute (WRI)*, *World Business Council for Sustainable Development (WBCSD)*, (2022).

[40] WRI & WBCSD. Product Life Cycle Accounting and Reporting Standard. *World Resources Institute (WRI), World Business Council for Sustainable Development (WBCSD)* (Washington, DC, 2011).

[41] Forster, E. J., Healey, J. R., Newman, G. & Styles, D. Circular wood use can accelerate global decarbonization but requires cross-sectoral coordination. *Nat. Commun.* **14**, 6766 (2023). 37880217 10.1038/s41467-023-42499-6 PMC10600095

[42] Zhang, Q. & Fang, K. Comment on “Consumption-based versus production-based accounting of CO_2_ emissions: Is there evidence for carbon leakage?. *Environ. Sci. Policy* **101**, 94–96 (2019).

[43] Schlömer S. *et al* Technology-specific cost and performance parameters. *Intergovernmental Panel on Climate Change (IPCC)*, (Cambridge University Press, 2014).

[44] Antonanzas, J. & Quinn, J. C. Net environmental impact of the PV industry from 2000–2025. *J. Cleaner Prod.* **311**, 127791 (2021).

[45] Wang, J., Feng, Y. & He, Y. Insights for China from EU management of recycling end-of-life photovoltaic modules. *Sol. Energy* **273**, 112532 (2024).

[46] Geng, Y., Sarkis, J. & Bleischwitz, R. How to build a circular economy for rare-earth elements. *Nature* **619**, 248–251 (2023). 37430110 10.1038/d41586-023-02153-z

[47] Wang, M. et al. Breaking down barriers on PV trade will facilitate global carbon mitigation. *Nat. Commun.* **12**, 6820 (2021). 34819496 10.1038/s41467-021-26547-7 PMC8613243

[48] IEA. Renewables 2023. *International Energy Agency (IEA)* (Paris, 2024).

[49] Wang, C. et al. Looming challenge of photovoltaic waste under China’s solar ambition: A spatial–temporal assessment. *Appl. Energy* **307**, 118186 (2022).

[50] Sun, L.-L., Cui, H.-J. & Ge, Q.-S. Will China achieve its 2060 carbon neutral commitment from the provincial perspective?. *Adv. Clim. Change Res.* **13**, 169–178 (2022).

[51] IEA. Will new PV manufacturing policies in the United States, India and the European Union create global PV supply diversification? *International Energy Agency (IEA)* (Paris, 2022).

[52] Kittner, N., Lill, F. & Kammen, D. M. Energy storage deployment and innovation for the clean energy transition. *Nat. Energy* **2**, 17125 (2017).

[53] Energy Storage Industry White Paper 2024. *China Energy Storage Alliance*, (2024).

[54] Bullich-Massagué, E. et al. A review of energy storage technologies for large scale photovoltaic power plants. *Appl. Energy* **274**, 115213 (2020).

[55] Carley, S. & Konisky, D. M. The justice and equity implications of the clean energy transition. *Nat. Energy* **5**, 569–577 (2020).

[56] Xing, Z., Ma, Y., Luo, L. & Wang, H. Harmonizing economies and ecologies: Towards an equitable provincial carbon quota allocation for China’s peak emissions. *Humanit. Soc. Sci. Commun.* **11**, 964 (2024).

[57] Huang, Y., Fang, K., Liu, G. & Guo, S. Has the carbon emission trading scheme induced investment leakage in China? Firm-level evidence from China’s stock market. *Energy Econ*. **141**, 108091 (2025).

[58] Willis, B. L. et al. Maximising environmental savings from silicon photovoltaics manufacturing to 2035. *Nat. Commun.* **17**, 2311 (2026). 41634031 10.1038/s41467-026-69165-x PMC12976067

[59] Peters, I. M., Hauch, J. A. & Brabec, C. J. The role of innovation for the economy and the sustainability of photovoltaic modules. *iScience* **25**, 105208 (2022). 36248740 10.1016/j.isci.2022.105208 PMC9557026

[60] Chen, S. et al. Deploying solar photovoltaic energy first in carbon-intensive regions brings gigatons more carbon mitigations to 2060. *Commun. Earth Environ.* **4**, 369 (2023).

[61] Herman, K. S., Hall, J. K., Sovacool, B. K. & Iskandarova, M. The industrial decarbonization paradigm: carbon lock-in or path renewal in the United Kingdom?. *Ecol. Econ.* **235**, 108628 (2025).

[62] Liu, Z. et al. CO(2) Sequestration Technologies May Undermine China’s Sustainable Development Goals through the Climate-Energy-Air-Health Cascade. *Environ. Sci. Technol.* **59**, 23–34 (2025). 39740060 10.1021/acs.est.4c09545

[63] Wu, Z., Wang, X., Tan, Y., Wu, X. & Fang, K. A typology of energy scaling in Chinese cities and its implication for sustainable urban transitions. *Sustainable Cities Soc*. **138**, 107168 (2026).

[64] Corwin, S. & Johnson, T. L. The role of local governments in the development of China’s solar photovoltaic industry. *Energy Policy* **130**, 283–293 (2019).

[65] Yu, S., Lu, T., Hu, X., Liu, L. & Wei, Y.-M. Determinants of overcapacity in China’s renewable energy industry: Evidence from wind, photovoltaic, and biomass energy enterprises. *Energy Econ* **97**, 105056 (2021).

[66] China *Announces 2035 Nationally Determined Contribution, Embarking on a New Journey to Address Climate Change (in Chinese**)* https://www.gov.cn/yaowen/liebiao/202509/content_7042502.htm (2025).

[67] Shao, C., Lv, C., Ning, J., Gao, Y. & Cui, Y. The impact of China’s manufacturing industry transfer on the comprehensive efficiency of pollution and carbon emissions reduction: an empirical analysis using a spatial panel model. *Energy Econ* **148**, 108624 (2025).

[68] Li, C., Mao, M., Li, R., Wiloso, E. I. & Fang, K. College students’ willingness to pay for carbon-labeled drinks. *Carbon Footprints* **4**, 37 (2025).

[69] Dong, C., Zhou, R. & Li, J. Rushing for subsidies: the impact of feed-in tariffs on solar photovoltaic capacity development in China. *Appl. Energy* **281**, 116007 (2021).

[70] Akyazi, T., Goti, A., Bayón, F., Kohlgrüber, M. & Schröder, A. Identifying the skills requirements related to industrial symbiosis and energy efficiency for the European process industry. *Environ. Sci. Eur.* **35**, 54 (2023).

[71] Spillias, S., Kareiva, P., Ruckelshaus, M. & McDonald-Madden, E. Renewable energy targets may undermine their sustainability. *Nat. Clim. Change* **10**, 974–976 (2020).

[72] Shi, H. et al. Critical mineral constraints pressure energy transition and trade toward the Paris Agreement climate goals. *Nat. Commun.* **16**, 4496 (2025). 40368945 10.1038/s41467-025-59741-y PMC12078721

[73] Fuso Nerini, F. et al. Mapping synergies and trade-offs between energy and the Sustainable Development Goals. *Nat. Energy* **3**, 10–15 (2018).

[74] Geng, T. et al. Increased occurrences of consecutive La Niña events under global warming. *Nature* **619**, 774–781 (2023). 37495880 10.1038/s41586-023-06236-9 PMC10371868

[75] Mohammed, A. et al. Performance evaluation and feasibility analysis of a 10 kWp PV system for residential buildings in Saudi Arabia. *Sustainable Energy Technol. Assess.* **51**, 101920 (2022).

[76] Ansanelli, G., Fiorentino, G., Tammaro, M. & Zucaro, A. A life cycle assessment of a recovery process from end-of-life photovoltaic panels. *Appl. Energy* **290**, 116727 (2021).

[77] Wang, L. et al. Carbon emissions and reduction performance of photovoltaic systems in China. *Renew. Sustain. Energy Rev.* **200**, 114603 (2024).

