# Supplementary material for: Strategic timing for the decarbonization contributions by China’s photovoltaic manufacturers in global energy transition
Source: Nat Commun. 2026 Aug 6;17:9465. doi: 10.1038/s41467-026-76424-4 (PMC13542174; doi:10.1038/s41467-026-76424-4)
Supplement: Supplementary file 1 [file 41467_2026_76424_MOESM1_ESM.pdf]

Supplementary Information for

Strategic Timing for the Decarbonization Contributions by China's Photovoltaic Manufacturers in Global Energy Transition

Qingyang Lin[1, #], Chenglin Li[2, #], Kai Wang[1, #], Kai Fang[2,3,4]*, Meixuan Xin[2], Siqi Wang[2,5], Anqi Xu[2,6], Zhenci Xu[7], Yue Lu[8], Xiang Gao[1,9]*

[1] State Key Laboratory of Clean Energy Utilization, Zhejiang University, Hangzhou 310027, China

[2] School of Public Affairs, Zhejiang University, Hangzhou 310058, China

[3] Institute for Public Policy, Zhejiang University, Hangzhou 310058, China

[4] Lab of Inclusive & Smart Governance for Urban-Rural Integration, Zhejiang University, Hangzhou 310058, China

[5] School of Environment and Safety Engineering, Qingdao University of Science Technology, Qingdao 266042, China

[6] School of Economics and Management, Zhejiang Sci-Tech University, Hangzhou 310018, China

[7] Department of Geography, The University of Hong Kong, Hong Kong 999077, China

[8] School of International Trade and Economics, University of International Business and Economics, Beijing 100105, China

[9] Dalian University of Technology, Dalian 116024, China

[#] These authors contributed equally: Qingyang Lin, Chenglin Li, Kai Wang

[*] Corresponding email: fangk@zju.edu.cn; xgao1@zju.edu.cn

Contents

Supplementary Notes

Supplementary Note 1 4
Supplementary Note 2 6
Supplementary Note 3 11
Supplementary Note 4 13
Supplementary Note 5 18
Supplementary Note 6 18

Supplementary Figures

Supplementary Figure 1 4
Supplementary Figure 2 5
Supplementary Figure 3 10
Supplementary Figure 4 12
Supplementary Figure 5 14
Supplementary Figure 6 16
Supplementary Figure 7 17
Supplementary Figure 8 18
Supplementary Figure 9 19
Supplementary Figure 10 19
Supplementary Figure 11 20
Supplementary Figure 12 20
Supplementary Figure 13 21
Supplementary Figure 14 22
Supplementary Figure 15 22
Supplementary Figure 16 23
Supplementary Figure 17 24
Supplementary Figure 18 25
Supplementary Figure 19 26
Supplementary Figure 20 26
Supplementary Figure 21 26

Supplementary Tables

Supplementary Table 1 6
Supplementary Table 2 6
Supplementary Table 3 7
Supplementary Table 4 8
Supplementary Table 5 11
Supplementary Table 6 14
Supplementary Table 7 15
Supplementary Table 8 17
Supplementary Table 9 27
Supplementary Table 10 27

Supplementary References 28

Supplementary Note 1. Life cycle assessment for the photovoltaic (PV) system

Decarbonization contribution by photovoltaic (PV) manufacturers

In this study, we assess producer decarbonization contribution aiming at determining manufacturers' decarbonization contributions across the life cycle based on three criteria: energy, economy, and carbon emissions (see Supplementary Fig. 1).

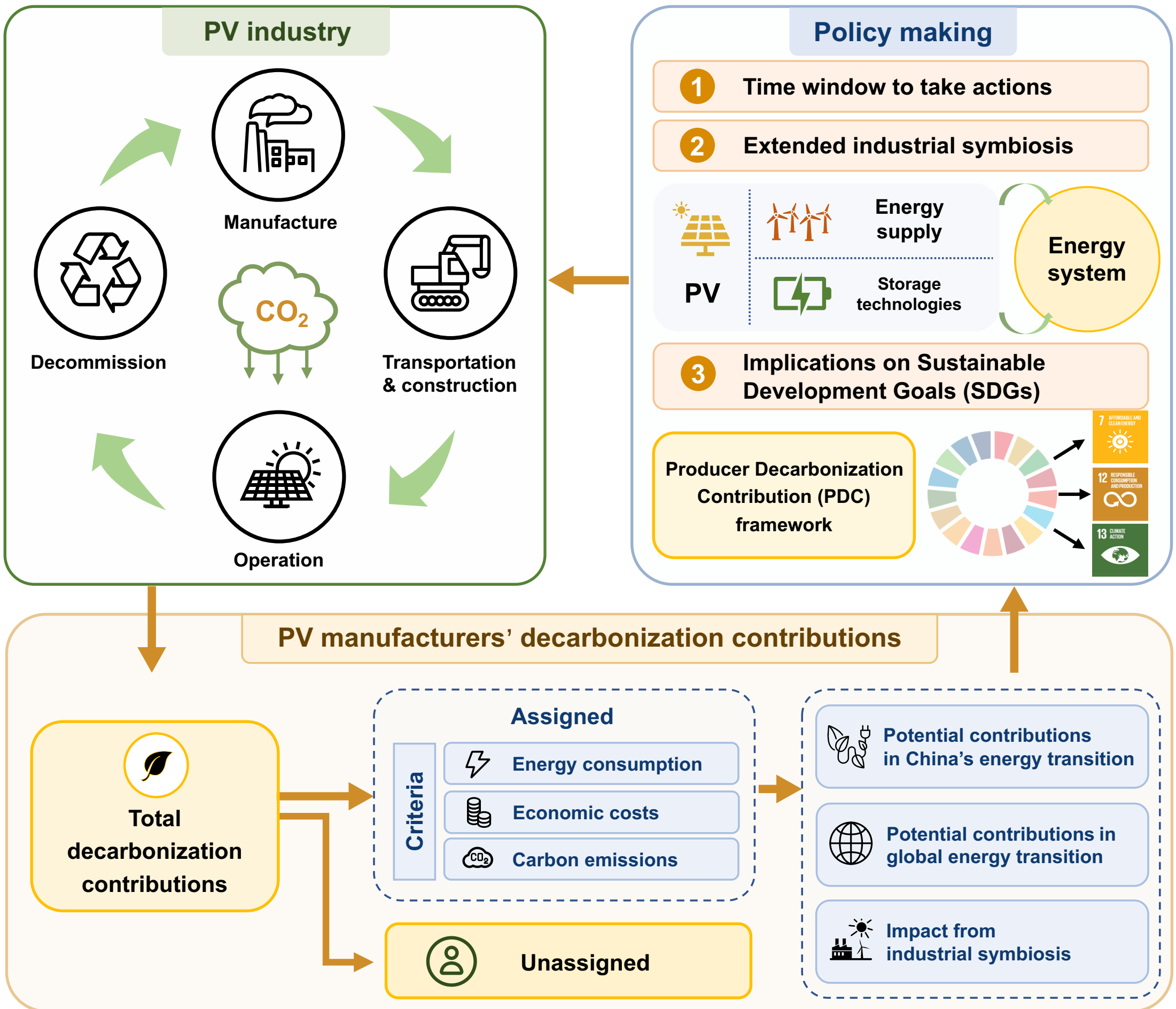

Supplementary Figure 1. Photovoltaic (PV) manufacturers' decarbonization contributions. The Sustainable Development Goals (SDGs) icons and colour wheel are used in accordance with the United Nations Sustainable Development Goals branding guidelines. The content of this publication has not been approved by the United Nations and does not reflect the views of the United Nations or its officials or Member States. Further information is available from the United Nations Sustainable Development Goals website: https://www.un.org/sustainabledevelopment.

The accounting boundary.

We have outlined three specific areas of focus. The first area, referred to as Layer I, examines the production steps involved in creating PV modules. These steps encompass five key processes: making industrial-Si, polysilicon, silicon wafers, cells, and PV panels. Moving on to Layer II, we analyze a 1 MWp mono-crystalline PV system that operates for a span of 30 years. This assessment covers various stages, including manufacturing (like PV panels and inverters), transportation, construction,

operation, and decommissioning processes. The third area, known as Layer III, explores the reduction in carbon emissions achieved by transitioning from fossil fuel-based electricity generation to solar PV. This analysis goes beyond the conventional Scopes 1, 2, and 3 of the Greenhouse Gas (GHG) Protocol and takes into account these specific boundaries. Layer II encompasses Scopes 1, 2, and 3, whereas Layer III further incorporates decarbonization. This makes it possible to recognize the decarbonization contributions of PV manufacturers, which are conceptually distinct from emission responsibilities. For a more detailed overview of these layers, please refer to Supplementary Fig. 2.

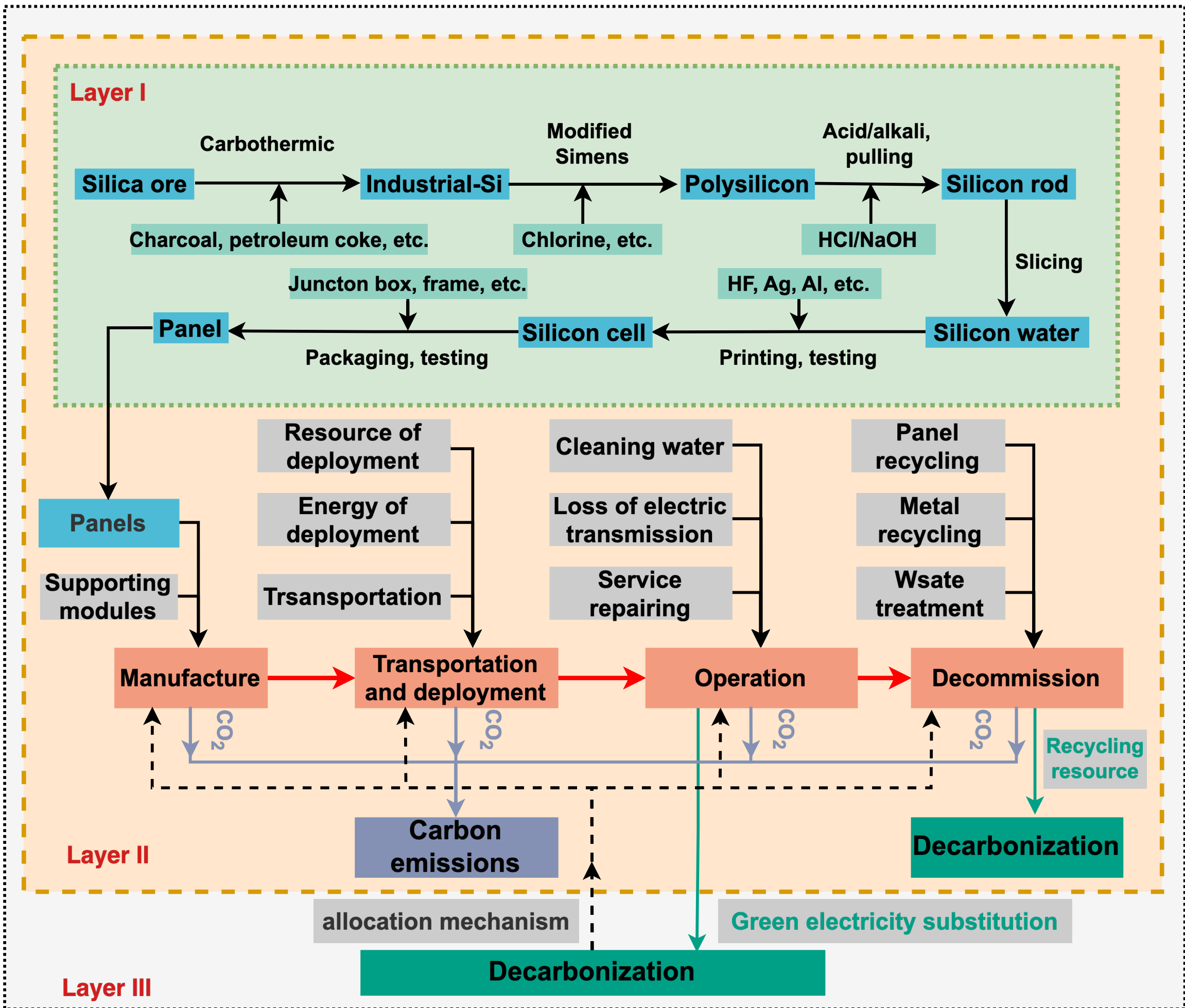

Supplementary Figure 2. Producer decarbonization contribution (PDC) framework.

We use a 1 MWp mono-crystalline PV system as our functional unit and typical PV system for several reasons. First, a 1 MWp system represents a suitable-sized PV installation that is commonly found in industrial parks and can also serve as a unit module for large-scale centralized PV systems. The use of 1 MWp as a functional unit is well-established in life cycle assessment (LCA) studies of PV systems, providing a balance between detail and generality. Moreover, mono-crystalline silicon modules have become the mainstream technology in the PV market. By 2023, the market share of mono-crystalline silicon wafers (both p-type and n-type) had exceeded 99% in the Chinese market, with p-type mono-crystalline silicon wafers alone accounting for 74.5% of the market.

The specifications of the PV system

The PV panels used in this study are assumed to be of the Passivated Emitter and Rear Cell (PERC) type. These panels have a single glass layer and are held in a fixed position (non-tracking).

The performance details of these PV panels can be found in Supplementary Table 1. This information is sourced from the PV panel specification sheets provided by LONGi Green Energy [1] and Trina Solar [2] between the years 2018 and 2022.

Supplementary Table 1. Performance characteristics of PV modules.

Year	Weight (kg)	Cell efficiency	Module efficiency	Size of cell (mm)	Modules area (m^2)	Border length (m)	Wafer Thickness (um)	Power (W)
2018	18	21.80%	18.60%	156-158	1.683	5.372	180	305
2019	18	22.30%	19.60%	158-166	1.855	5.638	175	330
2020	25	22.80%	20.60%	158-166	2.208	6.314	175	450
2021	32.4	23.10%	21.70%	166-182	2.558	6.778	170	545
2022	32.6	23.20%	22.50%	182-210	2.581	6.820	155	550

Note: The photovoltaic (PV) modules used in this study are assumed to use Passivated Emitter and Rear Cell (PERC) type of panels with single glass and non-tracking holder. The data is based on the PV panel specification sheets released by LONGi Green Energy and Trina Solar from 2018-2022 [1-3].

The manufacturing process of these PV modules involves five main stages: Industrial Silicon Production - carbon thermal reduction to produce industrial silicon. Polysilicon Production: - the modified Siemens process is employed to create polysilicon. Silicon Wafer Production - through the processes of pulling and slicing. Cell Production - the manufacturing of solar cells. PV Panel Assembly - Components are assembled to produce the final PV panels.

Supplementary Note 2. The life cycle inventory (LCI) for a 1 MWp PV system

The LCIs of photovoltaic (PV) panels throughout their manufacturing phase mainly come from the Annual Development Report of China's Photovoltaic Industry from 2018 to 2023, the Photovoltaic Power Systems Programme (PVPS) by International Energy Agency (IEA), industrial standard, industrial reports and literature [3-10]. Diesel consumption is during the mineral extraction process, referring to the energy consumption limit values for large-scale mining operations [6]. Additionally, Supplementary Table 2 presents the LCI concerning the manufacturing of supporting modules [11-13].

Supplementary Table 2. The Life Cycle Inventory (LCI) of supporting modules.

	Materials	Unit	Consumption
Inverter	Steel	kg	5,760
	Copper	kg	1,340
	Aluminum	kg	524
	Polyethylene (PE)	kg	88
	Polyamides (PA)	kg	284
	Lube	kg	3,524
	Polyester resin	kg	176
	Electricity	kWh	18,320
Holder	Steel	kg	8,410
	Aluminum	kg	8,410
Junction box	Acrylonitrile Butadiene Styrene (ABS)	kg	693
	Copper wire	kg	693

Cable	Copper	kg	4,360
	Insulating coating	kg	3,000

Note: The inverter has a life span of 15 years. The 1 MWp photovoltaic (PV) system is equipped with two inverters, and four inverters would be required over the 30-year operating cycle of the PV system. The inventory of four inverters is shown in Supplementary Table 2.

The assumptions and data sources for the transportation, construction, operation, and decommissioning stages of the PV system are described below. Transportation stage includes the transportation of mid-product (such as Quartzite ore, silicon wafers), the transportation of PV panels and the transportation of supporting modules (such as inverter, PV holder, cable, junction box), and the transportation distance is assumed according to Longi Green Energy (one of the largest photovoltaic industry companies in China) photovoltaic industry factory layout. Assuming that the distance between the silicon mine and the processing plant is 30 km, and transportation is carried out by heavy trucks with a maximum load weight of 15 t. The production sites for industrial silicon, polysilicon, and silicon wafers are located in Yinchuan, Ningxia, while cell production and PV panel manufacturing are situated in Taizhou, Jiangsu, and PV panels are installed in Suzhou, Jiangsu. The silicon wafer is transported over a distance of 1800 km by railway, while the transportation of PV panels is carried out by heavy trucks with a maximum load weight of 15 t over a distance of 170 km. Accessories such as inverters are assumed to be produced locally and are transported by heavy trucks over a distance of 30 km. The LCI of the construction stage comes from the literature [14,15]. The operational failure rate of PV panels is exceedingly low, estimated to be around 5 per 10,000 (US Renewable Energy Laboratory), rendering it negligible [14]. Therefore, only cleaning water consumption during operation is counted. Decommissioning stage includes the recycling of waste panels, the recycling of other metal and the disposal of non-recyclable materials. We assume that the aluminum frame of the photovoltaic panel is completely recycled after being removed, and the recovery process of others materials during recycling waste panel refers to the project of Recovery of Silicon and other materials from End-of-Life Photovoltaic Panels (ReSiELP) [16].

Supplementary Table 3 shows the recovery rate of main materials at the recycling process. In this study, we assume the complete recovery of inverters, photovoltaic holder and cables, which hold high recycling value. The recovery rates for other materials in PV panels are calculated based on data from the literature [16]. The inverter, holder, junction box, and cable also contained valuable metals, with an assumed recovery rate of 90% in this study. To better comprehend the ecological aspects, Supplementary Table 4 provides the carbon emission coefficients of the resources and energy consumed within this study. The ensuing step involves the computation of the total carbon emissions and the carbon intensity per kilowatt-hour (kWh) for a 1 MWp PV system across various production levels spanning from 2018 to 2022. These outcomes are depicted in Supplementary Fig. 3. Inclusively, the energy losses linked with electricity transmission during the process (as detailed in Supplementary Table 5) are considered. Supplementary Note 6 encompasses the accompanying sensitivity analysis.

Supplementary Table 3. The recovery rate of main materials.

Item	Materials	The recovery rate
Photovoltaic (PV) panels	Aluminum frame	100%
	Copper	84.3%
	Silicon	88.4%
	Glass	93.5%

	Silver	90%
	Plastic (including Tedlar–polyethylene terephthalate–Tedlar [TPT] and ethylene-vinyl acetate [EVA])	15.0%
Inverter	Steel	90%
	Copper	90%
	Aluminum	90%
Holder	Steel	90%
	Aluminum	90%
Junction box	Copper	90%
Cable	Copper	90%

Supplementary Table 4. The carbon coefficients of resources, energy consumption and recycling materials.

Materials	Unit	CO_2	CH_4	N_2O	SF_6	Sum
Electricity (China's grid)	g CO_2 eq kWh^{-1}	5.8E-01	4.6E-02	2.7E-03	1.1E-05	6.3E-01
Steam (180℃ 1.0 MPa steam from hard coal (85%))	g CO_2 eq kWh^{-1}	3.2E-01	2.7E-02	1.4E-03	4.2E-08	3.5E-01
Aluminum	g CO_2 eq kWh^{-1}	2.0E+01	1.5E+00	8.5E-02	8.9E-06	2.2E+01
Steel	g CO_2 eq kWh^{-1}	2.1E+00	1.5E-01	1.8E-03	1.4E-06	2.3E+00
Copper	g CO_2 eq kWh^{-1}	4.0E+00	1.2E-01	4.0E-02	1.3E-05	4.2E+00
Silver	g CO_2 eq kWh^{-1}	6.8E+01	2.9E+00	5.0E-01	2.8E-04	7.1E+01
Polyethylene (PE)	g CO_2 eq kWh^{-1}	3.0E+00	2.8E-01	1.5E-02	1.0E-05	3.3E+00
Ethylene-vinyl acetate (EVA)	g CO_2 eq kWh^{-1}	2.0E+00	3.7E-01	5.5E-03	6.4E-06	2.4E+00
Polyester resin	g CO_2 eq kWh^{-1}	2.2E+00	2.8E-01	6.3E-03	4.4E-06	2.5E+00
Polyamides (PA)	g CO_2 eq kWh^{-1}	8.3E+00	5.1E-01	4.1E-01	2.7E-05	9.2E+00
Polyethylene glycol	g CO_2 eq kWh^{-1}	2.1E+00	2.4E-01	2.4E-02	5.0E-06	2.4E+00
Glass	g CO_2 eq kWh^{-1}	1.3E+00	1.2E-01	3.2E-03	1.2E-08	1.5E+00
Sealing silicone	g CO_2 eq kWh^{-1}	1.0E+01	4.6E-01	5.2E-02	7.6E-05	1.1E+01
Insulating coating	g CO_2 eq kWh^{-1}	4.8E+00	2.7E-01	4.1E-02	7.3E-05	5.1E+00
Acrylonitrile–butadiene–styrene (ABS)	g CO_2 eq kWh^{-1}	2.6E+00	2.5E-01	1.9E-02	5.7E-06	2.9E+00
Tedlar–polyethylene terephthalate–Tedlar (TPT)	g CO_2 eq kWh^{-1}	1.1E+01	6.6E+00	5.7E-02	1.0E-04	1.7E+01
Polylactic acid (PLA)	g CO_2 eq kWh^{-1}	5.4E+00	2.5E-01	3.1E-01	3.2E-05	6.0E+00
Citric acid	g CO_2 eq kWh^{-1}	8.6E+00	3.8E-01	1.6E-01	3.0E-05	9.1E+00
Hard coal	g CO_2 eq kWh^{-1}	1.0E-01	2.1E-01	6.2E-04	3.3E-07	3.1E-01
Petroleum coke	g CO_2 eq kWh^{-1}	3.0E-01	8.6E-02	1.4E-03	1.9E-07	3.8E-01
Cooling water (Deionized water)	g CO_2 eq kWh^{-1}	1.8E-03	1.6E-04	1.6E-05	1.2E-08	2.0E-03
Tap water	g CO_2 eq kWh^{-1}	1.7E-04	1.5E-05	2.0E-06	1.2E-08	1.8E-04
NH_3	g CO_2 eq kWh^{-1}	5.1E+00	3.6E-01	1.4E-01	3.3E-06	5.6E+00
O_2 (l)	g CO_2 eq kWh^{-1}	4.0E-01	1.2E-02	4.4E-03	1.2E-05	4.1E-01

N_2 (g)	g CO_2 eq kWh^{-1}	1.1E-01	9.2E-03	5.3E-04	9.3E-07	1.2E-01
Detergent	g CO_2 eq kWh^{-1}	6.6E-01	6.6E-02	4.8E-03	4.1E-10	7.3E-01
HNO_3 (70%)	g CO_2 eq kWh^{-1}	3.7E-01	2.8E-02	6.7E-01	4.3E-07	1.1E+00
Sawdust	g CO_2 eq kWh^{-1}	6.1E-02	3.3E-03	1.2E-03	6.4E-06	6.6E-02
$POCI_3$	g CO_2 eq kWh^{-1}	3.6E+00	2.3E-01	1.0E-02	2.2E-05	3.8E+00
KOH (70%)	g CO_2 eq kWh^{-1}	9.0E-01	7.6E-02	2.5E-03	5.7E-06	9.8E-01
NaOH (30%)	g CO_2 eq kWh^{-1}	4.4E-01	1.9E-02	2.9E-03	7.6E-06	4.7E-01
Diamond wire (by GaBi)	kg CO_2 eq m^{-1}	5.6E-04	3.4E-05	9.3E-06	2.3E-09	6.1E-04
H_2SO_4 (98%)	g CO_2 eq kWh^{-1}	2.2E-01	3.5E-02	2.5E-03	1.3E-06	2.6E-01
HCI（37%)	g CO_2 eq kWh^{-1}	3.9E-01	1.7E-02	3.5E-03	7.6E-06	4.1E-01
HF (40%)	g CO_2 eq kWh^{-1}	1.3E+00	1.0E-01	8.4E-03	9.2E-06	1.4E+00
Glue	g CO_2 eq kWh^{-1}	9.1E-02	3.7E-03	5.9E-04	4.7E-07	9.5E-02
Cl_2	g CO_2 eq kWh^{-1}	9.6E-01	3.6E-02	3.0E-03	1.8E-05	1.0E+00
H_2	g CO_2 eq kWh^{-1}	1.4E+01	5.4E-01	1.9E-02	3.7E-05	1.4E+01
Anhydrous ethanol	g CO_2 eq kWh^{-1}	2.3E+00	1.9E-01	1.3E-02	1.2E-06	2.5E+00
Lube	g CO_2 eq kWh^{-1}	1.6E+00	2.1E-01	2.2E-03	-	1.8E+00
Charcoal	g CO_2 eq kWh^{-1}	1.4E+00	9.1E-03	3.1E-03	1.9E-07	1.4E+00
Graphite	g CO_2 eq kWh^{-1}	7.0E-01	6.0E-02	3.3E-03	5.6E-06	7.6E-01
Transport by truck	kg CO_2 eq t^{-1} km^{-1}	1.1E-01	4.1E-03	1.9E-04	1.1E-08	1.2E-01
Transport by rail	kg CO_2 eq t^{-1} km^{-1}	1.2E-02	9.7E-04	5.7E-05	2.4E-07	1.3E-02
Sewage treating	g CO_2 eq kWh^{-1}	5.2E-04	5.2E-04	5.8E-05	1.8E-09	1.1E-03
Waste water treating	g CO_2 eq kWh^{-1}	7.3E-04	1.8E-04	6.9E-05	4.9E-09	9.8E-04
Building materials treating	g CO_2 eq kWh^{-1}	1.6E-02	6.2E-04	7.6E-05	5.4E-08	1.6E-02
Solid waste treating	g CO_2 eq kWh^{-1}	2.9E+00	7.6E-02	7.8E-03	1.2E-05	3.0E+00
Concrete	g CO_2 eq kWh^{-1}	7.3E-02	2.8E-03	1.0E-04	7.6E-08	7.6E-02
Recycling aluminum	g CO_2 eq kWh^{-1}	-1.8E+01	-2.1E+00	-7.6E-02	-6.3E-06	-2.0E+01
Recycling steel	g CO_2 eq kWh^{-1}	-1.6E+00	-1.1E-01			-1.7E+00
Recycling silicon	g CO_2 eq kWh^{-1}	-1.5E+01	-5.7E-01	-3.7E-02	-6.3E-05	-1.5E+01
Recycling copper (Include direct emissions 0.257)	g CO_2 eq kWh^{-1}	-3.0E+00	-7.2E-02	-3.3E-02	-9.3E-06	-2.9E+00
Recycling silver	g CO_2 eq kWh^{-1}	-	-	-	-	-7.0E+01
Recycling glass	g CO_2 eq kWh^{-1}	-	-	-	-	-1.4E+00
Recycling sealing silicone	g CO_2 eq kWh^{-1}	-	-	-	-	-1.0E+01

Note: GaBi databases (GaBi: https://gabi.sphera.com) cover the carbon emissions coefficients of the main resources and energy consumption of each unit process (such as electricity, aluminum, steel, etc.) [17]. In particular, the data for China is used preferentially, and the data for those materials not included in the database comes from papers. The coefficients of recycling materials refer to the second product by using the waste, such as aluminum, steel, copper, glass and silicone. Supplementary Table 4 shows all associated carbon coefficients, including four greenhouse gases with CO_2 equivalent coefficients from the Sixth Assessment Report (IPCC) [18].

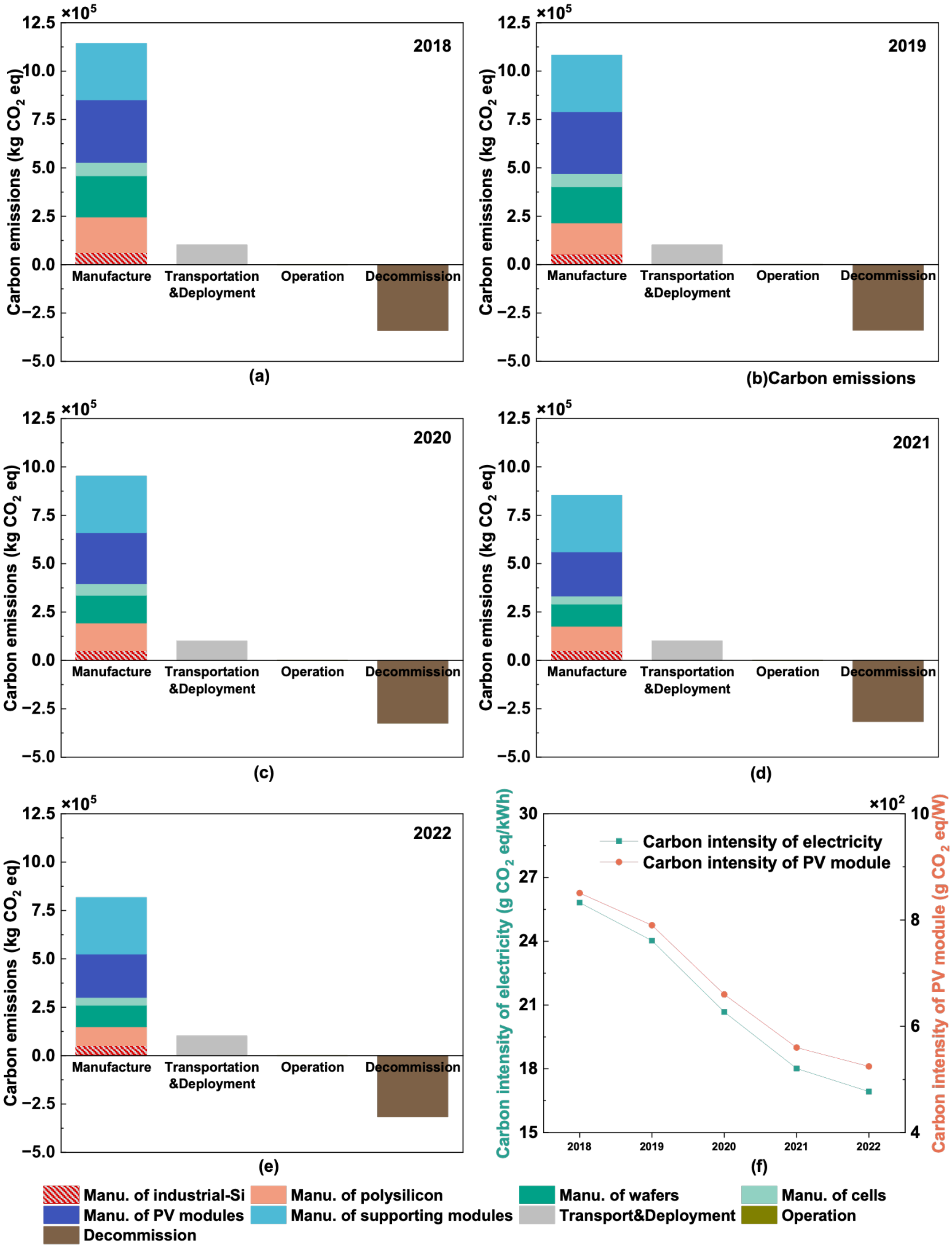

Supplementary Figure 3. Carbon emissions and intensity of electricity of photovoltaic (PV) system. (a-e) The carbon emissions for a 1 MWp PV system from cradle to grave at the years of 2018-2022. **(f)** The carbon intensity of electricity from solar PV for the year of 2018-2022.

Note: The performance parameters of the PV module from 2018-2022 are provided in Supplementary Table 1, while the global tilted irradiance at optimum angle in China ranges from 986 to 2529 kWh m^{-2} per year, with an area-weighted average of 1756 kWh m^{-2} per year. The loss coefficient of electricity transmission is presented in Supplementary Table 5 [19].

Supplementary Table 5. The loss coefficient of electricity transmission in year 2018-2022.

Year	Loss coefficient
2018	0.9373
2019	0.9407
2020	0.9440
2021	0.9474
2022	0.9516

Supplementary Note 3. Strategic timing for PV manufacturers' decarbonization contributions in China

To figure out the amount of decarbonization that can be allocated, the payback times for the system from the three criteria need to be calculated first. We then allocate the amount of decarbonization among the parts of the PV system based on the energy consumption, economic costs, and carbon emissions. The total life cycle of a photovoltaic (PV) system could be generally divided into nine processes: 1-industrial silicon, 2-solar-grade polysilicon, 3-silicon wafers, 4-cells, 5-PV modules, 6-supporting modules, 7-transportation and construction, 8-operation and 9-decommissioning. The processes of 1-6 are the manufacturing industry. The data of primary energy demand and carbon emissions are calculated by GaBi and its database based on the materials and energy consumption. The economy investment of PV systems and its distributions in China come from industrial report [3].

IEA Scenario

The Net Zero Emissions by 2050 (NZE) Scenario shows a narrow but achievable pathway for the global energy sector to achieve net zero CO_2 emissions by 2050, with advanced economies reaching net zero emissions in advance of the other scenarios. This scenario also meets key energy-related United Nations Sustainable Development Goals (SDGs), in particular achieving universal energy access by 2030. The NZE Scenario does not rely on emissions reductions from outside the energy sector to achieve its goals, but assumes that non-energy emissions will be reduced in the same proportion as energy emissions. It is consistent with limiting the global temperature rise to 1.5 °C without a temperature overshoot (with a 50% probability) [20].

The Announced Pledges Scenario (APS) takes account of all the climate commitments made by governments around the world including Nationally Determined Contributions as well as longer term net zero emissions targets, and assumes that they will be met in full and on time. The global trends in this scenario represent the cumulative extent of the world's ambition to tackle climate change as of mid-2022. The remaining difference in global emissions between the APS and the goals in the NZE Scenario shows the "ambition gap" that needs to be closed to achieve the goals agreed in the Paris Agreement in 2015 [20].

The Stated Policies Scenario (STEPS) does not take for granted that governments will reach all announced goals. Instead, it explores where the energy system might go without additional policy implementation. As with the APS, it is not designed to achieve a particular outcome. It takes a granular, sector-by-sector look at existing policies and measures and those under development. The remaining

difference in global emissions between the STEPS and the APS represents the "implementation gap" that needs to be closed for countries to achieve their announced decarbonization targets [20].

Payback time from the energy consumption criterion

We use primary energy demand (PED) as an indicator to evaluate the total energy consumption [21], including the direct electric consumption during manufacturing processes and the indirect energy consumption contained in consumed materials (e.g. aluminum, copper, steel, hydrochloric acid, etc.). During the life cycle of a 1 MWp PV system, the total energy consumption at all four stages is approximately 1.09×10^4 GJ. The energy consumption during the stages of manufacture, transportation, construction and operation is 1.26×10^4 GJ, with electricity being the primary contributor to the energy consumption, accounting for approximately 41.3%, while the energy consumption during the decommission stage is -1.76×10^3 GJ. During the operational phase, the PV system generates a total of approximately 1.28×10^5 GJ of electricity, resulting in payback time for energy consumption (T_{en}) of 2.7 years (by equation (7)). The difference of payback times for energy consumption in this study compared to the literature, varying from 1.2 to 4.9 years, is attributed to the utilization of the latest manufacturing performance data with improved energy utilization efficiency [10,22] and the difference in the scoping boundary [23].

Payback time from the economic costs criterion

We assume that all electricity generated by the PV system would feed into the grid with a fixed price of 0.454 CNY kWh^{-1} and consider only the revenue generated by the system. The discount rate varies from 0.1% to 5% to evaluate the financial feasibility of the system by computing the net present value. The cumulative revenue curve is calculated with a 1% discount rate, and the net present cost of a 1 MWp PV system in this study focuses on the initial investment during the early stage and the maintenance cost during the 30-year operation stage, which are 4.13 and 0.64 million CNY. The estimated payback time for economy (T_{eco}) is 8.2 years (by equation (8)), and it will be 7.79 and 10.2 years when the discount rate respectively changes to 0.1% and 5%, shown in Supplementary Fig. 4.

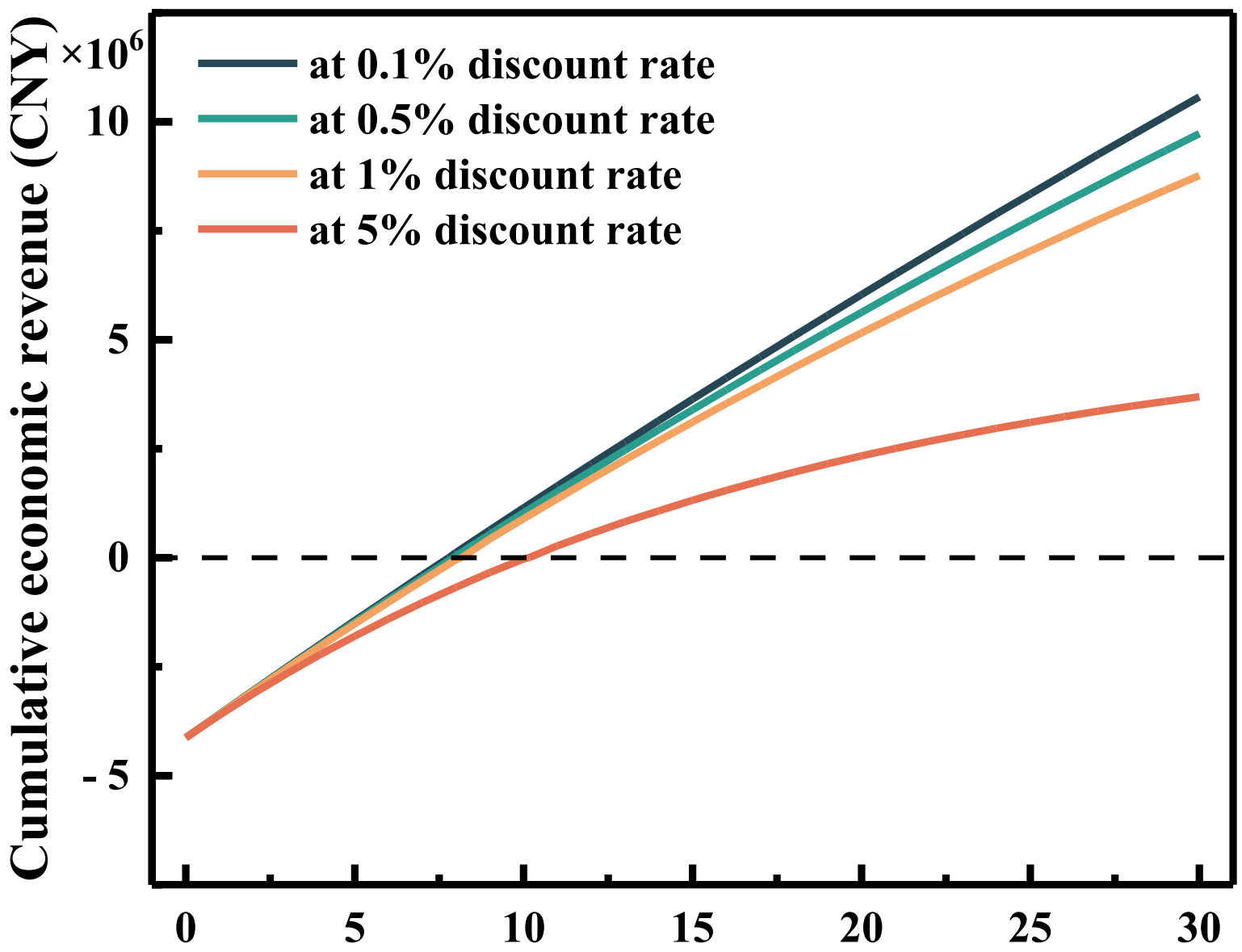

Supplementary Figure 4. The payback times for economy of PV in China at different discount

rate.

Note: Based on the electricity price and economic investment in China, we calculate the payback times for economy of photovoltaic (PV) system, varying the discount rate from 0.1% to 5%.

Payback time from the carbon emissions criterion

The payback time for carbon emissions (T_{em}) of a 1 MWp PV system could be determined by comparing the initial greenhouse gas (GHG) emissions (four types greenhouse gas including carbon dioxide (CO_2), methane (CH_4), nitrous oxide (N_2O) and sulfur hexafluoride (SF_6)) before decommission and avoided GHG emissions by generating electricity for the grid in China during the life cycle (two electricity structure scenarios), which are 920 and -1.28×10^4 (STEPS) and -1.08×10^4 (APS) tCO_2 eq from Fig. 2a, resulting T_{em} to be the same of 1.3 years (by equation (9)). Note that the carbon intensity of the grid in this study is the average intensity from different sources (e.g. PV, wind, coal, etc.) which is determined by the electricity composition and carbon emission intensity for each energy resource.

The value of carbon intensity of electricity generation from solar PV is based on the result of this paper shown in Supplementary Fig. 3. And the rest value of electricity generation from coal, natural gas, wind, modern bioenergy, hydro, nuclear, oil refers to the Fifth Report in Intergovernmental Panel on Climate Change (IPCC) [24]. The carbon intensity of electricity generation from fossil fuels with Carbon Capture, Utilization and Storage (CCUS) is assumed to be 10% of that from coal, and others (include concentrated solar power (CSP), geothermal, marine and hydrogen and ammonia) is assumed to be zero gCO_2 eq kWh^{-1}.

Supplementary Note 4. Strategic timing for PV manufacturers' decarbonization contributions at the global scale

On a global level, the payback periods of PV systems change in three different aspects. This study focuses on three scenarios for global electricity structures: STEPS, APS, and NZE. The time required to recover carbon emissions ranges from 1.5 to 1.6 years, while for energy and economic factors, it is 2.6 and 3.5 years, as calculated using equations (7)-(9). For a visual representation, see Supplementary Fig. 5, which illustrates payback times and decarbonization across these criteria. The variation in global payback times, compared to those in China, is primarily due to differences in average solar radiation intensity, electricity pricing, and grid carbon intensity. These variations arise from disparities in electricity supply structures. For a more detailed breakdown, refer to Supplementary Table 6, which presents global tilted irradiation (GTI) values at the optimal angle and electricity carbon intensity from solar PV for 18 representative countries [25]. Furthermore, we derive the global average of GTI and carbon intensity for PV systems by considering the weighted by the administrative areas of these 18 countries. At a global scale, we assume that the energy and carbon emission inputs for PV systems are consistent with those in China except for the transportation process. For the transportation process, there is an extra long-distance transport process from China to other countries. Supplementary Table 7 presents an uncertainty analysis of transport distances, with an ultimate selection of an average transport distance of 16000 km. The departure point is Ningbo-Zhoushan Port in China. The Netherlands, India, Brazil and Mexico are the main export destinations for Chinese solar panels [26]. As for the economic dimension, the average investment cost globally is

6.06×10⁶ CNY MWp^{-1} [27], assuming that the proportions of investment from industrial production to PV plant construction are the same as in China. The input during the operation and decommissioning stages of the photovoltaic plant is also assumed to be the same as in China.

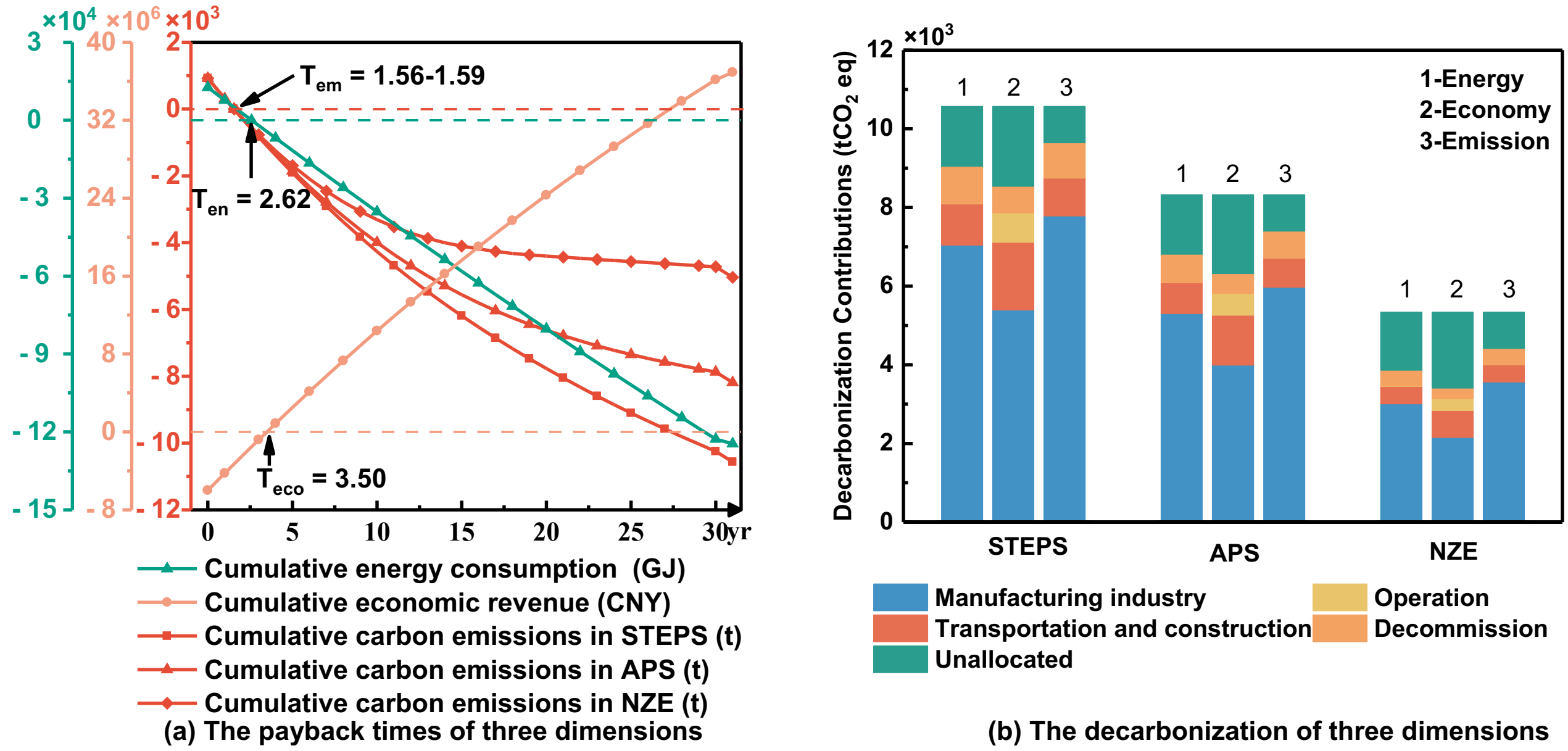

Supplementary Figure 5. The payback times and decarbonization of three dimensions at the global scale. (a) The payback times from the criteria of energy, economy and carbon emissions for a 1 MWp photovoltaic (PV) system operating for 30 years at the global scale. **(b)** The distribution of decarbonization contributions over PV lifecycle in three criteria and scenarios for 1 MWp PV system at the global scale.

Supplementary Table 6. Global tilted irradiation (GTI) from solar photovoltaic (PV) for 18 representative countries.

Number	Country	GTI (kWh m^{-2} $year^{-1}$)
1	China	1,756
2	United States of America	1,916
3	Japan	1,482
4	Germany	1,281
5	India	2,008
6	Australia	2,307
7	Italy	1,730
8	Brazil	1,986
9	Netherlands	1,237
10	Korea	1,668
11	Saudi Arabia	2,471
12	South Africa	2,310
13	Congo	1,913
14	Spain	1,949
15	Argentina	2,106
16	Kazakhstan	1,661

17	Russian Federation	1,427
18	Islamic Republic of Iran	2,267
	The global average	1,855

Note: Countries numbered 1-10 represent the top ten countries in terms of newly installed photovoltaic capacity in 2021, while countries numbered 11-18 represent the global geographical scope.

Supplementary Table 7. The uncertainty analysis of transportation distances at global scale.

Destination	Distances (km)	Increase in primary energy demands	Increase in carbon emissions
Port of Mumbai (India)	8534	2.0%	2.7%
Port of Mazatlan (Mexico)	12307	2.8%	4.0%
Port of Rotterdam (Netherlands)	19448	4.5%	6.3%
Port of Rio de Janeiro (Brazil)	23782	5.5%	7.7%

The payback times from the primary energy demand criterion

For the payback time for energy consumption (T_{en}) at the global scale, we assume the energy consumption to be the same as that producing in China, and the annual electricity generations of PV system is with a global average solar irradiation intensity. Based on the above assumptions, T_{en} at the global scale is calculated to be 2.6 years using equation (7).

The payback times from the economic costs criterion

In terms of the payback times for economy (T_{eco}), the initial investment for a globally averaged installed capacity photovoltaic system is 6.06×10^{6} CNY MWp^{-1} by IRENA [27]. The price of electricity for businesses ranges from 0.011 to 5.605 CNY kWh^{-1} [28]. To provide a clearer understanding of the electricity prices used in our analysis, for the context of China, we use an electricity price of 0.454 CNY kWh^{-1}. For global scenarios, sensitivity and uncertainty analyses are conducted with electricity prices ranging from 0.7 to 3.85 CNY kWh^{-1}. The final calculations are based on an electricity price of 1.33 CNY kWh^{-1}. We assume that the annual operation and maintenance costs of the photovoltaic system are the same as in China. Supplementary Fig. 6a shows the variations in economic investments for a 1 MWp photovoltaic system with a discount rate ranging from 0.1% to 5% when the electricity price is 1.33 CNY kWh^{-1}, and the T_{eco} is 3.4-3.8 years. Supplementary Fig. 6b displays the variations in economic investments for a 1 MWp photovoltaic system under different electricity prices ranging from 0.7 to 3.85 CNY kWh^{-1}, with the T_{eco} varying 1.2 to 7.0 years. In this study, we choose a case at the average electricity price (1.33 CNY kWh^{-1}) and discount rate (1%) at the global scale, resulting T_{eco} of 3.5 years by equation (8).

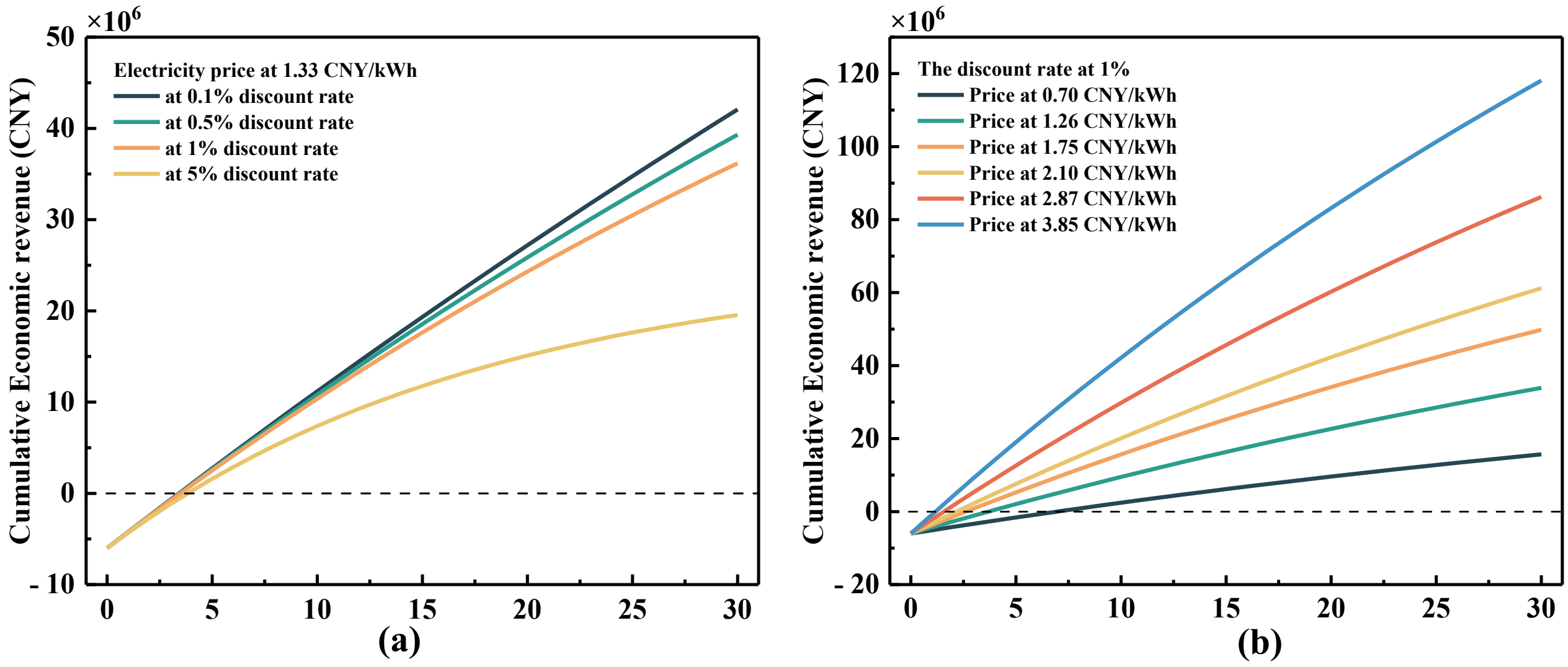

Supplementary Figure 6. The sensitivity analysis of the discount rate and electricity prices to payback times for economy. (a) The payback times for economy of PV system, varying the discount rate from 0.1% to 5% based on the economic investment at a global average level. **(b)** The payback times for economy of photovoltaic (PV) system, varying the electricity price from 0.7 to 3.85 CNY kWh^{-1} based on the economic investment at a global average level.

The payback times from the carbon emissions criterion

In terms of the payback times for carbon emissions (T_{em}), it could be determined by comparing the initial greenhouse gas emissions and avoided GHG emissions by replacing the electricity from the grid. Note that the carbon intensity of the grid in this study is the average intensity from different sources (e.g. PV, wind, coal, etc.) which is determined by the electricity composition and carbon emission intensity for each energy resource. We assume the initial emissions to be the same as that producing in China, and the decarbonization at each year can be calculated by equation (5). The carbon intensity of electricity from the global grid at an average level is shown in Supplementary Fig. 7, under three global electricity structure scenarios. T_{em} at the global scale is calculated to be 1.54 (STEPS), 1.55 (APS) and 1.56 (NZE) years by equation (9). Additionally, for a 1 MWp PV system at the global scale, based on the electricity structure from STEPS, APS, and NZE, the total decarbonization over a 30-year operational period is estimated to be 10,569 t, 8,320 t, and 5,338 t, respectively (Supplementary Fig. 5).

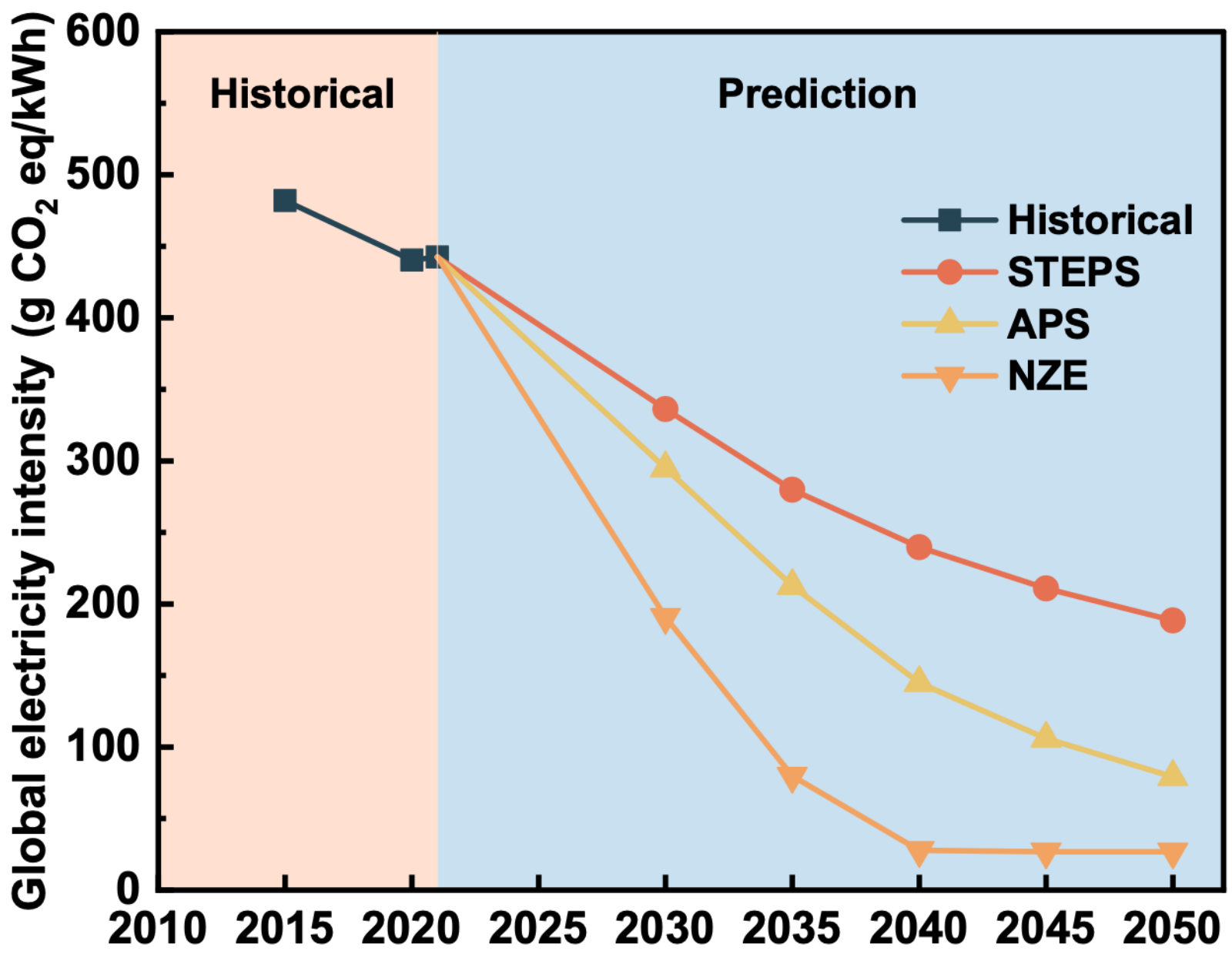

Supplementary Figure 7. The average carbon intensity of electricity from regional grid at the global scale.

Note: The carbon intensity of electricity from regional grid can be calculated by equation (6), and we consider 10 different electricity generation methods. Their carbon intensity mainly refers to the Intergovernmental Panel on Climate Change (IPCC) [24], and the rest which are fossil fuels with Carbon Capture, Utilization and Storage (CCUS) and others are assumed to be 10% of that without CCUS and zero gCO_2 eq kWh^{-1}**.** The electricity structure scenarios in global from 2030 to 2050 are obtained from the International Energy Agency (IEA) and its extended database [https://www.iea.org/reports/world-energy-outlook-2022].

The decarbonization contributions for PV industry at the global scale

In terms of the production of PV items in China, Supplementary Table 8 outline the proportions generated by the PV industry. Supplementary Note 6 delves into the accompanying sensitivity analysis for these findings.

Supplementary Table 8. The manufacturing ratios of photovoltaic (PV) products contributed by PV industry in China.

Product	2010	2015	2021	2027
Polysilicon	28.6%	48.2%	79.4%	72.6%
Wafers	78.3%	81.8%	96.8%	75.0%
Cells	57.9%	66.7%	85.2%	66.7%
PV modules	55.7%	64.7%	74.6%	62.5%
Average	55.1%	65.4%	84.0%	69.2%

Note: The manufacturing ratios of PV products contributed by PV industry in China is shown in Supplementary Table 8. We assume a linear progression, starting from 55.1% in 2010, reaching 65.4% in 2015 and 84.0% in 2021, and stabilizing at 69.2% since 2027 [26,29]. This is based on the projections from report by International Energy Agency (IEA), considering the impact of major policy incentives,

such as the US Inflation Reduction Act and India's Production Linked Incentive scheme (IEA, 2022) [30].

Supplementary Note 5. Strategic timing when considering industrial symbiosis' decarbonization contributions

We consider a 1 MWp PV system paired with a 200 kW / 400 kWh lithium battery energy storage system (BESS). We then analyze the potential effects of the integration between the energy storage and PV industries on China's PV manufacturing sector. The primary energy demands, economic investment and carbon emissions of the BESS are obtained from literature [3,31,32], and the rest part is assumed to be the same to the global average level in the NZE. We use the same criteria: energy consumption, economic costs, and carbon emissions. Under NZE, we employ equations (7)-(9) and the provided data to calculate the payback times from the three criteria. The results are illustrated in Supplementary Fig. 8, indicating payback times of 2.8, 4.2, and 1.8 years for the respective criterion. We then incorporate the energy storage systems and utilized coefficients to quantify the potential decarbonization contributions. Please refer to Supplementary Note 6 for more details and the sensitivity analysis linked to these findings.

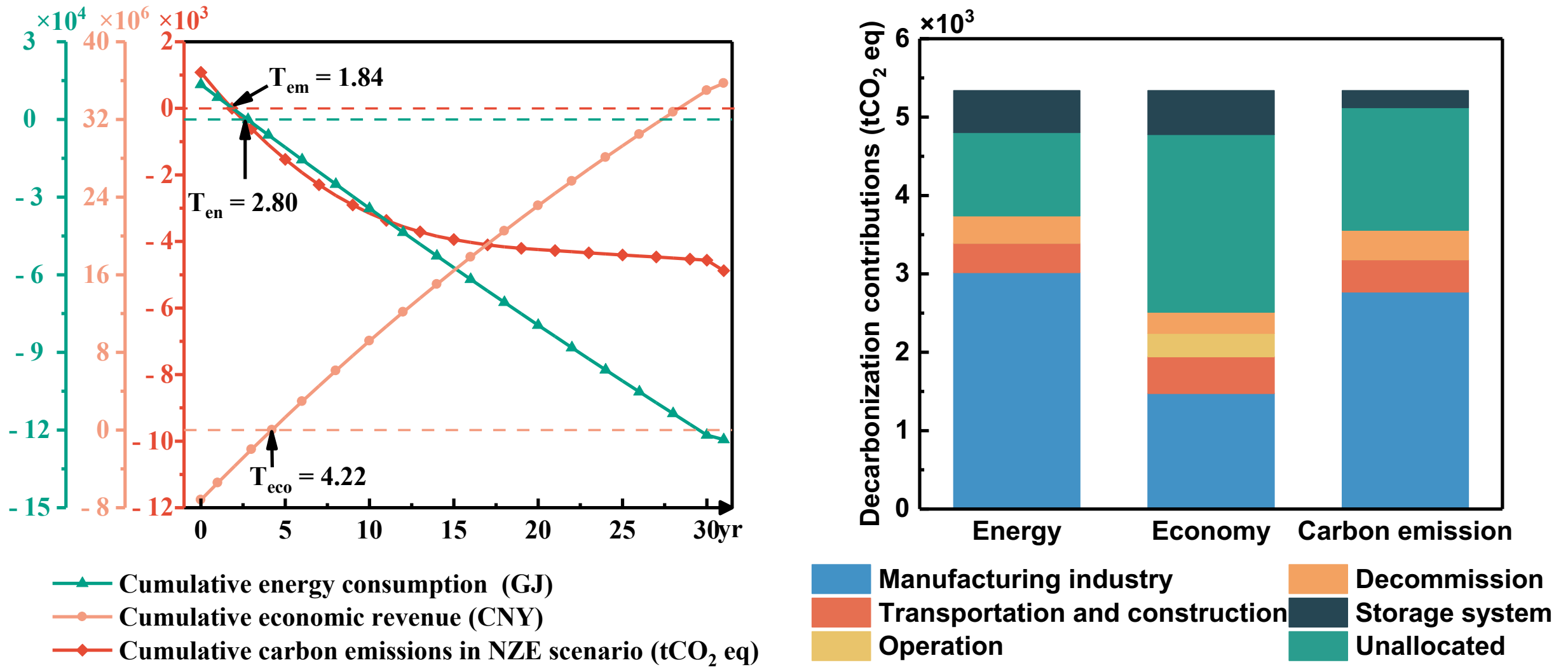

(a) The payback times of three dimensions **(b) The decarbonization of three dimensions**

Supplementary Figure 8. The payback times and decarbonization of three dimensions in global NZE electricity structure scenario, considering energy storage system. (a) The payback times from the criteria of energy, economy and carbon emissions for a 1 MWp photovoltaic (PV) system operating for 30 years at the global scale and the Net Zero Emissions by 2050 Scenario (NZE). **(b)** The distribution of decarbonization contributions over PV lifecycle in three criteria and scenarios for 1 MWp PV system at the global scale and the NZE scenarios.

Supplementary Note 6. The sensitivity and uncertainty analysis

We examine the carbon emissions and payback times by taking into account the average carbon intensity for electricity generation to the grid in China, solar radiation intensity, and a 30-year operational lifetime. This information is depicted in Fig. 2 and 3. To gain a comprehensive

understanding, we conduct various sensitivity analyses. Initially, we explore the impact of factors such as the grid's carbon intensity in different countries (Supplementary Fig. 9-10), the operational lifespan of the PV system (Supplementary Fig. 11), and solar radiation intensity (Supplementary Fig. 12). Furthermore, we delve into the economic aspects by analyzing payback times both in China and on a global scale. To do this, we scrutinize the effects of discount rates and electricity prices in China (Supplementary Fig. 4 and Supplementary Fig. 6).

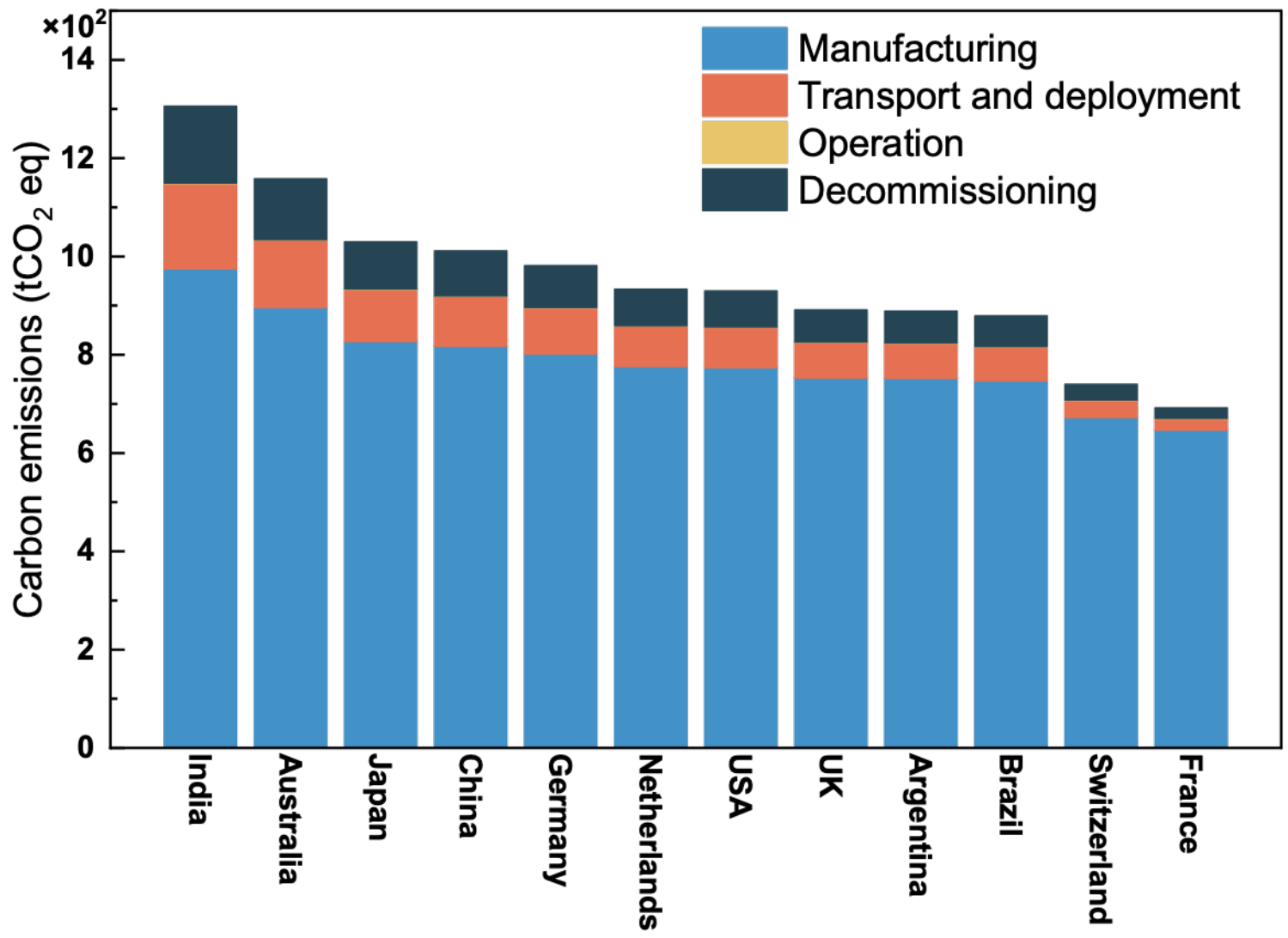

Supplementary Figure 9. The sensitivity analysis to total carbon emissions on the grid's carbon intensity of different countries.

Note: Assuming that the production process of the photovoltaic (PV) system varies in regions with different carbon intensities of electricity from the grid, we calculate the life-cycle carbon emissions for 1 MWp PV system considering different carbon intensity of the grid. The electricity structures of major countries are selected, with carbon intensities ranging from 0.106 to 1.112 $kgCO_2$ eq kWh^{-1}.

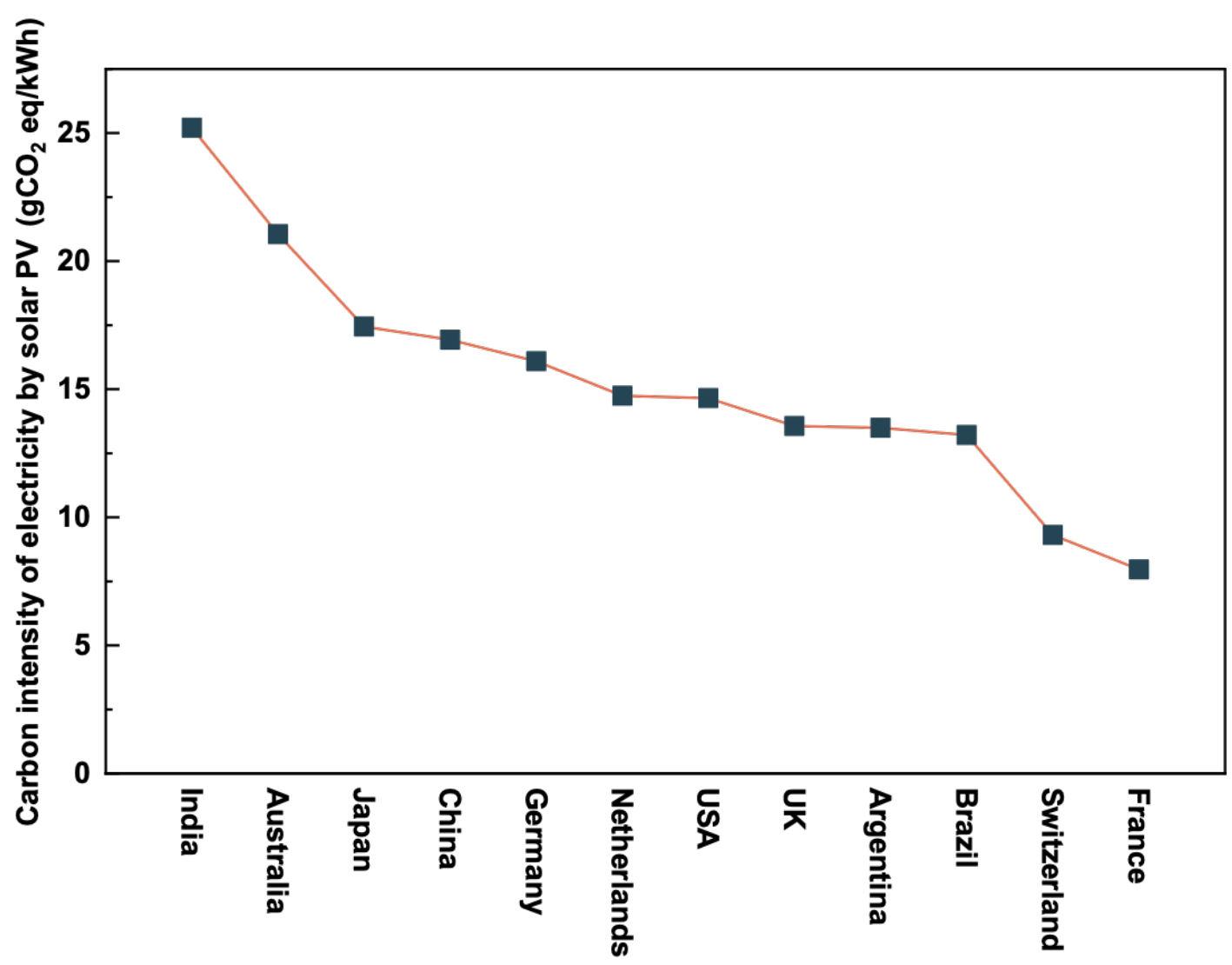

Supplementary Figure 10. The sensitivity analysis to the photovoltaic (PV) carbon intensity on the grid's carbon intensity of different countries.

Note: Based on the average solar radiation intensity, performance and manufacturing level at the year

of 2022 in China, with global tilted irradiance of 1756 kWh m^{-2} per year, we calculate the carbon intensity of electricity from PV by considering the carbon intensity of grid in different regions.

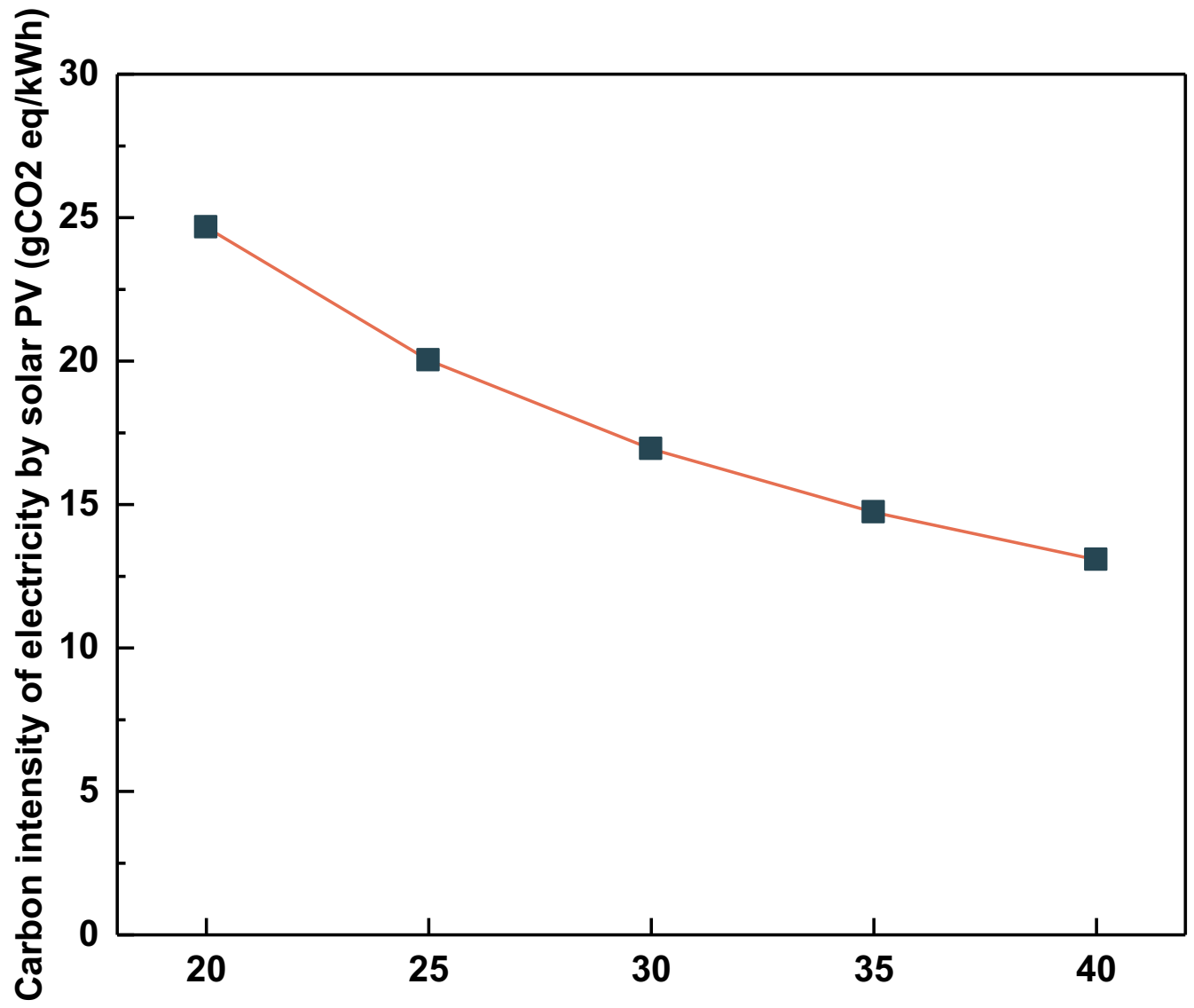

Supplementary Figure 11. The sensitivity analysis to the PV carbon intensity on photovoltaic (PV) system's lifetime.

Note: Based on the average solar radiation intensity in China and the production levels in 2022, we calculate the carbon intensity of electricity from PV by considering the lifetime from 20 to 40 years.

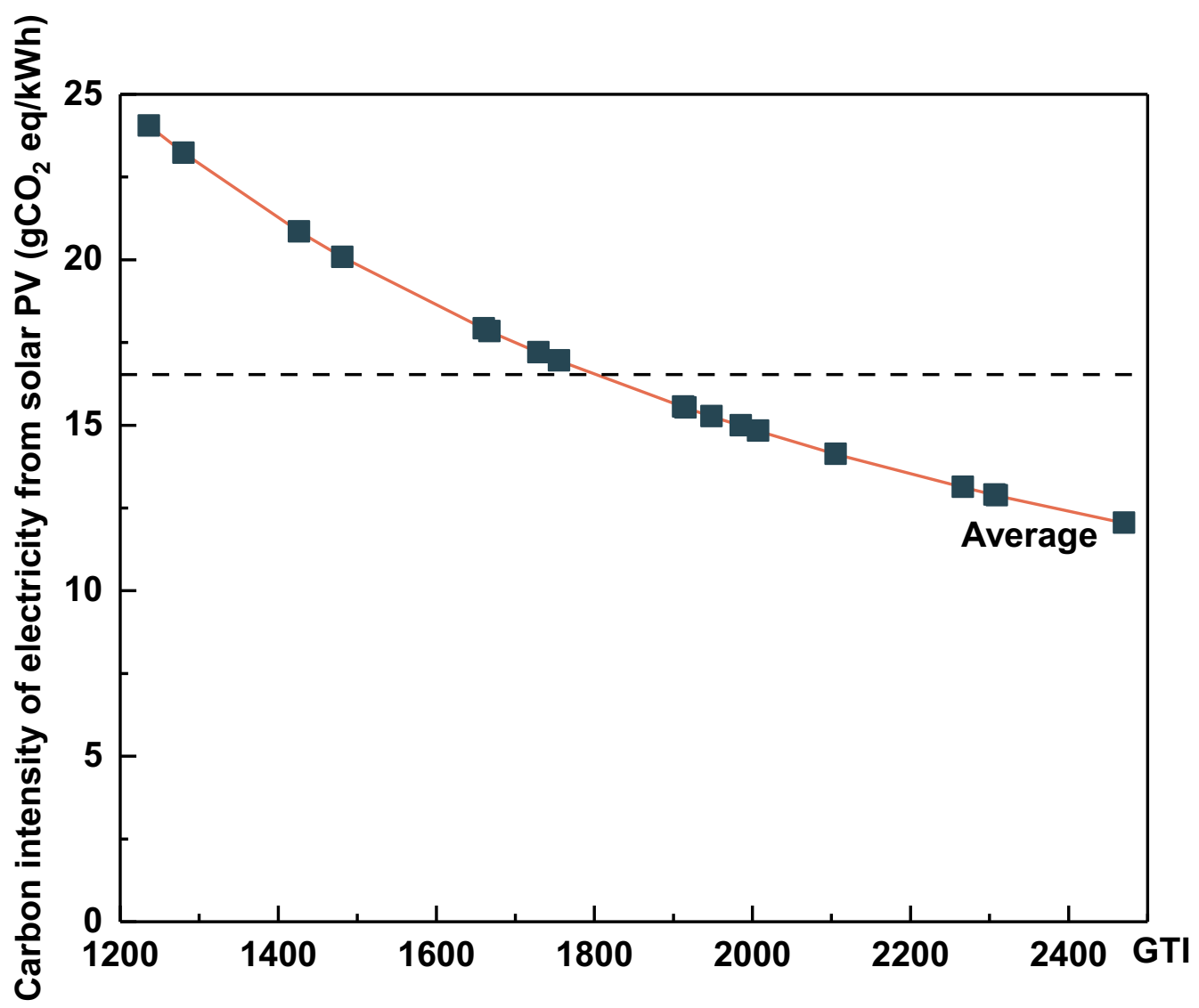

Supplementary Figure 12. The sensitivity analysis to the photovoltaic (PV) carbon intensity on solar radiation intensity.

Note: Based on the production levels in 2022, we calculate the carbon intensity of electricity from PV by considering the different solar radiation intensity. Here, we select the solar radiation intensity of 18 countries with GTI of 1237 to 2471 kWh m^{-2} per year.

We investigate the distribution of decarbonization contributions. Our focus is on how different electricity scenarios in China, global scenarios, and scenarios involving energy storage systems affect the potential decarbonization contributions by the PV manufacturers during the lifespan of PV

systems. Subsequently, by applying these allocation coefficients, we visualize the decarbonization amounts at various stages within the PV industry for China (Supplementary Fig. 13-15), the global (Supplementary Fig. 16-18), and scenarios involving energy storage system (Supplementary Fig. 19-21).

Given the uncertainties surrounding the future placement of PV systems, we evaluate the carbon intensity of PV electricity and decarbonization contributions within the PV industry. We conduct this by considering different solar irradiation levels across regions in China. These outcomes are presented in Supplementary Table 9 and 10. On a global scale, we employ the average solar radiation intensity from various countries, selecting 18 representative nations as shown in Supplementary Table 6.

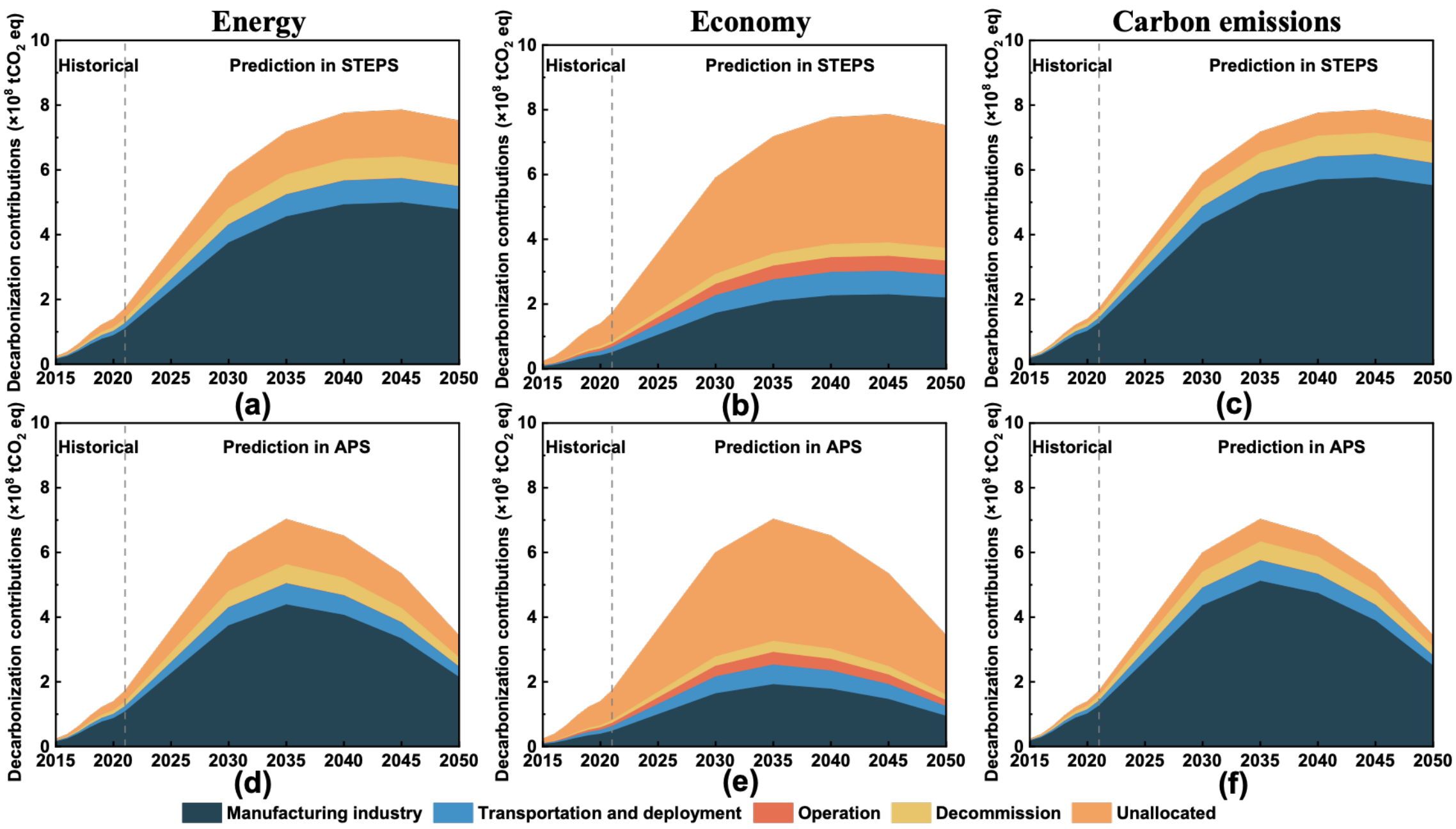

Supplementary Figure 13. The decarbonization contributions of photovoltaic (PV) system at each stage in China with lifetime of 20 years. (a-c) The decarbonization contributions allocated under the Stated Policies Scenario (STEPS) according to the energy, economic, and carbon emissions criteria, respectively. **(d-f)** The decarbonization contributions allocated under the Announced Pledges Scenario (APS) according to the energy, economic, and carbon emissions criteria, respectively.

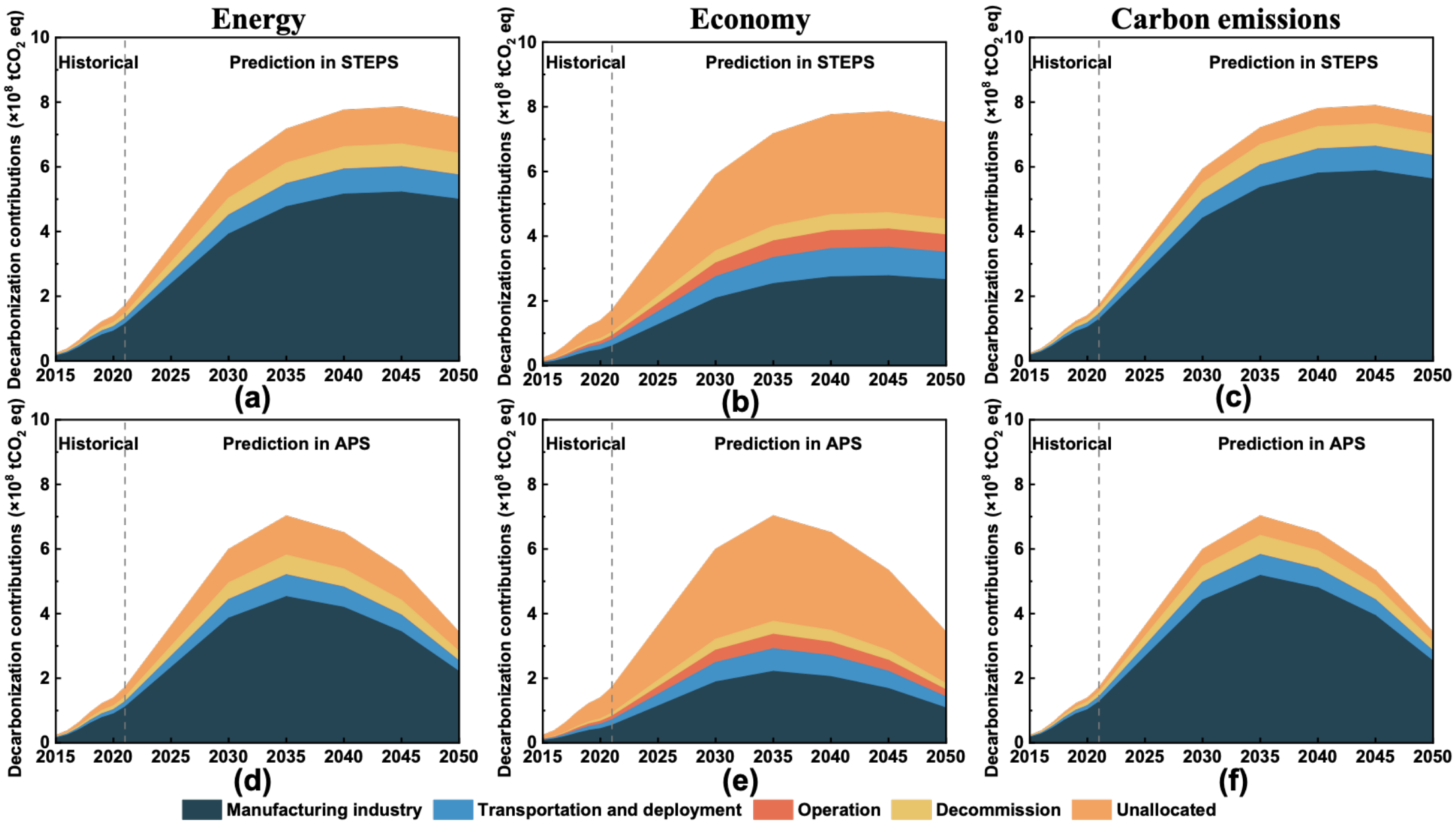

Supplementary Figure 14. The decarbonization contributions of photovoltaic (PV) system at each stage in China with lifetime of 30 years. (a-c) The decarbonization contributions allocated under the Stated Policies Scenario (STEPS) according to the energy, economic, and carbon emissions criteria, respectively. **(d-f)** The decarbonization contributions allocated under the Announced Pledges Scenario (APS) according to the energy, economic, and carbon emissions criteria, respectively.

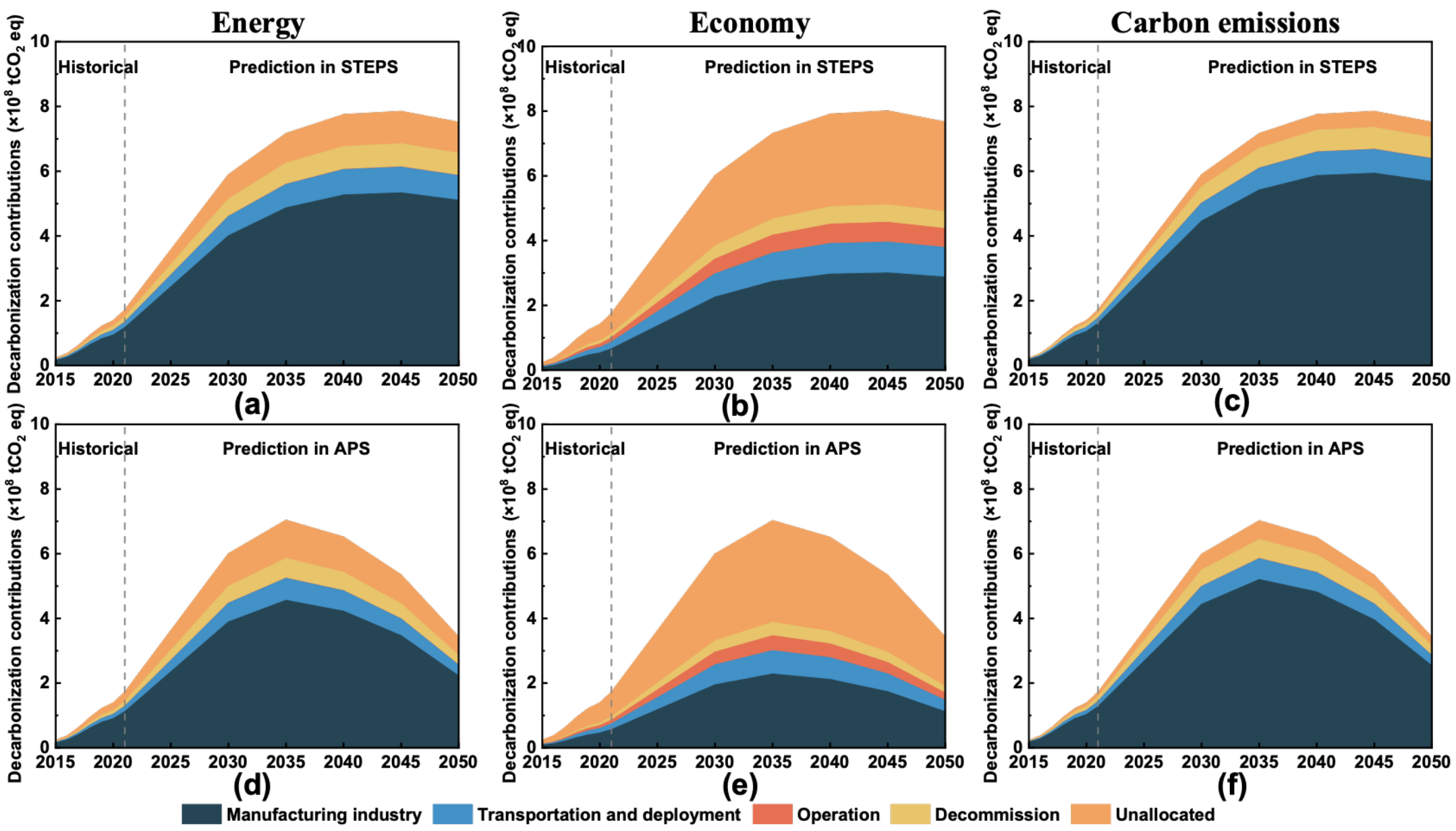

Supplementary Figure 15. The decarbonization contributions of photovoltaic (PV) system at each stage in China with lifetime of 40 years. (a-c) The decarbonization contributions allocated under the Stated Policies Scenario (STEPS) according to the energy, economic, and carbon emissions criteria, respectively. **(d-f)** The decarbonization contributions allocated under the Announced Pledges Scenario (APS) according to the energy, economic, and carbon emissions criteria, respectively.

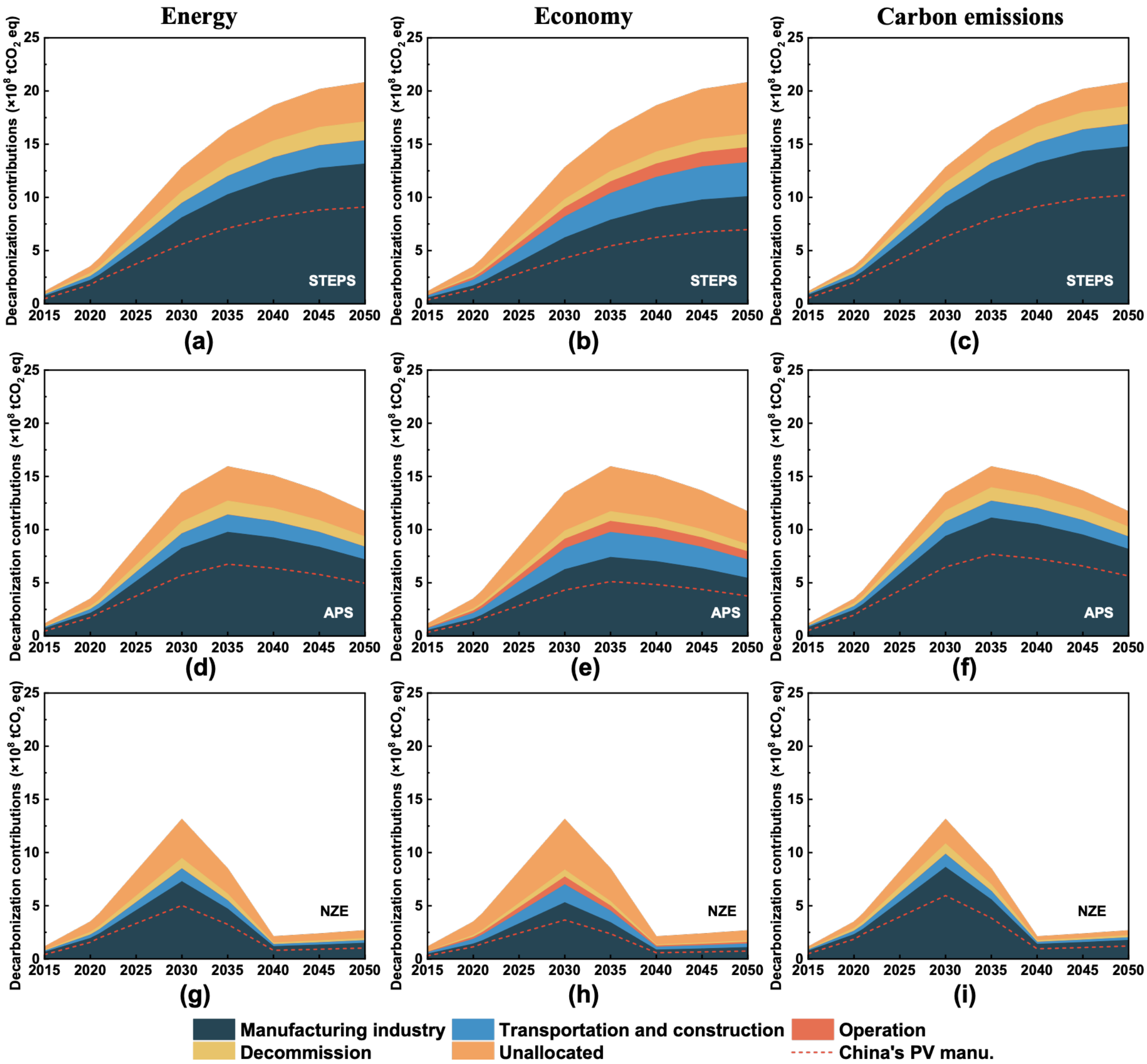

Supplementary Figure 16. The decarbonization contributions of photovoltaic (PV) system at each stage in global with lifetime of 20 years. (a-c) The decarbonization contributions allocated under the Stated Policies Scenario (STEPS) according to the energy, economic, and carbon emissions criteria, respectively. **(d-f)** The decarbonization contributions allocated under the Announced Pledges Scenario (APS) according to the energy, economic, and carbon emissions criteria, respectively. **(g-i)** The decarbonization contributions allocated under the Net Zero Emissions by 2050 Scenario (NZE) according to the energy, economic, and carbon emissions criteria, respectively.

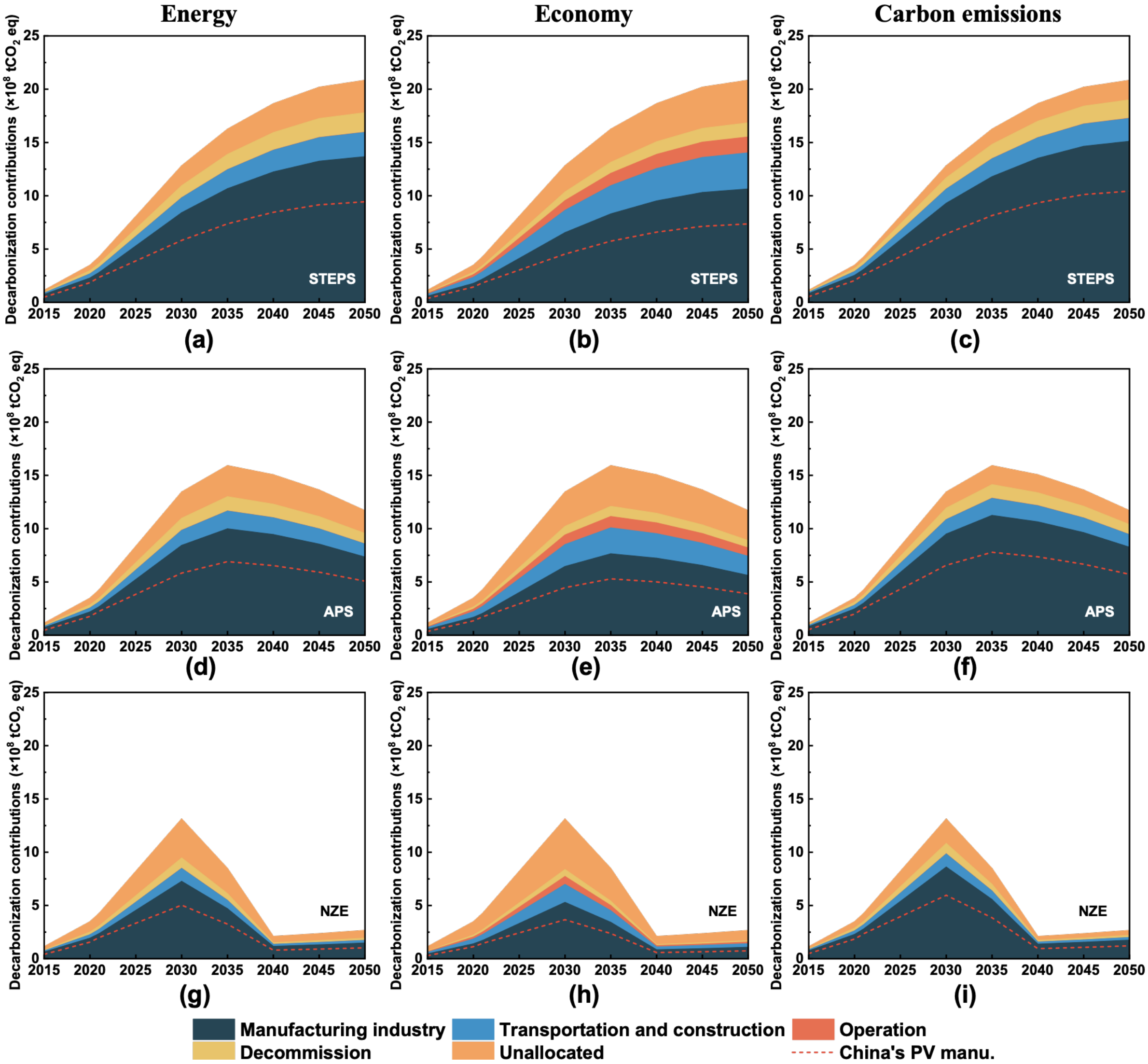

Supplementary Figure 17. The decarbonization contributions of photovoltaic (PV) system at each stage in global with lifetime of 30 years. (a-c) The decarbonization contributions allocated under the Stated Policies Scenario (STEPS) according to the energy, economic, and carbon emissions criteria, respectively. **(d-f)** The decarbonization contributions allocated under the Announced Pledges Scenario (APS) according to the energy, economic, and carbon emissions criteria, respectively. **(g-i)** The decarbonization contributions allocated under the Net Zero Emissions by 2050 Scenario (NZE) according to the energy, economic, and carbon emissions criteria, respectively.

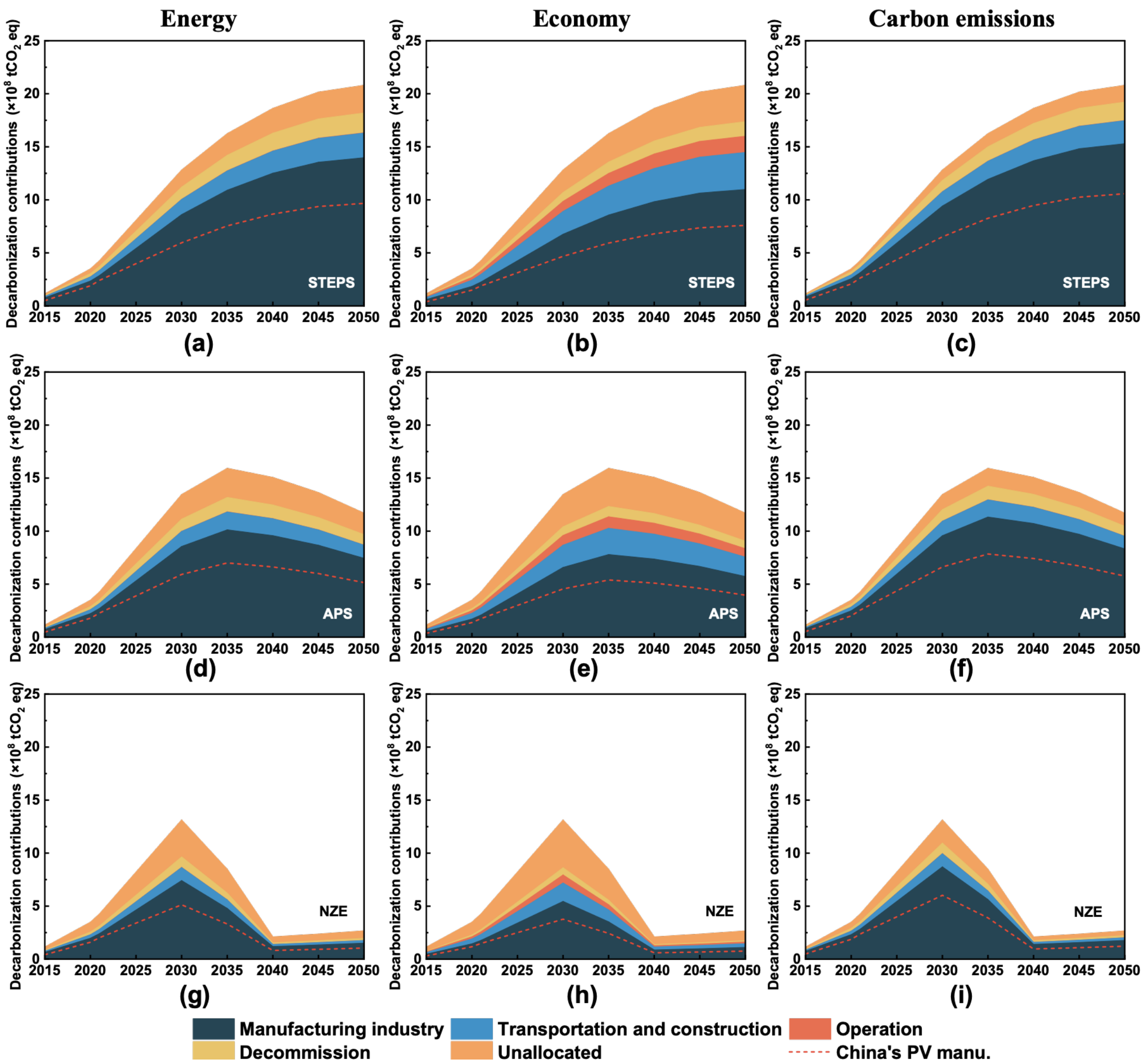

Supplementary Figure 18. The decarbonization contributions of photovoltaic (PV) system at each stage in global with lifetime of 40 years. (a-c) The decarbonization contributions allocated under the Stated Policies Scenario (STEPS) according to the energy, economic, and carbon emissions criteria, respectively. **(d-f)** The decarbonization contributions allocated under the Announced Pledges Scenario (APS) according to the energy, economic, and carbon emissions criteria, respectively. **(g-i)** The decarbonization contributions allocated under the Net Zero Emissions by 2050 Scenario (NZE) according to the energy, economic, and carbon emissions criteria, respectively.

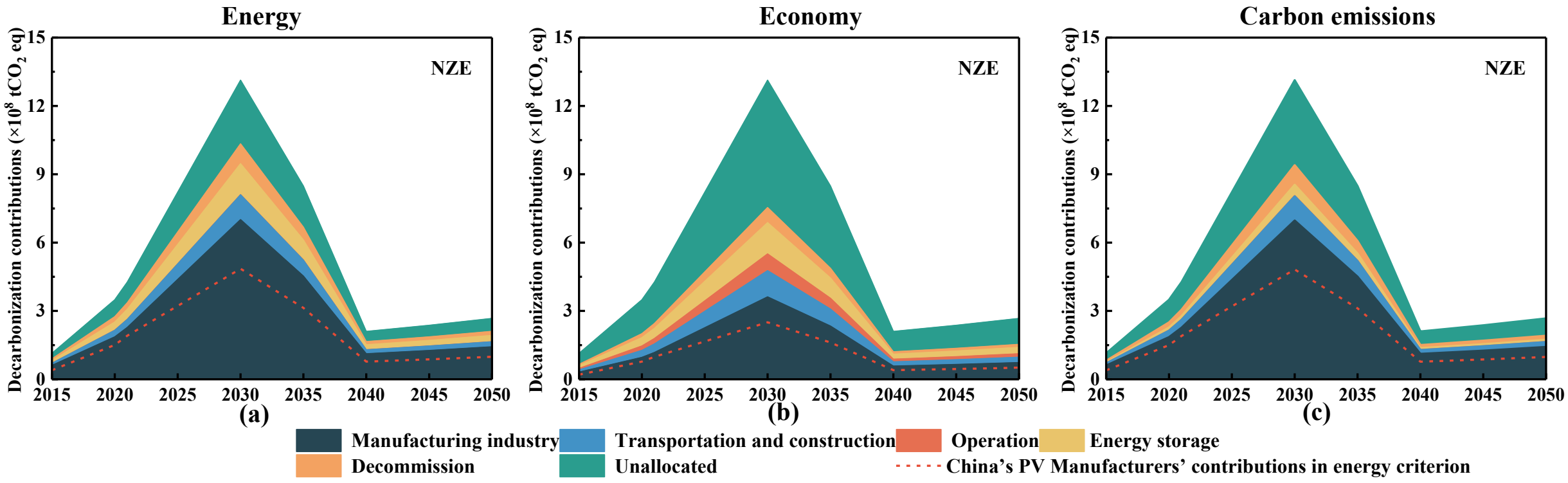

Supplementary Figure 19. The decarbonization contributions of photovoltaic (PV) system at each stage under the Net Zero Emissions by 2050 Scenario (NZE) with the lifetime of 20 years, considering energy storage industry. (a-c) The decarbonization contributions allocated according to the energy, economic, and carbon emissions criteria, respectively.

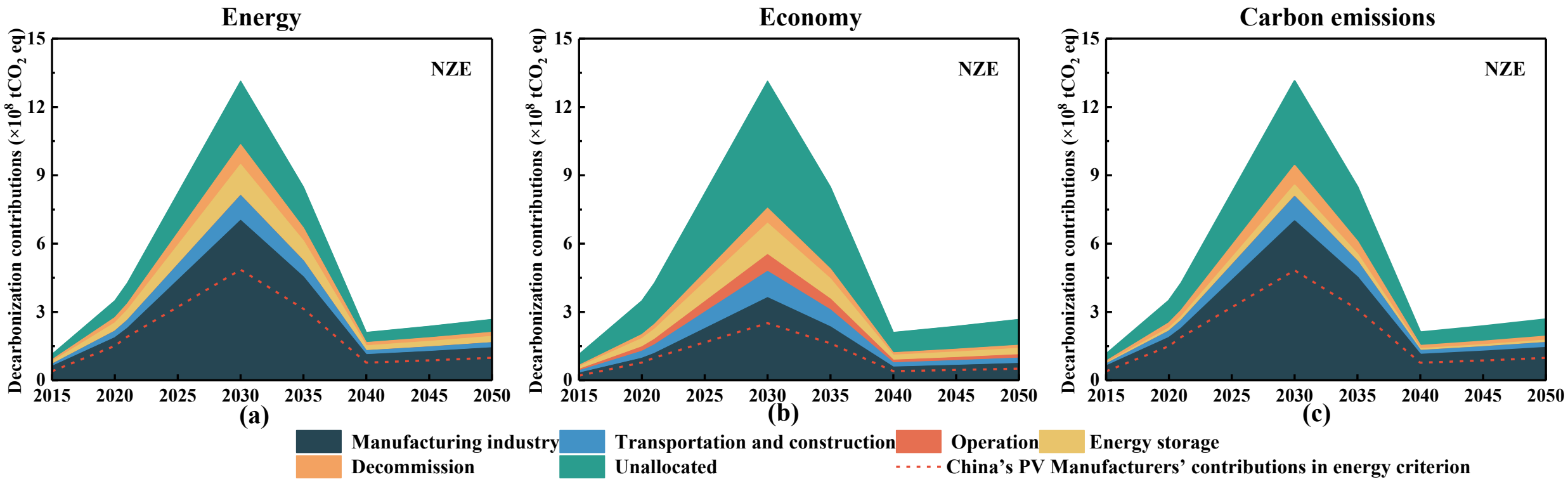

Supplementary Figure 20. The decarbonization contributions of photovoltaic (PV) system at each stage under the Net Zero Emissions by 2050 Scenario (NZE) with the lifetime of 30 years, considering energy storage industry. (a-c) The decarbonization contributions allocated according to the energy, economic, and carbon emissions criteria, respectively.

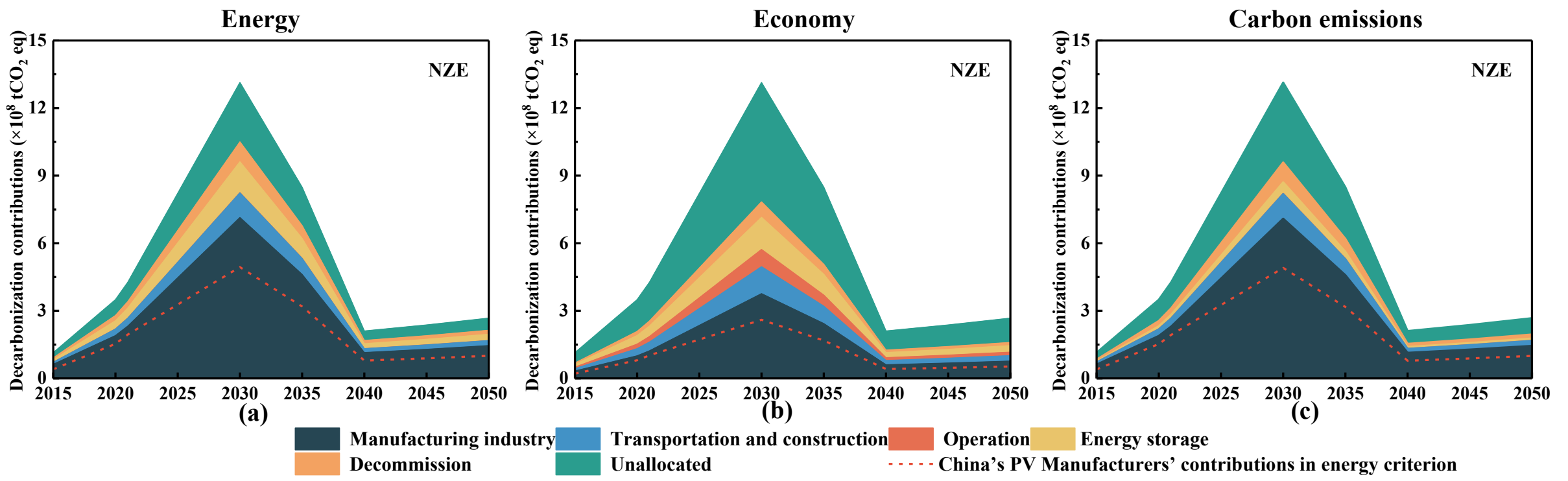

Supplementary Figure 21. The decarbonization contributions of photovoltaic (PV) system at each stage under the Net Zero Emissions by 2050 Scenario (NZE) with the lifetime of 40 years, considering energy storage industry. (a-c) The decarbonization contributions allocated according to the energy, economic, and carbon emissions criteria, respectively.

Supplementary Table 9. The uncertainty analysis to the decarbonization amounts considering the solar radiation intensity of different regions in China in 2015-2021 ($\times 10^8$ tCO_2).

Items	Carbon intensity of PV (gCO_2 kWh^{-1})	2015	2016	2017	2018	2019	2020	2021
Average	16.94	0.230	0.371	0.628	0.944	1.213	1.387	1.717
Min	30.18	0.225	0.362	0.613	0.921	1.182	1.352	1.674
Max	11.76	0.232	0.375	0.634	0.953	1.225	1.400	1.734
North	15.24	0.231	0.372	0.630	0.947	1.217	1.391	1.723
Northeast	16.84	0.230	0.371	0.628	0.944	1.213	1.387	1.717
East	20.60	0.229	0.369	0.624	0.938	1.205	1.377	1.705
Central	22.77	0.228	0.367	0.622	0.934	1.200	1.372	1.698
South	21.91	0.228	0.368	0.623	0.936	1.202	1.374	1.701
Southwest	16.42	0.230	0.371	0.629	0.945	1.214	1.388	1.719
Northwest	16.13	0.230	0.372	0.629	0.946	1.215	1.389	1.720

Supplementary Table 10. The uncertainty analysis to the decarbonization amounts considering the solar radiation intensity of different regions of China in 2030-2050 ($\times 10^8$ tCO_2).

Items	Stated Policies Scenario (STEPS)					Announced Pledges Scenario (APS)				
	2030	2035	2040	2045	2050	2030	2035	2040	2045	2050
Average	5.894	7.166	7.753	7.851	7.514	5.987	7.023	6.505	5.336	3.422
Min	5.699	6.876	7.373	7.392	6.996	5.777	6.691	6.027	4.724	2.701
Max	5.970	7.280	7.902	8.030	7.717	6.069	7.153	6.693	5.576	3.704
North	5.919	7.204	7.802	7.909	7.580	6.014	7.065	6.567	5.415	3.514
Northeast	5.895	7.169	7.756	7.854	7.518	5.989	7.025	6.509	5.341	3.427
East	5.840	7.086	7.648	7.724	7.371	5.929	6.931	6.373	5.167	3.222
Central	5.808	7.039	7.586	7.649	7.286	5.895	6.877	6.295	5.067	3.105
South	5.821	7.057	7.611	7.679	7.320	5.908	6.898	6.326	5.106	3.151
Southwest	5.901	7.178	7.768	7.868	7.534	5.995	7.036	6.524	5.360	3.450
Northwest	5.906	7.184	7.777	7.879	7.546	6.000	7.043	6.535	5.374	3.466

Note: The uncertainty for decarbonization amounts of Chinese photovoltaic (PV) industry comes from the variation of solar radiation intensity in different regions of China. According to Global Solar Atlas, global tilted irradiation (GTI) at optimum angle in different regions of China ranges from 986 to 2529 kWh m^{-2} per year, with an area-weighted average of 1756 kWh m^{-2} per year [25]. This range leads to variations in the carbon intensity of electricity from solar PV, with values ranging from 11.76 to 30.18 gCO_2 eq kWh^{-1} and an area-weighted average of 16.94 gCO_2 eq kWh^{-1} in China. Supplementary Table 9-10 depict the decarbonization contributions of Chinese PV industry under solar radiation in different regions from 2015-2050.

Supplementary References

1 LONGI. *Product specifications for photovoltaic modules*, <https://www.longi.com/cn/download/> (2022).

2 Trinasolar. *Product specifications for photovoltaic modules*, <https://www.trinasolar.com/cn/product/utility> (2022).

3 CPIA. China's Photovoltaic Industry Development Roadmap (2018-2023). *China Photovoltaic Industry Association (CPIA*), (2023).

4 Department of Industry and Information Technology of Yunnan Province. *Notification on the Regulatory Management of Resource and Energy Consumption in 2017 for 12 Industries, including Yellow Phosphorus, by the Industrial and Informationization Commission of Yunnan Province*, <http://gxt.yn.gov.cn/zwgk/fdzdgknr/jlm2ynsgxw/tzgggszdhzjcsx2ynsgxw/tbsx2ynsgxw/content_14420> (2019).

5 R. Frischknecht, P. S., L. Krebs, M. de Wild-Scholten, P. Sinha, V. Fthenakis, H. C. Kim, M. Raugei, M. Stucki Life Cycle Inventories and Life Cycle Assessment of Photovoltaic Systems. *International Energy Agency (IEA)*, (2020).

6 National iron ore and direct reduced iron standardization technical committee. Energy consumption quota standard of mining and dressing for mining enterprises - Part 1: Iron ore mining. (2014).

7 Yao, L. *Life cycle assessment of crystalline silicon modules in China*, Shanghai Jiao Tong University, (Master dissertation, 2015).

8 *Specification conditions for photovoltaic manufacturing industry (the version of 2018, 2020 and 2021)*, <https://www.miit.gov.cn/zwgk/zcwj/wjfb/gg/art/2021/art_24b585fce0f64c98be8efe14e0446458.html> (2021).

9 The trend of diamond wire thinning continues, and the substitution of tungsten wire busbars is timely. *Citic Securities*, (2022).

10 Yu, Z., Ma, W., Xie, K., Lv, G., Chen, Z., Wu, J. *et al*. Life cycle assessment of grid-connected power generation from metallurgical route multi-crystalline silicon photovoltaic system in China. *Appl. Energy* **185**, 68–81 (2017). https://doi.org/10.1016/j.apenergy.2016.10.051

11 Wang, Z., Chen, Z., Wennersten, R. & Sun, Q. *in 2022 International Conference on Power Energy Systems and Applications (ICoPESA).* 553–564 (IEEE).

12 Fthenakis, V. M. & Kim, H. C. Photovoltaics: Life-cycle analyses. *Sol. Energy* **85**, 1609–1628 (2011). https://doi.org/10.1016/j.solener.2009.10.002

13 Jia, L. *Environmental effects investigating for the grid-connected BAPV*, Tianjin University, (Ph.D dissertation, 2012).

14 Hou, G., Sun, H., Jiang, Z., Pan, Z., Wang, Y., Zhang, X. *et al*. Life cycle assessment of grid-connected photovoltaic power generation from crystalline silicon solar modules in China. *Appl. Energy* **164**, 882–890 (2016). https://doi.org/10.1016/j.apenergy.2015.11.023

15 Mason, J., Fthenakis, V., Hansen, T. & Kim, H. Energy payback and life-cycle CO_2 emissions of the BOS in an optimized 3.5 MW PV installation. *Prog. Photovoltaics Res. Appl.* **14**, 179–190 (2006). https://doi.org:https://doi.org/10.1002/pip.652

16 Ansanelli, G., Fiorentino, G., Tammaro, M. & Zucaro, A. A Life Cycle Assessment of a recovery process from End-of-Life Photovoltaic Panels. *Appl. Energy* **290**, 116727 (2021). https://doi.org/10.1016/j.apenergy.2021.116727

17 GaBi Databases, <https://ghgprotocol.org/gabi-databases> (2024).

18 IPCC. Climate Change 2022: Impacts, Adaptation, and Vulnerability. *Intergovernmental Panel on Climate Change (IPCC)*, (2022).

19 The Annual Development Report of Chinese Electricity Industry 2016-2022. *China Electricity Council*, Beijing (2023).

20 IEA. World Energy Outlook 2022. *International Energy Agency (IEA),* Paris (2022).

21 Peng, J., Lu, L. & Yang, H. Review on life cycle assessment of energy payback and greenhouse gas emission of solar photovoltaic systems. *Renewable Sustainable Energy Rev.* **19**, 255–274 (2013). https://doi.org/10.1016/j.rser.2012.11.035

22 Mohd Nordin, A. H., Sulaiman, S. I., Shaari, S. & Mustapa, R. F. Energy and environmental impacts of a 37.57 MW dc ground-mounted large-scale photovoltaic system in Malaysia: A life-cycle approach. *J. Cleaner Prod.* **335**, 130326 (2022). https://doi.org/10.1016/j.jclepro.2021.130326

23 Jia, X., Lv, F., Li, P. & Wang, W. Life-cycle assessment of p-type multi-Si back surface field (BSF) solar module in China of 2019. *Sol. Energy* **196**, 207–216 (2020). https://doi.org/10.1016/j.solener.2019.12.018

24 Schlömer S., T. B., L. Fulton, E. Hertwich, A. McKinnon, D. Perczyk, J. Roy, R. Schaeffer, R. Sims, P. Smith, and R. Wiser. Technology-specific cost and performance parameters. *Intergovernmental Panel on Climate Change (IPCC)*, Cambridge University Press (2014).

25 Global Solar Atlas. Global Solar Atlas 2.0 : Validation Report. *Global Solar Atlas*, (2020).

26 IEA. Solar PV Global Supply Chains Analysis. *International Energy Agency (IEA)*, Paris (2022).

27 IRENA. Renewable Power Generation Costs in 2021. *International Renewable Energy Agency (IRENA*), (2022).

28 *Electricity prices, September 2022* <https://www.globalpetrolprices.com/electricity_prices/> (2022).

29 CPIA. China's Photovoltaic Industry Development Roadmap (2018-2022). *China Photovoltaic Industry Association (CPIA)*, (2023).

30 IEA. Will new PV manufacturing policies in the United States, India and the European Union create global PV supply diversification? *International Energy Agency (IEA)*, Paris (2022).

31 Quan, J., Zhao, S., Song, D., Wang, T., He, W. & Li, G. Comparative life cycle assessment of LFP and NCM batteries including the secondary use and different recycling technologies. *Sci. Total Environ.* **819**, 153105 (2022). https://doi.org/10.1016/j.scitotenv.2022.153105

32 Sun, X., Luo, X., Zhang, Z., Meng, F. & Yang, J. Life cycle assessment of lithium nickel cobalt manganese oxide (NCM) batteries for electric passenger vehicles. *J. Cleaner Prod.* **273**, 123006 (2020). https://doi.org/10.1016/j.jclepro.2020.123006
