# Supplementary material for: Strategic timing for the decarbonization contributions by China’s photovoltaic manufacturers in global energy transition
Source: Nat Commun. 2026 Aug 6;17:9465. doi: 10.1038/s41467-026-76424-4 (PMC13542174; doi:10.1038/s41467-026-76424-4)
Supplement: Supplementary file 3 [file 41467_2026_76424_MOESM3_ESM.pdf]

nature portfolio

Peer Review File

Strategic Timing for the Decarbonization Contributions by China's Photovoltaic Manufacturers in Global Energy Transition

Corresponding Author: Professor Kai Fang

This file contains all reviewer reports in order by version, followed by all author rebuttals in order by version.

Attachments originally included by the reviewers as part of their assessment can be found at the end of this file.

Version 0:

Reviewer comments:

Reviewer #1

(Remarks to the Author)
Please see the attachment.

Reviewer #2

(Remarks to the Author)
The paper presents an interesting aspect of the responsibilities of carbon emissions not only on the side of the manufacturer but also on the side of the power enterprises to achieve decarbonization of the electricity grids. Hence, it is useful and important for scientist and policymakers alike, warranting for publication provided that the following remarks are taken into account.

Page 3, line 100
We find that in China the PV manufacturing stage account for 77.8% of energy consumption, 58.7% of economic costs, and 80.7% of carbon emissions (Fig. 1).

Please give an indication how the values presented here for the energy consumption, the economic costs, and the carbon emissions have been determined. The values fall out of the sky.

Page 3, line 101
The decarbonization contributions are achieved by comparing the carbon intensity of PV-generated electricity (16.94 gCO2 eq/kWh using the latest industrial production data, see Methods section for more details) and the average carbon intensity for various technologies supplying the grid (543 gCO2 eq/kWh) over 30 years.

Please give more details here. Which technologies are included in the average carbon intensity supplying the grid? Which grid region was considered for the average carbon intensity? How is the decarbonization contributions determined? In general, it is difficult to understand it when there is no guideline.

Page 3, line 103
The carbon intensity of PV-generated electricity has considered both emissions and emissions reduction through recycling in the decommissioning stage.

Is recycling of the module or recycling of the photovoltaic power plant meant here?

Page 3, line 106
The payback times on energy (T_{en}= 2.73 years), economy (T_{eco}= 8.16 years) and carbon emissions (T_{em}=1.32 years) criteria are used to determine the assigned decarbonization contributions, ...

Where do these values come from? How were the values determined?

Please give an indication how the values presented here for the energy, economy and carbon emissions payback times were determined. In general, it is difficult to understand it when there is no guideline.

Page 4, line 116
The determination of carbon emissions per unit capacity PV system, associated data sources, and the determination of payback times from different criteria are provided in the Supplementary Notes 1–3.

As the determination of carbon emissions and the determination of payback times is considered very important for the motivation of the paper, it should be place in the paper and not in the supplementary only.

Please find a good balance between presenting important and necessary information concerning the determination of carbon emissions and the determination of payback times in the paper and in the supplementary.

Page 5, line 128
The potential decarbonization contributions and associated strategic timing for PV manufacturing industry in China from 2015 to 2050 for STEPS and APS are shown in Fig. 3.

Please define “strategic timing”? What do you mean by that term?

Page 5, line 131
For STEPS, the strategic timing, referring to the maximum potential decarbonization contributions for adopting PV, are predicted to be 2045 to achieve an annual decarbonization of 7.85×108 tCO2 eq, while the PV manufacturing industry’s contributions are 2.77×108–5.88×108 tCO2 eq (percentage contributions: 35.4%–74.9%) following the three criteria.

What do you mean by “adopting PV”? What are the impacts on decarbonization contributions when PV is adopted before 2045 and after 2045? Is the impact less? To which extent are they less?

Page 8, line 201
On a global scale, to achieve net-zero emissions for 201 power sector by 2050, China’s PV manufacturers are anticipated to contribute 46.2%, and this figure slightly decreases to 37.6% without consideration of energy storage solutions.

What do you mean by energy storage solutions? How are they considered in the decarbonization contributions?

Page 8, line 213
Prior to this strategic timing, we anticipate a rising trajectory in the potential decarbonization contributions of the PV manufacturing industry primarily driven by the expanding deployment of PV systems, as the carbon intensity of PV-generated electricity is substantially lower than that of the grid electricity.

What do you mean by the statement “Prior to this strategic timing, we anticipate a rising trajectory in the potential decarbonization contributions of the PV manufacturing industry”? In which sense do you use trajectory here? Could you be more precise?

Reviewer #3

(Remarks to the Author)
The paper provides a comprehensive analysis of the decarbonisation contributions of PV systems manufactured in China. In general, it addresses the timely and critical need to understand the environmental impact of renewable energy sources in the context of the energy transition, and specifically it deals with the important topic of correctly attributing carbon emissions (or emission reduction) to the different stages of value chains.

The intuition is solid, the research paper is concise, and the work is well documented. The supplementary material provides comprehensive input for the reader.

Still, we see three major issues in the paper.

The first one deals with the concepts of decarbonisation contributions and strategic timing. Concerning the first one, clear

formulas are provided but throughout the text the reader cannot fully grasp to what the percentages refer. This should be better described. Concerning strategic timing, in the abstract we read that it is "a window of opportunity to accelerate the energy transition ambition to net-zero emissions" (lines 33-34); in the text, we read that strategic timing "refers to the maximum potential decarbonization contributions for adopting PV" (line 132). Practically, strategic timing is identified as one specific year (e.g. in line 135 it "occurs to be 2035"), but the reader does not get a clear understanding of what it ultimately is conceptually. The general concept is clear (the earlier one installs PV panels, the better it is because currently the energy system is more carbon-intensive), but what does this imply? Eventually, what is the year 2035 in the example before? What implications does it have on the actual PV deployment?

Another major issue is that mining seems to be neglected in the analysis. This is a major source of carbon emissions, so it should be considered in such a comprehensive work.

Third, the text needs to be revised from a language point of view. Apart from some normal typos (see at the end for some suggestions), it is often the case that sentences are not fully comprehensible and/or seem to have missing parts. We report a few examples in the following:
• Sentence in lines 49-50: authors likely refer to PV in this sentence, but good to specify (same in lines 146-147)
• Sentence in lines 83-85: maybe some brackets would help, e.g. "energy (characterized by the energy consumption), economy (reflected by the economic investments), and carbon emissions"
• Sentence in lines 91-93: "describes to achieve" is not very clear
• Sentence in lines 93-95: maybe "determined" should be "determine"?
• The sentence in line 100-101 should be contextualised
• The sentence in lines 109-112 does not seem very clear: at least one word is likely missing
• Line 299: "Carbon intensity of per unit electricity" is not very clear

Some additional minor points include:

• The paper often refers to the Sustainable Development Goals: these should be a bit better presented / put into context
• Line 30 (but often in other parts). We are not fully sure whether one decimal is needed in these percentages, but surely three decimals are not needed for the payback times e.g. in lines 160-162 of the Supplementary Material
• Is it 78% (line 52) or 80% (line 146)?
• Line 220: "desirable" is not a great word here; of course, PV's marginal effect decreases with time as the energy system becomes less and less carbon intensive, but it would still be desirable, at the end of the day
• Line 348: the reference discount rate is reported to be 0.1%, while in the Supplementary Material it seems to be 1% (see e.g. line 103 – which by the way is more reasonable than 0.1%), ranging from 0.1% to 5% in the sensitivity analysis. Please check.
• Line 375: from the references, we see that authors adopted the IEA's 2022 edition of the WEO. That is fine, but it is not the latest now, so they should write "2022" and not "latest" here
• Line 385: a reference to the Gabi database is reported in the Supplementary Material, but it is missing here, so it should be added
• Line 396: 2014 is quite far in the past now…
• Supplementary Figures 10-11-12: it is never nice if y axes start from a level different than zero, because then the proportion cannot be well captured

Finally, some typos / minor language suggestions:

• Line 29: "contributions would contribute" – maybe a different formulation can be found
• Line 92: repetition of "that"
• Line 99: "manufactures" should be "manufacturers"
• Line 100: "account" should be "accounts"
• Line 149: "higher" should be "a higher", or even better "a greater"
• Line 191: "remains to be" should be "remains"
• Line 211: "the three" should be "three"
• Line 96 SM: "differ" should likely be "difference"
• Line 380 SM: space before the comma

Reviewer #4

(Remarks to the Author)
I co-reviewed this manuscript with one of the reviewers who provided the listed reports. This is part of the Nature Communications initiative to facilitate training in peer review and to provide appropriate recognition for Early Career Researchers who co-review manuscripts.

Version 1:

Reviewer comments:

Reviewer #1

Please see the attachment.

Reviewer #2

(Remarks to the Author)
The paper has been improved according to the reviewers' comments and has gained a lot in clarity. It, therefore, warrants for publication.

Reviewer #3

(Remarks to the Author)
We appreciate the efforts made by the authors to address the issues raised in our review, and in those carried out by our colleagues (which were coherent with ours). Explanations provided are thorough and extensive. However, we are afraid that the authors did not clarify the main concern about the core of the paper, i.e. how the decarbonisation contribution and the strategic timing are conceived and calculated. Through the long explanations and descriptions, we fail to understand how these factors are actually calculated, how you can eventually say that e.g. "China's PV manufacturers could contribute up to 73.8% of the total decarbonization contributions". It may well be our own limitation, but all in all – although we still see value in the intuition of the paper (you cannot give full honour for decarbonisation to utilities installing PV modules and full blame to the manufacturers) – we must suggest that the paper should be rejected.

Reviewer #4

(Remarks to the Author)
I co-reviewed this manuscript with one of the reviewers who provided the listed reports. This is part of the Nature Communications initiative to facilitate training in peer review and to provide appropriate recognition for Early Career Researchers who co-review manuscripts.

Version 2:

Reviewer comments:

Reviewer #3

(Remarks to the Author)
We very much appreciate the further efforts made by the authors to improve the quality of the paper, and specifically to clarify the methodological issues that we had highlighted. Their work on the paper and the supplementary material is impressive.

Still, we are afraid that we are not convinced by the fundamental point of the paper.
In a nutshell, the authors say:

- Most PV emissions occur in manufacturing – and this is a fact
- Once the PV system is operational, it offsets emissions by displacing fossil fuels – and this is also true
- If we redistribute decarbonisation benefits proportionally, then the manufacturers (who are responsible for the high initial emissions) should also receive the largest credit for avoided emissions over the system's lifetime – and this is where the logic gets weak to our eyes

The authors do have a valid point in that PV manufacturers are essential for the clean energy transition, and they should not be unfairly penalised for their emissions while power companies get all the credit. We fully support this intuition. But their method of reallocating decarbonisation benefits is a stretch. Somehow, the logic is: "We create emissions so that emissions can be avoided later – therefore, we are the main drivers of decarbonisation." But that does not follow because the true driver of decarbonisation is deployment, not just production. No installation, no decarbonisation. In this perspective, the argument downplays the role of policymakers and utilities: the decision to install and use PV is made by energy providers and consumers, not manufacturers. If PV demand dropped, manufacturing would not reduce emissions on its own.

But even forgetting this point on deployment, the main objection remains: we support the idea of finding a criterion not to penalise manufacturers, but the following step so that they become the main contributors to decarbonisation only because originally they are the main emitters is not convincing.

Having said that, we very much respect the work of the authors, and we do not wish to further stress them out with additional discussion.

All in all, we leave the decision to the Editor. But thank you again for allowing us to reflect on this important point and for the valuable exchanges.

Reviewer #4

(Remarks to the Author)

I co-reviewed this manuscript with one of the reviewers who provided the listed reports. This is part of the Nature Communications initiative to facilitate training in peer review and to provide appropriate recognition for Early Career Researchers who co-review manuscripts.

Reviewer #5

(Remarks to the Author)
Thank you for the submission of this interesting paper. The concept to assign decarbonisation benefits to the manufacturers of RES systems is of innterest.
There are a few aspects, which are not yet clear to me.
1) Why only IEA 2022 scenarios, which are not very ambitious? (WEO 2024 with different numbers is available)
IPCC AR6 lists much more ambitious scenarios including 100% RES ones.
2) Which parts of the PV system value chain are considered and which share is attributed to the different production steps? E.g. mining, polysilicon production, wafer, solar cells, solar modules including contact materials, glass and polymers, BOS, installation.
The reason for this is that not all steps must be done in China.
3) The decarbonisation effects of PV manufacturing for China is described quite well, but the international effects could differ, if manufacturing takes palce in regions with a lower or even higher carbon footprint of the energy used. Please comment.
4) How will the energy mix in China change over time?
Its not clear if the decarbonisation is only due to the change of the electricity generation, or if there is also a decarbonisation effect of the whole energy mix (e,g. power to X, etc.)
5) line 176: What system prices are used for this calculation?
It is assumed that this is a micro economic analysis. Are macro economic aspects considered at all?
6) line 178: Assumption of stable Chinese share after 2027: Is this a realistic assumption?
So far there is only limited activity outside of China to build up the whole value chain of silicon PV (India). Some parts of the value chain namely polysilicon and wafer are concentrated in China and require a considerable time to scale somwhere else.
7) Fig 4: What are supporting modules? Is this the support structure where the modules are mounted? Do you differentiate between fixed mount, tracking and rooftop?

Version 3:

Reviewer comments:

Reviewer #5

(Remarks to the Author)
My comments were addressed adequately and the paper can now be published.

Reviewer #6

(Remarks to the Author)
General Comments

I congratulate the authors on the work undertaken to date and the detailed and careful manner in which their model has been built. This is a very relevant topic for consideration by Chinese policymakers and global policymakers, as well as by scholars of resource economics and policy.

Conceptually, however, the novelty or insight of the paper is not particularly clear. Although the allocation of manufacturing emissions to upstream firms whose output is constrained by policy based on those emissions (in China, at least) does create inefficiencies, the allocation of emissions to different parties across value chains depending on perspective is not a new one. To more clearly outline their contribution and argument, including in the paper's abstract, the authors should consider strengthen their engagement with the existing frameworks (e.g. GHG Protocol) and corresponding literature on emissions accounting, especially as it relates to industrial policy.

To illustrate this point, the paper argues that China's PV manufacturing industry has not been recognised for its contribution to decarbonisation. However, one could argue that existing GHG accounting frameworks do exactly this, by allocating the 'benefits' of PV manufacturing to producers in the form of low, or zero, Scope 3 emissions of the products they produce. When compared to Scope 3 emissions from substitutes (e.g. coal-fired power plants, gas-fired power plants), the benefits are clear. If previous work has not specifically highlighted this, it does not change the fact that current frameworks do, by design, measure the downstream emissions of manufactured products and, thereby, their relative performance against substitutes. Fundamentally, the insight here is not new, although the method used to arrive at it is different.

I do note that a range of points have been raised by other reviewers already. I have therefore aimed to focus my comments on areas that I do not believe have been addressed or discussed in previous rounds. Overall, while I am open to being convinced, the PDC modelling on its own is not sufficient to demonstrate the soundness of the policy implications. While

there is an argument to be made that the policies implied by the authors are desirable, the current paper's discussion section fails to make this argument in a compelling way, with meaningful reference with the very extensive literatures on resource economics and industrial policy that are instrumental to the interpretation of the paper's findings.

Specific Comments

The introduction makes clear that this approach is quite specific to China's domestic policy framework and incentives for producers, since there are no other comparable producers globally.

The economic theory underpinning the interpretation of the results in this paper is not very clear. The authors note that restrictions on high-emitting upstream enterprises is causing them to throttle production, particularly around the end of reporting periods. This is a well-known problem in China's current GHG management regime. However they do not convincingly establish that this is creating a supply shortage in PV panels, nor that it is desirable to remove restrictions on upstream suppliers' emissions if doing so disincentivises innovation in lower-carbon manufacturing. Again, it would be fruitful to engage more thoroughly with the literature on innovation in low-carbon technologies in order to explain why this theoretical trade-off is desirable. The authors could plausibly do so with reference to the payback period / decarbonisation contribution framework developed here: namely, how high would the decarbonisation contribution value have to be for the downsides of subsidising high-carbon upstream production to be overcome?

Indeed, the authors are currently arguing that it is always desirable, up to the maximum of the decarbonisation contribution curve (which varies in time according to the scenario and boundaries used), to provide policy support to PV manufacturing so that progress along pathways to net zero is not unnecessarily inhibited. This may be a defensible position, but in its current form the article should do more to base it in ongoing theory and evidence on how low-carbon industrial policy should be designed and why.

Since 2002, China has seen episodes of periodic, and in some cases structural, overproduction of industrial goods including steel, and of finished products including buildings and, arguably, PV panels. The authors mention this in the global context but do not address it with sufficient rigour in the domestic context, apparently dismissing it as a concern on the basis that China plans to quintuple installed solar & wind capacity between 2020 and 2035. Again, this may be a defensible position but it must be laid out in more detail.

Furthermore, the authors do not appear to consider that overproduction or overinstallation of PV itself decreases the marginal decarbonisation contribution of each panel, such that their proposed policies, to the extent that they generate overproduction, would reduce the contribution that they are trying to highlight. This highlights the risks of the 'blunt' approach to stimulating PV production proposed by the authors in (a) promoting the inefficient use of resources in the short term; (b) crowding out less carbon-intensive production within China and elsewhere. In other words, the authors do not engage properly with the risk that their proposals may well incentivise the embedding of a structural imbalance in upstream PV manufacturing that encourages incumbent high-carbon production and discourages innovation in either decarbonising the upstream element of the PV supply chain or developing entirely new technologies that complement or substitute for silicone PV.

Version 4:

Reviewer comments:

Reviewer #6

(Remarks to the Author)
The authors have addressed the comments that I raised and I have no further requests.

Open Access This Peer Review File is licensed under a Creative Commons Attribution 4.0 International License, which permits use, sharing, adaptation, distribution and reproduction in any medium or format, as long as you give appropriate credit to the original author(s) and the source, provide a link to the Creative Commons license, and indicate if changes were made.
In cases where reviewers are anonymous, credit should be given to 'Anonymous Referee' and the source.
The images or other third party material in this Peer Review File are included in the article's Creative Commons license, unless indicated otherwise in a credit line to the material. If material is not included in the article's Creative Commons license and your intended use is not permitted by statutory regulation or exceeds the permitted use, you will need to obtain permission directly from the copyright holder.
To view a copy of this license, visit https://creativecommons.org/licenses/by/4.0/

College of Energy Engineering
No. 38 Zheda Road, Zhejiang University
Hangzhou, P. R. China. 310027

Email: xgao1@zju.edu.cn

Xiang GAO, Ph.D., Professor, Academician
Member of the Chinese Academy of Engineering

Authors' Responses to Review Comments

Journal:	Nature Communications
Manuscript #:	NCOMMS-24-13270-T
Title of Paper:	Strategic Timing for the Potential Decarbonization Contributions by China's Photovoltaic Manufacturers in Global Energy Transition
Authors:	Qingyang Lin, Kai Wang, Chenglin Li, Kai Fang, Meixuan Xin, Siqi Wang, Anqi Xu, Zhenci Xu, Xiang Gao
Article Type:	Research paper

We appreciate the time and effort taken by the reviewers and editors in reviewing the manuscript and providing constructive and valuable comments, by which we believe that the quality of this paper has been much improved. We have substantially revised the manuscript by adding more examples and references, increasing transparency on some key concepts, and rephrasing certain sections in a more precise way. We hope that the changes we have made in the revised manuscript satisfies journal's standard. If not, however, we are happy to work further to make the paper acceptable for publication in Nature Communications.

Reviewer #1

This study tried to identify and quantify the historical and potential decarbonization contributions to national and global energy transition by China's PV manufacturers based on the most up-to-date data on industrial production from three criteria. It is an interesting topic, however the innovation is not enough, especially there are some heavy mistakes on research background, the EPR policy, and research gaps. Thus, I suggest rejecting it.

We appreciate your constructive feedback on our manuscript and feel encouraged by your comments. The paper has now been revised following your insightful comments and suggestions. In particular, we have substantially revised the introduction in such a way that the research background is now clearly stated. EPR and research gaps have also been carefully reviewed, demonstrating the unique contributions of our work. Following your comments, the manuscript has been largely improved with much enhanced innovations. We hope that you like the revised version.

Comment 1:

In general, the decarbonization contribution refers to the different lifecycle stage or products, which will be neutral. If you think the manufacturers will be the contribution body, whether it will result in the misunderstanding and overstate of the producer's role or not?

Response:

Thanks for bringing our attention to this important point. We think that recognizing the manufacturers as part of the contribution bodies does not cause misunderstanding and overstate producer's role. In the revised manuscript, we have clearly stated that we are not intended to recognize the manufacturers as the sole contribution body to PV decarbonization, but rather as part of the entire contribution bodies. In addition, we have used our proposed algorithms to allocate a reasonable share of the decarbonization contributions to manufacturers. Please find below our elaborations.

The lifecycle of PV spans a wide range of industries. The decarbonization contributions are made during the operation stage by producing electricity from solar energy, while most of the emissions are generated by the manufacturing industries. Note that there will be no decarbonization contributions at the operation stage without the initial emissions incurred during the manufacturing stage. As such, the production-based emissions have contributed to the overall decarbonization efforts. This perspective is supported by a growing number of literature (Liu et al., 2019; Jiang et al., 2019; Suvajit et al., 2021).

To avoid misunderstanding or overstating the producer's role, as pointed out by your comment, we have quantified the actual decarbonization contributions by manufacturers based on three criteria, namely energy, economic impact, and carbon emissions. This would ensure that the contribution of the manufacturing stage is fairly recognized.

References:

Liu, D., Liu, J., Wang, S., Xu, M. & Akbar, S. J. Contribution of international photovoltaic trade to global greenhouse gas emission reduction: the example of China. *Resources, Conservation and Recycling* **143**, 114-118 (2019).

Jiang, M., Gao, X., Guan, Q., Hao, X., An, F. The structural roles of sectors and their contributions to global carbon emissions: A complex network perspective. *Journal of Cleaner Production*, **208**, 426-435 (2019).

Suvajit, B., Muhammad, A. K., Muhammad I. H. Searching appropriate system boundary for accounting India's emission inventory for the responsibility to reduce carbon emissions. *Journal of Environmental Management*, **295**, 112907 (2021).

Comment 2:

In your paper- "China's PV manufacturers could contribute up to 74.9% of China's targets for decarbonizing in energy transition based on the announced pledges." How to calculate this contribution? Why so high? the other renewable energies only have 25% contribution in future?

Response:

Many thanks for pointing out this critical issue. We are sorry for our unclear expression that has led to the misunderstanding. The percentage mentioned in the paper does not refer to the contributions of China's PV manufacturers to the nation's overall energy transition. Instead, it represents the contributions from PV manufacturers to the decarbonization efforts specifically within the PV industry. This calculation, in this sense, excludes other renewable energy sources. We have removed this ambiguity and undertaken a refinement of the expression. To be more precise, China's PV manufacturers could contribute 35.4%-74.9% in the Stated Policies Scenario (STEPS) and 31.4%-73.8% in the Announced Pledges Scenario (APS) of the total decarbonization contributions attributed to the PV industry.

In this study, the total decarbonization contributions from PV are primarily attributed to the replacement of grid electricity with PV-generated electricity (Wang et al., 2024). When determining this total decarbonization contributions, we have utilized the grid-level carbon intensity that is changing. This is determined by the predicted electricity structure from various technologies and the total amount of generated electricity until 2050, as projected by the International Energy Agency (IEA) under different scenarios (See now Part 1 in the Methods section). We then applied our proposed algorithm, based on three criteria, to allocate a reasonable share of these contributions to the PV manufacturers.

We have modified the texts accordingly.

Abstract:

"Results show that China's PV manufacturers could contribute up to 73.8% of the total decarbonization contributions by the entire PV industry towards China's targets for decarbonizing the energy sector based on the announced pledges."

Discussion and implications:

"Our analysis indicates that China's PV manufacturers could contribute a substantial 73.8% of the total decarbonization contributions by the entire PV industry towards achieving China's decarbonization targets in energy transition under the APS scenario."

Methods:

*"**The potential decarbonization contributions of PV systems.** In this study, the decarbonization contributions of PV systems is defined as the sum of reduced emissions by PV-generated electricity replacing grid electricity instead of comparing PV with coal-fired power plants (Wang et al., 2024). To determine the total decarbonization contributions, we utilized a dynamically changing carbon intensity for the grid, as projected by the IEA under different scenarios, including STEPS, APS and NZE. This dynamic approach accounts for the predicted electricity structure from various technologies and the total amount of generated electricity until 2050."*

Reference:

Wang, L., Qiu, T., Zhang, M., Cao, Q., Qin, W., Wang, S., Wang, L., Chen, D., Wild M. Carbon emissions and reduction performance of photovoltaic systems in China, *Renewable and Sustainable Energy Reviews*, **200**, 114603 (2024).

Comment 3:

The strategic timing is your key point, what's the definition and explanation? Before and after strategic timing, what's the differences for the PV system application? I don't think that the decreasing avoid emissions from the electricity will have large effects on the PV industry development.

Response:

We appreciate your valuable comment. We have defined strategic timing as the predicted time point with the highest producer decarbonization contributions by PV manufacturers. We have revised the text where necessary to make the definition consistent throughout the manuscript. By doing so, there are two key policy implications for planning the development of the PV manufacturing industry:

1. Prior to this strategic timing, we anticipate a rising trajectory in the potential decarbonization contributions from PV manufacturers. This period is critical for maximizing the decarbonization contributions because it represents a phase where significant gains in decarbonization can be achieved through the deployment of PV systems. Therefore, during this period, supportive regulations and policies are essential to ensure rapid development of the PV manufacturing industry for accelerating energy transition.

2. After the strategic timing, we observe a decline in the decarbonization contributions from PV manufacturers as the carbon intensity of grid electricity continues to decrease. This decline aligns with your point that the decreasing avoided emissions from electricity will not impact the PV industry development. More stringent regulations and policies can be applied to the PV manufacturing industry to prevent potential excess production capacity and resource waste (IEA, 2024; Wang et al., 2022). Such actions, however, will not significantly hinder the overall energy transition, as the marginal decarbonization contributions from new PV installations will be lower.

We have now made sure that the definitions are clear and consistent throughout the manuscript and explained how it can affect the PV manufacturing industry.

Abstract:

"We also observe that the potential decarbonization contributions by China's PV manufacturers will increase until 2030–2035 and decrease later, which allows for the anticipation of the strategic timing — predicted time point with the highest producer decarbonization contributions by PV manufacturers indicating the critical time point to switch from supportive policies for PV manufacturers industry to more stringent ones to ensure success energy transition and avoid potential excess production capacity and resource waste."

Introduction:

"We then introduce strategic timing, the predicted period when the decarbonization contributions from PV manufacturers reach their maximum. This timing is critical for planning the development of the PV manufacturing industry and ensuring that supportive regulations and policies are in place to maximize contributions to the energy transition while more stringent regulations and policies can be applied to the PV manufacturing industry to prevent potential excess production capacity and resource waste."

Discussion and implications:

"Prior to this timing, we anticipate a rising trajectory in the potential decarbonization contributions of the PV manufacturing industry primarily driven by the expanding deployment of PV systems, as the carbon intensity of PV-generated electricity is substantially lower than that of the grid electricity. During this period, supportive regulations and policies are essential to ensure the rapid development of the PV manufacturing industry for successful energy transition. After the strategic timing, we observe a decline in these contributions as the carbon intensity of grid electricity reaches a significantly low level that is very close to the PV-generated electricity. All this suggests a closing window of opportunity to secure a cleaner and sustainable energy transition, beyond which rapidly expanding deployment of PV systems may not be desirable. Instead, more stringent regulations and policies can be applied to the PV manufacturing industry to prevent potential excess production capacity and resource waste (IEA, 2024; Wang et al., 2022). Such actions will not significantly hinder the overall energy transition, as the marginal decarbonization contributions from new PV installations will be lower."

References:

Renewables 2023. (International Energy Agency, Paris, 2024).

Wang, C., Feng, K., Liu, X., Wang, P., Chen, W.-Q. & Li, J. Looming challenge of photovoltaic waste under China's solar ambition: A spatial–temporal assessment. *Applied Energy,* **307**, 118186 (2022).

Comment 4:

In the introduction, I think that you have some misunderstanding on the EPR policy. EPR is not only the responsibility of producers, but also refers to other stakeholders (such as the Government and consumers). EPR is based on the principle that manufacturers (usually brand owners) have the greatest control over product design and marketing and have the greatest ability and responsibility to reduce toxicity and waste. The core goal is to realize the effective management of the product waste for improving product recovery and minimizing the environmental impact of waste. Thus, your background needs to be rewritten for the research gaps.

Response:

We appreciate your advice on understanding the EPR policy. In our study, we use EPR policy to explain that many studies focus on the responsibilities for environmental impact when discussing producers in industry chain (Louwen et al., 2016; Pu et al., 2021). However, for the PV industry chain, underestimating the PV manufactures decarbonization contributions would definitely weaken the efforts of emission reduction that the whole PV industry makes. This is the main research gap in which our study tries to fill.

We have followed your suggestion to bring clarity to the principle of EPR. OECD (2001) defined EPR as a law mandating that the financial and physical responsibility of products used by consumers is shifted from consumers to the other stakeholders (such as manufacturers and brand owners). EPR is based on the polluter-pays principle (Steenmans and Ulfbeck, 2023), aiming to foster improvements in product management to minimize associated environmental impact (Maitre-Ekern, 2021). Therefore, EPR is widely used in the studies that deal with waste management and pollution

prevention. In this study, EPR can explain why the implementation of Dual Carbon Reduction Strategy will pose substantial challenge to PV indurates, especially to manufacturing industry. PV manufacturers produce substantial carbon emissions are therefore responsible for reducing toxicity and waste across a product's entire lifespan.

We see your point that stressing on the EPR may be inappropriate, especially we did not intend to go to details as it is not the main focus of our study. To remove ambiguity, we have now removed the detailed discussion of the EPR. Instead, we have proposed a new framework—the Producers Decarbonization Contribution (PDC) that emphasizes the decarbonization contributions from producers over the lifecycle of the PV systems. Unlike EPR, the PDC focuses on producers' decarbonization contributions, with the aim of supporting sustainable development of the entire industry chain that facilitate decarbonization.

We have re-organized the introduction accordingly:

Introduction:

"Prevalent studies have tended to attribute the most emission responsibility to producers (Banerjee et al., 2021; Ye et al., 2023) while completely ignoring their decarbonization contributions. This means that only power enterprises are credited for the decarbonization of PV systems, overlooking the fact that PV manufacturers are indispensable for structural decarbonization. Ignoring the decarbonization contributions by PV manufacturers would definitely weaken the efforts of emission reduction that the whole PV industry makes."

"This study aims to show, for the first time, the overlooked contributions of PV manufacturing industry to both China's and global decarbonization progress. We propose the producer decarbonization contributions (PDC) framework emphasizing the contributions of emission reduction that producers make over the lifecycle of the PV system."

References:

Banerjee, S., Aamir Khan, M. & Iftikhar ul Husnain, M. Searching appropriate system boundary for accounting India's emission inventory for the responsibility to reduce carbon emissions. *Journal of Environmental Management,* **295**, 112907 (2021).

Extended producer responsibility. (OECD, 2001).

Louwen, A., van Sark, W., Faaij, A. et al. Re-assessment of net energy production and greenhouse gas emissions avoidance after 40 years of photovoltaics development. *Nature Communications*, **7**, 13728 (2016).

Maitre-Ekern M. Re-thinking producer responsibility for a sustainable circular economy from extended producer responsibility to pre-market producer responsibility. *Journal of Cleaner Production,* **286**: 125454 (2021).

Pu, Y., Wang P., Wang Y., Qiao, W., Wang L., Zhang, Y. Environmental effects evaluation of photovoltaic power industry in China on life cycle assessment. *Journal of Cleaner Production,* **278**, 123993 (2021).

Steenmans K, Ulfbeck V. Fostering the circular economy through private law: Perspectives from

the extended producer responsibility concept. *Resources, Conservation and Recycling,* **195**: 107016 (2023).

Ye, Q., Krol, M. S., Shan, Y., Schyns, J. F., Berger, M. & Hubacek, K. Allocating capital-associated CO2 emissions along the full lifespan of capital investments helps diffuse emission responsibility. *Nature Communications,* **14**, 2727 (2023).

Comment 5:

Line 206-209, "As China advances its Dual Carbon Reduction Strategy, policymakers are contemplating the imposition of more stringent regulations for those manufacturers generating carbon emissions enormously." Why it is the obstacles of the PV development?

Response:

Thanks for your comment. The China's main strategy to combat climate change currently involves using both total emissions and emission intensity as binding indicators to assess local government performance on emission reduction. This strategy sets carbon emissions as "budgets" at the provincial and corporate levels (United Nations Conference on Trade and Development, 2023; Wei et al., 2023). Consequently, many high-emission enterprises may be forced to halt or limit production, which can ultimately undermine industrial development. The PV industry has a long production chain that involves numerous upstream manufacturers labeled as high emitting enterprises, despite the provisioning of basic materials for the renewable energy sector (Tanveer et al., 2022; Muhammad et al., 2021). As a result, some PV manufacturers still face stringent regulations that lead to difficulties in capacity expansion and increase in product costs (Wang et al., 2023). All this pose obstacles to the development of the PV industry.

We have now updated the manuscript with the following texts in the introduction:

"The implementation of this strategy poses substantial challenges to various industries, including manufacturing industry, for their sustainable development. This is because manufacturers produce significant carbon emissions and are therefore responsible for managing environmental impacts and resource consumption across a product's entire lifespan (Khawaja et al., 2021l Jenkins et al., 2023; Heath et al., 2020). In practice, this involves setting carbon emissions or energy use 'budgets' at the provincial and enterprise levels (Wei et al., 2023). Consequently, many high-emission and high-energy-consuming enterprises may be forced to halt or limit production, which can ultimately constrain industry development and impact economic growth. The PV industry, in particular, has a long production chain involving numerous upstream manufacturers labelled as high emissions and high energy consumption enterprises, despite producing components for the renewable energy sector (Tanveer et al., 2022; Muhammad et al., 2021, Gan et al., 2023). Although the overall strategy is transitioning from 'dual control of energy consumption' to 'dual control of carbon emissions' (United Nations Conference on Trade and Development, 2023), partly to encourage the use of renewable energy, these manufacturers could still face significant disruptions, difficulties in capacity expansion, and increased product costs. This could ultimately hinder the overall development of the PV industry and energy transition."

References:

China's policy strategies for green low-carbon development: Perspective from South-South cooperation. (United Nations Conference on Trade and Development, 2023).

Gan, Y., Elgowainy, A., Lu, Z., Kelly, J. C., Wang, M., Boardman, R. D. *et al.* Greenhouse gas emissions embodied in the U.S. solar photovoltaic supply chain. *Environmental Research Letters* **18**, 104012 (2023).

Heath, G. A., Silverman, T. J., Kempe, M., Deceglie, M., Ravikumar, D., Remo, T. *et al.* Research and development priorities for silicon photovoltaic module recycling to support a circular economy. *Nature Energy* **5**, 502-510 (2020).

Jenkins, S., Kuijper, M., Helferty, H., Girardin, C. & Allen, M. Extended producer responsibility for fossil fuels*. *Environmental Research Letters* **18**, 011005 (2023).

Khawaja, M. K., Ghaith, M. & Alkhalidi, A. Public-private partnership versus extended producer responsibility for end-of-life of photovoltaic modules management policy. *Solar Energy* **222**, 193-201 (2021).

Muhammad, T., Amani, A., Feras, K., Emad, A., Fares, A., Malek, A. Environmental impacts of solar photovoltaic systems: A critical review of recent progress and future outlook. *Science of The Total Environment*, **759**, 143528 (2021).

Tanveer, H. M., Eskinder, G., Amit, K. Life cycle greenhouse gas emissions and energy footprints of utility-scale solar energy systems. *Applied Energy*, **314**, 118918 (2022).

Wei, Y., Zhao, T., Wang, J., Zhang, X. & Li, Z. Two-level allocation and its future change of CO2 reduction responsibility in China's power sector. *Environmental Impact Assessment Review,* **99** (2023).

Wang, W., Wang, Z., Yu, Z., Feng, C. Transition towards dual control of CO2 emissions and intensity through supply chain management in China. *Journal of Environmental Management*, **348**, 119493 (2023).

Comment 6:

In the discussion parts, it is suggested to discuss the excess production capacity of PV and the global competition challenges.

Response:

Thank you for this important suggestion, which is indeed closely related to our definition on strategic timing. We have now added discussion related excessive production capacity, which is reflected by the policy implications after the predicted strategic timing. This timing represents the point by which the decarbonization contributions of the PV manufacturing industry are maximized. After this point, the contributions would gradually decrease primarily due to the rapid decline in average emission intensity for the grid, based on which we quantify the decarbonization contributions. Note that in this study, rather than using static emission intensity from the grid or using coal-fired electricity to compute the PV decarbonization contributions, we take into account the dynamically changing emission intensity from the grid when approaching 2050. We have recommended flexible policies to first encourage development of the PV manufactures before the

decarbonization contributions reach the maximum by the strategic timing, and then turn to gradually strengthening regulations to prevent potential excessive production capacity and resource waste (IEA, 2024; Wang et al., 2022).

Below are the texts we revised:

Discussion and implications:

"After the strategic timing, we observe a decline in these contributions as the carbon intensity of grid electricity reaches a significantly low level that is very close to the PV-generated electricity. All this suggests a closing window of opportunity to secure a cleaner and sustainable energy transition, beyond which rapidly expanding deployment of PV systems may not be desirable. Instead, more stringent regulations and policies can be applied to the PV manufacturing industry to prevent potential excess production capacity and resource waste (IEA, 2024; Wang et al., 2022). Such actions will not significantly hinder the overall energy transition, as the marginal decarbonization contributions from new PV installations will be lower."

References:

Renewables 2023. (International Energy Agency, Paris, 2024).

Wang, C., Feng, K., Liu, X., Wang, P., Chen, W.-Q. & Li, J. Looming challenge of photovoltaic waste under China's solar ambition: A spatial–temporal assessment. *Applied Energy,* **307**, 118186 (2022).

Comment 7:

For the PV system, did you consider the difference between the distributed PV and centralized PV?

Response:

Thank you for this comment. Indeed, we did not explicitly differentiate between these two types of PV systems. The amount of electricity generated by PV modules has been determined by considering the installed capacity of the PV system (MWp), the annual peak sunshine hours, the performance ratio of the PV power plant (assumed to be 79%), and the average loss coefficients of electricity transmission in China (currently below 5%, see Supplementary Table 16). In our framework, following the current policies that ensure efficient utilization of renewable-generated electricity, centralized PV systems are connected to the grid that allows for efficient distribution and use of generated electricity, while distributed PV systems can be equipped with energy storage solutions, enabling effective management and optimization of electricity usage (China Energy Storage Alliance, 2024; Bullich-Massagué, 2020, National Development and Reform Commission and National Energy Administration, 2021). Moreover, with respect to economic costs, the annual operation and maintenance costs in 2023 for centralized and distributed PV systems are 39,000 CNY/MW and 47,000 CNY/MW, respectively (China Photovoltaic Industry Association, 2023). The difference in operation and maintenance costs is minimal compared to the initial investment cost of 3.18–3.40 million CNY/MW for PV systems. Therefore, the difference in terms of costs also does not significantly impact the main conclusions of this study.

Reference:

China's Photovoltaic Industry Development Roadmap (2018-2022). (China Photovoltaic Industry Association, 2023).

Energy Storage Industry White Paper 2024. (China Energy Storage Alliance, 2024).

Bullich-Massagué, E., Cifuentes-García, F., Glenny-Crende, I., et al. A review of energy storage technologies for large scale photovoltaic power plants. *Applied Energy,* **275**, 116727 (2020).

Promoting the integration of charge and storage and multi-energy complementary development of power sources (in Chinese). (National Development and Reform Commission and National Energy Administration, 2021). https://www.gov.cn/zhengce/zhengceku/2021-03/06/content_5590895.htm

Comment 8:

When calculating the PV developing potential in future, do you consider the saturability situation?

Response:

Thank you for bringing the saturability situation into focus. The saturability can indeed influence the PV developing potential in future, as it can the amount of electricity generated by PV, particular for long term. Therefore, it becomes critical when quantifying decarbonization contributions by PV particularly when using static emission intensity from the grid or using coal-fired electricity as benchmarks. Especially, for long term, due to massive amount of electricity generated by PV (See Table 1 below), saturability can have a significant influence, causing big uncertainties when assessing decarbonization contributions.

In this study, instead of using a static emission intensity, we have considered the dynamically changing intensity from the grid based on the predicted electricity generation structure and amount. This means that in addition to the amount of PV-generated electricity, the emission intensity of the grid which is gradually declining, is another main controlling parameters determining decarbonization contributions. At early stage, due to the large difference between carbon intensities between PV-generated and grid electricity, PV's decarbonization contributions increases rapidly. For long term, where saturability plays an important role, even though the PV-generated electricity could increase 138 times (from 40 to 5442 TWh), the actual decarbonization contributions from PV increases <15 times (see Figure 1 below). This is because the grid intensity is continuously declining. Therefore, in our framework, saturability's influence to the PV's contributions is becoming less and less insignificant, especially for long term when approaching net-zero. This means that saturability will not affect our conclusions.

Table 1. PV-generated electricity, carbon intensities of the grid and PV-generated electricity in China under APS scenario.

Year	PV-generated electricity (TWh)	Carbon intensity of the grid (gCO_2 eq/kWh)
2015	40	599.40
2020	261	549.22
2030	1586	394.36
2035	2505	297.24
2040	3614	196.93

2045	4625	132.32
2050	5442	79.82

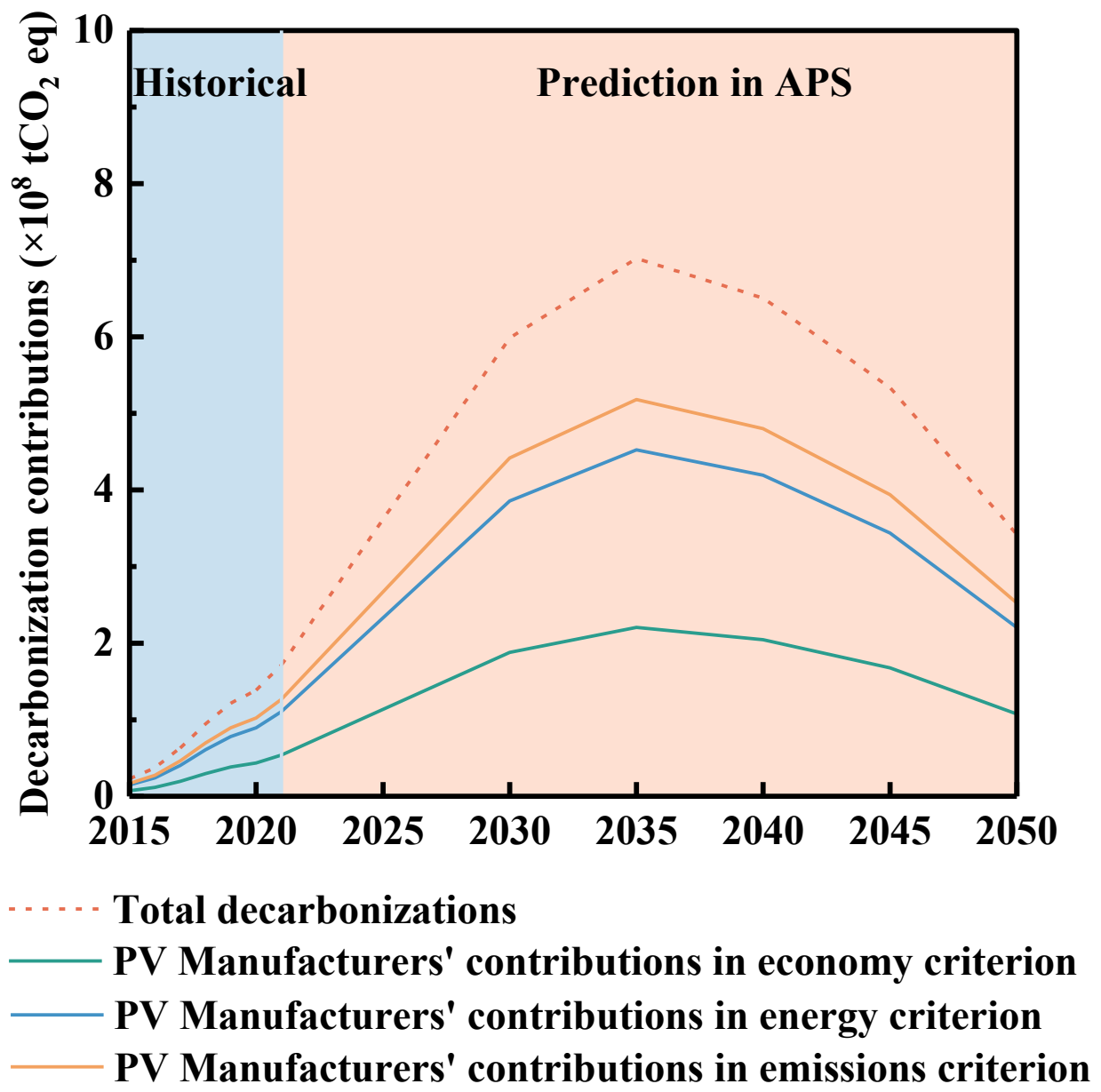

Figure 1. The potential decarbonization contributions and associated strategic timing of PV manufacturing industry in decarbonization of power sector in China (APS).

Comment 9:

You use "1 MWp mono-crystalline PV system" as your functional units and typical PV system, please show the reasons why 1 MWp and mono-crystalline PV system.

Response:

1 MWp system represents a suitable-sized PV installation that is commonly found in industrial parks and can also serve as a unit module for large-scale centralized PV systems. The use of 1 MWp as a functional unit is well-established in life cycle assessment (LCA) studies of PV systems, that can provide a balance between detail and generality (Yu et al., 2017). Moreover, mono-crystalline silicon modules have become the mainstream technology in the PV market. By 2023, the market share of mono-crystalline silicon wafers (both p-type and n-type) had exceeded 99% in the Chinese market, with p-type mono-crystalline silicon wafers alone accounting for 74.5% of the market (CPIA, 2023).

We have now added the following information in the Supplementary Notes 1.

"We used a 1 MWp mono-crystalline PV system as our functional unit and typical PV system for several reasons. First, a 1 MWp system represents a suitable-sized PV installation that is commonly found in industrial parks and can also serve as a unit module for large-scale centralized PV systems. The use of 1 MWp as a functional unit is well-established in life cycle assessment (LCA) studies of PV systems, providing a balance between detail and generality. Moreover, Mono-crystalline silicon modules have become the mainstream technology in the PV market. By 2023, the market share of mono-crystalline silicon wafers (both p-type and n-type) had exceeded 99% in the Chinese market, with p-type mono-crystalline silicon wafers alone accounting for 74.5% of the market."

References:

China's Photovoltaic Industry Development Roadmap 2023. (CPIA, 2023).

Yu, Z., Ma, W., Xie, K., Lv, G., Chen, Z., Wu, J., Yu, J. Life cycle assessment of grid-connected power generation from metallurgical route multi-crystalline silicon photovoltaic system in China. *Applied Energy*, **185**, 68–81 (2017).

Comment 10:

More details of three scenarios should be explained in the manuscript or supporting information.

Response:

We have added the following details to the supporting information.

*"**The Net Zero Emissions by 2050** (NZE) Scenario shows a narrow but achievable pathway for the global energy sector to achieve net zero CO2 emissions by 2050, with advanced economies reaching net zero emissions in advance of the other scenarios. This scenario also meets key energy-related United Nations Sustainable Development Goals (SDGs), in particular achieving universal energy access by 2030. The NZE Scenario does not rely on emissions reductions from outside the energy sector to achieve its goals, but assumes that non-energy emissions will be reduced in the same proportion as energy emissions. It is consistent with limiting the global temperature rise to 1.5 °C without a temperature overshoot (with a 50% probability) (IEA, 2023).*

***The Announced Pledges Scenario** (APS) takes account of all the climate commitments made by governments around the world including Nationally Determined Contributions as well as longer term net zero emissions targets, and assumes that they will be met in full and on time. The global trends in this scenario represent the cumulative extent of the world's ambition to tackle climate change as of mid-2022. The remaining difference in global emissions between the APS and the goals in the NZE Scenario shows the "ambition gap" that needs to be closed to achieve the goals agreed in the Paris Agreement in 2015 (IEA, 2023).*

***The Stated Policies Scenario** (STEPS) does not take for granted that governments will reach all announced goals. Instead, it explores where the energy system might go without additional policy implementation. As with the APS, it is not designed to achieve a particular outcome. It takes a granular, sector-by-sector look at existing policies and measures and those under development. The remaining difference in global emissions between the STEPS and the APS represents the "implementation gap" that needs to be closed for countries to achieve their announced decarbonization targets (IEA, 2023)."*

Reference:

World energy outlook 2022. IEA (2023).

Supporting information

Comment 11:

In the table 9, what' the transportation distances from China to other countries?

Response:

We appreciate your comment regarding the transportation distances from China to other countries. At the China national scale, we assumed a transport distance of 170 km, where the impact of the transportation process was minimal, accounting for only 0.17% of primary energy demand and 0.23% of carbon emissions. However, at the global scale, the transport distances increase significantly. We have added an uncertainty analysis for transport distances at the global scale (see Supplementary Table 35). For instance, transporting from the Port of Ningbo-Zhoushan in China to the Port of Mumbai, India (8,534 km), the Port of Rotterdam, Netherlands (19,448 km), the Port of Rio de Janeiro, Brazil (23,782 km), and the Port of Mazatlán, Mexico (12,307 km) results in increased energy consumption and carbon emissions. These international transport distances lead to an increase of 2.0%–5.5% in lifecycle energy consumption and 2.7%–7.7% in lifecycle carbon emissions. Thus, we have incorporated the long-distance transport process of this portion of PV components into our study, by assuming an average transport distance of 16,000 km in the global scenario for our calculations. All the calculations have been updated with longer distance between China and other countries and an uncertainty analysis below has been provided in Supplementary Material as well. Noted that the values in Supplementary 35 also considers the emissions related to the mining of silica ores inspired by Reviewer #3. Nevertheless, the conclusions of this study remain unchanged.

We have updated the texts in Supplementary Note 4:

"For the transportation process, there is an extra long-distance transport process from China to other countries. Supplementary Table 35 presents an uncertainty analysis of transport distances, with an ultimate selection of an average transport distance of 16000 km."

Supplementary Table 35. The uncertainty analysis of transport distances.

Destination	Distances (km)	Increase in primary energy demands	Increase in carbon emissions
Port of Mumbai (India)	8534	2.0%	2.7%
Port of Mazatlan (Mexico)	12307	2.8%	4.0%
Port of Rotterdam (Netherlands)	19448	4.5%	6.3%
Port of Rio de Janeiro (Brazil)	23782	5.5%	7.7%

Comment 12:

Line 113-114, "which are 920 and -1.28×10^4 (STEPS) and -1.08×10^4 (APS) tCO2 eq from Fig. 3a", please check the data.

Response:

Sorry for the typo. It should be Fig. 2a. We have now corrected it.

Comment 13:

For the table 12, not all the materials can have the 100% substitution of the primary materials. The ratio should be given for different materials.

Response:

We assume that only primary materials are used during the manufacturing stage, as recycled materials can be used for various purposes beyond making new PV systems. During the decommissioning stage, the associated decarbonization contribution through avoid usage of primary materials based on the amount of valuable materials that can be recycled. The emission intensities for recycled products obtained from the Gabi database.

We agree that not all the materials can be fully recycled. To make the data clearer, we have now included a new table (Supplementary Table 14) in the supplementary information that clearly states the percentage of valuable materials that can be recycled, ensuring transparency and accuracy in our calculations.

Supplementary Table 14. The recovery rate of main materials.

Item	Materials	The recovery rate
PV panels	Aluminum frame	100%
	Copper	84.3%
	Silicon	88.4%
	Glass	93.5%
	Sliver	90%
	Plastic (Including TPT and EVA)	15.0%
Inverter	Steel	90%
	Copper	90%
	Aluminum	90%
Holder	Steel	90%
	Aluminum	90%
Junction box	Copper	90%
Cable	Copper	90%

Note: The aluminium frames of PV panels were assumed to be 100% recovered as they were manually dismantled. The recovery rates for other materials in PV panels were calculated based on data from the literature (Ansanelli, 2021). The inverter, holder, junction box, and cable also contained valuable metals, with an assumed recovery rate of 90% in this study.

Reference:

Ansanelli, G., Fiorentino, G., Tammaro, M., & Zucaro, A., A Life Cycle Assessment of a recovery process from End-of-Life Photovoltaic Panels. *Applied Energy,* **290**, 116727 (2021).

Comment 14:

Line 153-154, “The price of electricity for businesses ranges from 0.011 to 5.605 CNY/kWh”. Why so large difference differences (560 times) for the electricity prices?

Response:

Our initial description included some extreme cases when we performed calculations at the global scale (e.g., very low prices in regions like Iran, Syria, Sudan; and higher prices in regions like Poland and Italy) (data sources: (https://www.globalpetrolprices.com/electricity_prices/?ref=interneteconomist.com). For the calculation at the global scale, we have ultimately used 1.33 CNY/kWh for our final calculations, and have also conducted sensitivity and uncertainty analyses with electricity prices ranging from 0.7 to 3.85 CNY/kWh.

We have now added the following statement to the supporting information:

"To provide a clearer understanding of the electricity prices used in our analysis, for the context of China, we used an electricity price of 0.454 CNY/kWh. For global scenarios, sensitivity and uncertainty analyses were conducted with electricity prices ranging from 0.7 to 3.85 CNY/kWh. The final calculations were based on an electricity price of 1.33 CNY/kWh."

Comment 15:

The data source in the text and tables should show the references.

Response:

We have now updated the entire supporting information file and cited reference in the text as well.

Reviewer #2

The paper presents an interesting aspect of the responsibilities of carbon emissions not only on the side of the manufacturer but also on the side of the power enterprises to achieve decarbonization of the electricity grids. Hence, it is useful and important for scientist and policymakers alike, warranting for publication provided that the following remarks are taken into account.

Thanks for your positive and supportive comments. Your interpretation is exactly the way we intended. We have now addressed all your comments and believe the manuscript has been significantly improved following your advice.

Comment 1:

Page 3, line 100: We find that in China the PV manufacturing stage account for 77.8% of energy consumption, 58.7% of economic costs, and 80.7% of carbon emissions (Fig. 1). Please give an indication how the values presented here for the energy consumption, the economic costs, and the carbon emissions have been determined. The values fall out of the sky.

The contributions of energy consumption, economic costs, and emissions from PV manufacturers were determined by evaluating these three criteria over the lifecycle of the PV system. This includes nine processes spanning the manufacturing, transportation and construction, operation, and decommission stages (Processes 1-6 shown in blue in Fig. 1) (Müller et al., 2021). Detailed equations can be found in Part 2 of the Methods section, with source data provided in Supplementary

Notes 1-2.

Page 3, line 101: The decarbonization contributions are achieved by comparing the carbon intensity of PV-generated electricity (16.94 gCO2 eq/kWh using the latest industrial production data, see Methods section for more details) and the average carbon intensity for various technologies supplying the grid (543 gCO2 eq/kWh) over 30 years. Please give more details here. Which technologies are included in the average carbon intensity supplying the grid? Which grid region was considered for the average carbon intensity? How is the decarbonization contributions determined? In general, it is difficult to understand it when there is no guideline.

The grid intensity refers to the average intensity for China's national grid, considering 10 different electricity generation methods, including coal, natural gas, wind, solar PV, modern bioenergy, hydro, nuclear, oil, fossil fuels with CCUS, and others. This information has now been clearly stated in the Methods section:

"The annual dynamic carbon intensity of the grid ($CI_{grid,t}$) is calculated based on the energy supply structure and carbon intensities for different means using equation (2). In this study, we consider 10 different electricity generation methods, including coal, natural gas, wind, solar PV, modern bioenergy, hydro, nuclear, oil, fossil fuels with CCUS, and others (see Supplementary Notes 3–4 for more details)."

The decarbonization contributions of PV systems have been determined by comparing the carbon intensity of PV-generated electricity with the average carbon intensity of the 10 different electricity generation methods supplying the grid in China over the PV system's lifespan (see Part 1 in Methods for detailed equations). Note that we have utilized a dynamically changing carbon intensity for the grid based on the projected energy structure and electricity generation capacity by the IEA under different scenarios.

Page 3, line 106: The payback times on energy (Ten= 2.73 years), economy ($Teco$= 8.16 years) and carbon emissions (Tem=1.32 years) criteria are used to determine the assigned decarbonization contributions, ... Where do these values come from? How were the values determined? Please give an indication how the values presented here for the energy, economy and carbon emissions payback times were determined. In general, it is difficult to understand it when there is no guideline.

In this study, we have evaluated the energy consumption, economic costs, and associated emissions over the lifecycle of the PV system, covering nine processes including manufacturing, transportation and construction, operation, and decommission stages (see source data in Supplementary Note 2). The decarbonization contributions of PV systems are determined by comparing the carbon intensity of PV-generated electricity, computed to be 16.94 gCO_2 eq/kWh, with the dynamically changing intensity for the grid based on the predicted energy structure. Knowing the amount of electricity generated, revenue generated, and emissions reduced each year, we have determined the year after which the operation of the PV system covers its lifecycle energy consumption, economic costs, and associated emissions. These are referred to as payback times in this study. Specifically, the decarbonization assigned to manufacturers as contributions is the amount of decarbonization achieved after the payback time until the end of the lifecycle (Antonanzas and Quinn, 2021).

Page 4, line 116: The determination of carbon emissions per unit capacity PV system, associated data sources, and the determination of payback times from different criteria are provided in the Supplementary Notes 1−3. As the determination of carbon emissions and the determination of payback times is considered very important for the motivation of the paper, it should be place in the paper and not in the supplementary only. Please find a good balance between presenting important and necessary information concerning the determination of carbon emissions and the determination of payback times in the paper and in the supplementary.

Thank you for the comment for further improving the manuscript. In the previous version, even though we have included relevant details in the Methods and Supplementary Information, understanding these details while reading was indeed not smooth enough. Following your suggestion, we have now added more descriptions at the beginning of the results section to better link with details in Methods section and supplementary information. With your advice, we believe the manuscript has been significantly improved.

Response:

We have re-written the first paragraph of the results section to reflect the changes:

"The decarbonization contributions of PV systems are determined by comparing the carbon intensity of PV-generated electricity with the average carbon intensity of 10 different electricity generation methods supplying the grid in China over the lifespan of the PV system. We utilize a dynamically changing carbon intensity for the grid, based on the projected energy structure and electricity generation capacity provided by the IEA under three different scenarios. For PV systems, we evaluate the energy consumption, economic costs, and associated emissions over the lifecycle of the PV system including manufacturing, transportation and construction, operation, and decommission stages (Müller et al., 2021). Our findings indicate that in China, the PV manufacturing stage (Processes 1-6 out of 9 belong to manufacturing stage) accounts for 77.8% of energy consumption, 58.7% of economic costs, and 80.7% of carbon emissions (see Fig. 1). The carbon intensity for PV-generated electricity is determined to be 16.94 gCO_2 eq/kWh. The determination of carbon intensity for the PV-generated and grid electricity can be found in Parts 1-2 of the Methods. By considering the amount of generated electricity, revenue, and reduced emissions by PV each year, we can determine the payback times (Antonanzas and Quinn, 2021) for covering lifecycle energy consumption (T_{en} = 2.7 years), economic costs (T_{eco} = 8.2 years), and associated emissions (T_{em} =1.3 years) (see Fig. 2a and Part 3 in Methods). The decarbonization contributions assigned to manufacturers, involving 6 out of 9 processes (see Part 4 in Methods), are calculated as the amount of decarbonization achieved after the payback time until the end of the lifecycle. As shown in Fig. 2, for a 1MWp PV system operating for 30 years in China, considering the payback times from the three criteria, the corresponding assigned decarbonization contributions are estimated to be 60.2%–92.8% in STEPS, and 53.5%–91.5% in APS. The corresponding percentage potential decarbonization contributions from manufacturing stage are 35.4%–74.9% in STEPS, and 31.4%–73.8% in APS. The associated source data is provided in the Supplementary Notes 1–3."

References:

Antonanzas, J., Quinn, J.C. Net environmental impact of the PV industry from 2000-2025. *Journal of Cleaner Production*, **311**, 127791 (2021).

Müller, A., Friedrich, L., Reichel, C., Herceg, S., Mittag, M. & Neuhaus, D. H. A comparative life cycle assessment of silicon PV modules: Impact of module design, manufacturing location and inventory. *Solar Energy Materials and Solar Cells,* **230**, 111277 (2021).

Comment 2:

Page 3, line 103: The carbon intensity of PV-generated electricity has considered both emissions and emissions reduction through recycling in the decommissioning stage. Is recycling of the module or recycling of the photovoltaic power plant meant here?

Response:

In this study, the decommissioning stage, we are recycling the plant, including PV modules and valuable materials from inverters, holders, junction box, cables (See Supplementary Tables 12-13) (Ansanelli et al., 2021). We have also considered associated emissions when dealing with non-recyclable concrete (See Supplementary tables 13). We have now removed this sentence to improve the flow of the paragraph. Instead, in the Methods section, we have the following text describing this information:

"During decommissioning, in addition to PV modules, a significant amount of metal resources, especially from components including aluminum frames, inverters, PV holders, and cables, can be recycled (Ansanelli et al., 2021). This accounts for 1.4% of the total reduction. The associated emissions due to non-recyclable materials, such as concrete, are also considered."

Reference:

Ansanelli. G., Fiorentino, G., Tammaro, M. & Zucaro, A. A Life Cycle Assessment of a recovery process from End-of-Life Photovoltaic Panels. *Applied Energy* **290**, 116727 (2021).

Comment 3:

Page 5, line 128: The potential decarbonization contributions and associated strategic timing for PV manufacturing industry in China from 2015 to 2050 for STEPS and APS are shown in Fig. 3. Please define "strategic timing"? What do you mean by that term?

Page 5, line 131: For STEPS, the strategic timing, referring to the maximum potential decarbonizati

Page 8, line 213: Prior to this strategic timing, we anticipate a rising trajectory in the potential decarbonization contributions of the PV manufacturing industry primarily driven by the expanding deployment of PV systems, as the carbon intensity of PV-generated electricity is substantially lower

than that of the grid electricity. What do you mean by the statement "Prior to this strategic timing, we anticipate a rising trajectory in the potential decarbonization contributions of the PV manufacturing industry"? In which sense do you use trajectory here? Could you be more precise?

Response:

Thank you for these valuable comments. Indeed, this "strategic timing" has also been pointed out by other reviewers. In our study, strategic timing refers to the predicted time point with the highest producer decarbonization contributions by PV manufacturers. This concept of strategic timing is crucial for planning the development of the PV manufacturing industry and has two key implications before and after the timing:

1. Prior to this strategic timing, we anticipate a rising trajectory in the potential decarbonization contributions from PV manufacturers. This period is critical for maximizing the decarbonization contributions because it represents a phase where significant gains in decarbonization can be achieved through the deployment of PV systems. Therefore, during this period, supportive regulations and policies are essential to ensure rapid development of the PV manufacturing industry for accelerating energy transition.

2. After the strategic timing, we observe a decline in the decarbonization contributions from PV manufacturers as the carbon intensity of grid electricity continues to decrease. This decline aligns with your point that the decreasing avoided emissions from electricity will not impact the PV industry development. More stringent regulations and policies can be applied to the PV manufacturing industry to prevent potential excess production capacity and resource waste (IEA, 2024; Wang et al., 2022). Such actions, however, will not significantly hinder the overall energy transition, as the marginal decarbonization contributions from new PV installations will be lower.

We have now made sure that the definitions are clear and consistent throughout the manuscript and explained how it can affect the PV manufacturing industry.

Abstract:

"We also observe that the potential decarbonization contributions by China's PV manufacturers will increase until 2030–2035 and decrease later, which allows for the anticipation of the strategic timing — predicted time point with the highest producer decarbonization contributions by PV manufacturers indicating the critical time point to switch from supportive policies for PV manufacturers industry to more stringent ones to ensure success energy transition and avoid potential excess production capacity and resource waste."

Introduction:

"We then introduce strategic timing, the predicted period when the decarbonization contributions from PV manufacturers reach their maximum. This timing is critical for planning the development of the PV manufacturing industry and ensuring that supportive regulations and policies are in place to maximize contributions to the energy transition while more stringent regulations and policies can be applied to the PV manufacturing industry to prevent potential excess production capacity and resource waste."

Discussion and implications:

"Prior to this timing, we anticipate a rising trajectory in the potential decarbonization contributions

of the PV manufacturing industry primarily driven by the expanding deployment of PV systems, as the carbon intensity of PV-generated electricity is substantially lower than that of the grid electricity. During this period, supportive regulations and policies are essential to ensure the rapid development of the PV manufacturing industry for successful energy transition. After the strategic timing, we observe a decline in these contributions as the carbon intensity of grid electricity reaches a significantly low level that is very close to the PV-generated electricity. All this suggests a closing window of opportunity to secure a cleaner and sustainable energy transition, beyond which rapidly expanding deployment of PV systems may not be desirable. Instead, more stringent regulations and policies can be applied to the PV manufacturing industry to prevent potential excess production capacity and resource waste (IEA, 2024; Wang et al., 2022). Such actions will not significantly hinder the overall energy transition, as the marginal decarbonization contributions from new PV installations will be lower."

References:

Renewables 2023. (International Energy Agency, Paris, 2024).

Wang, C., Feng, K., Liu, X., Wang, P., Chen, W.-Q. & Li, J. Looming challenge of photovoltaic waste under China's solar ambition: A spatial–temporal assessment. *Applied Energy* **307**, 118186 (2022).

Comment 4:

Page 8, line 201: On a global scale, to achieve net-zero emissions for power sectors by 2050, China's PV manufacturers are anticipated to contribute 46.2%, and this figure slightly decreases to 37.6% without consideration of energy storage solutions. What do you mean by energy storage solutions? How are they considered in the decarbonization contributions?

Response:

One of the main contributions is to recognize and quantify the decarbonization contributions of upstream manufacturers in the PV industry. We also aim to demonstrate that our framework can be extended to other manufacturing industries related to the renewable energy sector, such as the energy storage industry.

Energy storage solutions, such as Lithium-ion batteries, play a critical role in the energy supply system with renewable energy (Chu, et al., 2017). In China, it is recommended to equip up to 20% of the installed renewable energy capacity with energy storage systems to enhance grid stability and manage intermittent energy supply (National Development and Reform Commission and National Energy Administration, 2021; Bullich-Massagué, 2020; China Energy Storage Alliance, 2024). In our study, we have considered energy storage systems as producers. When assessing the producer decarbonization contribution for a system that includes both PV and energy storage, the energy storage system is allocated part of the decarbonization credit. This allocation results in a slight decrease in the decarbonization contributions attributed solely to PV manufacturers.

The following texts have been updated in the revised manuscript:

Results - Strategic timing when considering industrial symbiosis' decarbonization contributions

"We assume that PV system is equipped with a lithium-ion battery energy storage system (BESS) that can store up to 20% of the installed PV capacity (China Energy Storage Alliance, 2024; Bullich-Massagué, 2020) (see Supplementary Note 5 for more details). In our study, we view energy storage systems as producers. When evaluating the decarbonization contributions of a system that includes both PV and energy storage, part of the decarbonization credit is allocated to the energy storage system."

Discussion and implications

"This study can be extended to evaluate the potential decarbonization contributions of industrial symbiosis beyond PV by recognizing their roles as producers. This includes various energy supply and storage tools in building a flexible, effective, and safe energy system. Such an approach provides a comprehensive assessment of the shared decarbonization contributions within the renewable energy system, supporting the sustainable development of renewable energy."

References:

Energy Storage Industry White Paper 2024. (China Energy Storage Alliance, 2024).

Chu, S., Cui, Y. & Liu, N. The path towards sustainable energy. *Nature Materials*, **16**, 16022 (2017).

Bullich-Massagué, E., Cifuentes-García, F., Glenny-Crende, I., et al. A review of energy storage technologies for large scale photovoltaic power plants. *Applied Energy,* **275**, 116727 (2020).

Promoting the integration of charge and storage and multi-energy complementary development of power sources (in Chinese). (National Development and Reform Commission and National Energy Administration, 2021). https://www.gov.cn/zhengce/zhengceku/2021-03/06/content_5590895.htm

Reviewer #3

The paper provides a comprehensive analysis of the decarbonization contributions of PV systems manufactured in China. In general, it addresses the timely and critical need to understand the environmental impact of renewable energy sources in the context of the energy transition, and specifically it deals with the important topic of correctly attributing carbon emissions (or emission reduction) to the different stages of value chains.

The intuition is solid, the research paper is concise, and the work is well documented. The supplementary material provides comprehensive input for the reader. Still, we see three major issues in the paper.

We thank your positive feedback immensely and feel encouraged by your detailed comments. Your acknowledgment of the importance of understanding the environmental impact of renewable energy sources and the correct attribution of carbon emissions throughout the value chains is greatly appreciated. We have carefully revised the manuscript and we believe your concerns should have been well addressed.

Comment 1:

The first one deals with the concepts of decarbonisation contributions and strategic timing. Concerning the first one, clear formulas are provided but throughout the text the reader cannot fully grasp to what the percentages refer. This should be better described. Concerning strategic timing, in the abstract we read that it is "a window of opportunity to accelerate the energy transition ambition to net-zero emissions" (lines 33-34); in the text, we read that strategic timing "refers to the maximum potential decarbonization contributions for adopting PV" (line 132). Practically, strategic timing is identified as one specific year (e.g. in line 135 it "occurs to be 2035"), but the reader does not get a clear understanding of what it ultimately is conceptually. The general concept is clear (the earlier one installs PV panels, the better it is because currently the energy system is more carbon-intensive), but what does this imply? Eventually, what is the year 2035 in the example before? What implications does it have on the actual PV deployment?

Response:

The decarbonization contributions of PV systems are determined by comparing the carbon intensity of PV-generated electricity with the average carbon intensity of the 10 different electricity generation methods supplying the grid in China over the PV system's lifespan. We then applied our proposed algorithm, based on three criteria—energy consumption, economic investment, and carbon emissions—to allocate a reasonable share of these contributions to the PV manufacturers. It is important to clarify that the percentage mentioned in the paper represents the contributions from PV manufacturers to the decarbonization efforts specifically within the PV industry. This calculation excludes other renewable energy sources. To be more precise, China's PV manufacturers could contribute 35.4%-74.9% in the Stated Policies Scenario (STEPS) and 31.4%-73.8% in the Announced Pledges Scenario (APS) of the total decarbonization contributions attributed to the PV industry. We have now revised the manuscript to clearly state that China's PV manufacturers could contribute up to 73.8% of the total decarbonization contributions by the entire PV industry towards China's targets for decarbonizing the energy sector based on the announced pledges

Below are the revised texts at the beginning of the results section defining decarbonization contributions:

"The decarbonization contributions of PV systems are determined by comparing the carbon intensity of PV-generated electricity with the average carbon intensity of 10 different electricity generation methods supplying the grid in China over the lifespan of the PV system. We utilize a dynamically changing carbon intensity for the grid, based on the projected energy structure and electricity generation capacity provided by the IEA under three different scenarios."

Below are the texts we provide clear statement for the meaning of decarbonization contributions by PV manufacturers in percentage.

Abstract:

"Results show that China's PV manufacturers could contribute up to 73.8% of the total decarbonization contributions by the entire PV industry towards China's targets for decarbonizing the energy sector based on the announced pledges."

Discussion and implications:

"Our analysis indicates that China's PV manufacturers could contribute a substantial 73.8% of the

total decarbonization contributions by the entire PV industry towards achieving China's decarbonization targets in energy transition under the APS scenario."

In our study, strategic timing refers to the predicted time point with the highest producer decarbonization contributions by PV manufacturers.

This concept is crucial for planning the development of the PV manufacturing industry and has two key implications:

1. Prior to this strategic timing, we anticipate a rising trajectory in the potential decarbonization contributions from PV manufacturers. This period is critical for maximizing the decarbonization contributions because it represents a phase where significant gains in decarbonization can be achieved through the deployment of PV systems. Therefore, during this period, supportive regulations and policies are essential to ensure rapid development of the PV manufacturing industry for accelerating energy transition.

2. After the strategic timing, we observe a decline in the decarbonization contributions from PV manufacturers as the carbon intensity of grid electricity continues to decrease. This decline aligns with your point that the decreasing avoided emissions from electricity will not impact the PV industry development. More stringent regulations and policies can be applied to the PV manufacturing industry to prevent potential excess production capacity and resource waste (IEA, 2024; Wang et al., 2022). Such actions, however, will not significantly hinder the overall energy transition, as the marginal decarbonization contributions from new PV installations will be lower.

We have now made sure that the definitions are clear and consistent throughout the manuscript and explained how it can affect PV manufacturing industry.

Abstract:

"We also observe that the potential decarbonization contributions by China's PV manufacturers will increase until 2030–2035 and decrease later, which allows for the anticipation of the strategic timing — predicted time point with the highest producer decarbonization contributions by PV manufacturers indicating the critical time point to switch from supportive policies for PV manufacturers industry to more stringent ones to ensure success energy transition and avoid potential excess production capacity and resource waste."

Introduction:

"We then introduce strategic timing, the predicted period when the decarbonization contributions from PV manufacturers reach their maximum. This timing is critical for planning the development of the PV manufacturing industry and ensuring that supportive regulations and policies are in place to maximize contributions to the energy transition while more stringent regulations and policies can be applied to the PV manufacturing industry to prevent potential excess production capacity and resource waste."

Discussion and implications:

"Prior to this timing, we anticipate a rising trajectory in the potential decarbonization contributions of the PV manufacturing industry primarily driven by the expanding deployment of PV systems, as the carbon intensity of PV-generated electricity is substantially lower than that of the grid electricity.

During this period, supportive regulations and policies are essential to ensure the rapid development of the PV manufacturing industry for successful energy transition. After the strategic timing, we observe a decline in these contributions as the carbon intensity of grid electricity reaches a significantly low level that is very close to the PV-generated electricity. All this suggests a closing window of opportunity to secure a cleaner and sustainable energy transition, beyond which rapidly expanding deployment of PV systems may not be desirable. Instead, more stringent regulations and policies can be applied to the PV manufacturing industry to prevent potential excess production capacity and resource waste (IEA, 2024; Wang et al., 2022). Such actions will not significantly hinder the overall energy transition, as the marginal decarbonization contributions from new PV installations will be lower."

References:

Renewables 2023. (International Energy Agency, Paris, 2024).

Wang, C., Feng, K., Liu, X., Wang, P., Chen, W.-Q. & Li, J. Looming challenge of photovoltaic waste under China's solar ambition: A spatial–temporal assessment. *Applied Energy* **307**, 118186 (2022).

Comment 2:

Another major issue is that mining seems to be neglected in the analysis. This is a major source of carbon emissions, so it should be considered in such a comprehensive work.

Response:

This is an important point, thanks a lot. The current Gabi database has already included the mining stages for major materials such as aluminium, iron, and copper, which are "from cradle to the gate".

We have now also included the energy consumption for mining of silica ores as suggested. We have referenced the National Standards for ore mining in China for energy consumption. Based on our calculations, a 1 MWp PV system contains approximately 2.83 ton of industrial-grade silicon, which requires around 6.84 tons of silica to be mined. The energy consumption for open-pit mining is 0.96 kg of standard coal (or 0.66 kg diesel) per ton of mineral. By including this energy consumption from mining of silica ores, the carbon emissions for the production of a 1 MWp PV system increase by 16.4 kg CO_2 eq. However, considering that the total lifecycle carbon emissions of a 1 MWp PV system are approximately 602 tons, this addition does not affect our main conclusions. Noted that the relevant data and figures have been updated accordingly, which has also incorporated the comment about transportation distances from China to other countries by Reviewer 1.

Reference:

Quota standard of mining and beneficiation production energy consumption in mining enterprise (YB / T 4417.1). (Ministry of Industry and Information Technology of the People's Republic of China, 2014).

Comment 3:

Third, the text needs to be revised from a language point of view. Apart from some normal typos (see at the end for some suggestions), it is often the case that sentences are not fully comprehensible and/or seem to have missing parts. We report a few examples in the following:

• Sentence in lines 49-50: authors likely refer to PV in this sentence, but good to specify (same in lines 146-147)

• Sentence in lines 83-85: maybe some brackets would help, e.g. "energy (characterized by the energy consumption), economy (reflected by the economic investments), and carbon emissions"

• Sentence in lines 91-93: "describes to achieve" is not very clear

• Sentence in lines 93-95: maybe "determined" should be "determine"?

• The sentence in line 100-101 should be contextualized

• The sentence in lines 109-112 does not seem very clear: at least one word is likely missing

• Line 299: "Carbon intensity of per unit electricity" is not very clear

Response:

We thank you for pointing out the language issue. We have checked the document carefully and has asked native speaker for proofreading. Regarding to the specific sentence, we have modified as follows:

- *lines 49-50: As of 2022, China has contributed 35.0% of the global cumulative PV capacity and 44% of the global newly installed PV capacity.*
- *lines 146-147: It has currently made up more than 80% of global mass PV production and is projected to lessen to 69.2%, still being dominant, by 2027.*
- *Sentence in lines 83-85: energy (characterized by the energy consumption), economy (reflected by the economic investments), and carbon emissions.*
- *lines 91-93: The Net Zero Emissions by 2050 (NZE) Scenario that shows an achievable pathway for the global power sector to achieve net-zero CO_2 emissions by 2050.*
- *lines 93-95: We then use these scenarios to quantify China's PV manufactures' potential decarbonization contributions and ultimately determine associated strategic timing.*
- *line 100-101 and 109-112: The decarbonization contributions of PV systems are determined by comparing the carbon intensity of PV-generated electricity with the average carbon intensity of 10 different electricity generation methods supplying the grid in China over the lifespan of the PV system. We utilize a dynamically changing carbon intensity for the grid, based on the projected energy structure and electricity generation capacity provided by the IEA under three different scenarios. For PV systems, we evaluate the energy consumption, economic costs, and associated emissions over the lifecycle of the PV system including manufacturing, transportation and construction, operation, and decommission stages. Our findings indicate that in China, the PV manufacturing stage (Processes 1-6 out of 9 belong to manufacturing stage) accounts for 77.8% of energy consumption, 58.7% of economic costs, and 80.7% of carbon emissions (see Fig. 1).*
- *line 299: Carbon intensity of per unit electricity production*

Comment 4:

Some additional minor points include:

• The paper often refers to the Sustainable Development Goals: these should be a bit better presented / put into context

Thanks for this valuable comment, we now put more details into context:

Introduction:

"This could further postpone global net-zero emission transition, and even the achievement of SDGs. For instance, PV industry cannot provide affordable and clean energy (SDG 7) without PV manufacturers; assessing only responsibilities but not contributions of PV manufacturers cannot accurately analyze their net environmental impact, which hinders responsible consumption and production (SDG 12); weakening efforts of emission reduction of the PV industry is a departure from the climate action (SDG 13)."

Discussion:

"Our analysis highlights the substantial decarbonization contributions of PV manufacturers throughout the world in the past and the future. About 65% of the SDG targets are highly related to action within the energy system, encompassing renewable energies like PV (Fuso Nerini et al., 2018), to achieve SDG 7. SDG 12 emphasizes reducing waste through prevention, reduction, recycling, and reuse, based on responsibility perspective. SDG 13 calls for the integration of climate change measures into national policies, strategies and planning (Geng et al., 2023). Unfortunately, the contributions of manufacturers, particularly those supporting energy transition, often go unnoticed within the SDGs. This requires more attention to renewable energy producers as a basis for low-carbon transitions in the whole supply chain. The recognition of potential decarbonization contributions from producers is therefore meant to complement the 2030 Agenda for Sustainable Development. Given that 2030 marks a milestone for both the Paris Agreement and the SDGs, the PDC framework can further serve as a valuable tool to assist policymakers in effectively monitoring global progress in energy transition and creating policy synergies."

References:

Fuso Nerini, F., Tomei, J., To, L. S., Bisaga, I., Parikh, P., Black, M. et al. Mapping synergies and trade-offs between energy and the Sustainable Development Goals. Nature Energy 3, 10-15 (2018).

Geng, T., Jia, F., Cai, W., Wu, L., Gan, B., Jing, Z. et al. Increased occurrences of consecutive La Niña events under global warming. Nature 619, 774-781 (2023).

• Line 30 (but often in other parts). We are not fully sure whether one decimal is needed in these percentages, but surely three decimals are not needed for the payback times e.g. in lines 160-162 of the Supplementary Material

Thanks for the comments, we now keep one decimal for all payback times.

• Is it 78% (line 52) or 80% (line 146)?

We have double checked the data sources and now kept it consistent as 84%.

• Line 220: "desirable" is not a great word here; of course, PV's marginal effect decreases with time as the energy system becomes less and less carbon intensive, but it would still be desirable, at the end of the day

Here, we want to reflect the potential excess production causing resource waste, which has also been pointed out by Reviewer 1. We have now changed to *"All this suggests a closing window of opportunity to secure a cleaner and sustainable energy transition, beyond which rapidly expanding deployment of PV systems may cause potential issues such as excess production capacity and resource waste."*

• Line 348: the reference discount rate is reported to be 0.1%, while in the Supplementary Material it seems to be 1% (see e.g. line 103 - which by the way is more reasonable than 0.1%), ranging from 0.1% to 5% in the sensitivity analysis. Please check.

Apologize, we used 1% to perform the calculation. In the sensitivity analysis, we did cover 0.1%-5%.

• Line 375: from the references, we see that authors adopted the IEA's 2022 edition of the WEO. That is fine, but it is not the latest now, so they should write "2022" and not "latest" here

We have now clarified the data source:

"We use the predicted electricity structure and amount from IEA World Energy Outlook 2022 …"

• Line 385: a reference to the Gabi database is reported in the Supplementary Material, but it is missing here, so it should be added

We have now added the link for Gabi in the manuscript:

"we access associated boundary data from the Gabi databases (Gabi: https://gabi.sphera.com)"

• Line 396: 2014 is quite far in the past now…

We appreciate your point regarding the use of carbon intensity values from 2014. For the carbon intensity of PV systems, we used the values calculated within our study. For other technologies, we referenced the publicly available data from the IPCC AR5's Technology-specific cost and performance parameters. Since the latest IPCC Sixth Assessment Report (AR6) does not provide updated data, we utilized the values from the Fifth Assessment Report (AR5).

Using the PV carbon intensity calculated in our study and the carbon intensities of other technologies from the IPCC AR5, combined with the energy structure data from the IEA, we calculated the carbon intensity of China's grid for 2021 to be 543 gCO_2/kWh. This value is close to the 556 gCO_2/kWh reported in the April 2024 announcement by the Ministry of Ecology and Environment and the National Bureau of Statistics of China for the carbon emission factor of

electricity in 2021. Therefore, we believe our chosen data is representative and provides a reliable basis for our analysis.

Data from the Ministry of Ecology and Environment:

https://www.mee.gov.cn/xxgk2018/xxgk/xxgk01/202404/W020240412827267102800.pdf

• Supplementary Figures 10-11-12: it is never nice if y axes start from a level different than zero, because then the proportion cannot be well captured

We have updated the three figures with y axes starting from 0.

Finally, some typos / minor language suggestions:

• Line 29: "contributions would contribute" – maybe a different formulation can be found

We have modified the texts:

"With the ambition for the global power sector to achieve net-zero emissions by 2050, China's PV manufacturers are anticipated to account for up to 45.4% of the sector's decarbonization."

• Line 92: repetition of "that"

Repetition of "that" has now been deleted.

• Line 99: "manufactures" should be "manufacturers"

Thanks.

• Line 100: "account" should be "accounts"

Corrected as suggested.

• Line 149: "higher"should be "a higher"or even better "a greater"

Here is the modified version:

"Compared to China, the global level experiences higher average solar radiation intensity, greater costs per unit of installed PV capacity, and higher electricity prices."

• Line 191: "remains to be" should be "remains"

Corrected as suggested.

• Line 211: "the three" should be "three"

We have deleted "the".

• Line 96 SM: "differ" should likely be "difference"

Thanks, we have changed to "difference".

- Line 380 SM: space before the comma

Thanks, this has been corrected.

Reviewer #4

I co-reviewed this manuscript with one of the reviewers who provided the listed reports. This is part of the Nature Communications initiative to facilitate training in peer review and to provide appropriate recognition for Early Career Researchers who co-review manuscripts.

We appreciate your contribution in reviewing our manuscript as a co-reviewer. We have addressed all the comments proposed by Reviewer #1 and other reviewers. Your collaborative effort and insights have certainly facilitated the improvement of our manuscript. We hope that you like the revised version.

Authors' Responses to Review Comments

Journal:	Nature Communications
Manuscript #:	NCOMMS-24-13270A
Title of Paper:	Strategic Timing for the Decarbonization Contributions by China's Photovoltaic Manufacturers in Global Energy Transition
Authors:	Qingyang Lin, Kai Wang, Chenglin Li, Kai Fang, Meixuan Xin, Zhenci Xu, Yue Lu, Siqi Wang, Anqi Xu, Xiang Gao
Article Type:	Research paper

We appreciate the time and efforts taken by the Reviewers and Editors in reviewing our revised manuscript. We are encouraged that our efforts in improving the manuscript have been recognized. We have carefully addressed the remaining comments point by point with detailed responses as follows, whereby the manuscript has been substantially revised. As we have rewrote many sections of the manuscript and restructured the entire document, we believe that it does not make sense to provide a track-change version. Instead, we highlight in yellow the major changes made to the manuscript. We hope that the revised manuscript satisfies the journal's standard. If not, however, we are happy to work further to make the paper acceptable for publication in *Nature Communications*.

Reviewer #1

The authors haves made large efforts to revise the manuscript, and have addressed most of my questions. I still have some little suggestions as follows.

We appreciate your helpful and detailed comments on our manuscript and feel encouraged by your positive feedbacks on our revisions. The manuscript has now been carefully revised in response to your remaining concerns. We hope that you like the revised version.

Comment 1:

For previous Comment 5, Dual Carbon Reduction Strategy maybe the potential adverse effects in the short term, but from the perspective of long term, it will prompt the technology transition of the PV industry.

Response:

We fully agree that the Dual Carbon Reduction Strategy will promote the technology transition of the PV industry from the perspective of long term. In our study, we choose to focus on the strict policies, particularly those in short term that may make upstream manufacturers face potential disruptions, such as difficulties in capacity expansion and increased product costs. This could ultimately hinder the overall development of the PV industry and energy transition in the long term. By determining the decarbonization contributions of PV manufacturers, we aim to highlight that these manufacturers, despite their high energy consumption and carbon emissions, lay a foundation for the PV industry. As such, they need policy support, particularly in the short term, to ensure the sustainable development of the entire PV industry. This would also make substantial contributions to the global energy transition in the long term.

Comment 2:

In addition, more systematic policy consideration from the whole lifecycle time should be advised to the policy makers.

We find that there is a time window for the implementation of the policy, as reflected by the calculated strategic timing—the predicted time point that PV manufacturers reach their maximum contributions. Prior to this timing, supportive regulations and policies should be in place to maximize contributions to energy transition. Once this strategic timing has passed and the decarbonization contributions of PV manufacturers begin to decline, more stringent regulations and policies would be required to prevent potential issues such as overproduction and resource waste within the PV industry.

Specifically, the strategic timing is projected to occur in 2035 for China to achieve net-zero goal at the national level. And a five-year extended support for PV manufacturers may be necessary to sustain the decarbonization contributions by PV manufacturers since the strategic timing occurs later than the China's 2030 peak emissions goal. Moreover, a shift in policy focus from the global market to China's domestic market is needed between 2030 and 2035 to avoid potential PV overproduction at the global level.

We have now revised the text as follows.

Abstract: "*Our further analysis highlights a few key points: (i) a strategic timing—the predicted time point that PV manufacturers reach their maximum contributions—is projected to occur in 2035 for China to achieve net-zero goal at the national level; (ii) a five-year extended support for PV manufacturers may be necessary to sustain the decarbonization contributions by PV manufacturers since the strategic timing occurs later than China's 2030 peak emissions goal; and (iii) a shift in policy focus from the global market to China's domestic market is needed between 2030 and 2035 to avoid potential PV overproduction at the global level. The proposed PDC framework that provides a first measure of the overlooked contributions of the PV manufacturing industry to both China's and global decarbonization progresses can be extended to other renewables to inform policymakers on energy transition.*"

Introduction: *"While the implementation of this strategy is crucial for addressing long-term environmental goals by prompting technological transitions, it, however, may pose substantial short-term challenges to various industries, including the PV manufacturing industry, as they normally produce most carbon emissions within a product's entire lifespan (Khawaja et al., 2021; Jenkins et al., 2023; Heath et al., 2020)"*

"Although China's carbon reduction goals will prompt the technology transition of the PV industry from the perspective of long term, the strict policies, particularly in short term, could still make upstream manufacturers face potential disruptions, difficulties in capacity expansion, and increased product costs (Helveston et al., 2022; Kozul-Wright, 2023). This could ultimately hinder the overall development of the PV industry and energy transition (Ou et al., 2024)."

Discussion and implications: *"Moreover, our analysis indicates that the strategic timing under the NZE scenario is projected to occur in 2030, five years earlier than 2035 timing under the APS scenario in China. As China has been playing a leading role in the world's PV production, its potential overproduction is of particular concern (International Energy Agency, Paris, 2024; Wang*

et al., 2022; Schlömer et al., 2014). While our analysis demonstrates the substantial decarbonization contributions by China's PV manufacturers at both national and global levels, we argue, on the other hand, that between 2030 and 2035 a shift in policy focus from the global market to China's domestic market is needed to avoid potential PV overproduction at the global level (Yu et al., 2021). After 2035 – the strategic timing in China under APS, the decarbonization contributions from China's PV manufacturers would begin to decline; therefore, more stringent regulations should be implemented to China's PV manufacturers to avoid potential issues such as excess production capacity."

References:

Heath, G. A., Silverman, T. J., Kempe, M., Deceglie, M., Ravikumar, D., Remo, T. et al. Research and development priorities for silicon photovoltaic module recycling to support a circular economy. Nature Energy 5, 502-510 (2020).

Helveston, J. P., He, G. & Davidson, M. R. Quantifying the cost savings of global solar photovoltaic supply chains. Nature 612, 83-87 (2022).

Jenkins, S., Kuijper, M., Helferty, H., Girardin, C. & Allen, M. Extended producer responsibility for fossil fuels. Environmental Research Letters 18, 011005 (2023

Khawaja, M. K., Ghaith, M. & Alkhalidi, A. Public-private partnership versus extended producer responsibility for end-of-life of photovoltaic modules management policy. Solar Energy 222, 193-201 (2021).

Kozul-Wright, R. China's policy strategies for green low-carbon development: Perspective from South-South cooperation. United Nations Conference on Trade and Development (UNCTD), (2023).

Ou, Y., Hsiao, C. Y.-L. & Chui, C. M. How does the supply chain market respond to policy shocks? Evidence from solar photovoltaic sectors in China. Renewable Energy 232 (2024).

IEA. Renewables 2023. International Energy Agency (IEA), Paris (2024).

Schlömer S., T. B., L. Fulton, E. Hertwich, A. McKinnon, D. Perczyk, J. Roy, R. Schaeffer, R. Sims, P. Smith, and R. Wiser. Technology-specific cost and performance parameters. Intergovernmental Panel on Climate Change (IPCC), Cambridge University Press (2014).

Wang, C., Feng, K., Liu, X., Wang, P., Chen, W.-Q. & Li, J. Looming challenge of photovoltaic waste under China's solar ambition: A spatial–temporal assessment. Applied Energy 307, 118186 (2022).

Yu, S., Lu, T., Hu, X., Liu, L. & Wei, Y.-M. Determinants of overcapacity in China's renewable energy industry: Evidence from wind, photovoltaic, and biomass energy enterprises. Energy Economics 97 (2021).

Comment 3:

It is suggested that the research limitation and uncertainty (like my previous comments) should be discussed in your study.

Response:

Thank you for your valuable comment. We have followed your suggestion to add a discussion of

limitations to the Methods section as follows:

*"**Limitations.** As a first exploration of the decarbonization contributions by China's PV in global energy transition, our study remains some limitations that should be addressed in future work. For the reason of simplicity, we assume that all electricity generated by PV systems can be entirely integrated into the national grid. Moreover, our study focuses exclusively on PV and BESS, although it can be easily extended to other renewables, such as wind power, with minor changes to the current PDC framework. In that sense, decarbonization contributions should consider not only PV manufacturers but also all other manufactures across the entire power supply chain, with contributions assigned accordingly. This broader perspective is nevertheless beyond the scope of this study and warrants a follow-up analysis using the PDC framework developed in this work."*

Reviewer #2

The paper has been improved according to the reviewers' comments and has gained a lot in clarity. It, therefore, warrants for publication.

Many thanks for your positive comments. Your interpretation of this paper is exactly the way we intended. We hope that you like the revised version.

Reviewer #3

We appreciate the efforts made by the authors to address the issues raised in our review, and in those carried out by our colleagues (which were coherent with ours). Explanations provided are thorough and extensive. However, we are afraid that the authors did not clarify the main concern about the core of the paper, i.e. how the decarbonisation contribution and the strategic timing are conceived and calculated. Through the long explanations and descriptions, we fail to understand how these factors are actually calculated, how you can eventually say that e.g. "China's PV manufacturers could contribute up to 73.8% of the total decarbonization contributions". It may well be our own limitation, but all in all – although we still see value in the intuition of the paper (you cannot give full honour for decarbonisation to utilities installing PV modules and full blame to the manufacturers) – we must suggest that the paper should be rejected.

Response:

First of all, we are sorry for the ambiguity in defining and quantifying the decarbonisation contributions and the strategic timing. We value a lot your constructive feedbacks and have substantially revised the manuscript by making concrete changes to the text. We believe that all the points have been addressed in the new version of the manuscript. We hope you like it.

To be clear, our responses will be organized in the following order: 1) detailed steps on how the decarbonization contributions are calculated; 2) clarification of the proportions of decarbonization contributions; and 3) clarification of the strategic timing for PV manufacturers.

1) Detailed Steps on How the Decarbonization Contributions are Calculated

- **Step 1:** We first calculate carbon intensity per unit electricity production for a PV system over the lifetime. The carbon emissions are measured over the four stages (manufacturing,

transportation and construction, operation, and decommissioning) using a 1 MWp PV system on carbon emissions and decarbonization contributions over a 30-year period using manufacturing performance data in China in 2022 (Equations 1-2). The total amount of electricity over the lifecycle is measured considering the installed capacity of PV system, annual peak sunshine hours, performance ratio, average loss coefficients of electricity transmission, etc. (Equation 3). The carbon intensity per unit electricity production for a PV system is determined as 16.94 gCO_2 eq/kWh (Equation 4).

---See **"Lifecycle carbon intensity per unit electricity production for a PV system" in the Methods section** and Supplementary Note 2 for source data.

- **Step 2:** We determine the total decarbonization contributions of PV systems, which is the sum of reduced carbon emissions by PV-generated electricity replacing grid electricity (Equation 5). We utilize a dynamically changing carbon intensity for the grid, as projected by the IEA under different scenarios, including STEPS, APS and NZE. For each year (t), the carbon intensity for the grid can be measured using Equation 6. The energy structure for the grid includes ten different electricity generation methods.

  ---See **"Total decarbonization contributions of PV systems" in the Methods section** and Supplementary Notes 3, 4 and 5 for detailed source data.

- **Step 3:** We then divide the total decarbonization contributions into unassigned and assigned contributions under each criterion (energy consumption, economic costs, or carbon emissions) and allocate them over the lifecycle. The assigned contribution that can be allocated to PV system across for stages refers to the cumulative decarbonization contributions after the payback time for each criterion is reached until end of the lifetime. The payback time for energy consumption, economic costs, or carbon emissions are defined as T_{en} (Equation 7), T_{eco} (Equation 8) and T_{em}, (Equation 9), respectively.

  ---See **"Payback time from the three criteria" in the Methods section** and Supplementary Note 3 for detailed source data.

- **Step 4:** The assigned decarbonization contributions of the PV industry under each criterion can then be calculated by adding up the decarbonization contributions between each payback time and the end of the lifecycle using Equation 10.

  ---See **"Assigned decarbonization contributions after payback time" in the Methods section**.

- **Step 5:** When determining how much decarbonization contributions should be allocated to manufacturers, we first divide the entire lifecycle consisting of four stages into 9 processes. By accounting for the energy consumptions, economic costs and carbon emissions for individual processes, we obtain the corresponding proportion values for each process under each criterion (Equations 11-12, Fig. 4). The decarbonization contributions for manufacturing stage can be measured (Equation 12).

  ---See **"Decarbonization contributions for manufacturers within the PV industry" in the Methods section**.

The Methods section has been re-organized in such a way that it corresponds to the logic of the five steps provided above. We have added Fig. 6 to visualizes this logic.

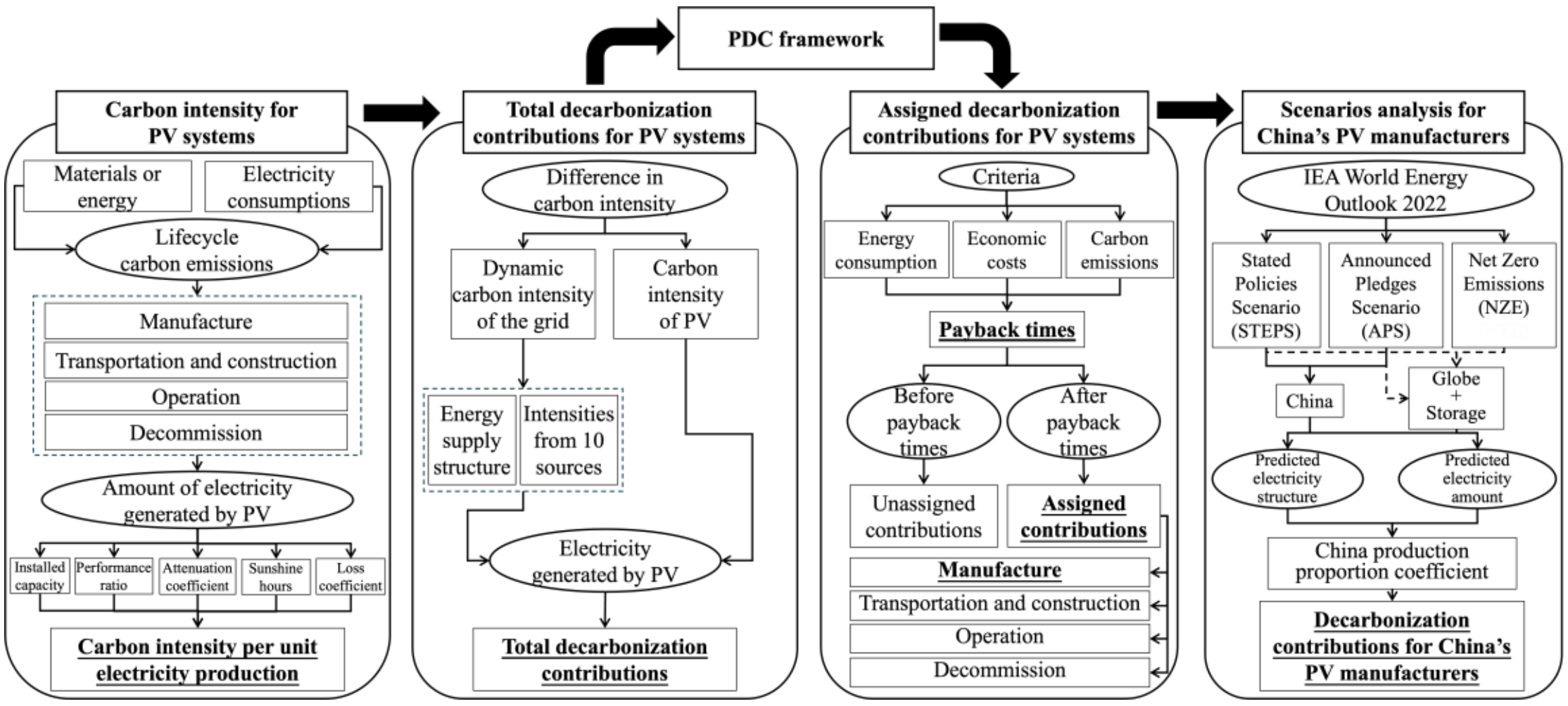

Fig. 6 | Schematic diagram of the methodological framework determining the decarbonization contributions for China's PV manufacturers under different scenarios and criteria.

2) Clarification of Proportions of Decarbonization Contributions

When it comes to the proportion of China's PV manufacturers in total PV's decarbonization contributions under the three criteria, the value for the economic costs criterion is much lower than that for energy consumption and carbon emissions. To avoid potential misunderstandings about the decarbonization percentages, particularly under the emission criterion, we have made use of numerical ranges throughout the manuscript to clarify China's PV manufacturers' contributions under each criterion. For example, *"China's PV manufacturers could contribute **31.4%-73.8%** of the total PV decarbonization in China's energy transition under pledged targets, and **27.9%-45.4%** globally without consideration of energy storage integrated."* We have added Table 1 to the text to compare the proportions of total PV's decarbonization contributions at four stages of the lifecycle under different assessment criteria and scenarios.

Table 1 | Proportions of total PV's decarbonization contributions at four stages of the lifecycle under different assessment criteria and scenarios.

**China-STEPS**							
**Criteria**	Manufacturing	Transportation and construction	Operation	Decommission	Unassigned	Sum	China's PV manufacture*
Energy	66.5%	9.9%	0.1%	8.9%	14.6%	100%	66.5%
Economy	35.4%	11.3%	7.2%	6.4%	39.8%	100%	35.4%
Emissions	74.9%	9.3%	0.1%	8.5%	7.2%	100%	74.9%
**China-APS**							
**Criteria**	Manufacturing	Transportation and construction	Operation	Decommission	Unassigned	Sum	China's PV manufacture*
Energy	64.4%	9.6%	0.1%	8.7%	17.2%	100%	64.4%
Economy	31.4%	10.0%	6.4%	5.7%	46.5%	100%	31.4%
Emissions	73.8%	9.2%	0.1%	8.4%	8.54%	100%	73.8%
**Global-STEPS**							
**Criteria**	Manufacturing	Transportation and construction	Operation	Decommission	Unassigned	Sum	China's PV manufacture*
Energy	65.6%	10.9%	0.1%	8.8%	14.6%	100%	45.4%
Economy	51.1%	16.3%	7.1%	6.4%	19.2%	100%	35.3%
Emissions	72.5%	10.3%	0.1%	8.3%	8.8%	100%	50.2%
**Global-APS**							
**Criteria**	Manufacturing	Transportation	Operation	Decommission	Unassigned	Sum	China's PV

		and construction					manufacture*
Energy	62.7%	10.4%	0.1%	8.4%	18.4%	100%	43.4%
Economy	48.0%	15.3%	6.7%	6.0%	24.0%	100%	33.2%
Emissions	70.6%	10.0%	0.1%	8.0%	11.2%	100%	48.9%
**Global-NZE**							
**Criteria**	Manufacturing	Transportation and construction	Operation	Decommission	Unassigned	Sum	China's PV manufacture*
Energy	55.3%	9.2%	0.1%	7.4%	27.9%	100%	38.3%
Economy	40.4%	12.9%	5.6%	5.1%	36.1%	100%	27.9%
Emissions	65.6%	9.3%	0.1%	7.5%	17.5%	100%	45.4%
**Global-Storage-NZE**							
**Criteria**	Manufacturing	Transportation and construction	Operation	Decommission	Storage and Unassigned	Sum	China's PV manufacture*
Energy	53.4%	8.3%	0.1%	6.6%	10.3% + 21.2%	100%	37.0%
Economy	27.6%	8.8%	5.6%	5.1%	10.5% + 42.3%	100%	19.1%
Emissions	53.1%	8.1%	0.1%	6.5%	3.8% + 28.4%	100%	36.8%

3) Clarification of Strategic Timing for PV Manufacturers

This comment aligns with Reviewer 1's comment. Here we define a strategic timing as the predicted time point that PV manufacturers reach their maximum contributions, with the aim of assisting policymakers in creating systematic and balanced policies over the lifecycle time of PV system. In principle, prior to this timing, supportive regulations and policies should be in place to maximize PV's contributions to energy transition. Once this strategic timing has passed and the decarbonization contributions of PV manufacturers begin to decline, more stringent regulations and policies would be required to prevent potential issues such as overproduction and resource waste within the PV industry.

Our analysis demonstrates that the strategic timing is projected to occur in 2035 for China to achieve net-zero goal at the national level. And a five-year extended support for PV manufacturers may be necessary to sustain the decarbonization contributions by PV manufacturers since the strategic timing occurs later than the China's 2030 peak emissions goal. Moreover, a shift in policy focus from the global market to China's domestic market is needed between 2030 and 2035 to avoid potential PV overproduction at the global level. We have modified the manuscript as follows.

Abstract: *"Results show that China's PV manufacturers could contribute 31.4%-73.8% of the total PV decarbonization in China's energy transition under pledged targets, and 27.9%-45.4% globally without consideration of energy storage integrated, justifying policy support to maximize these decarbonization contributions. Our further analysis highlights a few key points: (i) a strategic timing—the predicted time point that PV manufacturers reach their maximum contributions—is projected to occur in 2035 for China to achieve net-zero goal at the national level; (ii) a five-year extended support for PV manufacturers may be necessary to sustain the decarbonization contributions by PV manufacturers since the strategic timing occurs later than China's 2030 peak emissions goal; and (iii) a shift in policy focus from the global market to China's domestic market is needed between 2030 and 2035 to avoid potential PV overproduction at the global level. The proposed PDC framework that provides a first measure of the overlooked contributions of the PV manufacturing industry to both China's and global decarbonization progresses can be extended to other renewables to inform policymakers on energy transition"*

Rewrite part of results sections: To improve the readability of the paper, we have re-written the Results section to ensure the key findings are clearly presented and the definition and measurement

of the strategic timing can be easily understood and verified.

Discussion and implications: *"…we provide a more integrated view of the lifecycle contributions of PV systems; that is, China's PV manufacturers could contribute a 31.4%-73.8% of the total PV decarbonization towards achieving China's decarbonization goals in energy transition under the APS scenario at the strategic timing of 2035. At the global level, under the NZE scenario to achieve net-zero emissions for power sector by 2050, China's PV manufacturers are anticipated to contribute 27.9%-45.4% and this figure slightly decreases to 19.1%-37.0% without consideration of energy storage solutions at the strategic timing of 2030. Table 1 summarizes the proportions of total PV's decarbonization contributions across four stages of the lifecycle under different assessment criteria and scenarios"*

"Moreover, our analysis indicates that the strategic timing under the NZE scenario is projected to occur in 2030, five years earlier than 2035 timing under the APS scenario in China. As China has been playing a leading role in the world's PV production, its potential overproduction is of particular concern (International Energy Agency, Paris, 2024; Wang et al., 2022; Schlömer et al., 2014). While our analysis demonstrates the substantial decarbonization contributions by China's PV manufacturers at both national and global levels, we argue, on the other hand, that between 2030 and 2035 a shift in policy focus from the global market to China's domestic market is needed to avoid potential PV overproduction at the global level (Yu et al., 2021). After 2035 – the strategic timing in China under APS, the decarbonization contributions from China's PV manufacturers would begin to decline; therefore, more stringent regulations should be implemented to China's PV manufacturers to avoid potential issues such as excess production capacity."

References:

IEA. Renewables 2023. International Energy Agency (IEA), Paris (2024).

Schlömer S., T. B., L. Fulton, E. Hertwich, A. McKinnon, D. Perczyk, J. Roy, R. Schaeffer, R. Sims, P. Smith, and R. Wiser. Technology-specific cost and performance parameters. Intergovernmental Panel on Climate Change (IPCC), Cambridge University Press (2014).

Shiraishi K, Park W Y, Kammen D M. The role of hydrogen as long-duration energy storage and as an international energy carrier for electricity sector decarbonization. Environmental Research Letters, 2024, 19(8): 084011.

Sun L L, Cui H J, Ge Q S. Will China achieve its 2060 carbon neutral commitment from the provincial perspective? Advances in Climate Change Research, 2022, 13(2): 169-178.

Wang J, Feng Y, He, Y Q. Insights for China from EU management of recycling end-of-life photovoltaic modules. Solar Energy, 2024, 273: 112532.

Wang, C., Feng, K., Liu, X., Wang, P., Chen, W.-Q. & Li, J. Looming challenge of photovoltaic waste under China's solar ambition: A spatial–temporal assessment. Applied Energy 307, 118186 (2022).

Yu, S., Lu, T., Hu, X., Liu, L. & Wei, Y.-M. Determinants of overcapacity in China's renewable energy industry: Evidence from wind, photovoltaic, and biomass energy enterprises. Energy Economics 97 (2021).

Reviewer #4

I co-reviewed this manuscript with one of the reviewers who provided the listed reports. This is part of the Nature Communications initiative to facilitate training in peer review and to provide appropriate recognition for Early Career Researchers who co-review manuscripts.

We appreciate your contribution in reviewing our manuscript as a co-reviewer. We have addressed all the remaining comments proposed by the reviewers. We hope that you like the revised version.

Authors' Responses to Review Comments

Journal:	Nature Communications
Manuscript #:	NCOMMS-24-13270B
Title of Paper:	Strategic Timing for the Decarbonization Contributions by China's Photovoltaic Manufacturers in Global Energy Transition
Article Type:	Research paper

We appreciate the time and efforts taken by the Reviewers and Editors in reviewing our revised manuscript. We have carefully addressed all the comments point by point, with detailed responses provided below and tracked changes in this thoroughly revised version of the manuscript. We hope that the now the manuscript satisfies the journal's standards. If not, however, we are happy to work further to make the paper acceptable for publication in *Nature Communications*.

Reviewer #3

Comment 1:

The authors do have a valid point in that PV manufacturers are essential for the clean energy transition, and they should not be unfairly penalised for their emissions while power companies get all the credit. We fully support this intuition. But their method of reallocating decarbonisation benefits is a stretch. Somehow, the logic is: "We create emissions so that emissions can be avoided later – therefore, we are the main drivers of decarbonisation." But that does not follow because the true driver of decarbonisation is deployment, not just production. No installation, no decarbonisation. In this perspective, the argument downplays the role of policymakers and utilities: the decision to install and use PV is made by energy providers and consumers, not manufacturers. If PV demand dropped, manufacturing would not reduce emissions on its own.

Response:

Thanks for your concerns. We agree with the reviewer that "no deployment means no decarbonization". In this study, projections of PV deployment determine the scale of production and, in turn, the overall system-wide decarbonization contributions. Exactly for this reason, in our methodology we account for the total decarbonization contributions of the entire PV industry based on the deployment projections from the

IEA both at the national and global scales. To avoid ambiguity, we have removed the statement that may lead to some misunderstanding. Now the text reads as:

Introduction: "*Our analysis takes into account future electricity production structure and deployment of PV under three scenarios released by the International Energy Agency (IEA) (IEA, 2022).*"

Furthermore, the differences across data sources (e.g., IEA, IPCC) and publication years may lead to variations in the amount of electricity generated by PV, which somehow affect the total decarbonization contributions of PV at the system level. However, we focus on the relative share of decarbonization contributions attributed to PV manufacturers in China and on identifying the strategic timing with policy relevance. These relative shares and the associated strategic timing would not be influenced by the absolute amount of PV-generated electricity. Nonetheless, when quantifying the absolute decarbonization contributions, the most up-to-date datasets from various sources should be considered.

We have added a statement in the Data sources section:

Data sources: "*The electricity structure data for the three scenarios (STEPS, APS, and NZE) are obtained from the World Energy Outlook 2022. While differences across the data sources and publication years may affect the absolute values of PV-generated electricity, our analysis focuses on the relative share of decarbonization contributions from PV manufacturers and the associated strategic timing, both of which remain robust across datasets. For estimating the absolute decarbonization contributions, however, the most up-to-date data are preferred.*"

References:

1. IEA. World Energy Outlook 2022. International Energy Agency (IEA), Paris (2022).
2. Jiang, M., Gao, X., Guan, Q., Hao, X., An, F. The structural roles of sectors and their contributions to global carbon emissions: A complex network perspective. Journal of Cleaner Production, 208, 426-435 (2019).
3. Liu, D., Liu, J., Wang, S., Xu, M. & Akbar, S. J. Contribution of international photovoltaic trade to global greenhouse gas emission reduction: the example of China. Resources, Conservation and Recycling 143, 114-118 (2019).
4. Suvajit, B., Muhammad, A. K., Muhammad I. H. Searching appropriate system boundary for accounting India's emission inventory for the responsibility to reduce carbon emissions. Journal of Environmental Management, 295, 112907 (2021).

Comment 2:

But even forgetting this point on deployment, the main objection remains: we support the idea of finding a criterion not to penalise manufacturers, but the following step so that they become the main contributors to decarbonisation only because originally they are the main emitters is not convincing.

Having said that, we very much respect the work of the authors, and we do not wish to further stress them out with additional discussion.

All in all, we leave the decision to the Editor. But thank you again for allowing us to reflect on this important point and for the valuable exchanges.

Response:

As also agreed by all the reviewers in the past rounds of reviews, recognizing manufacturers' contributions to decarbonization deserves are highly worthy of discussion in addition to the emission responsibility, which has already been widely discussed in the literature. In this study, we were not intended to equate more emissions with more decarbonization contributions. Instead, we aim to propose a framework that allocates a fair share of decarbonization contributions to the PV manufacturers using three criteria: energy (characterized by the energy consumption), economy (characterized by the economic costs), and carbon emissions, respectively. They capture the core dimensions of resource use, economic viability and environmental impact, respectively. The share of decarbonization contributions of PV manufacturers could vary across different criteria. For instance, for China under APS scenario, the relative contributions may differ by more than 50%, with a notably decline observed when shifting from the emissions criterion to the economy criterion (Table 1). We later show that the strategic timing is consistent (Figures 1 and 2), and this furthermore validates the robustness and reliability of our results. For instance, the strategic timing is projected to occur in 2035 for China to achieve net-zero goal at the national level, despite variations in the allocation criteria.

We acknowledge that fairly assessing the role of manufacturing in decarbonization is inherently complex and shaped by many interrelated factors. We do not expect our proposed framework allocating decarbonization contributions to manufacturers to be the only and final solution, but rather a starting point that could lead to a broader discussion within the research community. We would be pleased to see our framework inspire further refinements and alternative approaches proposed by other researchers to

benefit not only PV industries but also other renewables industries for a sustainable future.

We have now revised the text as follows.

Abstract: "*Our further analysis highlights a few key points: (i) a strategic timing—the predicted time point that PV manufacturers reach their maximum contributions—is projected to occur in 2035 for China to achieve net-zero goal at the national level, despite variations in the allocation criteria; (ii) a five-year extended support for PV manufacturers may be necessary to sustain the decarbonization contributions by PV manufacturers since the strategic timing occurs later than China's 2030 peak emissions goal; and (iii) a shift in policy focus from the global market to China's domestic market is needed when the world has reached its strategic timing, while China has not yet, so as to avoid potential PV overproduction at the global level.*"

Table 1 | Proportions of total PV' s decarbonization contributions at four stages of the lifecycle under different assessment criteria and scenarios.

**China-STEPS**							
**Criteria**	Manufacturing	Transportation and construction	Operation	Decommission	Unassigned	Sum	China's PV manufacture*
Energy	66.5%	9.9%	0.1%	8.9%	14.6%	100%	66.5%
Economy	35.4%	11.3%	7.2%	6.4%	39.8%	100%	35.4%
Emissions	74.9%	9.3%	0.1%	8.5%	7.2%	100%	74.9%
**China-APS**							
**Criteria**	Manufacturing	Transportation and construction	Operation	Decommission	Unassigned	Sum	China's PV manufacture*
Energy	64.4%	9.6%	0.1%	8.7%	17.2%	100%	64.4%
Economy	31.4%	10.0%	6.4%	5.7%	46.5%	100%	31.4%
Emissions	73.8%	9.2%	0.1%	8.4%	8.54%	100%	73.8%
**Global-STEPS**							
**Criteria**	Manufacturing	Transportation and construction	Operation	Decommission	Unassigned	Sum	China's PV manufacture*
Energy	65.6%	10.9%	0.1%	8.8%	14.6%	100%	45.4%
Economy	51.1%	16.3%	7.1%	6.4%	19.2%	100%	35.3%
Emissions	72.5%	10.3%	0.1%	8.3%	8.8%	100%	50.2%
**Global-APS**							
**Criteria**	Manufacturing	Transportation and construction	Operation	Decommission	Unassigned	Sum	China's PV manufacture*
Energy	62.7%	10.4%	0.1%	8.4%	18.4%	100%	43.4%
Economy	48.0%	15.3%	6.7%	6.0%	24.0%	100%	33.2%
Emissions	70.6%	10.0%	0.1%	8.0%	11.2%	100%	48.9%
**Global-NZE**							

**Criteria**	Manufacturing	Transportation and construction	Operation	Decommission	Unassigned	Sum	China's PV manufacture*
Energy	55.3%	9.2%	0.1%	7.4%	27.9%	100%	38.3%
Economy	40.4%	12.9%	5.6%	5.1%	36.1%	100%	27.9%
Emissions	65.6%	9.3%	0.1%	7.5%	17.5%	100%	45.4%
**Global-Storage-NZE**							
**Criteria**	Manufacturing	Transportation and construction	Operation	Decommission	Storage and Unassigned	Sum	China's PV manufacture*
Energy	53.4%	8.3%	0.1%	6.6%	10.3% + 21.2%	100%	37.0%
Economy	27.6%	8.8%	5.6%	5.1%	10.5% + 42.3%	100%	19.1%
Emissions	53.1%	8.1%	0.1%	6.5%	3.8% + 28.4%	100%	36.8%

* We multiply the predicted China's PV production share by the share of manufacturing stage. In China, the share value is set to be 1; at the global level, the share value is set to be 69.2% based on the prediction (IEA, 2022).

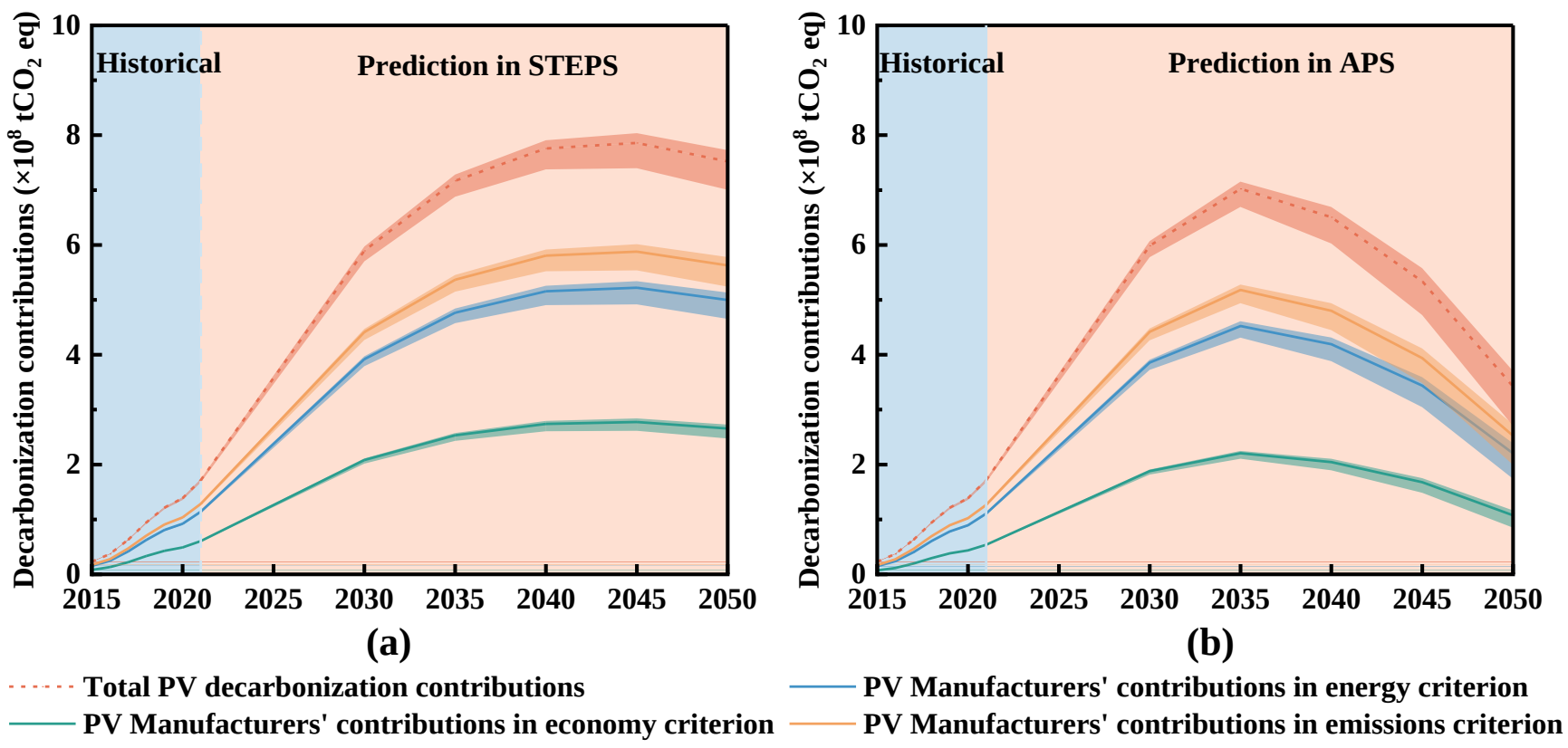

Fig. 1 | The decarbonization contributions and associated strategic timing of the PV manufacturers at China's national level: (a) STEPS and (b) APS.

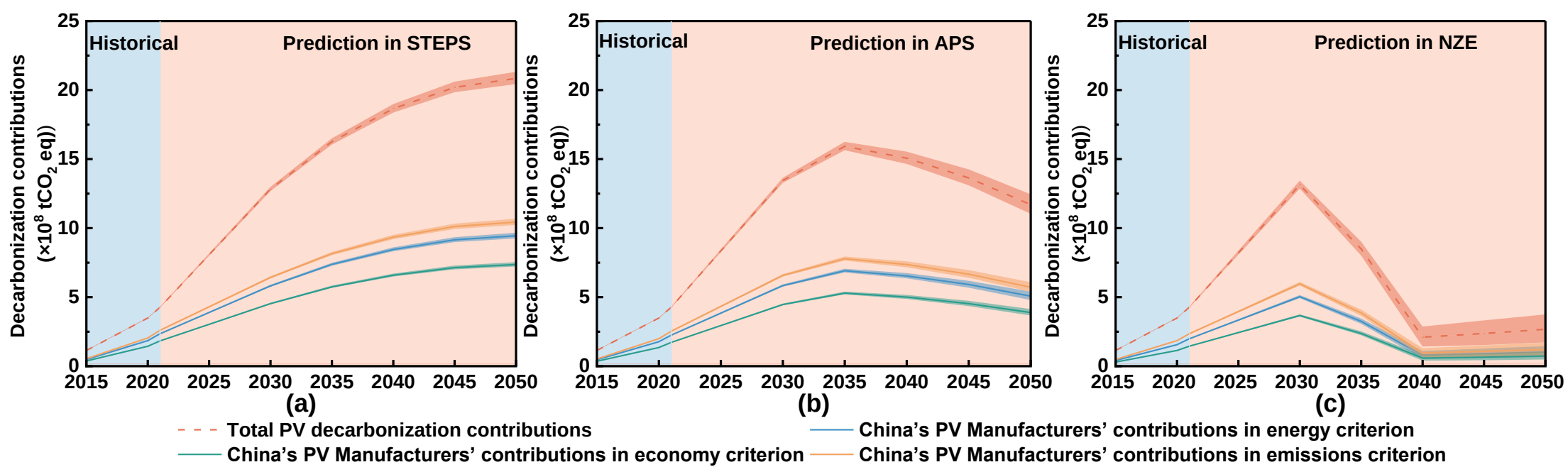

Fig. 2 | The decarbonization contributions and associated strategic timing of China's PV manufactures at the global level: (a) STEPS, (b) APS and (c) NZE.

Reviewer #4

I co-reviewed this manuscript with one of the reviewers who provided the listed reports. This is part of the Nature Communications initiative to facilitate training in peer review and to provide appropriate recognition for Early Career Researchers who co-review manuscripts.

Response:

We appreciate your contributions in reviewing our manuscript as a co-reviewer. We have addressed all the remaining comments proposed by the reviewers. We hope that you like the revised version.

Reviewer #5

Thank you for the submission of this interesting paper. The concept to assign decarbonization benefits to the manufacturers of RES systems is of interest.

There are a few aspects, which are not yet clear to me.

Comment 1:

Why only IEA 2022 scenarios, which are not very ambitious? (WEO 2024 with different numbers is available). IPCC AR6 lists much more ambitious scenarios including 100% RES ones.

Response:

Thanks for this insightful comment. Our study was originally submitted in early 2024, approximately ten months before the release of WEO 2024. Nonetheless, we have followed your suggestion to compare key indicators between WEO 2022 and WEO 2024. We see no significant discrepancies between these two versions. For example, the share of renewables in total primary energy was projected to be 70.07% in WEO 2024 versus 70.80% in WEO 2022. The share of solar PV is 23.23% in WEO 2024 and 25.75% in WEO 2022. These differences—within 5 percentage points—suggest that the overall projections remain consistent across both versions.

The electricity structure data for the three scenarios (STEPS, APS, and NZE) from 2030 to 2050 are obtained from the World Energy Outlook 2022 and its extended database by the IEA. We acknowledge that the differences across data sources (e.g., IEA, IPCC) and publication years may lead to variations in the absolute values of PV-generated electricity, which somehow affect the total decarbonization contributions of PV at the system level. However, we focus on assessing the relative share of decarbonization

contributions attributed to PV manufacturers in China and on identifying the strategic timing with policy relevance. These relative shares and the associated strategic timing would not be influenced by the absolute amount of PV-generated electricity. Nonetheless, when quantifying the absolute decarbonization contributions, the most up-to-date datasets from various sources should be considered.

We have added a statement in the Data sources section:

Data sources: "*The electricity structure data for the three scenarios (STEPS, APS, and NZE) are obtained from the World Energy Outlook 2022. While differences across the data sources and publication years may affect the absolute values of PV-generated electricity, our analysis focuses on the relative share of decarbonization contributions from PV manufacturers and the associated strategic timing, both of which remain robust across datasets. For estimating the absolute decarbonization contributions, however, the most up-to-date data are preferred.*"

Supplementary information: *"We have also compared this database with the recently released WEO 2024 (IEA, 2024) [https://www.iea.org/reports/world-energy-outlook-2024]. We find that the fractional contributions from PV manufacturers and the associated strategic timing remain consistent, thereby indicating that the core policy implications presented in this study are robust."*

Reference:

1. IEA. World Energy Outlook 2024. International Energy Agency (IEA), Paris (2024).

Comment 2:

Which parts of the PV system value chain are considered and which share is attributed to the different production steps? E.g. mining, polysilicon production, wafer, solar cells, solar modules including contact materials, glass and polymers, BOS, installation. The reason for this is that not all steps must be done in China.

Response:

Thank you for raising this important point regarding the scope of our life cycle assessment. The carbon emission coefficients for PV manufacturing obtained from the GaBi database (https://gabi.sphera.com) provides cradle-to-gate emission factors for key materials and energy inputs across the production chain—including electricity, aluminum, steel, and polymers. The analysis encompasses upstream and midstream

processes such as mining, polysilicon production, wafer and cell fabrication, module assembly (including encapsulation, contact materials, and PV glass), and balance-of-system (BOS) components. Supplementary Table 15 provides a full inventory of the components included in the life cycle boundary.

We acknowledge that certain upstream processes, particularly raw material extraction, may occur outside China. However, we believe that the impact of this on our results is tiny. According to a recent report by the Ministry of Industry and Information Technology of China (see in the reference list below), the domestic self-sufficiency rate for key PV materials—such as steel, aluminum, and PV glass—exceeds 98% since 2020. Thus, our calculation can reflect the highly integrated domestic supply chain in China.

We have further clarified this point in the Introduction:

Introduction: "*Meanwhile, China has achieved domestic self-sufficiency (>98%) for critical PV materials since 2020, including steel, aluminium, and PV glass (Ministry of Industry and Information Technology of the People's Republic of China et al., 2021). These emphasize China's influential role in guiding a net-zero transition through the PV manufacturing industry (Wang et al., 2023).*"

Supplementary Table 15. The carbon coefficients of resources, energy consumption and recycling materials.

Materials	Unit	CO_2	CH_4	N_2O	SF_6	Sum
Electricity (China's grid)	g CO_2 eq/kWh	5.8E-01	4.6E-02	2.7E-03	1.1E-05	6.3E-01
Steam (180°C 1.0 MPa steam from hard coal (85%))	g CO_2 eq/kWh	3.2E-01	2.7E-02	1.4E-03	4.2E-08	3.5E-01
Aluminum	g CO_2 eq/kWh	2.0E+01	1.5E+00	8.5E-02	8.9E-06	2.2E+01
Steel	g CO_2 eq/kWh	2.1E+00	1.5E-01	1.8E-03	1.4E-06	2.3E+00
Copper	g CO_2 eq/kWh	4.0E+00	1.2E-01	4.0E-02	1.3E-05	4.2E+00
Sliver	g CO_2 eq/kWh	6.8E+01	2.9E+00	5.0E-01	2.8E-04	7.1E+01
Polyethylene (PE)	g CO_2 eq/kWh	3.0E+00	2.8E-01	1.5E-02	1.0E-05	3.3E+00
EVA	g CO_2 eq/kWh	2.0E+00	3.7E-01	5.5E-03	6.4E-06	2.4E+00
Polyester resin	g CO_2 eq/kWh	2.2E+00	2.8E-01	6.3E-03	4.4E-06	2.5E+00
Polyamides (PA)	g CO_2 eq/kWh	8.3E+00	5.1E-01	4.1E-01	2.7E-05	9.2E+00
Proethylene glycol	g CO_2 eq/kWh	2.1E+00	2.4E-01	2.4E-02	5.0E-06	2.4E+00
Glass	g CO_2 eq/kWh	1.3E+00	1.2E-01	3.2E-03	1.2E-08	1.5E+00

Sealing silicone	g CO_2 eq/kWh	1.0E+01	4.6E-01	5.2E-02	7.6E-05	1.1E+01
Insulating coating	g CO_2 eq/kWh	4.8E+00	2.7E-01	4.1E-02	7.3E-05	5.1E+00
ABS	g CO_2 eq/kWh	2.6E+00	2.5E-01	1.9E-02	5.7E-06	2.9E+00
TPT	g CO_2 eq/kWh	1.1E+01	6.6E+00	5.7E-02	1.0E-04	1.7E+01
PLA	g CO_2 eq/kWh	5.4E+00	2.5E-01	3.1E-01	3.2E-05	6.0E+00
Citric acid	g CO_2 eq/kWh	8.6E+00	3.8E-01	1.6E-01	3.0E-05	9.1E+00
Hard coal	g CO_2 eq/kWh	1.0E-01	2.1E-01	6.2E-04	3.3E-07	3.1E-01
Petroleum coke	g CO_2 eq/kWh	3.0E-01	8.6E-02	1.4E-03	1.9E-07	3.8E-01
Cooling water (Deionized water)	g CO_2 eq/kWh	1.8E-03	1.6E-04	1.6E-05	1.2E-08	2.0E-03
Tap water	g CO_2 eq/kWh	1.7E-04	1.5E-05	2.0E-06	1.2E-08	1.8E-04
NH_3	g CO_2 eq/kWh	5.1E+00	3.6E-01	1.4E-01	3.3E-06	5.6E+00
O_2 (l)	g CO_2 eq/kWh	4.0E-01	1.2E-02	4.4E-03	1.2E-05	4.1E-01
N_2 (g)	g CO_2 eq/kWh	1.1E-01	9.2E-03	5.3E-04	9.3E-07	1.2E-01
Detergent	g CO_2 eq/kWh	6.6E-01	6.6E-02	4.8E-03	4.1E-10	7.3E-01
HNO_3(70%)	g CO_2 eq/kWh	3.7E-01	2.8E-02	6.7E-01	4.3E-07	1.1E+00
Sawdust	g CO_2 eq/kWh	6.1E-02	3.3E-03	1.2E-03	6.4E-06	6.6E-02
$POCI_3$	g CO_2 eq/kWh	3.6E+00	2.3E-01	1.0E-02	2.2E-05	3.8E+00
KOH (70%)	g CO_2 eq/kWh	9.0E-01	7.6E-02	2.5E-03	5.7E-06	9.8E-01
NaOH (30%)	g CO_2 eq/kWh	4.4E-01	1.9E-02	2.9E-03	7.6E-06	4.7E-01
Diamond wire (by Gabi)	kg CO_2 eq/m	5.6E-04	3.4E-05	9.3E-06	2.3E-09	6.1E-04
H_2SO_4 (98%)	g CO_2 eq/kWh	2.2E-01	3.5E-02	2.5E-03	1.3E-06	2.6E-01
HCI（37%)	g CO_2 eq/kWh	3.9E-01	1.7E-02	3.5E-03	7.6E-06	4.1E-01
HF (40%)	g CO_2 eq/kWh	1.3E+00	1.0E-01	8.4E-03	9.2E-06	1.4E+00
Glue	g CO_2 eq/kWh	9.1E-02	3.7E-03	5.9E-04	4.7E-07	9.5E-02
Cl_2	g CO_2 eq/kWh	9.6E-01	3.6E-02	3.0E-03	1.8E-05	1.0E+00
H_2	g CO_2 eq/kWh	1.4E+01	5.4E-01	1.9E-02	3.7E-05	1.4E+01
Anhydrous ethanol	g CO_2 eq/kWh	2.3E+00	1.9E-01	1.3E-02	1.2E-06	2.5E+00
Lube	g CO_2 eq/kWh	1.6E+00	2.1E-01	2.2E-03		1.8E+00
Charcoal	g CO_2 eq/kWh	1.4E+00	9.1E-03	3.1E-03	1.9E-07	1.4E+00
Graphite	g CO_2 eq/kWh	7.0E-01	6.0E-02	3.3E-03	5.6E-06	7.6E-01
Transport by truck	kg CO_2 eq/(tkm)	1.1E-01	4.1E-03	1.9E-04	1.1E-08	1.2E-01
Transport by rail	kg CO_2 eq/(tkm)	1.2E-02	9.7E-04	5.7E-05	2.4E-07	1.3E-02
Sewage treating	g CO_2 eq/kWh	5.2E-04	5.2E-04	5.8E-05	1.8E-09	1.1E-03
Waste water treating	g CO_2 eq/kWh	7.3E-04	1.8E-04	6.9E-05	4.9E-09	9.8E-04
Building materials treating	g CO_2 eq/kWh	1.6E-02	6.2E-04	7.6E-05	5.4E-08	1.6E-02

Solid waste treating	g CO_2 eq/kWh	2.9E+00	7.6E-02	7.8E-03	1.2E-05	3.0E+00
Concrete	g CO_2 eq/kWh	7.3E-02	2.8E-03	1.0E-04	7.6E-08	7.6E-02
Recycling aluminum	g CO_2 eq/kWh	-1.8E+01	-2.1E+00	-7.6E-02	-6.3E-06	-2.0E+01
Recycling steel	g CO_2 eq/kWh	-1.6E+00	-1.1E-01			-1.7E+00
Recycling silicon	g CO_2 eq/kWh	-1.5E+01	-5.7E-01	-3.7E-02	-6.3E-05	-1.5E+01
Recycling copper (Include direct emissions 0.257)	g CO_2 eq/kWh	-3.0E+00	-7.2E-02	-3.3E-02	-9.3E-06	-2.9E+00
Recycling silver	g CO_2 eq/kWh	-	-	-	-	-7.0E+01
Recycling glass	g CO_2 eq/kWh	-	-	-	-	-1.4E+00
Recycling sealing silicone	g CO_2 eq/kWh	-	-	-	-	-1.0E+01

Note: Gabi databases (Gabi: https://gabi.sphera.com) cover the carbon emissions coefficients of the main resources and energy consumption of each unit process (such as electricity, aluminum, steel, etc.) (GaBi Databases, 2024). In particular, the data for China is used preferentially, and the data for those materials not included in the database comes from papers. The coefficients of recycling materials refer to the second product by using the waste, such as aluminum, steel, copper, glass and silicone. Supplementary Table 15 shows all associated carbon coefficients, including four greenhouse gases with CO_2 equivalent coefficients from the Sixth Assessment Report (IPCC) (IPCC, 2022).

References:

1. GaBi Databases, <https://ghgprotocol.org/gabi-databases> (2024).
2. IPCC. Climate Change 2022: Impacts, Adaptation, and Vulnerability. Intergovernmental Panel on Climate Change (IPCC), (2022).
3. Ministry of Industry and Information Technology of the People's Republic of China, Ministry of Science and Technology of the People's Republic of China & Ministry of Natural Resources of the People's Republic of China. 14th Five-Year Plan for the Development of the Raw Materials Industry (in Chinese), <https://www.gov.cn/zhengce/zhengceku/2021-12/29/content_5665166.htm> (2021).
4. Wang, Y., Wang, R., Tanaka, K., et al. Accelerating the energy transition towards photovoltaic and wind in China. Nature 619, 761-767 (2023).

Comment 3:

The decarbonisation effects of PV manufacturing for China is described quite well, but the international effects could differ, if manufacturing takes place in regions with a lower or even higher carbon footprint of the energy used. Please comment.

Response:

Thank you for this insightful comment. Indeed, the decarbonization contributions of PV manufacturing is highly dependent on the regional electricity mix. In principle, the emissions associated with PV manufacturing should reflect the carbon intensity of electricity in the region where the manufacturing occurs, whereas the decarbonization benefits should be calculated based on the grid intensity where the PV systems are deployed (Supplementary Figures 10 and 11). In our study, we focus on the decarbonization contributions of China's PV manufacturing both domestically—using China's national grid emission intensity—and globally—using the world average grid intensity (Supplementary Figure 8). While a detailed analysis of PV manufacturing in other regions is beyond the scope of this study, our methodology is adaptable and can be applied to other countries or regions with appropriate local energy data.

We have added extra clarification in the Methods section.

Methods: "*The carbon emissions associated with PV manufacturing are determined by the electricity intensity of the region where production occurs, whereas the decarbonization contributions of PV systems are determined by the gap between the carbon intensity of PV-generated electricity and the average grid intensity at the deployment location. The regional grid intensity is calculated based on the mix of ten different electricity generation technologies, including coal, natural gas, wind, solar PV, oil, fossil fuels with CCUS, and others (Schlömer et al., 2014).*"

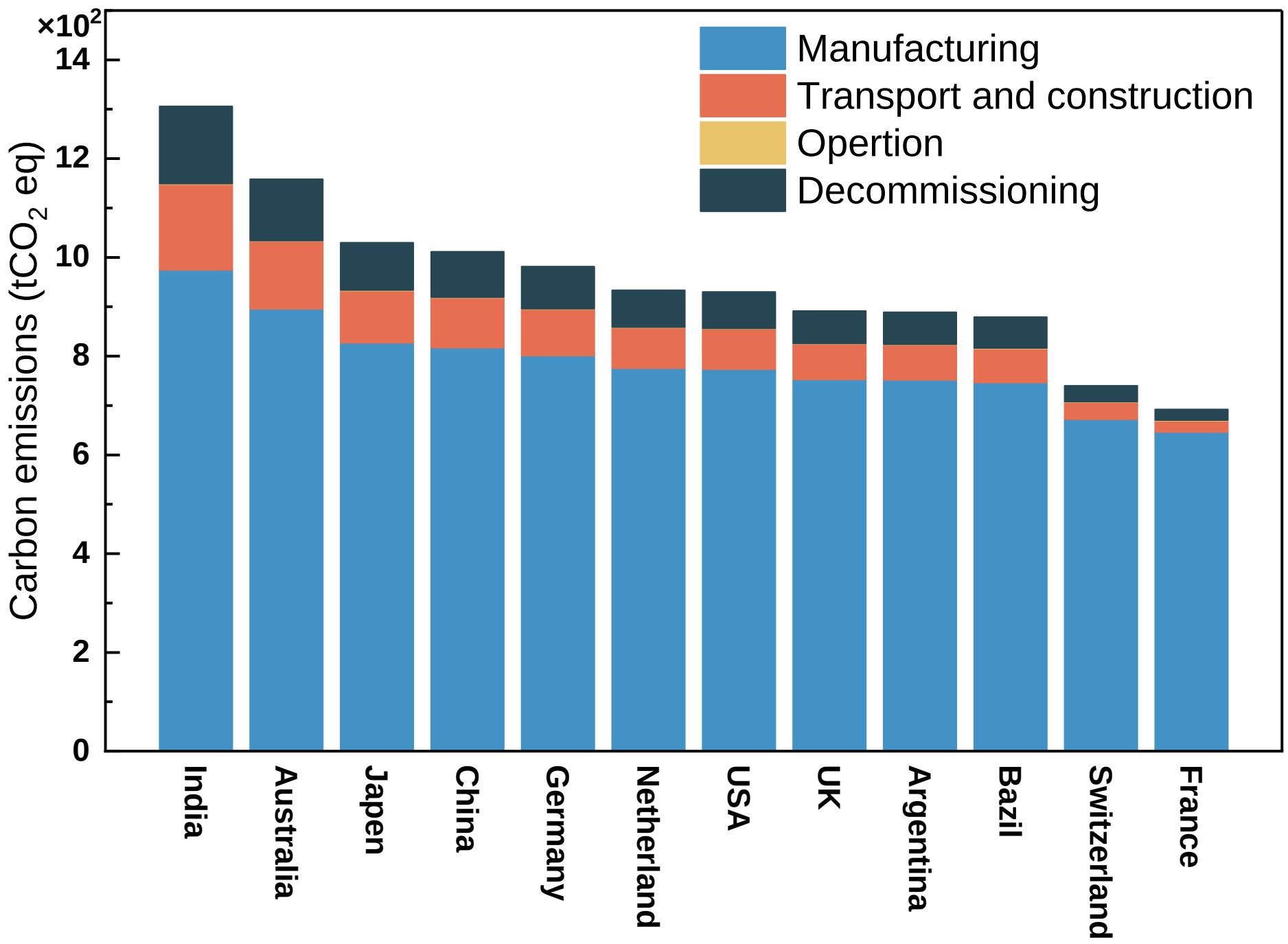

Supplementary Figure 10. The sensitivity analysis to total carbon emissions on the grid's carbon intensity of different countries.

Note: Assuming that the production process of the PV system varies in regions with different carbon intensities of electricity from the grid, we calculate the life-cycle carbon emissions for 1MWp PV system considering different carbon intensity of the grid. The electricity structures of major countries are selected, with carbon intensities ranging from 0.106 to 1.112 gCO2 eq/kWh.

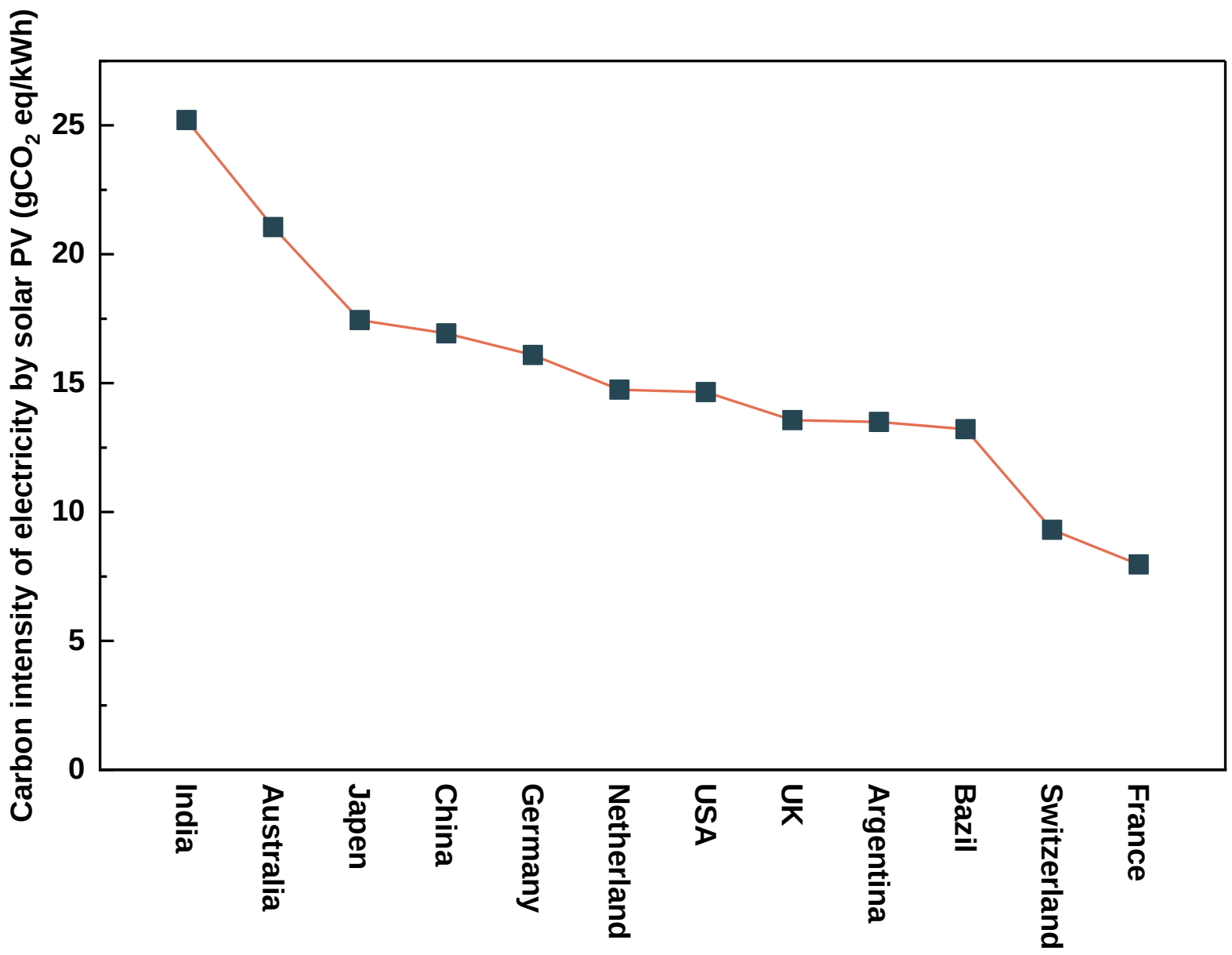

Supplementary Figure 11. The sensitivity analysis to the PV carbon intensity on the grid's carbon intensity of different countries.

Note: Based on the average solar radiation intensity, performance and manufacturing level at the year of 2022 in China, with global tilted irradiance of 1756kWh/m2 per year, we calculate the carbon intensity of electricity from PV by considering the carbon intensity of grid in different regions.

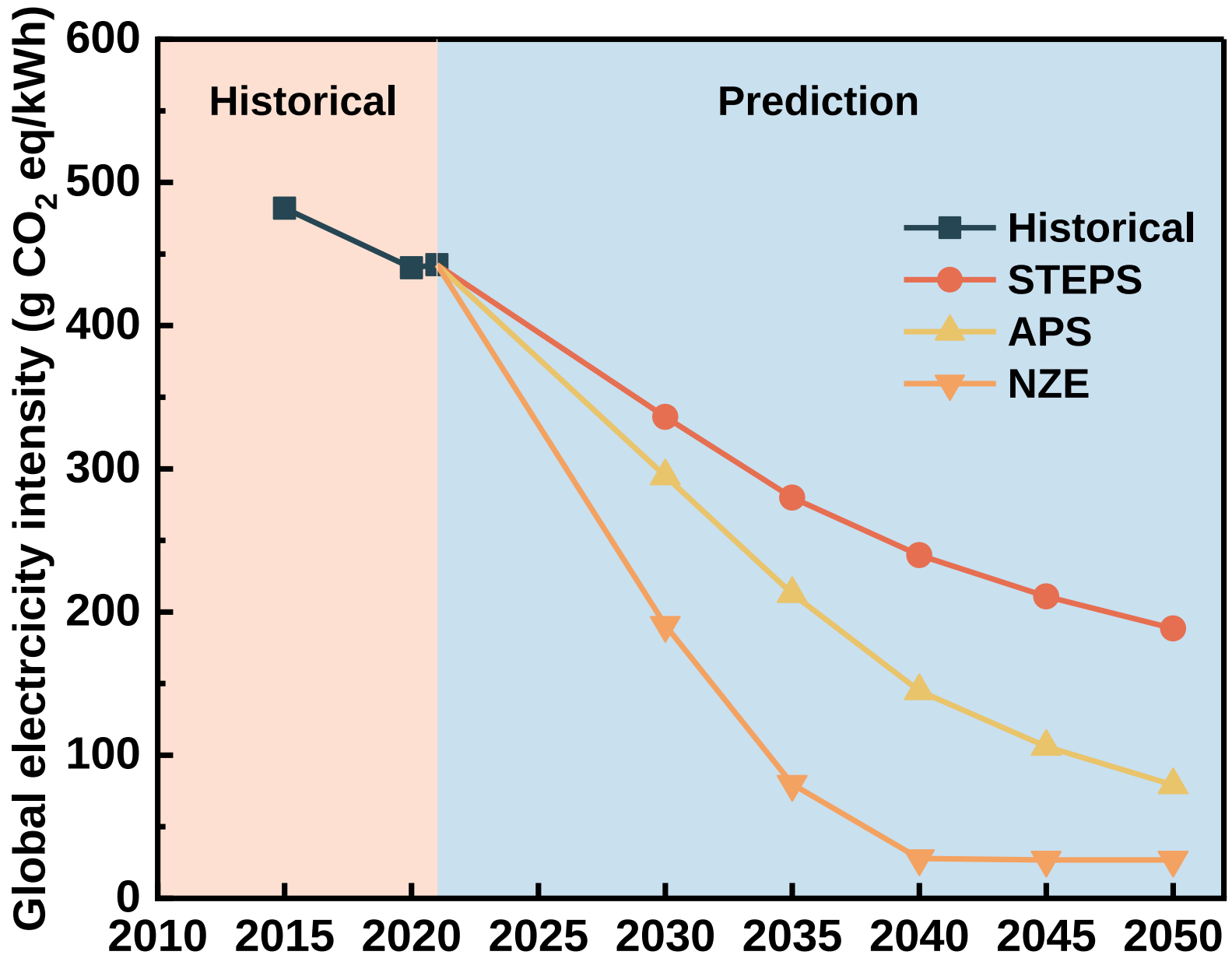

Supplementary Figure 8. The average carbon intensity of electricity from PV at the global scale.

Note: The carbon intensity of electricity from regional grid can be calculated by equation 6, and we consider 10 different electricity generation methods. Their carbon intensity mainly refers to the Intergovernmental Panel on Climate Change (IPCC) (Schlöme et al., 2014), and the rest which are fossil fuels with CCUS and others are assumed to 10% of that without CCUS and zero gCO2 eq/kWh. The electricity structure scenarios in global from 2030 to 2050 are obtained from the International Energy Agency (IEA) and its extended database [https://www.iea.org/reports/world-energy-outlook-2022].

Reference:

1. Schlömer S., T. B., L. Fulton, E. Hertwich, A. McKinnon, D. Perczyk, J. Roy, R. Schaeffer, R. Sims, P. Smith, and R. Wiser. Technology-specific cost and performance parameters. Intergovernmental Panel on Climate Change (IPCC), Cambridge University Press (2014).

Comment 4:

How will the energy mix in China change over time? Its not clear if the decarbonisation is only due to the change of the electricity generation, or if there is also a decarbonisation effect of the whole energy mix (e,g. power to X, etc.)

Response:

Thanks for this valuable comment. The decarbonization contributions of PV systems are calculated by comparing the carbon intensity of PV-generated electricity with the dynamically changing grid carbon intensity. Our analysis focuses on the lifecycle of PV systems and the electricity PV generates, without extending to downstream applications with PV-generated electricity such as power-to-X pathways. We acknowledge that further decarbonization may occur depending on how PV electricity is utilized. However, such pathways are beyond the scope of this study. Considering them could likely change the total decarbonization potential attributed to PV systems, but would not alter the relative contributions of PV manufacturing to total PV-induced decarbonization, nor would it change the identified strategic timing. Therefore, our key conclusions and policy implications remain robust.

In response, we have revised the Methods section to reflect the point above.

Methods: "*Note that the scope of this study does not include the end-use applications of PV-generated electricity (e.g., power-to-X). While such uses may have a limited impact on the total decarbonization contributions of PV, they do not affect the proportional contributions from PV manufactures or the predicted strategic timing, and therefore would not compromise the policy recommendations of our study.*"

Comment 5:

line 176: What system prices are used for this calculation? It is assumed that this is a micro economic analysis. Are macro economic aspects considered at all?

Response:

Thanks for your questions. In our economic cost evaluation, we differentiate between domestic and international system prices. For the Chinese domestic market, the system prices are obtained from China's Photovoltaic Industry Development Roadmap (2018-2023) (CPIA. 2023), which includes the investment costs of PV systems in China. For the global market, the system prices come from Renewable Power Generation Costs in

2021 (IRENA, 2022). Supplementary Table 25 presents the distribution of economic costs for PV system in China and the global average. Please note that we make use of discount rates ranging from 0.1% to 5% in our economic analysis (Supplementary Figure 14).

Supplementary Table 25. The economic cost of PV system at the global scale.

Process	Economic cost in China ($\times10^4$ CNY)	Ratio in China	Economic cost in global ($\times10^4$ CNY)	Ratio in global
1-Industrial silicon	5	0.97%	8	1.04%
2-Polysilicon	22	4.13%	32	4.44%
3-Silicon wafers	26	4.78%	37	5.15%
4-Cell	48	9.04%	70	9.73%
5-PV modules	92	17.26%	134	18.57%
6- Supporting modules	120	22.54%	175	24.25%
7- transport and construction	100	18.70%	145	20.12%
8-Operation	63	11.90%	63	8.80%
9-Decommission	57	10.68%	57	7.90%
Sum	534	100%	721	100%

Note: The economic costs are divided into nine stages: 1-industrial silicon, 2-solar-grade polysilicon, 3-silicon wafers, 4-cells, 5-PV modules, 6-supporting modules, 7-transportation and construction, 8-operation and 9-decommissioning. Supplementary Table 25 presents the distribution of economic costs for PV system.

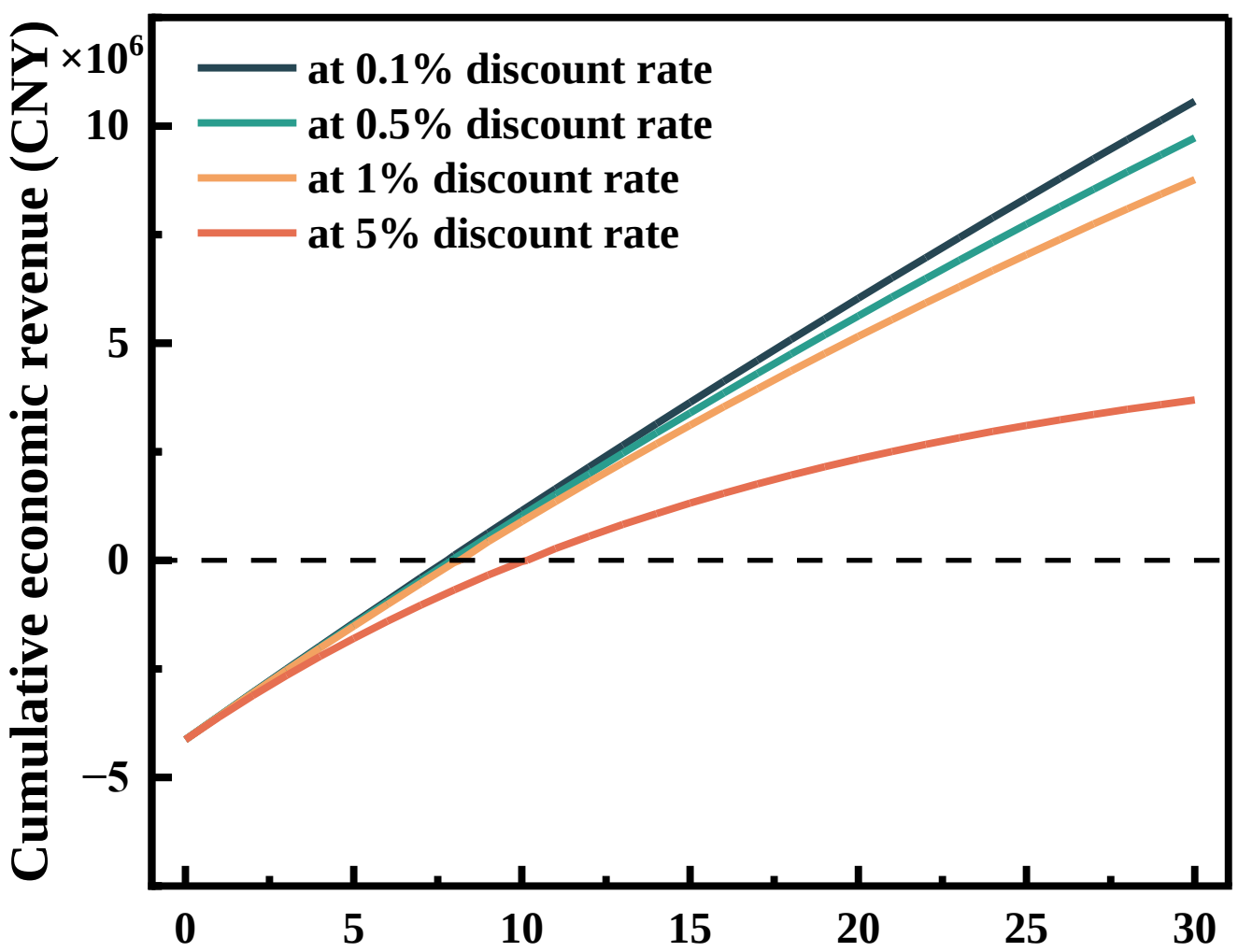

Supplementary Figure 1. The payback times for economy of PV in China at different discount rate.

Note: Based on the electricity price and economic investment in China, we calculate the payback times for economy of PV system, varying the discount rate from 0.1% to 5%.

References:

1. CPIA. China's Photovoltaic Industry Development Roadmap (2018-2023). China Photovoltaic Industry Association (CPIA), (2023).
2. IRENA. Renewable Power Generation Costs in 2021. International Renewable Energy Agency (IRENA), (2022).

Comment 6:

line 178: Assumption of stable Chinese share after 2027: Is this a realistic assumption? So far there is only limited activity outside of China to build up the whole value chain of silicon PV (India). Some parts of the value chain namely polysilicon and wafer are concentrated in China and require a considerable time to scale somewhere else.

Response:

Thank you for your insightful comments. The assumption regarding China's shares in this study is based on the projections from a report by IEA, which accounts for the expected impact of major policy incentives, such as the US Inflation Reduction Act and India's Production Linked Incentive Scheme. These initiatives are anticipated to drive significant growth in PV manufacturing capacity outside China over the next five years. According to the IEA (2022), such developments may reduce China's global share of PV manufacturing to around 60%-75% post-2027. We recognize that the upstream segments of the value chain, such as polysilicon and wafer production, remain highly concentrated in China and will take time to diversify geographically. However, while uncertainties in China's future market share may influence the absolute decarbonization contributions of China's PV industry, they do not affect the proportional contribution from China's PV manufacturing or the predicted strategic timing. Therefore, it is our conviction that the major conclusions and policy implications remain valid.

We have made a note to the Supplementary information.

Supplementary Table 29 Note: *"We assume a linear progression, starting from 55.13% in 2010, reaching 65.37% in 2015 and 84.00% in 2021, and stabilizing at 69.20% since 2027 (CPIA, 2023; IEA, 2022). This is based on the projections from report by IEA, considering the impact of major policy incentives, such as the US Inflation Reduction*

Act and India's Production Linked Incentive scheme (IEA, 2022).”

References:

1. CPIA. China's Photovoltaic Industry Development Roadmap (2018-2022). China Photovoltaic Industry Association (CPIA), (2023).
2. IEA. Solar PV Global Supply Chains Analysis. International Energy Agency (IEA), Paris (2022).
3. IEA. Will new PV manufacturing policies in the United States, India and the European Union create global PV supply diversification? International Energy Agency (IEA), Paris (2022).

Comment 7:

Fig 4: What are supporting modules? Is this the support structure where the modules are mounted? Do you differentiate between fixed mount, tracking and rooftop?

Response:

Thanks for your insightful questions. In our study, a supporting module refers not only to support structures for mounting PV panels but more broadly to all components excluding the PV panels themselves. This includes inverters, PV holders, cables, junction boxes, etc. (see Supplementary Table 8).

We did not explicitly differentiate between fixed mount, tracking and rooftop PV systems. The amount of electricity generated by PV modules has been determined by considering the installed capacity of the PV system (MWp), the annual peak sunshine hours, the performance ratio of the PV power plant (assumed to be 79%), and the average loss coefficients of electricity transmission in China (currently below 5%, see Supplementary Table 16). In our framework, following the current policies that ensure efficient utilization of renewable-generated electricity, centralized PV systems are connected to the grid that allows for efficient distribution and use of generated electricity, while distributed PV systems can be equipped with energy storage solutions, enabling effective management and optimization of electricity usage (China Energy Storage Alliance, 2024; Bullich-Massagué, 2020, National Development and Reform Commission and National Energy Administration, 2021). Moreover, with respect to economic costs, the annual operation and maintenance costs in 2023 for centralized and distributed PV systems are 39,000 CNY/MW and 47,000 CNY/MW, respectively (China Photovoltaic Industry Association, 2023). The difference in operation and

maintenance costs is minimal compared to the initial investment cost of 3.18-3.40 million CNY/MW for PV systems. Therefore, the difference in terms of costs also does not significantly impact the main conclusions of this study.

Supplementary Table 8. The LCI of supporting modules (Fthenakis et al., 2011; Jia, 2012; Wang et al., 2022)

	Materials	Unit	Consumption
Inverter	Steel	kg	5,760
	Copper	kg	1,340
	Aluminum	kg	524
	Polyethylene (PE)	kg	88
	Polyamides (PA)	kg	284
	Lube	kg	3,524
	Polyester resin	kg	176
	Electricity	kWh	18,320
Holder	Steel	kg	8,410
	Aluminum	kg	8,410
Junction box	ABS	kg	693
	Copper wire	kg	693
Cable	Copper	kg	4,360
	Insulating coating	kg	3,000

Note: The inverter has a life span of 15 years. The 1MWp PV system is equipped with two inverters, and four inverters would be required over the 30-year operating cycle of the PV system. The inventory of four inverters is shown in Supplementary Table 8.

Supplementary Table16. The loss coefficient of electricity transmission in year 2018-2022 (China Electricity Council, 2023).

Year	Loss coefficient
2018	0.9373
2019	0.9407
2020	0.9440
2021	0.9474
2022	0.9516

References:

1. Bullich-Massagué, E., Cifuentes-García, F., Glenny-Crende, I., et al. A review of energy storage technologies for large scale photovoltaic power plants. Applied Energy, 275, 116727 (2020).

2. China’s Photovoltaic Industry Development Roadmap (2018-2022). (China Photovoltaic Industry Association, 2023).
3. Energy Storage Industry White Paper 2024. (China Energy Storage Alliance, 2024).
4. Fthenakis, V. M. & Kim, H. C. Photovoltaics: Life-cycle analyses. Solar Energy 85, 1609-1628 (2011).
5. Jia, L. Environmental effects investigating for the grid-connected BAPV, Tianjin University, (Ph.D dissertation, 2012).
6. Promoting the integration of charge and storage and multi-energy complementary development of power sources (in Chinese). (National Development and Reform Commission and National Energy Administration, 2021). https://www.gov.cn/zhengce/zhengceku/2021-03/06/content_5590895.htm
7. The Annual Development Report of Chinese Electricity Industry 2016-2022. China Electricity Council, Beijing (2023).
8. Wang, Z., Chen, Z., Wennersten, R. & Sun, Q. in 2022 International Conference on Power Energy Systems and Applications (ICoPESA). 553-564 (IEEE).

Authors' Responses to Reviewer Comments

Journal: Nature Communications

Manuscript #: NCOMMS-24-13270C

Title of Paper: Strategic Timing for the Decarbonization Contributions by China's Photovoltaic Manufacturers in Global Energy Transition

Authors: Qingyang Lin, Chenglin Li, Kai Wang, Kai Fang, Meixuan Xin, Siqi Wang, Anqi Xu, Zhenci Xu, Yue Lu, Xiang Gao

Article Type: Research paper

We appreciate the time and efforts taken by the Reviewers in reviewing our revised manuscript. We have carefully addressed all the comments point by point, with detailed responses provided below and highlighted changes in the revised version of the manuscript. We hope that the now the manuscript satisfies the journal's standards. If not, however, we are happy to work further to make the paper acceptable for publication in *Nature Communications*.

Reviewer #5

My comments were addressed adequately and the paper can now be published.

Response:

We thank the reviewer for the supportive feedback.

Reviewer #6

General Comments

I congratulate the authors on the work undertaken to date and the detailed and careful manner in which their model has been built. This is a very relevant topic for consideration by Chinese policymakers and global policymakers, as well as by scholars of resource economics and policy.

Response:

Thanks for the supportive feedback, we have addressed all comments with detailed response below.

Comment 1:

Conceptually, however, the novelty or insight of the paper is not particularly clear. Although the allocation of manufacturing emissions to upstream firms whose output is constrained by policy based on those emissions (in China, at least) does create inefficiencies, the allocation of emissions to different parties across value chains depending on perspective is not a new one. To more clearly outline their contribution and argument, including in the paper's abstract, the authors should consider strengthen their engagement with the existing frameworks (e.g. GHG Protocol) and corresponding literature on emissions accounting, especially as it relates to industrial policy.

Response:

We fully agree that the previous version did not sufficiently articulate either the conceptual novelty of the paper or its relationship to existing emissions accounting frameworks, particularly in the context of industrial policy. In response, we have substantially revised the text to clarify our contributions to the literature more explicitly. In the following response, we will first clarify the role of GHG Protocol in our study, and then elaborate on the main contribution of this study.

First, we would like to declare that our study does not aim to challenge the GHG Protocol frameworks. Instead, our approach aligns well with the GHG Protocol in accounting for the emissions of the lifetime of the PV system. In this study, we took a further step to quantify the total decarbonization contributions and propose an allocation principle to determine the share of PV manufacturers from three criteria: energy consumption, economic costs and carbon emissions. Therefore, the GHG Protocol serves as a reference for emissions accounting, which is merely a part of our methodological framework. To clarify our framework, we have three layers in our framework (see updated Supplementary Figure 1 below). The first, referred to as Layer I, examines the detailed production steps for PV panels. Layer II assessment covers various stages, including manufacturing (like PV panels and inverters), transportation, construction, operation, and decommissioning processes. The third, known as Layer III, further incorporates decarbonization contributions. Specifically, the Layer II encompasses Scopes 1, 2, and 3 of the GHG Protocol. We have followed your suggestion to add references on emissions accounting such as the GHG Protocol to the manuscript.

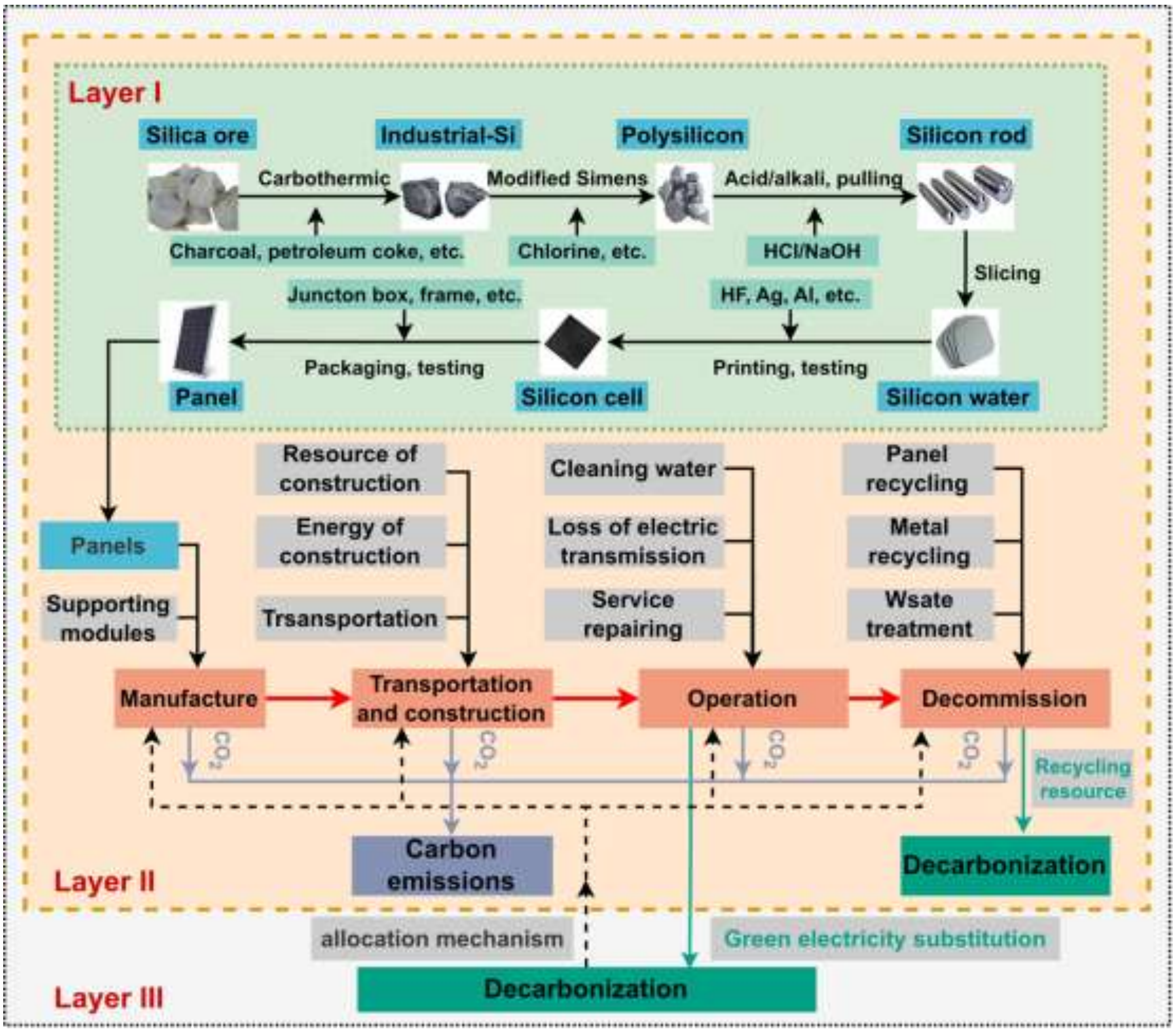

Supplementary Figure 1. Producer decarbonization contribution (PDC) framework.

Second, the main contributions of this paper include:

1. Recognizing the decarbonization contributions of PV manufacturers, which is conceptually distinct from emission responsibilities. Our study demonstrates that all stages of the lifecycle of PV systems contribute to decarbonization, including manufacturing, transportation and construction, operation, and decommissioning. By focusing on the manufacturing stage, we emphasize that the manufactures' emissions are a prerequisite for the overall decarbonization of PV industry. Without these initial emissions, the deployment of PV systems and their subsequent benefits associated with emissions reduction would not be achieved. This argument has been supported by a growing number of literature (e.g., Jiang et al., 2019; Liu et al., 2019; Suvajit et al., 2021), and has been accepted by other reviewers. Thus, recognizing the decarbonization contributions from PV manufacturers is particularly important in the policy context.

Although the long-term decarbonization goals of China depend largely on development in renewable energy, short-term policies tend to pose a strict restriction on the manufacturers of PV (Helveston et al., 2022; Kozul-Wright, 2023; Liang and You, 2023), as they produce most carbon emissions within PV's entire lifespan, and China has set ambitious goals to peak carbon emissions before 2030. In particular, key upstream suppliers within the PV value chain (e.g. PV glass manufacturers) are not explicitly recognized as part of the PV industry and may therefore be subject to regulatory treatment that does not fully reflect their role in enabling PV downstream decarbonization. Such disruptions risk undermines the sustainable development of the PV industry and may impede the progress towards China's energy transition goals that non-fossil energy makes up over 80% of the total energy consumption by 2060 (Ou et al., 2024). Therefore, rather than concluding that PV manufacturers are a primary driver of decarbonization, we aim to bring attention to the overlooked contributions of the PV manufacturing industry to both China's and global decarbonization progresses by proposing a producer decarbonization contribution (PDC) framework.

2. Quantifying these decarbonization contributions using three alternative criteria—energy consumption, carbon emissions, and economic costs. In this study, we first account for the total decarbonization contributions of the entire PV industry based on the national and global deployment projections from the IEA. Noted that in existing studies, decarbonization was commonly evaluated using a fixed emission intensity as benchmark (e.g., compare with coal-fired electricity). While this approach is widely used, it may not provide an adequate basis for policy-relevant insights, because a fixed benchmark can overstate decarbonization contributions as PV deployment expands. We therefore adopt a dynamic grid-based benchmark in this study. A more detailed explanation is provided in our response to Comment 2.

We then allocate a fair share of these contributions to the PV manufacturers following three criteria: energy (characterized by the energy consumption), economy (characterized by the economic costs), and carbon emissions, respectively (Supplementary Figures 6 and 7). They span the core dimensions of resource use, economic viability and environmental impact. Anyhow, fairly assessing the role of manufacturing in decarbonization is inherently complex and shaped by many interrelated factors. The criteria used in this study are not fixed, and alternatives may offer additional insights. All in all, we hope that this work contributes to a broader dialogue on decarbonization contributions and encourages further investigation within the research community.

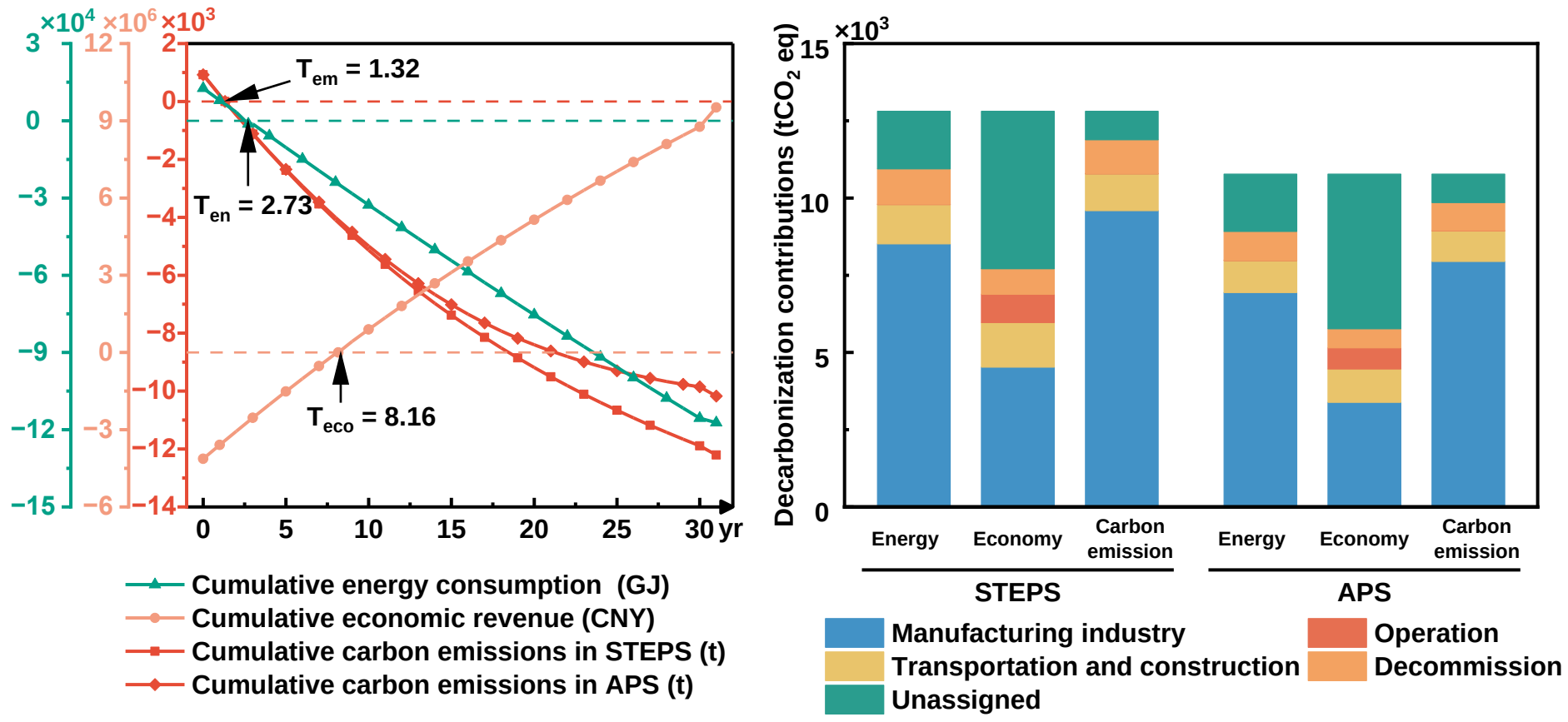

(a) The payback times of three criteria (b) The decarbonization of three criteria

Supplementary Figure 6. The payback times and decarbonization of three dimensions in China.

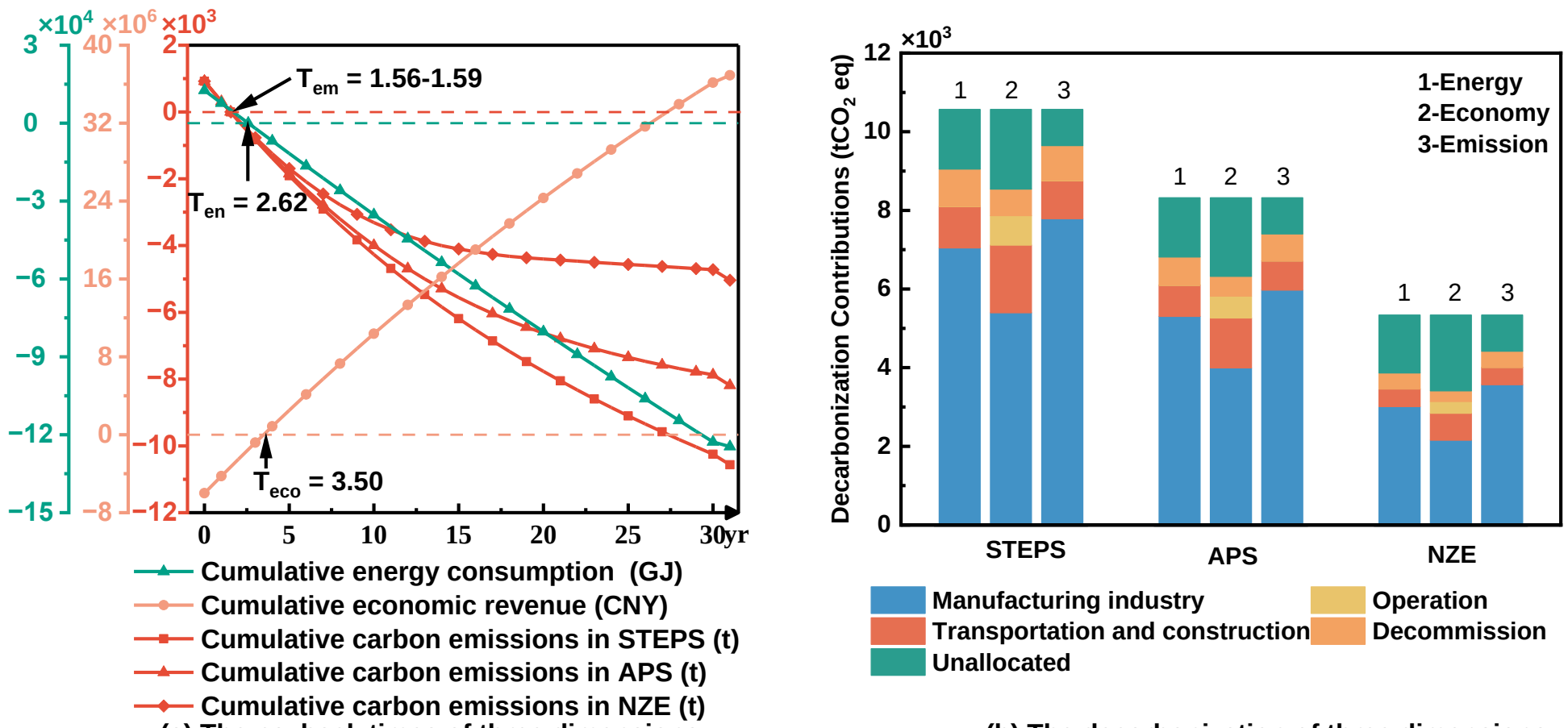

(a) The payback times of three dimensions (b) The decarbonization of three dimensions

Supplementary Figure 7. The payback times and decarbonization of three dimensions at the global scale.

3. Identifying the strategic timing of these contributions together with their policy implications. One key contribution of our paper is that we define and quantify, for the first time, the concept of strategic timing. Simply put, it represents the predicted period during which PV manufacturers make their highest decarbonization contributions. This concept aims to better serve both policymakers and industrial experts in the evaluation and choice of strategies for the PV manufacturing sector. There are two major policy implications:

(1) Prior to this timing, supportive regulations are needed to ensure rapid development of the PV manufacturing industry for successful energy transition. Nevertheless, the

strategic timing under the NZE scenario is projected to occur in 2030 at the global level, five years earlier than 2035— the strategic timing under the APS scenario for China. When the world has reached its strategic timing, while China has not yet, a shift in policy focus from the global market to China's domestic market is needed to avoid potential PV overproduction at the global level (Yu et al., 2021). This strategy aligns with China's latest nationally determined contributions, announced in 2025, to expand installed capacity of solar and wind to over six times the 2020 level by 2035 (China Announces 2035 Nationally Determined Contribution, 2025). Noted that we draw this conclusion, based on the submission in 2024, much earlier than the announced year, to some extent confirming the robustness of our framework.

(2) After this timing, stricter regulations may be warranted to prevent overcapacity and resource inefficiency (IEA, 2024; Wang et al., 2022). Notably, we find that the strategic timing is consistent across all the three assessment criteria, and this reinforces the robustness and reliability of our results (Figures 1 and 2).

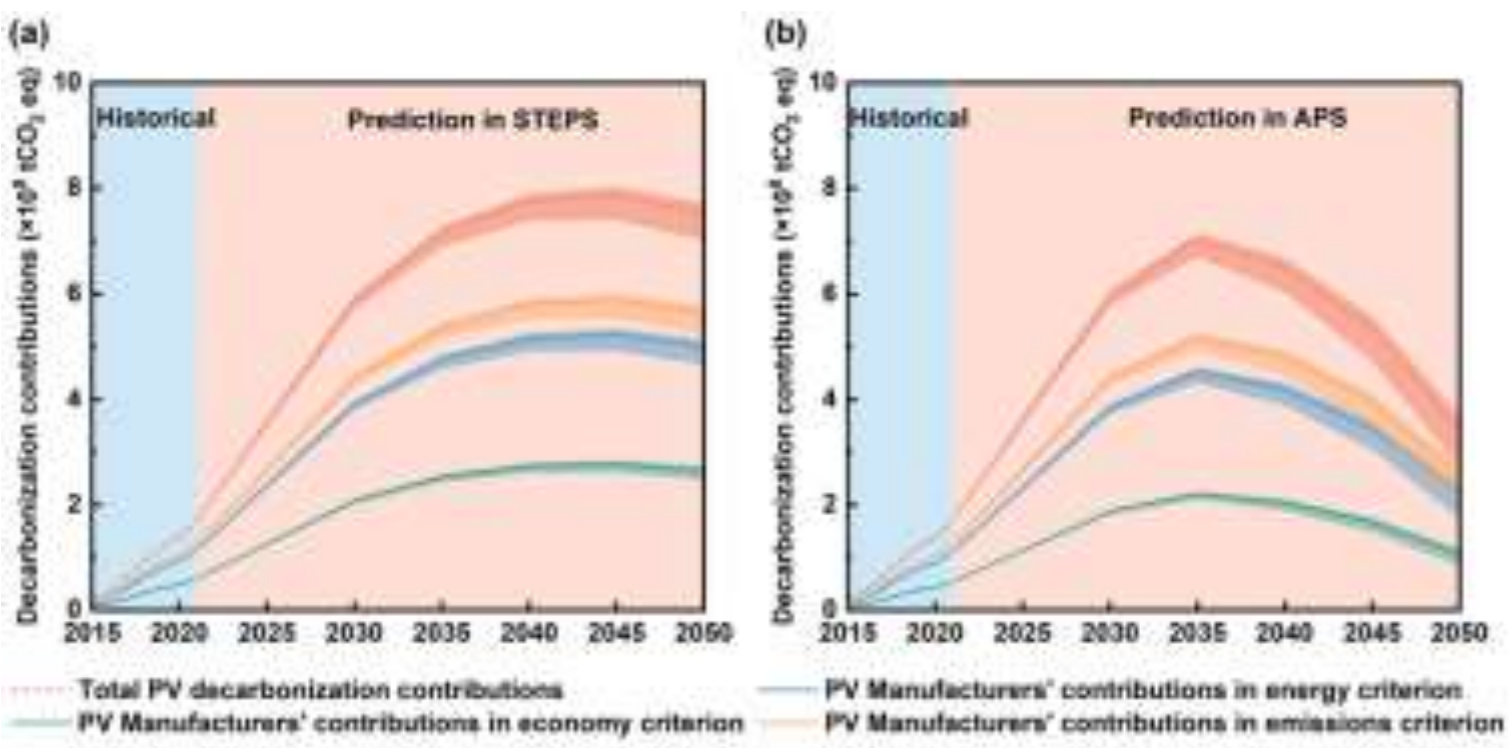

Fig. 1 | The decarbonization contributions and associated strategic timing of the PV manufacturers at China's national level: (a) STEPS and (b) APS.

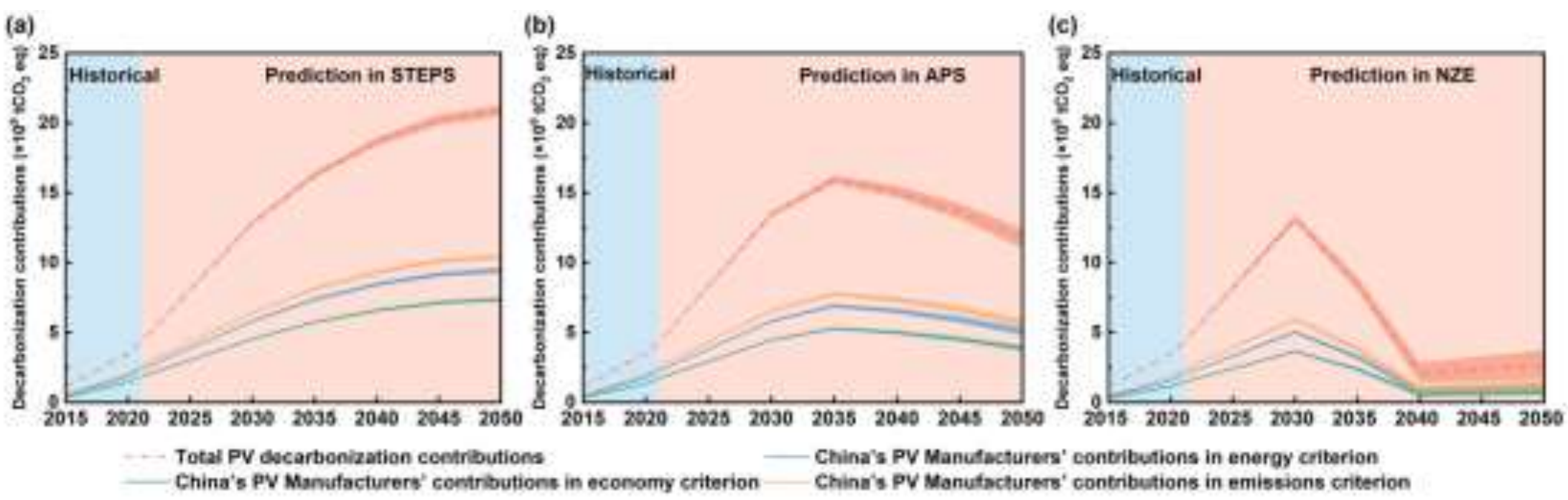

Fig. 2 | The decarbonization contributions and associated strategic timing of China's PV manufactures at the global level: (a) STEPS, (b) APS and (c) NZE.

We have now revised the text as follows.

Abstract: *"Photovoltaic (PV) technology is central to clean electricity and the Sustainable Development Goals. However, China's PV manufacturing industry that accounts for nearly two-thirds of global capacity is criticized for high carbon emissions, without which PV deployment and operation cannot be sustainable. Existing studies often assign emission responsibility to PV manufacturers while attributing decarbonization contributions to power generation entities, thereby overlooking upstream producers' decarbonization contributions. This study proposes a Producer Decarbonization Contribution (PDC) framework to quantify these long-overlooked contributions at both national and global scales by using three alternative criteria: energy, economic, and carbon emissions. We show that China's PV manufacturers could contribute 31.4%-73.8% of the total PV decarbonization contributions domestically and 27.9%-45.4% globally. We further identify a "strategic timing", defined as the point at which the PV's decarbonization contributions peak, is projected around 2035 in China and 2030 globally under IEA's NZE Scenario. These findings suggest that supportive policies need to be sustained in China before 2035, whereas more stringent measures should be required globally from 2030 to prevent potential overproduction and resource waste. The PDC framework, which provides a first measure of the decarbonization contributions of PV manufacturers, enables more sound and wise industrial policy design by avoiding misleading signals for overcapacity, and can be extended to making strategic planning for other renewables."*

Methods: *"We have three layers in our framework (see Supplementary Figure 2). The first, referred to as Layer I, examines the detailed production steps for PV panels. Layer II assessment covers various stages, including manufacturing (like PV panels and inverters), transportation, construction, operation, and decommissioning processes. The third, known as Layer III, further incorporates decarbonization contributions. Specifically, the Layer II encompasses Scopes 1, 2, and 3 of the GHG Protocol."*

References:

1. *China Announces 2035 Nationally Determined Contribution, Embarking on a New Journey to Address Climate Change (in Chinese),* <https://www.gov.cn/yaowen/liebiao/202509/content_7042502.htm> (2025).
2. Helveston, J. P., He, G. & Davidson, M. R. Quantifying the cost savings of global solar photovoltaic supply chains. *Nature* **612**, 83–87 (2022).

3. IEA. Renewables 2023. *International Energy Agency (IEA)*, Paris (2024).
4. Jiang, M., Gao, X., Guan, Q., Hao, X. & An, F. The structural roles of sectors and their contributions to global carbon emissions: A complex network perspective. *Journal of Cleaner Production* **208**, 426–435 (2019).
5. Kozul-Wright, R. China's policy strategies for green low-carbon development: Perspective from South-South cooperation. *United Nations Conference on Trade and Development (UNCTD)*, (2023).
6. Liang, H. & You, F. Reshoring silicon photovoltaics manufacturing contributes to decarbonization and climate change mitigation. *Nature Communications* **14**, 1274 (2023).
7. Liu, D., Liu, J., Wang, S., Xu, M. & Akbar, S. J. Contribution of international photovoltaic trade to global greenhouse gas emission reduction: the example of China. *Resources, Conservation and Recycling* **143**, 114–118 (2019).
8. Ou, Y., Hsiao, C. Y.-L. & Chui, C. M. How does the supply chain market respond to policy shocks? Evidence from solar photovoltaic sectors in China. *Renewable Energy* **232**, 121129 (2024).
9. Suvajit, B., Muhammad, A. K., Muhammad I. H. Searching appropriate system boundary for accounting India's emission inventory for the responsibility to reduce carbon emissions. *Journal of Environmental Management* **295**, 112907 (2021).
10. Wang, C., Feng, K., Liu, X., Wang, P., Chen, W.-Q. & Li, J. Looming challenge of photovoltaic waste under China's solar ambition: A spatial–temporal assessment. *Applied Energy* **307**, 118186 (2022).
11. Yu, S., Lu, T., Hu, X., Liu, L. & Wei, Y.-M. Determinants of overcapacity in China's renewable energy industry: Evidence from wind, photovoltaic, and biomass energy enterprises. *Energy Economics* **97**, 105056 (2021).

Comment 2:

To illustrate this point, the paper argues that China's PV manufacturing industry has not been recognised for its contribution to decarbonisation. However, one could argue that existing GHG accounting frameworks do exactly this, by allocating the 'benefits' of PV manufacturing to producers in the form of low, or zero, Scope 3 emissions of the products they produce. When compared to Scope 3 emissions from substitutes (e.g. coal-fired power plants, gas-fired power plants), the benefits are clear. If previous work has not specifically highlighted this, it does not change the fact that current frameworks

do, by design, measure the downstream emissions of manufactured products and, thereby, their relative performance against substitutes. Fundamentally, the insight here is not new, although the method used to arrive at it is different.

Response:

Thank you for this insightful comment, which allows us to better clarify the novelty of our study in relation to existing GHG accounting frameworks. Existing studies on PV systems predominantly focus on assigning emission responsibilities across the value chain, often attributing a substantial share to PV manufacturers while overlooking their role in enabling downstream decarbonization (Banerjee et al., 2021; Ye et al., 2023). More broadly, while existing literature has made significant progress in allocating emissions along value chains, relatively limited attention has been given to systematically attributing downstream decarbonization benefits back to upstream producers (Davis et al., 2025; Klaassen and Stoll, 2021; Zhang et al., 2023). As a result, the benefits of emissions reduction are typically credited to power generation entities during the operational phase, with limited recognition of upstream contributions. For example, prevailing accounting frameworks such as the GHG Protocol treat emission reductions from products separately from Scope 1, 2, and 3 inventories (Greenhouse Gas Protocol, 2022), and do not explicitly incorporate avoided emissions into core accounting boundaries (WRI and WBCSD, 2011). Under such frameworks, producers may be associated with low or even zero emissions, which can be interpreted as a favorable outcome (Forster et al., 2023). However, this perspective reflects merely a reduction in responsibility rather than an explicit recognition of contribution. In other words, the absence of emissions does not equate to a measurable contribution to decarbonization. This gap rises a fundamental question: how large are the decarbonization contributions of PV manufacturers?

In addition, even when the "benefits" of PV relative to fossil-based alternatives are considered, these are often evaluated using fixed emission intensity benchmarks (e.g., coal-fired electricity) (Table 1). While this approach is widely used, it may not provide an adequate basis for policy-relevant insights. Under such a fixed benchmark, the estimated avoided emissions of PV would increase substantially with installed capacity, potentially leading to the conclusion that continuous and unconditional PV capacity expansion is always reasonable. which we know is not correct (Figure 1). This is because the avoided emissions enabled by PV depend on the carbon intensity of the electricity that PV replaces. As power systems become increasingly decarbonized, the marginal contribution of upcoming PV deployment may narrow. This raises a further

question: how will the decarbonization contributions of PV manufacturers vary over time? To overcome this flaw, instead of using fixed emission intensity from coal-fired electricity, our paper makes use of the dynamically changing intensity from the grid based on the projection of the electricity generation structure (Wang et al., 2024). The decarbonization contributions of PV systems are determined by comparing the carbon intensity of PV-generated electricity, with the dynamically changing intensity for the grid based on the predicted energy structure. As the grid becomes progressively decarbonized, the marginal decarbonization contribution of PV correspondingly declines. This dynamics allows us to identify a peak in PV decarbonization contributions—defined as the strategic timing—beyond which the marginal benefit decreases. In turn, this provides a more realistic and policy-relevant basis for assessing when supportive measures should be strengthened or moderated, avoiding misleading signals for the PV industry.

Overall, while the general insight that PV outperforms fossil-based alternatives is not new, our contribution lies in a framework that links decarbonization contributions to long-term energy transition and translates this into policy implications based on strategic timing for sustainable industrial development.

Table 1. PV-generated electricity, carbon intensities of the grid, and carbon intensity of the coal-fired electricity.

<table>
<tr><th>Year</th><th>PV-generated electricity (TWh)</th><th>Predicted carbon intensity of the grid (gCO$_2$ eq/kWh)</th><th>Carbon intensity of the coal-fired electricity (gCO$_2$ eq/kWh)</th></tr>
<tr><td>2015</td><td>40</td><td>599.40</td><td rowspan="7">750</td></tr>
<tr><td>2020</td><td>261</td><td>549.22</td></tr>
<tr><td>2030</td><td>1586</td><td>394.36</td></tr>
<tr><td>2035</td><td>2505</td><td>297.24</td></tr>
<tr><td>2040</td><td>3614</td><td>196.93</td></tr>
<tr><td>2045</td><td>4625</td><td>132.32</td></tr>
<tr><td>2050</td><td>5442</td><td>79.82</td></tr>
</table>

Note: The PV-generated electricity and carbon intensities of the grid in China under APS scenario are based on IEA. The carbon intensity of the coal-fired electricity is taken from the GHG Protocol (WRI, 2007).

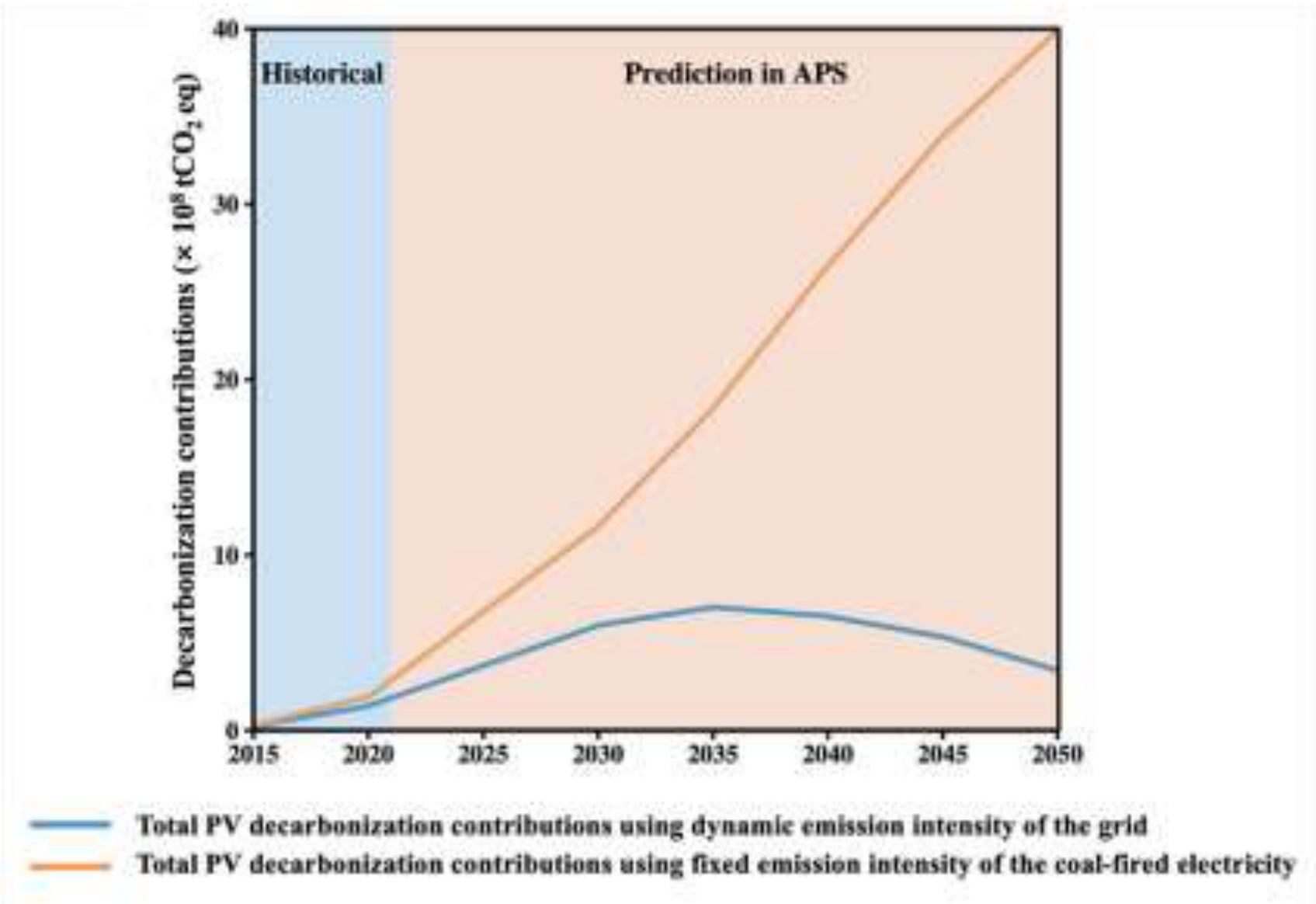

Figure 1. The decarbonization contributions of the PV system at China's national level in APS.

We have now revised the text as follows.

Introduction: *"However, this perspective reflects merely a reduction in responsibility rather than an explicit recognition of contribution. This gap rises a fundamental question: how large are the decarbonization contributions of PV manufacturers? Furthermore, even if the decarbonization contributions of PV manufacturers has been recognized, they should not be assumed to increase indefinitely. This is because the avoided emissions enabled by PV depend on the carbon intensity of the electricity that PV replaces. As power systems become increasingly decarbonized, the marginal contribution of upcoming PV deployment may narrow. This raises a further question: how will the decarbonization contributions of PV manufacturers vary over time?"*

References:

1. Banerjee, S., Aamir Khan, M. & Iftikhar ul Husnain, M. Searching appropriate system boundary for accounting India's emission inventory for the responsibility to reduce carbon emissions. *Journal of Environmental Management* **295**, 112907 (2021).
2. Davis, S. J., Dumit, A., Li, M., Maldonado, Y., Steffen, M., Stevenson, M. *et al.* The importance of multiregional accounting for corporate carbon emissions. *Nature Communications* **17**, 1014 (2025).

3. Forster, E. J., Healey, J. R., Newman, G. & Styles, D. Circular wood use can accelerate global decarbonisation but requires cross-sectoral coordination. *Nature Communications* **14**, 6766 (2023).
4. Greenhouse Gas Protocol. Scope 3 Frequently Asked Questions. *World Resources Institute (WRI), World Business Council for Sustainable Development (WBCSD)*, (2022).
5. Klaassen, L. & Stoll, C. Harmonizing corporate carbon footprints. *Nature Communications* **12**, 6149 (2021).
6. Wang, L., Qiu, T., Zhang, M., Cao, Q., Qin, W., Wang, S. *et al.* Carbon emissions and reduction performance of photovoltaic systems in China. *Renewable and Sustainable Energy Reviews* **200**, 114603 (2024).
7. WRI & WBCSD. Product Life Cycle Accounting and Reporting Standard. *World Resources Institute (WRI), World Business Council for Sustainable Development (WBCSD). 2011. Product Life Cycle Accounting and Reporting Standard*, Washington, DC (2011).
8. WRI. Guidelines for Quantifying GHG Reductions from Grid-Connected Electricity Projects. *World Resources Institute (WRI)*, Washington, DC (2007).
9. Ye, Q., Krol, M. S., Shan, Y., Schyns, J. F., Berger, M. & Hubacek, K. Allocating capital-associated CO_2 emissions along the full lifespan of capital investments helps diffuse emission responsibility. *Nature Communications* **14**, 2727 (2023).
10. Zhang, Z., Li, J. & Guan, D. Value chain carbon footprints of Chinese listed companies. *Nature Communications* **14**, 2794 (2023).

Comment 3:

I do note that a range of points have been raised by other reviewers already. I have therefore aimed to focus my comments on areas that I do not believe have been addressed or discussed in previous rounds. Overall, while I am open to being convinced, the PDC modelling on its own is not sufficient to demonstrate the soundness of the policy implications. While there is an argument to be made that the policies implied by the authors are desirable, the current paper's discussion section fails to make this argument in a compelling way, with meaningful reference with the very extensive literatures on resource economics and industrial policy that are instrumental to the interpretation of the paper's findings.

Response:

This is a very rigorous and important points. We agree that the previous version did not make the policy implications of the PDC framework sufficiently compelling, particularly comparing with the extensive literature on resource economics, industrial policy, and low-carbon technological innovation. In response, we have substantially strengthened the Discussion section. The revised manuscript now makes clearer that the PDC framework is not intended to call for unconditional support of PV manufacturing, but rather an critical tool for determining when support should be justified and when it should be altered or even removed according to the strategic timing — the predicted time point that PV manufacturers reach their maximum contributions.

Specifically, we have strengthened the policy discussion in three ways.

(1) We clarify that policy design should be more detailed and targeted, going beyond output expansion merely to include support for innovation in PV module manufacturing technologies (Peters et al., 2022; Quagraine et al., 2026; Willis et al., 2026), the reshaping of the spatial configuration (Chen et al., 2023), and the removal of bottlenecks in upstream material supply (IEA, 2022). Such measures would ensure that policy support for PV manufacturing is aligned with reducing the risks of carbon lock-in and innovation crowding-out (Herman et al., 2025). More importantly, technological innovation in manufacturing directly reduces energy consumption, economic costs, and carbon emissions at the production stage, thereby shortening the payback time and increasing the magnitude and duration of net decarbonization contributions. From this perspective, support for PV manufacturing is not aimed at sustaining high-emission production, but at enabling earlier and greater net decarbonization contributions through accelerated technological improvement.

(2) By moving away from fixed emission benchmarks and adopting a dynamic, system-based perspective, our framework provides a more realistic and scientifically grounded basis for policy design under evolving energy systems. We define the concept of strategic timing and replace a fixed emission intensity from the grid or coal-fired electricity—the way suggested by the GHG Protocol—with a dynamically changing intensity from the grid as benchmark. By doing so, we manage to avoid the impression that PV’s decarbonization contributions would increase infinitely.

(3) We explicitly acknowledge the risks associated with overcapacity and misallocation, and position our PDC framework as a tool for identifying when such risks may occur.

As the grid becomes progressively decarbonized, the marginal decarbonization contribution of PV correspondingly declines, enabling us to identify a peak in contributions—defined as the strategic timing—beyond which marginal benefits decrease, and thus providing a more realistic and policy-relevant basis for determining when supportive measures should be moderated while avoiding misleading signals from fixed benchmarking approaches. However, fully resolving them is beyond the scope of this study. To our knowledge, overcapacity can be understood as a mismatch between supply and demand. Our study does not seek to explain all causes of overcapacity, nor is this a problem that can be simply resolved within the scope of this study. We would be pleased to see our framework inspire further refinements and alternative approaches proposed by other researchers to benefit not only PV industries but also other renewable industries for a sustainable future.

We have now revised the text as follows.

Discussion: *"Note that policy support for PV manufacturing should not be simply understood as the removal of constraints on upstream emissions to increase production. Rather, it should be more detailed and targeted by going beyond output expansion merely to include support for innovation in PV module manufacturing technologies (Peters et al., 2022; Quagraine et al., 2026; Willis et al., 2026), the reshaping of the spatial configuration (Chen et al., 2023), and the removal of bottlenecks in upstream material supply (IEA, 2022). Such measures would ensure that policy support for PV manufacturing is aligned with reducing the risks of lock-in emissions from incumbent high-carbon production and avoiding innovation crowding-out by refocusing incentives toward low-carbon technologies (Herman et al., 2025; Liu et al., 2025; Wu et al, 2026). More importantly, technological innovation facilitates shortening the payback time by reducing energy consumption, economic costs, and carbon emissions at the production stage, thereby increasing the magnitude and duration of the net decarbonization contributions in manufacturing. In this sense, support for PV manufacturing is not aimed at sustaining high-emission production, but at enabling earlier and greater net decarbonization contributions through accelerated technological improvement."*

References:

1. Chen, S., Lu, X., Nielsen, C. P., McElroy, M. B., He, G., Zhang, S. *et al*. Deploying solar photovoltaic energy first in carbon-intensive regions brings gigatons more carbon mitigations to 2060. *Communications Earth & Environment* **4** (2023).

2. Herman, K. S., Hall, J. K., Sovacool, B. K., & Iskandarova, M. The industrial decarbonization paradigm: Carbon lock-in or path renewal in the United Kingdom? *Ecological Economics* **235**, 108628 (2025).
3. IEA. Solar PV Global Supply Chains Analysis. *International Energy Agency (IEA)*, Paris (2022).
4. Liu, Z., Fang, K., Xu, A., Zhang, Z., Zhu, J., Wang, H. *et al.* CO(2) Sequestration Technologies May Undermine China's Sustainable Development Goals through the Climate-Energy-Air-Health Cascade. *Environ Sci Technol* **59**, 23–34 (2025).
5. Peters, I. M., Hauch, J. A. & Brabec, C. J. The role of innovation for economy and sustainability of photovoltaic modules. *iScience* **25**, 105208 (2022).
6. Quagraine, K. A., Lynas, M. & Ellis, E. C. As we breach 1.5 degrees C, we must replace temperature limits with clean-energy targets. *Nature* **649**, 1103–1106 (2026).
7. Willis, B. L., Rigby, O. M., Pain, S. L., Grant, N. E., Murphy, J. D., Bonilla, R. S. *et al.* Maximising environmental savings from silicon photovoltaics manufacturing to 2035. *Nature Communications* **17** (2026).
8. Wu, Z., Wang, X., Tan, Y., Wu, X. & Fang, K. A typology of energy scaling in Chinese cities and its implication for sustainable urban transitions. *Sustainable Cities and Society* **138** (2026).

Specific Comments

The introduction makes clear that this approach is quite specific to China's domestic policy framework and incentives for producers, since there are no other comparable producers globally.

Response:

We gratefully thank you for this important comment. We agree that our analysis is grounded in the context of China’s domestic policy framework. Given this high degree of global reliance on China’s PV manufacturing capacity (dominant share of global mass production for PV cells and modules up to 84%), we believe that insights derived from the China-specific context have broader relevance to the global energy transition.

We have now revised the text as follows.

Introduction: *"Given the high degree of global reliance on China's PV manufacturing capacity, findings derived from the China-specific context would have global implications for energy transition and can be extended to other renewables."*

Comment 4:

The economic theory underpinning the interpretation of the results in this paper is not very clear. The authors note that restrictions on high-emitting upstream enterprises is causing them to throttle production, particularly around the end of reporting periods. This is a well-known problem in China's current GHG management regime. However they do not convincingly establish that this is creating a supply shortage in PV panels, nor that it is desirable to remove restrictions on upstream suppliers' emissions if doing so disincentivises innovation in lower-carbon manufacturing. Again, it would be fruitful to engage more thoroughly with the literature on innovation in low-carbon technologies in order to explain why this theoretical trade-off is desirable. The authors could plausibly do so with reference to the payback period / decarbonisation contribution framework developed here: namely, how high would the decarbonisation contribution value have to be for the downsides of subsidising high-carbon upstream production to be overcome?

Response:

We agree that policy support for PV manufacturing should not be interpreted simply as relaxing constraints on upstream emissions to expand the output of PV modules. In the revised manuscript, we explicitly emphasize that such support should be understood more broadly, particularly in terms of promoting technological innovation in PV manufacturing (Peters et al., 2022; Quagraine et al., 2026; Willis et al., 2026), which is essential for achieving long-term decarbonization. We have also discussed how our study could impact on the payback time and decarbonization contributions. In short, technological innovation can increase net decarbonization contribution by shortening the payback time. This implies that only when these contributions reach a sufficiently large level can the potential downsides of supporting high-carbon upstream production be effectively overcome.

Specifically, although China's carbon reduction goals may promote the technological transition of the PV industry in the long term, strict policies, particularly those in short term, may make upstream manufacturers face potential disruptions, such as difficulties in capacity expansion and increased production costs (Helveston and Davidson, 2022).

In some cases, key upstream suppliers within the PV value chain (e.g. PV glass manufacturers) are not explicitly recognized as part of the PV industry and may therefore be subject to regulatory treatment that does not fully reflect their role in enabling downstream decarbonization. While these effects may not necessarily result to immediate supply shortages, they could weaken firms' ability and incentives to invest in low-carbon innovation (Quagraine et al., 2026; Zhang et al., 2023). Therefore, quantifying the decarbonization contributions of PV manufacturers by this study is valuable for policymakers by stressing the critical role of the PV production stage. No doubt that there would be no subsequent deployment and operation of PV system without production, and thus PV manufacturing serves as a driver behind China's and the global energy transition.

Within the PDC framework, the role of innovation can be more explicitly interpreted through its impact on payback time and decarbonization contributions. Specifically, decarbonization contributions are divided into two phases: (1) the period before payback, during which lifecycle energy use, emissions, and costs are offset. These contributions are not for allocation; and (2) the period after payback, during which net decarbonization contributions are generated and can be allocated across the value chain. Technological innovation in manufacturing directly reduces energy consumption, economic costs, and carbon emissions at the production stage, thereby shortening the payback time and increasing the magnitude and duration of net decarbonization contributions. From this perspective, support for PV manufacturing is not aimed at sustaining high-emission production, but at enabling earlier and greater net decarbonization contributions through accelerated technological improvement.

We have now revised the text as follows.

Introduction: *"In some cases, key upstream suppliers within the PV value chain (e.g., PV glass manufacturers) are not explicitly recognized as part of the PV industry and may therefore be subject to regulatory treatment that does not fully reflect their role in enabling downstream decarbonization. Stringent short-term policies may disrupt upstream manufacturers by constraining capacity expansion and increasing production costs (Helveston et al., 2022; Kozul-Wright, 2023; Liang and You, 2023; Nijsse et al., 2023), thereby weakening incentives for innovation (Quagraine et al., 2026; Zhang et al., 2023), and ultimately hindering the development of the PV industry and even the energy transition (Ou et al., 2024)."*

Discussion: *"Note that policy support for PV manufacturing should not be simply understood as the removal of constraints on upstream emissions to increase production. Rather, it should be more detailed and targeted by going beyond output expansion merely to include support for innovation in PV module manufacturing technologies (Peters et al., 2022; Quagraine et al., 2026; Willis et al., 2026), the reshaping of the spatial configuration (Chen et al., 2023), and the removal of bottlenecks in upstream material supply (IEA, 2022). Such measures would ensure that policy support for PV manufacturing is aligned with reducing the risks of lock-in emissions from incumbent high-carbon production and avoiding innovation crowding-out by refocusing incentives toward low-carbon technologies (Herman et al., 2025; Liu et al., 2025; Wu et al, 2026). More importantly, technological innovation facilitates shortening the payback time by reducing energy consumption, economic costs, and carbon emissions at the production stage, thereby increasing the magnitude and duration of the net decarbonization contributions in manufacturing. In this sense, support for PV manufacturing is not aimed at sustaining high-emission production, but at enabling earlier and greater net decarbonization contributions through accelerated technological improvement."*

"Such measures could help reduce the lifecycle carbon intensity of PV-generated electricity, thereby maintaining or even expanding the mitigation advantage over grid electricity. By doing so, the PV manufacturing can continue to support energy transition, not merely through capacity expansion, but through low-carbon production."

References:

1. Chen, S., Lu, X., Nielsen, C. P., McElroy, M. B., He, G., Zhang, S. *et al*. Deploying solar photovoltaic energy first in carbon-intensive regions brings gigatons more carbon mitigations to 2060. *Communications Earth & Environment* **4** (2023).
2. Helveston, J. P., He, G. & Davidson, M. R. Quantifying the cost savings of global solar photovoltaic supply chains. *Nature* **612**, 83–87 (2022).
3. Helveston, J. P., He, G. & Davidson, M. R. Quantifying the cost savings of global solar photovoltaic supply chains. *Nature* **612**, 83–87 (2022).
4. Herman, K. S., Hall, J. K., Sovacool, B. K., & Iskandarova, M. The industrial decarbonization paradigm: Carbon lock-in or path renewal in the United Kingdom? *Ecological Economics* **235**, 108628 (2025).
5. IEA. Solar PV Global Supply Chains Analysis. *International Energy Agency (IEA)*, Paris (2022).

6. Kozul-Wright, R. China's policy strategies for green low-carbon development: Perspective from South-South cooperation. *United Nations Conference on Trade and Development (UNCTD)*, (2023).
7. Liang, H. & You, F. Reshoring silicon photovoltaics manufacturing contributes to decarbonization and climate change mitigation. *Nature Communications* **14**, 1274 (2023).
8. Liu, Z., Fang, K., Xu, A., Zhang, Z., Zhu, J., Wang, H. *et al.* CO(2) Sequestration Technologies May Undermine China's Sustainable Development Goals through the Climate-Energy-Air-Health Cascade. *Environ Sci Technol* **59**, 23–34 (2025).
9. Nijsse, F., Mercure, J. F., Ameli, N., Larosa, F., Kothari, S., Rickman, J. *et al.* The momentum of the solar energy transition. *Nature Communications* **14**, 6542 (2023).
10. Ou, Y., Hsiao, C. Y.-L. & Chui, C. M. How does the supply chain market respond to policy shocks? Evidence from solar photovoltaic sectors in China. *Renewable Energy* **232**, 121129 (2024).
11. Peters, I. M., Hauch, J. A. & Brabec, C. J. The role of innovation for economy and sustainability of photovoltaic modules. *iScience* **25**, 105208 (2022).
12. Quagraine, K. A., Lynas, M. & Ellis, E. C. As we breach 1.5 degrees C, we must replace temperature limits with clean-energy targets. *Nature* **649**, 1103–1106 (2026).
13. Willis, B. L., Rigby, O. M., Pain, S. L., Grant, N. E., Murphy, J. D., Bonilla, R. S. *et al.* Maximising environmental savings from silicon photovoltaics manufacturing to 2035. *Nature Communications* **17** (2026).
14. Wu, Z., Wang, X., Tan, Y., Wu, X. & Fang, K. A typology of energy scaling in Chinese cities and its implication for sustainable urban transitions. *Sustainable Cities and Society* **138** (2026).
15. Zhang, X., Zhu, Q., Li, X. & Pan, Y. The impact of government subsidy on photovoltaic enterprises independent innovation based on the evolutionary game theory. *Energy* **285**, 129385 (2023).

Comment 5:

Indeed, the authors are currently arguing that it is always desirable, up to the maximum of the decarbonisation contribution curve (which varies in time according to the scenario and boundaries used), to provide policy support to PV manufacturing so that progress along pathways to net zero is not unnecessarily inhibited. This may be a defensible position, but in its current form the article should do more to base it in

ongoing theory and evidence on how low-carbon industrial policy should be designed and why.

Since 2002, China has seen episodes of periodic, and in some cases structural, overproduction of industrial goods including steel, and of finished products including buildings and, arguably, PV panels. The authors mention this in the global context but do not address it with sufficient rigour in the domestic context, apparently dismissing it as a concern on the basis that China plans to quintuple installed solar & wind capacity between 2020 and 2035. Again, this may be a defensible position but it must be laid out in more detail. Furthermore, the authors do not appear to consider that overproduction or overinstallation of PV itself decreases the marginal decarbonisation contribution of each panel, such that their proposed policies, to the extent that they generate overproduction, would reduce the contribution that they are trying to highlight. This highlights the risks of the 'blunt' approach to stimulating PV production proposed by the authors in (a) promoting the inefficient use of resources in the short term; (b) crowding out less carbon-intensive production within China and elsewhere. In other words, the authors do not engage properly with the risk that their proposals may well incentivise the embedding of a structural imbalance in upstream PV manufacturing that encourages incumbent high-carbon production and discourages innovation in either decarbonising the upstream element of the PV supply chain or developing entirely new technologies that complement or substitute for silicone PV.

Response:

We appreciate this highly insightful comment. We are sorry for the previous version that leaves the impression that we advocate unconditional support for PV manufacturing. We fully agree with you that overproduction may reduce the marginal decarbonization contributions of each additional PV panel. This is exactly why we define the concept of strategic timing and replace a fixed emission intensity from the grid or coal-fired electricity—the way suggested by the GHG Protocol—with a dynamically changing intensity from the grid as benchmark. By doing so, we manage to avoid the impression that PV’s decarbonization contributions would increase infinitely. Specifically, this strategic timing occurs in 2035 for China under the APS scenario and in 2030 at the global level under the NZE scenario. After the strategic timing, we observe a decline in these contributions as the carbon intensity of grid electricity reaches a significantly low level that is very close to the PV-generated electricity. In the manuscript, we have argued that more stringent regulations and policies should be applied to the PV manufacturing industry after the strategic timing

to potential overproduction and resource waste, such as phasing out inefficient capacity and enforcing stricter standards on carbon emissions. We believe that our findings are able to inform industrial policy design by eliminating misleading signals for infinite capacity expansion.

We have further proposed policy recommendations to effectively support the PV manufacturing. Overall, our policy recommendations are not a "blunt" expansionary strategy, but a phased and dynamically adjusted one. Both on the China's and global levels, policy intensity and instruments should be adjusted in line with the strategic timing identified in our paper to prevent potential issues such as overproduction and resource waste within the PV industry. We also acknowledge the risk that poorly designed policies may reinforce carbon-intensive upstream production or crowd out innovation in supply-chain decarbonization as well as in complementary or substitute technologies. We have therefore revised the manuscript to clarify that policy design should be more detailed and targeted, going beyond output expansion merely to include support for innovation in PV module manufacturing technologies (Peters et al., 2022; Quagraine et al., 2026; Willis et al., 2026), the reshaping of the spatial configuration (Chen et al., 2023), and the removal of bottlenecks in upstream material supply (IEA, 2022). Such measures would ensure that policy support for PV manufacturing is aligned with reducing the risks of carbon lock-in and innovation crowding-out (Herman et al., 2025).

While policy recommendations may be idealized in theory, the real-world context is much more complex. We acknowledge that China's PV industry has indeed experienced periodic, and in some cases structural overcapacity. However, this cannot be attributed to any single cause, but instead reflects the interaction of multiple drivers that including demand fluctuations (Falk and Hagsten, 2025), underdeveloped electricity market (Guo et al., 2020), lagging grid and storage infrastructure (Nijsse et al., 2023), insufficient transmission capacity (Chen et al., 2021), changing trade conditions (Cui et al., 2025), geopolitical factors (Markard et al., 2020), etc. In this sense, overcapacity can be understood as a misalignment between supply expansion and demand absorption. Our study does not seek to explain all causes of overcapacity, nor is this a problem that can be simply resolved within the scope of this study.

We do not expect our proposed PDC framework allocating decarbonization contributions to manufacturers to be the only and final solution, but rather a starting point that could lead to a broader discussion among scholars of resource economics and policy. We would be pleased to see our framework inspire further refinements and

alternative approaches proposed by other researchers to benefit not only PV industries but also other renewable industries for a sustainable future.

We have now revised the text as follows.

Introduction: *"In some cases, key upstream suppliers within the PV value chain (e.g., PV glass manufacturers) are not explicitly recognized as part of the PV industry and may therefore be subject to regulatory treatment that does not fully reflect their role in enabling downstream decarbonization. Stringent short-term policies may disrupt upstream manufacturers by constraining capacity expansion and increasing production costs (Helveston et al., 2022; Kozul-Wright, 2023; Liang and You, 2023; Nijsse et al., 2023), thereby weakening incentives for innovation (Quagraine et al., 2026; Zhang et al., 2023), and ultimately hindering the development of the PV industry and even the energy transition (Ou et al., 2024)."*

Discussion: *"Note that policy support for PV manufacturing should not be simply understood as the removal of constraints on upstream emissions to increase production. Rather, it should be more detailed and targeted by going beyond output expansion merely to include support for innovation in PV module manufacturing technologies (Peters et al., 2022; Quagraine et al., 2026; Willis et al., 2026), the reshaping of the spatial configuration (Chen et al., 2023), and the removal of bottlenecks in upstream material supply (IEA, 2022). Such measures would ensure that policy support for PV manufacturing is aligned with reducing the risks of lock-in emissions from incumbent high-carbon production and avoiding innovation crowding-out by refocusing incentives toward low-carbon technologies (Herman et al., 2025; Liu et al., 2025; Wu et al, 2026). More importantly, technological innovation facilitates shortening the payback time by reducing energy consumption, economic costs, and carbon emissions at the production stage, thereby increasing the magnitude and duration of the net decarbonization contributions in manufacturing. In this sense, support for PV manufacturing is not aimed at sustaining high-emission production, but at enabling earlier and greater net decarbonization contributions through accelerated technological improvement."*

"Such measures could help reduce the lifecycle carbon intensity of PV-generated electricity, thereby maintaining or even expanding the mitigation advantage over grid electricity. By doing so, the PV manufacturing can continue to support energy transition, not merely through capacity expansion, but through low-carbon production."

References:

1. Chen, S., Lu, X., Nielsen, C. P., McElroy, M. B., He, G., Zhang, S. *et al*. Deploying

solar photovoltaic energy first in carbon-intensive regions brings gigatons more carbon mitigations to 2060. *Communications Earth & Environment* **4**, 369 (2023).
2. Chen, X., Liu, Y., Wang, Q., Lv, J., Wen, J., Chen, X. *et al*. Pathway toward carbon-neutral electrical systems in China by mid-century with negative CO_2 abatement costs informed by high-resolution modeling. *Joule* **5(10)**, 2715–2741 (2021).
3. Cui, C., Lonergan, K. E. & Sansavini, G. Policy-driven transformation of global solar PV supply chains and resulting impacts. *Nature Communications* **16**, 6742 (2025).
4. Falk, M. T. & Hagsten, E. The impact of rising electricity prices on demand for photovoltaic solar systems. *Energy Economics* **147**, 108660 (2025).
5. Guo, H., Davidson, M. R., Chen, Q., Zhang, D., Jiang, N., Xia, Q. *et al*. Power market reform in China: Motivations, progress, and recommendations. *Energy Policy* **145**, 111717 (2020).
6. Herman, K. S., Hall, J. K., Sovacool, B. K., & Iskandarova, M. The industrial decarbonization paradigm: Carbon lock-in or path renewal in the United Kingdom? *Ecological Economics* **235**, 108628 (2025).
7. IEA. Solar PV Global Supply Chains Analysis. *International Energy Agency (IEA)*, Paris (2022).
8. Markard, J., Geels, F. W. & Raven, R. Challenges in the acceleration of sustainability transitions. *Environmental Research Letters* **15**, 081001 (2020).
9. Nijsse, F. J. M. M., Mercure, J., Ameli, N., Larosa, F., Kothari, S., Rickman, J. *et al*. The momentum of the solar energy transition. *Nature Communications* **14**, 7607 (2023).
10. Peters, I. M., Hauch, J. A. & Brabec, C. J. The role of innovation for economy and sustainability of photovoltaic modules. *iScience* **25**, 105208 (2022).
11. Quagraine, K. A., Lynas, M. & Ellis, E. C. As we breach 1.5 degrees C, we must replace temperature limits with clean-energy targets. *Nature* **649**, 1103–1106 (2026).
12. Willis, B. L., Rigby, O. M., Pain, S. L., Grant, N. E., Murphy, J. D., Bonilla, R. S. *et al*. Maximising environmental savings from silicon photovoltaics manufacturing to 2035. *Nature Communications* **17** (2026).
13. Zhang, X., Zhu, Q., Li, X. & Pan, Y. The impact of government subsidy on photovoltaic enterprises independent innovation based on the evolutionary game theory. *Energy* **285** (2023).

Comments

This study tried to identify and quantify the historical and potential decarbonization contributions to national and global energy transition by China's PV manufacturers based on the most up-to-date data on industrial production from three criteria. It is an interesting topic, however the innovation is not enough, especially there are some heavy mistakes on research background, the EPR policy, and research gaps. Thus, I suggest rejecting it.

1. In general, the decarbonization contribution refers to the different lifecycle stage or products, which will be neutral. If you think the manufacturers will be the contribution body, whether it will result in the misunderstanding and overstate of the producer's role or not?
2. In your paper- "China's PV manufacturers could contribute up to 74.9% of China's targets for decarbonizing in energy transition based on the announced pledges." How to calculate this contribution? Why so high? the other renewable energies only have 25% contribution in future?
3. The strategic timing is your key point, what's the definition and explanation? Before and after strategic timing, what's the differences for the PV system application? I don't think that the decreasing avoid emissions from the electricity will have large effects on the PV industry development.
4. In the introduction, I think that you have some misunderstanding on the EPR policy. EPR is not only the responsibility of producers, but also refers to other stakeholders (such as the Government and consumers). EPR is based on the principle that manufacturers (usually brand owners) have the greatest control over product design and marketing and have the greatest ability and responsibility to reduce toxicity and waste. The core goal is to realize the effective management of the product waste for improving product recovery and minimizing the environmental impact of waste. Thus, your background needs to be rewritten for the research gaps.
5. Line 206-209, "As China advances its Dual Carbon Reduction Strategy, policymakers are contemplating the imposition of more stringent regulations for those manufacturers generating carbon emissions enormously." Why it is the obstacles of the PV development?
6. In the discussion parts, it is suggested to discuss the excess production capacity of PV and the global competition challenges.
7. For the PV system, did you consider the difference between the distributed PV and centralized PV?
8. When calculating the PV developing potential in future, do you consider the saturability situation?
9. You use "1 MWp mono-crystalline PV system" as your functional units and typical PV system, please show the reasons why 1 MWp and mono-crystalline PV system.
10. More details of three scenarios should be explained in the manuscript or supporting information.

Supporting information:

11. In the table 9, what' the transportation distances from China to other countries?

12. Line 113-114, “which are 920 and -1.28×104 (STEPS) and -1.08×104 114 (APS) tCO2 eq from Fig. 3a”, please check the data.
13. For the table 12, not all the materials can have the 100% substitution of the primary materials. The ratio should be given for different materials.
14. Line 153-154, “The price of electricity for businesses ranges from 0.011 to 5.605 CNY/kWh”. Why so large difference differences (560 times) for the electricity prices?
15. The data source in the text and tables should show the references.

Comments

The authors haves made large efforts to revise the manuscript, and have addressed most of my questions. I still have some little suggestions as follows:

1. For previous Comment 5, Dual Carbon Reduction Strategy maybe the potential adverse effects in the short term, but from the perspective of long term, it will prompt the technology transition of the PV industry. In addition, more systematic policy consideration from the whole lifecycle time should be advised to the policy makers.
2. It is suggested that the research limitation and uncertainty (like my previous comments) should be discussed in your study.
